# Quality and Utility of European Cardiovascular and Orthopaedic Registries for the Regulatory Evaluation of Medical Device Safety and Performance Across the Implant Lifecycle: A Systematic Review

**DOI:** 10.34172/ijhpm.2023.7648

**Published:** 2023-07-18

**Authors:** Lotje A. Hoogervorst, Timon H. Geurkink, Anne Lübbeke, Sergio Buccheri, Jan W. Schoones, Marina Torre, Paola Laricchiuta, Paul Piscoi, Alma B. Pedersen, Chris P. Gale, James A. Smith, Aldo P. Maggioni, Stefan James, Alan G. Fraser, Rob G.H.H. Nelissen, Perla J. Marang-van de Mheen

**Affiliations:** ^1^Department of Orthopaedics, Leiden University Medical Center, Leiden, The Netherlands; ^2^Department of Biomedical Data Sciences & Medical Decision Making, Leiden University Medical Center, Leiden, The Netherlands; ^3^Division of Orthopaedic Surgery and Traumatology, Geneva University Hospitals and University of Geneva, Geneva, Switzerland; ^4^Nuffield Department of Orthopaedics, Rheumatology and Musculoskeletal Sciences, University of Oxford, Oxford, UK; ^5^Department of Cardiology, Uppsala University, Uppsala, Sweden; ^6^Directorate of Research Policy (Formerly: Walaeus Library), Leiden University Medical Center, Leiden, The Netherlands; ^7^Scientific Secretariat of the Presidency, Istituto Superiore di Sanità, Rome, Italy; ^8^Health Technology Unit B6, Directorate General for Health (DG SANTE), European Commission, Brussels, Belgium; ^9^Department of Clinical Epidemiology, Aarhus University Hospital, Aarhus, Denmark; ^10^Department of Clinical Medicine, Aarhus University, Aarhus, Denmark; ^11^Leeds Institute of Cardiovascular and Metabolic Medicine, University of Leeds, Leeds, UK; ^12^Leeds Institute for Data analytics, University of Leeds, Leeds, UK; ^13^Department of Cardiology, Leeds Teaching Hospitals NHS Trust, Leeds, UK; ^14^Botnar Research Centre and Centre for Statistics in Medicine, Nuffield Department of Orthopaedics, Rheumatology and Musculoskeletal Sciences, University of Oxford, Oxford, UK; ^15^National Institute for Health Research Oxford Biomedical Research Centre, John Radcliffe Hospital, Oxford, UK; ^16^Centro Studi ANMCO, Via Alfonso la Marmora, Florence, Italy; ^17^Department of Medical Science, Uppsala University, Uppsala, Sweden; ^18^Clinical Research Center, Uppsala University, Uppsala, Sweden; ^19^Department of Cardiology, University Hospital of Wales, Cardiff, UK

**Keywords:** Medical Device Registries, Cardiovascular, Orthopaedic

## Abstract

**Background:** The European Union Medical Device Regulation (MDR) requires manufacturers to undertake post-market clinical follow-up (PMCF) to assess the safety and performance of their devices following approval and Conformité Européenne (CE) marking. The quality and reliability of device registries for this Regulation have not been reported. As part of the Coordinating Research and Evidence for Medical Devices (CORE-MD) project, we identified and reviewed European cardiovascular and orthopaedic registries to assess their structures, methods, and suitability as data sources for regulatory purposes.

**Methods:** Regional, national and multi-country European cardiovascular (coronary stents and valve repair/replacement) and orthopaedic (hip/knee prostheses) registries were identified using a systematic literature search. Annual reports, peer-reviewed publications, and websites were reviewed to extract publicly available information for 33 items related to structure and methodology in six domains and also for reported outcomes.

**Results:** Of the 20 cardiovascular and 26 orthopaedic registries fulfilling eligibility criteria, a median of 33% (IQR: 14%-71%) items for cardiovascular and 60% (IQR: 28%-100%) items for orthopaedic registries were reported, with large variation across domains. For instance, no cardiovascular and 16 (62%) orthopaedic registries reported patient/ procedure-level completeness. No cardiovascular and 5 (19%) orthopaedic registries reported outlier performances of devices, but each with a different outlier definition. There was large heterogeneity in reporting on items, outcomes, definitions of outcomes, and follow-up durations.

**Conclusion:** European cardiovascular and orthopaedic device registries could improve their potential as data sources for regulatory purposes by reaching consensus on standardised reporting of structural and methodological characteristics to judge the quality of the evidence as well as outcomes.

## Background

 A vital mechanism for assuring safety and performance of high-risk medical devices in patients is that they are subject to systematic post-market surveillance, which includes the collection of high-quality clinical data by registries. For regulatory purposes, such post-market clinical follow-up (PMCF) is mandatory for cardiovascular devices like stents and valves and for orthopaedic devices like hip and knee implants.

 The International Medical Device Regulators Forum (IMDRF) defines a medical device registry as “an organized system with a primary aim to increase the knowledge on medical devices contributing to improve the quality of patient care that continuously collects relevant data, evaluates meaningful outcomes and comprehensively covers the population defined by exposure to particular device(s) at a reasonably generalizable scale (eg, international, national, regional, and health system).”^1^ A medical device registry is thus an unselected population-based health information system collecting large numbers of real-world data regarding safety and performance of specific devices over time, with the aim to improve the quality of patient care,^1-4^ and therefore well suited to provide clinical evidence on PMCF of devices for regulatory purposes.

 The European Medical Device Regulation (MDR) requires manufacturers to plan and conduct surveillance of their devices (see Article 83 of (EU) 2017/745^5^), but the list of sources of available information that can be used for this purpose includes “relevant specialist or technical literature, databases and/or registers” and “information, including feedbacks and complaints, provided by users, distributors and importers” (see Annex III, clause 1.1(a)).^5^ Real-world data collected by medical device registries are particularly useful as they enable continuous benchmarking across longer follow-up in many more patients than enrolled in clinical trials.^6-10^

 The utility of medical device registries organized by medical professional associations is exemplified by the case of the ‘‘Metal on Metal’’ (MoM) hip implants. Originally developed as a more durable alternative to implants with ceramic or polyethylene components, mid-term follow-up registry data of patients with MoM showed far higher revision rates when compared with other implants.^11^ The Australian Orthopaedic Association National Joint Replacement Registry identified these implants as having an outlier performance, three years before their withdrawal from the market in 2010.^12-14^ For cardiovascular diseases, device registries have provided important insights on the safety of coronary stents, by documenting increased rates of low-frequency events such as stent thrombosis with specific stent platforms.^15,16^

 Principles have been proposed by regulators to evaluate whether the quality of clinical data on medical devices meets the scientific standards to be used for PMCF. They include coverage (ie, extent of participation in data collection), completeness (ie, data used in analyses are consistently captured), accuracy (ie, data recorded is an accurate reflection of the healthcare event), consistency (ie, uniformity in following the same procedures for data capture), integrity (ie, consistent recording of unique identification of medical devices), and reliability (ie, reproducibility of data elements).^1^ Specific criteria have not been proposed, however, and it is therefore unknown if existing medical device registries in Europe would allow manufacturers to meet the MDR requirements to an acceptable standard. As part of the Coordinating Research and Evidence for Medical Devices (CORE-MD) project, this systematic review therefore aims to: (1) identify current European cardiovascular and orthopaedic medical device registries, and (2) review these registries by 33 items that related to their structures, methodologies, and quality of data.

## Methods

 This systematic review was conducted according to the Preferred Reporting Items for Systematic Reviews and Meta-Analyses (PRISMA) 2020 guidelines,^17^ and it was registered in the Center for Open Science in October 2021 (https://osf.io/7yuwx/) prior to data collection.

###  Search Strategy 

 A previous study identified European registries on implantable medical devices^18^ from which we adapted and updated its search strategy in order to identify new registries and expand the list of registries for this systematic review. Eight literature libraries (Centre for Reviews and Dissemination York, Cochrane library, Embase, Emcare, Google Scholar, Medline, PubMed, and Web of Science) were searched for publications between January 1, 2013 and July 7, 2021, using a systematic search strategy (Supplementary file 1) created by a librarian (JWS). References were imported to EndNote (Version X9, Clarivate Analytics, Philadelphia, the USA) which was used to remove duplicate publications, and subsequently exported to the web application Rayyan (Doha, Qatar)^19^ which was used for study selection.

###  Study Selection 

 Two reviewers (LAH and THG) independently screened titles and abstracts and then independently assessed eligibility of full texts. Discrepancies were resolved by discussion. If consensus could not be reached, the senior researcher (PJMvdM) was consulted for a decisive vote. Studies were included firstly if they described a European regional, national, or multi-country cardiovascular medical device registry in which data were captured on coronary stents and/or on percutaneous or surgical valve repair or replacement. We focused on coronary artery stents as they are commonly used high-risk devices with a low frequency of adverse events so that a large number of patients is needed to detect safety issues, and on valve prostheses because there are many new devices for which guidance is needed on benchmarking safety and performance. Secondly, we also included European registries capturing data on hip and/or knee prostheses since they are the most common orthopaedic high-risk devices. By applying these criteria and by excluding multicenter studies, we complied with the IMDRF definition of a registry,^1^ which is particularly relevant to evaluate implant performance in the entire population receiving such a device in daily practice, rather than in selected (high-performing) centers. Additional inclusion criteria were: (*i*) an active/accessible website at the time of study collection; or (*ii*) at least one publication and/or annual report containing registries’ data between 2013 and 2021. We defined an “active registry” as a registry that published at least one annual report and/or peer-reviewed paper containing registries’ data, during or later than 2018. The reason for making a distinction between “active” and “non-active” registries is to give a better estimate regarding the number of registries able to contribute evidence for regulatory purposes in practice. In addition, “active” registries may also report the structural and methodological characteristics determining the quality of the data more consistently. No language restriction was applied. Data were extracted from any peer-reviewed publication(s) that described the registries’ structure and methodology, and combined with data from the most recent published annual report(s) (if available) and/or registries’ website (if available). To identify any more registries that were not yet included in this review, the references in publications and annual reports were checked, and clinical experts were consulted (five for the cardiovascular and eight for the orthopaedic field). For orthopaedic registries, we also checked the list on the EFORT — Network of Orthopaedic Registries of Europe (NORE) —website (https://efortnet.efort.org/nore-map/#/nore/map-all).

###  Data Extraction and Analysis

 Based on the literature including a study reporting best-practice recommendations,^20^ LAH and PJMvdM developed a list of items that could be used to assess registries’ structures and methodological characteristics, reflecting the previously mentioned principles^1^ and therefore relevant to judge the quality of registry data for regulatory purposes as required by the MDR. These were sent to 13 experts in the cardiovascular (n = 7) and/or orthopaedic (n = 6) fields, for feedback and suggestions of relevant additional items. Consensus was reached on a total of 33 quality items covering six domains: (1) Identification (6 items) to understand which population the registry intends to describe; (2) Maturity (3 items) to contextualize the numbers of procedures and extent to which longer-term outcomes may already be captured; (3) Governance (5 items) to enable assessment of the integrity of data; (4) Coverage, design & organisation (8 items) to reflect the aforementioned principles of coverage and consistency; (5) Data quality & completeness (4 items) to reflect the aforementioned principles of completeness and accuracy, and (6) Safety & performance (7 items) to capture reliability of data in using standard definitions to assess safety; details of each item are given in Box 1. Data were also collected on: (*i*) the number of peer-reviewed publications since foundation of the registry, as an indicator of scientific utility; (*ii*) the number of included manufacturers and the total number of patients/procedures, to indicate the average experience with a specific device, that would potentially be relevant when assessing the performance based on a minimum sample size to obtain reliable estimates, and (*iii*) reported outcomes, including definitions and durations of follow-up.

**Box 1. Description of the Items in Each Domain That Were Extracted for Each Registry**

**Identification**

1. Class of device (cardiovascular registries – stents/cardiovascular registries – valves/cardiovascular registries – combined)/(orthopaedic arthroplasty registries – combined/orthopaedic arthroplasty registries – hips/orthopaedic arthroplasty registries – knees)

2. Name of registry

3. Initial motivation/goal to set up the registry

4. Country (country or countries in which the registry is conducted)

5. Design (regional/national/multi-country)

6. Website (available yes/no)

**Maturity**

7. Starting year (year of first patient/procedure included)

8. First annual report (year of publication)

9. Most recent (or last, if registry no longer active) annual report (year of publication)

**Governance**

10. Mandatory (if mandatory for surgeons/hospitals to submit to the registry; yes/no)

11. Patients’ consent (patients’ consent required before entering their data to the registry; required/not-required)

12. Funding (public/private/both)

13. Who can access the data and see results?

14. Privacy regulation for patients’ identifiable information (privacy regulation reported as implemented: yes/no? And if yes: how?)

**Coverage, design & organisation**

15. Number of participating hospitals and % of hospital-level coverage (defined as number of participating hospitals relative to the total number of eligible hospitals)

16. Number of patients/procedures (cumulative total in registry)

17. Number of selected patients/procedures in study population (if cumulative total in registry is not reported)

18. Annual number of patients/procedures in registry

19. Data capture and collection method (eg, electronic/manual/barcodes-industry/surgeon-reported)

20. Method of access to registry for users/members (eg, dashboard/real-time/secure server)

21. Level of information provided (data is reported at hospital/medical device/surgeon level)

22. Data linkage with other sources (eg, registry data is linked to hospital statistics/manufacturer vigilance data/national competent authority on medical devices)

**Data quality & completeness**

23. Quality assurance system defined/quality check of data (eg, data verification)

24. Missing data for patients’ characteristics reported (%) (eg, BMI, ASA classification, gender)

25. Methods for handling missing data described

26. Data completeness reported at patient/procedure-level (%)

**Safety & performance**

27. Frequency of feedback provided to surgeons/hospitals (eg, annually/quarterly)

28. Level of feedback information provided (eg, hospital/medical device/surgeon level)

29. Feedback time period (the duration of observation before assessment of performance is possible)

30. Outlier reports procedures (the type of outlier reports or procedures a registry has established and published methods to define outlier performance)

31. Accessibility of outlier results (eg, publicly available or only accessible for individual hospitals/surgeons/members)

32. Definition of an outlier (eg, using funnel plots)

33. Number of outliers identified (has this registry identified and published details of any specific hospitals/medical devices/surgeons with outlier performance?)

-----------------

Abbreviations: BMI, body mass Index; ASA, American Society of Anesthesiologists.

Using a prespecified format, publicly available data were extracted independently by LAH and THG for each registry and each item. Otherwise, items were recorded as ‘‘Not reported” (N/R). Median values (given the skewed distributions) and interquartile ranges (IQRs) were calculated for the percentage of items reported per domain and across all domains, for both cardiovascular and orthopaedic registries. Analyses were performed using Microsoft Excel (Excel version 2012, Microsoft, Redmond, the USA).

## Results

###  Literature Search

**Figure 1:**
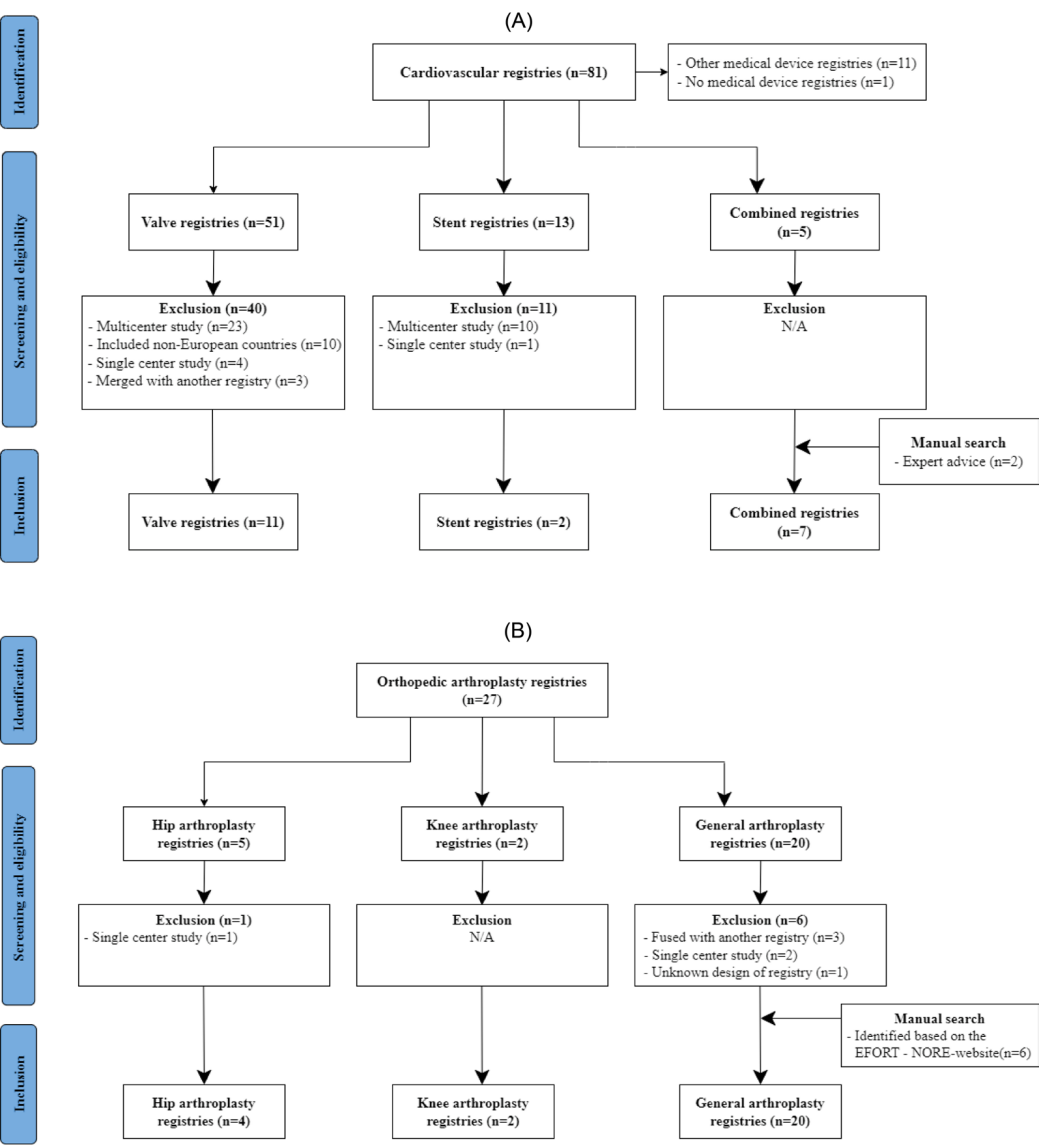
PRISMA Flowchart – (A) Cardiovascular and (B) Orthopaedic registries. Abbreviations: PRISMA, Preferred Reporting Items for Systematic Reviews and Meta-Analyses; N/A, not reported.

 The searches identified 4538 cardiovascular and 4485 orthopaedic publications, of which 1727 cardiovascular and 1360 orthopaedic publications remained after removing duplicates. Title and abstract screening identified a total of 81 cardiovascular and 27 orthopaedic registries, mentioned in publications from January 2013 to July 2021 (Figure 1). Twelve cardiovascular registries were excluded because they focused on other cardiovascular devices (eg, pacemakers) (n = 11) or no devices (n = 1) and a further 51 cardiovascular and seven orthopaedic registries were excluded during full-text screening, mostly because of reporting on a single or multicenter study, or due to registry mergers (Figure 1). Manual search identified two additional cardiovascular^21,25^ and six orthopaedic registries,^47,51,53,57,60,66^ that did not publish any peer-reviewed papers and therefore were not found in the literature search. Thus, a total of 20 cardiovascular^21-40^ and 26 orthopaedic registries^41-66^ were selected for data extraction.

###  Overall Findings

**Figure 2:**
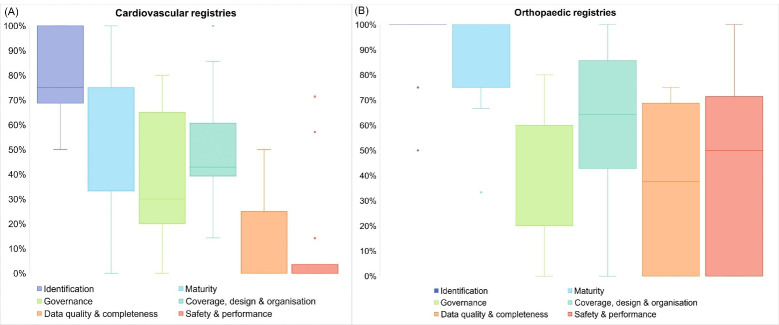
Reported Items by Cardiovascular (A) and Orthopaedic (B) Registries in Each Domain Indicating the Variation in Reporting Across Registries (with the lower end of the boxes representing the first quartile and the higher upper end the third quartile; the solid lines in the boxes representing the median values (if not visible the solid lines are at the same level as the 1st or 3rd quartile); the T-shaped whiskers the maximum or minimum values (without outliers); the individual points representing outlier values).

 Across all domains, a median of 33% (IQR 14%-71%) of the predefined 33 quality items were reported by cardiovascular registries and 60% (IQR 28%-100%) by orthopaedic registries. The highest median value was reached for the domain ‘Identification’ since almost all registries reported information on eg, the type of registry: 75% (IQR 69%-100%) for cardiovascular and 100% (IQR 100%-100%) for orthopaedic registries (Figure 2). The lowest percentages were observed for the domains ‘Data quality & completeness’ and ‘Safety & performance’; for cardiovascular registries these were respectively 25% (IQR 0%-25%) and 0% (IQR 0%-4%) and for orthopaedic registries they were 38% (IQR 0%-69%) and 50% (IQR 0%-71%) (Figure 2).

###  Domains “Identification” and “Maturity” 

**Figure 3:**
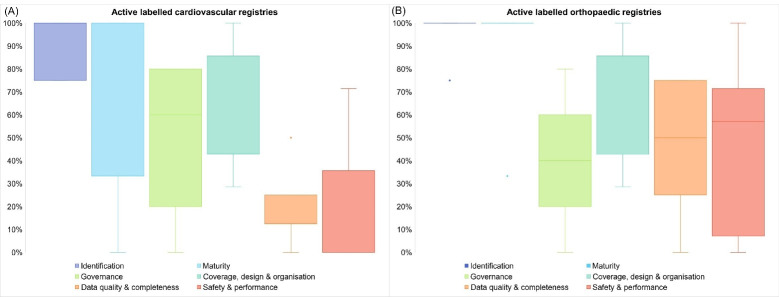
Reported Items by the Active Labelled Cardiovascular (A) and Orthopaedic (B) Registries in Each Domain Indicating the Variation in Reporting Across Registries (with the lower end of the boxes representing the 1st quartile and the higher upper end the 3rd quartile; the solid lines in the boxes representing the median values (if not visible the solid lines are at the same level as the first or third quartile); the T-shaped whiskers the maximum or minimum values (without outliers); the individual points representing outlier values).

 The majority of included registries (41 out of 46; 89%) were national registries,^21-26,28-48,51,53,54,56-66^ with only 3 (7%) regional registries^27,52,55^ and 2 (4%) multi-country registries^49,50^ (Table S1A and S1B, Supplementary files 2 and 3). The first cardiovascular registry was founded in 1978^23^ and the two most recent in 2013,^35,37^ while the first orthopaedic registry was established in 1975^65^ and the most recent in 2019.^53^ Initial motivations to set up a registry were mostly reported (by 60% of cardiovascular^21,23,25-27,29,33,35-37,39,40^ and 92% of orthopaedic registries^42-44,46-66^) and often involved ensuring patients’ safety. More orthopaedic than cardiovascular registries publish annual reports (77% versus30%), although for some registries (35%) data were last reported more than four years ago and therefore labelled as ‘‘non-active’’ (Table). Of the active registries (65%), a median of 43% (IQR 25%-80%) of the 33 quality items were reported by cardiovascular registries and 75% (IQR 41%-100%) by orthopaedic registries (Figure 3).

**Table:** Recent Activity of Included Registries

	**Published Paper(s) Containing Registries’ Data (2018 and Beyond) **	**Published Annual Report(s) Containing Registries’ Data (2018 and Beyond)**	**Active Registry**
Cardiovascular registries – combined			5 out of 7 (71%)
BCIS	No	Yes	Yes
East Denmark Heart Registry	No	No	No
German Society for Thoracic and Cardiovascular Surgery	Yes	Yes	Yes
Polish National Database of Cardiac Surgery Procedures	Yes	No	Yes
Portuguese National Registry of Intervention Cardiology	No	No	No
Spanish Cardiac Catheterization and Coronary Intervention Registry	Yes	Yes	Yes
Western Denmark Heart Registry	Yes	No	Yes
Cardiovascular registries – stents			2 out of 2 (100%)
Polish National Percutaneous Coronary Intervention Registry	Yes	No	Yes
Swedish Coronary Angiography and Angioplasty Registry	Yes	Yes	Yes
Cardiovascular registries – valves			4 out of 11 (36%)
Quality Assurance Registry on Aortic Valve Replacement	No	No	No
Austrian-TAVI Registry	No	No	No
Belgian TAVI Registry	No	No	No
Czech TAVI Registry	No	No	No
FinnValve Registry	No	No	No
FRANCE-TAVI Registry	No	No	No
German Aortic Valve Registry	Yes	No	Yes
Polish Registry of TAVI	Yes	No	Yes
Spanish Registry of Heart Valves Repair	No	No	No
Swedish Transcatheter Cardiac Intervention Registry	Yes	Yes	Yes
Swiss TAVI Registry	Yes	No	Yes
Orthopaedic arthroplasty registries – combined			14 out of 20 (70%)
Croatian Register of Endoprothesis	No	No	No
German Arthroplasty Register	Yes	Yes	Yes
Finnish Arthroplasty Register	No	Yes	Yes
Irish National Orthopaedic Register	No	Yes	Yes
Lithuanian Arthroplasty Register	Yes	No	Yes
Dutch Arthroplasty Register	Yes	Yes	Yes
Hungarian Arthroplasty Register	No	No	No
Norwegian Arthroplasty Register	Yes	Yes	Yes
Nordic Arthroplasty Register Association	Yes	No	Yes
National Joint Registry for England, Wales, Northern Ireland, the Isle of Man, and the States of Guernsey	Yes	Yes	Yes
Belgian National Arthroplasty Register	No	Yes	Yes
Catalan Arthroplasty Register	No	No	No
National Arthroplasty Registry of Slovenia	No	Yes	Yes
Italian Arthroplasty Registry	No	Yes	Yes
Emilia-Romagna Region Arthroplasty Register	Yes	Yes	Yes
Romanian National Arthroplasty Register	No	No	No
Portuguese National Arthroplasty Register	No	No	No
Scottish Arthroplasty Project Joint Registry	No	Yes	Yes
Slovakian National Arthroplasty Register	No	No	No
Swiss Arthroplasty Register	No	Yes	Yes
Orthopaedic arthroplasty registries – hips			3 out of 4 (75%)
Czech Republic Arthroplasty Register	No	No	No
French Arthroplasty Register	No	Yes	Yes
Danish Hip Arthroplasty Register	Yes	Yes	Yes
Swedish Hip Arthroplasty Register	Yes	Yes	Yes
Orthopaedic arthroplasty registries – knees			2 out of 2 (100%)
Danish Knee Arthroplasty Register	Yes	No	Yes
Swedish Knee Arthroplasty Register	Yes	Yes	Yes

Abbreviations: TAVI, Transcatheter Aortic Valve Implantation; BCIS, British Cardiovascular Intervention Society.

###  Domains “Governance” and “Coverage, Design & Organisation ”

 Mandatory enrolment of eligible patients was implemented in 8 (40%) cardiovascular^22,24,27,29,30,37,39,40^ and 12 (46%) orthopaedic registries^42,43,46,48,50,51,55,56,59,60,62,64^ (Table S2A and S2B). Few cardiovascular^21,24,27,29,35-37,39,40^ and orthopaedic^42-44,46,53,54,61-63,65^ registries have reported on their funding and few report on the patient informed consent process^24,25,27,29,31,33-37,39,40,42,44,46,48,50,54,60,63,64^ (Table S3A and S3B). The number of participating hospitals per registry varied largely, with a median of 28 (IQR 17-89) hospitals for cardiovascular registries and 71 (IQR 42-116) hospitals for orthopaedic registries (Table S4A and S4B). The proportion of all eligible hospitals that participated in the registry (ie, hospital-level coverage) was only reported by 6 (30%) cardiovascular registries,^24,26-28,31,34^ with a median hospital-level coverage of 100% (IQR 98%-100%) and by 9 (35%) orthopaedic registries,^44-46,48,52,54,60,64,65^ also with a median hospital-level coverage of 100% (IQR 95%-100%) (Table S4A and S4B).

 In general, cardiovascular registries report on studies for which selected patient groups are included, so data on the total number of patients receiving an implant were reported by only 4 (20%) registries.^21,25,29,34^ The median for stents was 12 395 (IQR 3985-201 647) and the median for valves was 2325 (IQR 861-10 479) (Table S4A and S4B). Given the regular publication of annual reports, the total and annual volume of implant procedures in orthopaedic registries was mostly reported; details were on both items was not available for 7 (27%) registries.^41,45,47,49,53,54,61^ Overall, orthopaedic registries reported on a median of 120 408 (IQR 52 391-218 445) hip implants and a median of 102 649 (IQR 51 700-194 076) knee implants (Table S4A and S4B). Data linkage with other sources — mostly national clinical databases — was reported by 8 (40%) cardiovascular^21,24,27,29,34,36,37,39^ and 14 (54%) orthopaedic registries.^42,44-46,48,50,52,54,55,60,62-65^

 Information was mostly provided on hospital and/or device-level, while in some cases also surgeon-level information was provided. There were more different types of implants in orthopaedic than in cardiovascular registries, shown by totals of 37 different manufacturers for knee implants and 63 for hip implants compared with 13 different manufacturers of valves and 11 of stents (Table S5A and S5B).

###  Domain “Data Quality & Completeness”

 None of the cardiovascular registries reported patient/procedure-level data completeness (Table S6A and S6B). Techniques to handle missing data were described in only 1 cardiovascular registry (5%),^21^ which applied a data completeness threshold (ie, a certain variable will only be analyzed if its completeness is ≥95%). Most (55%) cardiovascular registries^21,23,26,27,29,30,34-37,40^ reported on procedures to check the quality of their data, such as checking on the range and consistency of entries, and verification by audits or an external electronic tool.

 Patient/procedure-level completeness was reported by 16 (62%) orthopaedic registries,^42-46,48,50,52-55,60,62-65^ which varied from 19% for hip prostheses in the Irish National Orthopaedic Register to 98%-99% for knee prostheses in the Danish Knee Arthroplasty Register. Both registries used data linkage with national patient databases to determine patient/procedure-level completeness (Table S6A and S6B). Techniques to handle missing data were clearly described by only 1 orthopaedic registry (4%),^50^ which sent requests for missing data to each orthopaedic department once every three months. Almost half (46%) of the orthopaedic registries,^42,43,46,50,52-55,60,63-65^ reported that they implemented techniques for quality assurance of the data, which in the majority consisted of comparing registry data with national patient databases or implant databases.

###  Reported Outcomes, Definitions, and Duration of Follow-up 

 The number of peer-reviewed publications per registry in the period January 2013 – July 2021 varied, with a median of 11 (IQR 3-33) published articles among cardiovascular registries and 9 (IQR 2-45) among orthopaedic registries. A wide variety of outcomes as well as their definitions and durations of follow-up were reported by both cardiovascular and orthopaedic registries (Table S7A and S7B).

 The most frequently reported outcome in cardiovascular registries was mortality; reported by 18 (90%) registries.^21-24,26-37,39,40^ Mortality was reported using 70 different time-points, from in-hospital mortality to mortality at 21 years, the majority of registries (80%) reported on 30-day mortality.^21,22,24,27-37,39,40^ Major cardiovascular events (MACE) were reported as combined end-points by 8 (40%) registries,^21,27-29,32,36,37,40^ but with 7 different combinations of complications included in this endpoint and 7 different time intervals with most (50%) registries reporting on 1-year MACE.^28,29,36,40^ Reporting on other single outcomes also showed large variability, ranging from 3 to 40 outcome variables per registry (Table S7A and S7B).

 In orthopaedic registries, revision surgery (for any cause) was the most frequently reported outcome, reported by 20 (77%) registries.^42-44,46,48,50-60,62,63,65^ It was mostly reported as the revision rate or cumulative revision risk but at 30 different time-points up to 25 years, with the most common end-point being the 1-year revision rate which was reported by 10 registries (38%).^42,43,46,50-52,56,59,60,66^ Specific reasons for revision were reported by 19 (73%) registries,^42-44,46,48,50-57,59,60,62,63,65,66^ but these reasons for revision varied between registries (eg, infection, loosening, component failure, etc). Patient-reported outcome measurements (PROMs) were reported by 5 (19%) orthopaedic registries,^44,46,48,63,65^ with a total of 8 different scores for knee surgery patients and 11 scores for hip surgery patients. All registries measuring PROMs reported pre-operative PROMs, but post-operative PROMs were measured at different time-points up to 10-years post-operatively. Other outcomes (eg, renal failure, hip dislocation, deep venous thrombosis, etc) were inconsistently reported by 13 (50%) registries,^44,46,48,50,51,54-56,58,60,62,63,65^ the majority (77%) reported on mortality^44,50,51,55,56,58,60,62,63,65^ (Table S7A and S7B).

###  Domain “Safety & Performance”

 Public reporting on how feedback on eg, devices, hospitals, and surgeons is provided was reported by 3 (15%) cardiovascular registries^21,29,36^ (Table S8A and S8B). Managerial procedures to detect individual hospitals or specific devices using an outlier performance analysis based on benchmark thresholds was reported by 1 (5%) cardiovascular registry, the British Cardiovascular Intervention Society registry (BCIS). The outlier was defined using funnel plots, with 2 and 3 standard deviations. Outlier results regarding the timing of treatment (to assess any delay before treatment is delivered) compared between hospitals, as well as adverse outcomes per hospital, were publicly available. However, outlier reports on patients’ survival data per hospital were only disclosed confidentially to each hospital. No outlier reports for specific implants were reported by cardiovascular registries.

 Public reporting on the frequency of feedback provided was reported by 14 (54%) orthopaedic registries.^42-44,46,48,50,53,55,58,60,62,63,65,66^ Most registries report that they provide annual feedback, while 2 registries (the Irish National Orthopaedic Register and the Swiss national registry for hip and knee replacement) do so both annually and quarterly. The majority provided feedback both at the hospital level and for individual devices. Details of outlier procedures including statistical testing were reported by 8 (31%) registries, of which 3 reported solely on outlier devices,^59,60,66^ 2 solely on outlier hospitals,^58,62^ 1 on outlier devices and hospitals,^65^ and 2 on outlier devices, hospitals, and surgeons.^50,63^ Outlier procedures were mostly publicly available. No registries shared the same definition of an outlier (eg, above the 95% control limit in the funnel plot *versus *revision rates of more than twice compared to the relevant group). Overall, in all annual reports, a total of 95 total hip arthroplasty (THA) component combinations, 3 THA cups, 2 THA stems, and 24 total knee arthroplasty (TKA) implants were identified by these 8 registries as outlier implants. Overall, registries all identified different outlier implants, with only 1 outlier implant (a THA component combination) identified by more than 1 registry.

## Discussion

 In this systematic review we have evaluated structural and methodological characteristics as well as the data quality of 46 European cardiovascular and orthopaedic medical device registries, in an attempt to gain insight into the usability of these data sources for regulatory purposes. Medical device registries are potentially well suited for post-market surveillance as they may collect data from unselected patient populations and monitor safety and performance throughout the lifetime of specific devices. However, we found heterogeneity and incomplete transparency in quality items related to their structure and methodology, implying that it would be difficult currently for registries to agree upon common principles, to report the information needed by regulators to judge the quality of their data, and to collect and report comparable information across Europe.

 The European Union (EU) has regulatory requirements relating to the PMCF of medical devices.^67-69^ As stated by the MDR in Article 83, manufacturers have to set up, document, maintain, and update a post-market surveillance system for each device, in which relevant data on the quality, performance, and safety of an implant are evaluated, directly after Conformité Européenne (CE) approval and throughout the entire expected lifetime of a device.^68^ To allow for lifetime evaluation and benchmarking of implants, registries need clearly defined methods to detect outliers and to report safety concerns for specific implants, but these were reported by only 5% of the cardiovascular and 31% of the orthopaedic registries that were included in this systematic review. Even more, none of the registries used the same definition, making it difficult for manufacturers, regulators, but also patients to assess whether the device performs worse in all or only in some settings. Furthermore, four orthopaedic registries identified >100 components and combinations of implants as outliers, with only one outlier implant identified by more than one registry, which may partly result from the different definitions used from the fact that and that not all implants are used in all countries and/or regions and thereby included in the registry.

 Another way to enable benchmarking of implants across registries is to implement objective performance classification systems such as the Orthopaedic Data Evaluation Panel (ODEP). The ODEP rating provides benchmarks for orthopaedic prostheses (hip, knee, and shoulder implants) based on the number of years for which the product has been monitored and on the strength of the evidence provided by different data sources, including registry data, randomized controlled trials, peer-reviewed publications, podium presentations, and manufacturers’ in-house data sources.^70,71^ The ODEP rating can be considered as an absolute benchmark to identify if implants meet the benchmark criteria, whereas others have suggested relative benchmark approaches within a given registry eg, comparing with the best implant construct^72-75^ or with all other similar implants.^8^

 The MDR in Article 108 states that registries need to establish common principles, so that they can collect comparable information and thereby contribute to the independent evaluation of the long-term safety and performance of devices.^69^ They need to capture the same outcomes, based on the same definitions and the same durations of follow-up, before they can be used to benchmark devices and pool data for early detection of safety concerns. Current European device registries do not meet these recommended principles, however, since our systematic review showed large heterogeneity between recorded outcomes, definitions of outcome variables, and time-points for follow-up. Comparable findings were reported by a recent study of the quality of cardiac registries across all subspecialties of cardiac care, in which several registries gave explicit definitions for only a low percentage of variables.^76^ Similar findings were also observed for orthopaedic registries, with considerable heterogeneity in captured outcomes and definitions used for revision procedures.^77-79^ Another aspect to consider before outcomes across registries can be pooled, is whether registries use the same implant library to classify implants by relevant device characteristics.^80^ The European Medical Device nomenclature is a generic classification intended for this purpose, but more detailed libraries are used by registries to capture their specialty-specific characteristics as well. For orthopaedic devices for instance, the International Society of Arthroplasty Registers (ISAR) has proposed a global registry library in 2019 to ensure the same classification of orthopaedic devices across registries.^80^ Also, this problem of using different implant libraries can be solved if registries document the unique device identifier for each implant.

 In combination, these findings highlight the importance of international agreement on definitions of data and outcomes, as well as time-points used for measuring outcomes within registries. This might be reached by developing consensus frameworks to achieve common datasets that must be captured by registries^81^ such as the clinical outcome endpoints in heart failure trials created by the European Society of Cardiology Heart Failure Association, the common dataset for acute coronary syndromes and percutaneous coronary interventions created by the EuroHeart data science group, the benchmarking document for hip and knee arthroplasties by the ISAR, and the common dataset for demographics and implant survival following THA by the Nordic Arthroplasty Register Association.^82-85^

 In addition to these common data specifications, the IMDRF states that registries should include at least 95% of all patients receiving a device, to have sufficiently robust high-quality data to inform regulatory decisions.^1^ As shown in our systematic review, patient/procedure-level completeness was not reported publicly by any of the cardiovascular registries, but it was available for the majority (65%) of orthopaedic registries. Of the latter only 11 of 13 orthopaedic registries reported recent data (2018 and beyond) that reached a patient/procedure-level completeness of 95% or above. Similar findings were shown for European THA and TKA registries by Lübbeke et al, with 67% reporting patient-level completeness,^79^ and for cardiovascular registries, of which the majority had data completeness below 50% or not available.^76^

 Making it mandatory to enroll all patients in a registry would help to increase patient/procedure-level completeness.^86^ In this systematic review, however, none of the mandatory cardiovascular registries and only 75% of the mandatory orthopaedic registries reported patient/procedure-level completeness. Since completeness of patients is often checked against electronic medical records, it could also help to automatically populate certain data fields regarding patient and implant characteristics from the electronic medical records, so that less information needs to be entered by medical professionals, thereby preventing data loss as well as double data entry. However, rather than considering single items that on their own will contribute to higher quality data, the quality of the evidence provided by registry data is ultimately determined by the combination of multiple factors.

 The strength of this systematic review is its’ comprehensiveness. We updated the search strategy used by Niederländer et al,^18^ and expanded it with support from an experienced librarian. In addition, experts in the field (cardiologists and orthopaedic surgeons) were consulted, resulting in the addition of two cardiovascular registries. Furthermore, European orthopaedic registries listed on the EFORT – NORE-website were checked for their eligibility, resulting in an additional six orthopaedic registries and the completeness of included European cardiovascular registries as well as orthopaedic registries was checked by experts in the relevant field. Thus the likelihood of missing relevant registries is very low. However, some limitations remain. Firstly, we relied on publicly available information regarding registries’ structure and methodological characteristics as well as outcomes, which means that some items that we did not find may have been available if we had approached each registry directly. Therefore, the regulatory utility of the data generated by some registries may be higher than that found by this analysis. Secondly, this systematic review only focuses on cardiovascular and orthopaedic registries, because they represent the most commonly used high-risk medical devices aiming to reduce patients’ mortality and morbidity.^87^ However, the items used to determine the regulatory utility of these registries would also be applicable to other (high-risk) medical device registries.

 An overview of publicly available information, as summarized in this systematic review, demonstrates the transparency of European cardiovascular and orthopaedic medical device registries and what information could already be available for regulators. We have proposed characteristics that can be used to interpret whether the data provided by registries are of sufficient quality, and we have identified registries that had an active/accessible website at the time of study selection and/or that published at least one paper or annual report between 2013 and 2021. No data were collected since 2018 were available for 35% of these registries (shown in Table), and so there is a chance that some are no longer active and thereby would not be able to contribute evidence for regulatory purposes. However, the cut-off point to define an active registry was arbitrary and we therefore highlighted that the median of items reported across all domains among active registries was higher than items reported across all registries combined (ie, both “active” and “in-active” labelled registries).

## Conclusion

 This systematic review showed large heterogeneity and incomplete public transparency related to structure and methodological characteristics of the registries that were reviewed, which implies that it would be difficult to combine and judge the regulatory utility of data reported by registries. Effort is needed from registries to agree upon a minimum set of quality criteria that all registries should publicly report to provide information needed by regulators to judge the quality of registry data and use them for medical device safety surveillance. Developing comprehensive and trustworthy medical device registries will be tremendously valuable, not only for manufacturers to meet the requirements of the MDR for PMCF of their devices, but also for healthcare professionals and patients to support evidence-based choices of devices and contribute to their long-term safety and efficacy.

## Ethical issues

 Not applicable.

## Competing interests

 Authors declare that they have no competing interests.

## Funding

 This work was supported by the by the European Union’s Horizon 2020 Research and Innovation Programme (grant number 965246) and was part of the CORE-MD project.

## Disclaimers

 For Paul Piscoi: the information and views set out in this article are those of the authors and do not necessarily reflect the official opinion of the European Commission.

## Supplementary files


Supplementary file 1. Literature Search Strategy.

Supplementary file 2. Tables S1A-S8A.

Supplementary file 3. Tables S1B-S8B.

## References

[1] International Medical Device Regulators Forum (IMDRF). Principles of International System of Registries Linked to Other Data Sources and Tools. September 30, 2016. https://www.imdrf.org/sites/default/files/docs/imdrf/final/technical/imdrf-tech-160930-principles-system-registries.pdf.

[2] AHRQ Methods for Effective Health Care. In: Gliklich RE, Leavy MB, Dreyer NA, eds. Registries for Evaluating Patient Outcomes: A User’s Guide. Rockville, MD: Agency for Healthcare Research and Quality; 2020. 24945055

[3] International Medical Device Regulators Forum (IMDRF). Tools for Assessing the Usability of Registries in Support of Regulatory Decision-Making. March 27, 2018. https://www.imdrf.org/sites/default/files/docs/imdrf/final/technical/imdrf-tech-180327-usability-tools-n46.pdf.

[4] Lübbeke A, Carr AJ, Hoffmeyer P (2019). Registry stakeholders. EFORT Open Rev.

[5] Medical Device Coordination Group, European Commission. Regulation (EU) 2017/745: Clinical Evidence Needed for Medical Devices Previously CE Marked Under Directives 93/42/EEC or 90/385/EEC. A Guide for Manufacturers and Notified Bodies. 2020. https://ec.europa.eu/health/system/files/2020-09/md_mdcg_2020_6_guidance_sufficient_clinical_evidence_en_0.pdf.

[6] Klonoff DC, Gutierrez A, Fleming A, Kerr D (2019). Real-world evidence should be used in regulatory decisions about new pharmaceutical and medical device products for diabetes. J Diabetes Sci Technol.

[7] Tarricone R, Boscolo PR, Armeni P (2016). What type of clinical evidence is needed to assess medical devices?. Eur Respir Rev.

[8] de Steiger RN, Miller LN, Davidson DC, Ryan P, Graves SE (2013). Joint registry approach for identification of outlier prostheses. Acta Orthop.

[9] Campbell B, Wilkinson J, Marlow M, Sheldon M (2019). Generating evidence for new high-risk medical devices. BMJ Surg Interv Health Technol.

[10] Melvin T, Torre M (2019). New medical device regulations: the regulator’s view. EFORT Open Rev.

[11] Pijls BG, Meessen J, Tucker K (2019). MoM total hip replacements in Europe: a NORE report. EFORT Open Rev.

[12] de Steiger RN, Hang JR, Miller LN, Graves SE, Davidson DC (2011). Five-year results of the ASR XL acetabular system and the ASR hip resurfacing system: an analysis from the Australian Orthopaedic Association National Joint Replacement Registry. J Bone Joint Surg Am.

[13] Australian Orthopaedic Association National Joint Replacement Registry (AOANJRR). Annual Report 2007. https://aoanjrr.sahmri.com/documents/10180/42612/Annual+Report+2007.

[14] DePuy Synthes. DePuy Synthes ASR^TM^ Hip Recall Contact Information. https://www.jnjmedicaldevices.com/en-US/depuy-synthes/support-resources/asr-recall. Accessed March 18, 2022.

[15] Lagerqvist B, Carlsson J, Fröbert O (2009). Stent thrombosis in Sweden: a report from the Swedish Coronary Angiography and Angioplasty Registry. Circ Cardiovasc Interv.

[16] Capodanno D, Gori T, Nef H (2015). Percutaneous coronary intervention with everolimus-eluting bioresorbable vascular scaffolds in routine clinical practice: early and midterm outcomes from the European multicentre GHOST-EU registry. EuroIntervention.

[17] Page MJ, McKenzie JE, Bossuyt PM (2021). The PRISMA 2020 statement: an updated guideline for reporting systematic reviews. BMJ.

[18] Niederländer C, Wahlster P, Kriza C, Kolominsky-Rabas P (2013). Registries of implantable medical devices in Europe. Health Policy.

[19] Ouzzani M, Hammady H, Fedorowicz Z, Elmagarmid A (2016). Rayyan-a web and mobile app for systematic reviews. Syst Rev.

[20] Niederländer CS, Kriza C, Kolominsky-Rabas P (2017). Quality criteria for medical device registries: best practice approaches for improving patient safety–a systematic review of international experiences. Expert Rev Med Devices.

[21] British Cardiovascular Intervention Society. https://www.bcis.org.uk/.

[22] East Denmark Heart Registry.

[23] German Society for Thoracic and Cardiovascular Surgery. https://www.dgthg.de/.

[24] Polish National Database of Cardiac Surgery Procedures. https://krok.csioz.gov.pl/krok/.

[25] Portuguese National Registry of Intervention Cardiology. https://www.apic.pt/.

[26] Spanish Cardiac Catheterization and Coronary Intervention Registry.

[27] Western Denmark Heart Registry.

[28] Polish National Percutaneous Coronary Intervention Registry. https://www.orpki.cm-uj.krakow.pl/.

[29] Swedish Coronary Angiography and Angioplasty Registry. https://www.ucr.uu.se/swedeheart/start-scaar/.

[30] Quality Assurance Registry on Aortic Valve Replacement.

[31] Austrian-TAVI Registry. https://www.tavi.at/.

[32] Belgian TAVI Registry.

[33] Czech TAVI Registry.

[34] FinnValve Registry.

[35] FRANCE-TAVI Registry.

[36] German Aortic Valve Registry. https://www.aortenklappenregister.de/.

[37] Polish Registry of Transcatheter Aortic Valve Implantation.

[38] Spanish Registry of Heart Valves Repair.

[39] Swedish Transcatheter Cardiac Intervention Registry. https://www.ucr.uu.se/swedeheart/start-swentry/.

[40] Swiss TAVI Registry. https://www.swisstavi.ch/.

[41] Croatian Register of endoprothesis.

[42] German Arthroplasty Register. https://www.eprd.de/de/.

[43] Finnish Arthroplasty Register. https://www.thl.fi/far/.

[44] Irish National Orthopaedic Register. https://www.noca.ie/audits/irish-national-orthopaedic-register/.

[45] Lithuanian Arthroplasty Register. https://lsed.lt/.

[46] Dutch Arthroplasty Register. https://www.lroi.nl/.

[47] Hungarian Arthroplasty Register. https://www.ortopedtarsasag.hu/.

[48] Norwegian Arthroplasty Register. https://nrlweb.ihelse.net/.

[49] Nordic Arthroplasty Register Association.

[50] National Joint Registry for England W, Northern Ireland, the Isle of Man, and the States of Guernsey. https://www.njrcentre.org.uk/.

[51] Belgian National Arthroplasty Register. https://www.ehealth.fgov.be/nl/egezondheid/beroepsbeoefenaars-in-de-gezondheidszorg/qermidorthopride/.

[52] Catalan Arthroplasty Register.

[53] National Arthroplasty Registry of Slovenia. https://www.ob-valdoltra.si/.

[54] Italian Arthroplasty Registry. https://riap.iss.it/riap/it/.

[55] Emilia-Romagna Region Arthroplasty Register. https://www.ior.it/en/curarsi-al-rizzoli/register-orthopaedic-prosthetic-implants/.

[56] Romanian National Arthroplasty Register. https://www.rne.ro/.

[57] Portuguese National Arthroplasty Register. https://www.rpa.spot.pt/.

[58] Scottish Arthroplasty Project Joint Registry. https://www.arthro.scot.nhs.uk/.

[59] Slovakian National Arthroplasty Register. https://sar.mfn.sk/.

[60] Swiss Arthroplasty Register. https://www.siris-implant.ch/.

[61] Czech Republic Arthroplasty Register. https://www.ksrzis.cz/.

[62] Danish Hip Arthroplasty Register. https://www.dhr.dk/.

[63] Swedish Hip Arthroplasty Register. https://shpr.registercentrum.se/.

[64] Danish Knee Arthroplasty Register. https://www.danishhealthdata.com/find-health-data/Dansk-Knaealloplastik-Register/.

[65] Swedish Knee Arthroplasty Register. https://www.myknee.se/.

[66] French Arthroplasty Register. https://www.sofcot.fr/.

[67] Medical Device Coordination Group, European Commission. Post-Market Clinical Follow-Up (PMCF) Plan Template. A Guide for Manufacturers and Notified Bodies. 2020. https://ec.europa.eu/health/system/files/2020-09/md_mdcg_2020_7_guidance_pmcf_plan_template_en_0.pdf.

[68] Medical Device Coordination Group, European Commission. MDR – Article 83 – Post-Market Surveillance System of the Manufacturer. https://www.medical-device-regulation.eu/tag/mdr-article-83-post-market-surveillance-system-of-the-manufacturer/. Accessed March 28, 2022.

[69] Medical Device Coordination Group, European Commission. MDR – Article 108 – Device Registers and Databanks. https://www.medical-device-regulation.eu/tag/mdr-article-108/. Accessed March 28, 2022.

[70] Orthopedic Data Evaluation Panel (ODEP). Introducing ODEP. https://www.odep.org.uk/ODEPExplained/IntroductiontoODEP.aspx. Accessed March 21, 2022.

[71] Orthopedic Data Evaluation Panel (ODEP). Data Sources and Reliability. https://www.odep.org.uk/methodology/data-sources-and-reliability/. Accessed June 30, 2022.

[72] Wyatt M, Frampton C, Whitehouse M, Deere K, Sayers A, Kieser D (2021). Benchmarking total hip replacement constructs using noninferiority analysis: the New Zealand joint registry study. BMC MusculoskeletDisord.

[73] Deere KC, Whitehouse MR, Porter M, Blom AW, Sayers A (2019). Assessing the non-inferiority of prosthesis constructs used in hip replacement using data from the National Joint Registry of England, Wales, Northern Ireland and the Isle of Man: a benchmarking study. BMJ Open.

[74] Keurentjes JC, Pijls BG, Van Tol FR (2014). Which implant should we use for primary total hip replacement? A systematic review and meta-analysis. J Bone Joint Surg Am.

[75] Deere KC, Whitehouse MR, Porter M, Blom AW, Sayers A (2019). Assessing the non-inferiority of prosthesis constructs used in total and unicondylar knee replacements using data from the National Joint Registry of England, Wales, Northern Ireland and the Isle of Man: a benchmarking study. BMJ Open.

[76] Dawson LP, Biswas S, Lefkovits J (2021). Characteristics and quality of national cardiac registries: a systematic review. Circ Cardiovasc Qual Outcomes.

[77] Liebs TR, Splietker F, Hassenpflug J (2015). Is a revision a revision? An analysis of national arthroplasty registries’ definitions of revision. Clin OrthopRelat Res.

[78] van Schie P, Hasan S, van Bodegom-Vos L, Schoones JW, Nelissen R, Marang-van de Mheen PJ (2022). International comparison of variation in performance between hospitals for THA and TKA: is it even possible? A systematic review including 33 studies and 8 arthroplasty register reports. EFORT Open Rev.

[79] Lübbeke A, Silman AJ, Barea C, Prieto-Alhambra D, Carr AJ (2018). Mapping existing hip and knee replacement registries in Europe. Health Policy.

[80] Denissen GAW, van Steenbergen LN, Lollinga WT, Verdonschot NJJ, Schreurs BW, Nelissen R (2019). Generic implant classification enables comparison across implant designs: the Dutch Arthroplasty Register implant library. EFORT Open Rev.

[81] Batra G, Aktaa S, Wallentin L (2023). Methodology for the development of international clinical data standards for common cardiovascular conditions: European Unified Registries for Heart Care Evaluation and Randomised Trials (EuroHeart). Eur Heart J Qual Care Clin Outcomes.

[82] Zannad F, Garcia AA, Anker SD (2013). Clinical outcome endpoints in heart failure trials: a European Society of Cardiology Heart Failure Association consensus document. Eur J Heart Fail.

[83] Batra G, Aktaa S, Wallentin L (2022). Data standards for acute coronary syndrome and percutaneous coronary intervention: the European Unified Registries for Heart Care Evaluation and Randomised Trials (EuroHeart). Eur Heart J.

[84] Havelin LI, Fenstad AM, Salomonsson R (2009). The Nordic Arthroplasty Register Association: a unique collaboration between 3 national hip arthroplasty registries with 280,201 THRs. Acta Orthop.

[85] International Society of Arthroplasty Registries (ISAR). International Prosthesis Benchmarking Working Group Guidance Document: Hip and Knee Arthroplasty Devices. 2018. https://www.isarhome.org/publications.

[86] Govatsmark RES, Janszky I, Slørdahl SA (2020). Completeness and correctness of acute myocardial infarction diagnoses in a medical quality register and an administrative health register. Scand J Public Health.

[87] Fraser AG, Nelissen R, Kjærsgaard-Andersen P, Szymański P, Melvin T, Piscoi P (2021). Improved clinical investigation and evaluation of high-risk medical devices: the rationale and objectives of CORE-MD (Coordinating Research and Evidence for Medical Devices). EFORT Open Rev.

