# Supplementary material for: Quality and Utility of European Cardiovascular and Orthopaedic Registries for the Regulatory Evaluation of Medical Device Safety and Performance Across the Implant Lifecycle: A Systematic Review
Source: Int J Health Policy Manag. 2023 Jul 18;12:7648. doi: 10.34172/ijhpm.2023.7648 (PMC10702370; doi:10.34172/ijhpm.2023.7648)
Supplement: Supplementary file 1 — Literature Search Strategy. [file ijhpm-12-7648-s001.pdf]

Article title: Quality and Utility of European Cardiovascular and Orthopaedic Registries for the Regulatory Evaluation of Medical Device Safety and Performance Across the Implant Lifecycle: A Systematic Review

Journal name: International Journal of Health Policy and Management (IJHPM)

Authors' information: Lotje A. Hoogervorst[1,2]*, Timon H. Geurkink[1], Anne Lübbeke[3,4], Sergio Buccheri[5], Jan W. Schoones[6], Marina Torre[7], Paola Laricchiuta[7], Paul Piscoi[8], Alma B. Pedersen[9,10], Chris P. Gale[11,12,13], James A. Smith[14,15], Aldo P. Maggioni[16], Stefan James[5,17,18], Alan G. Fraser[19], Rob G.H.H. Nelissen[1], Perla J. Marang-van de Mheen[2]

*Correspondence to: Lotje A. Hoogervorst, Email: l.a.hoogervorst@lumc.nl

Citation: Hoogervorst LA, Geurkink TH, Lübbeke A, et al. Quality and utility of European cardiovascular and orthopaedic registries for the regulatory evaluation of medical device safety and performance across the implant lifecycle: a systematic review. Int J Health Policy Manag. 2023;12:7648. doi:10.34172/ijhpm.2023.7648

Supplementary file 1. Literature Search Strategy

Centre for Reviews and Dissemination York – Orthopaedic registries

(2013 OR 2014 OR 2015 OR 2016 OR 2017 OR 2018 OR 2019 OR 2020 OR 2021 OR 2022)

(("Hip" OR "hips" OR "Knee" OR "knees" OR "Shoulder" OR "Shoulders" OR "Ankle" OR "ankles") AND ("Prosthesis" OR "Prostheses" OR "Implants" OR "Implant" OR "replacement" OR "replacements" OR "arthroplasty" OR "arthroplast*") AND ("Register" OR "registers" OR "registry" OR "registries"))

Centre for Reviews and Dissemination York – Cardiovascular registries

(2013 OR 2014 OR 2015 OR 2016 OR 2017 OR 2018 OR 2019 OR 2020 OR 2021 OR 2022)

(("cardiac implantable electronic device" OR "artificial heart pacemaker" OR "pacemaker" OR "pacemakers" OR "Artificial Heart" OR "artificial heart" OR "artificial hearts" OR "Heart Assist Device" OR "Artificial Heart" OR "Artificial Ventricle" OR "Artificial Ventricles" OR "Heart Assist Device" OR "Heart Assist Devices" OR "Heart Assist Pump" OR "Heart Assist Pumps" OR "Vascular Assist Device" OR "Vascular Assist Devices" OR "Ventricle Assist Device" OR "Ventricle Assist Devices" OR "Ventricular Assist Device" OR "Ventricular Assist Devices" OR "Heart Valve Prosthesis" OR "Heart

Valve Prosthesis" OR "Heart Valve Prosthesis" OR "Cardiac Valve Prosthesis" OR "Cardiac Valve Prostheses" OR "Heart Prosthesis" OR "Heart Prosthesis" OR "Cardiac Prosthesis" OR "Cardiac Prostheses" OR "artificial heart valves" OR "artificial heart valve" OR "artificial valves" OR "artificial valves" OR "Implantable Defibrillator" OR "Implantable Defibrillator" OR "Implantable Defibrillators" OR "Implantable Cardioverter Defibrillator" OR "Implantable Cardioverter Defibrillators" OR "bioresorbable vascular stent" OR "bioresorbable vascular scaffold" OR "bioresorbable vascular scaffolds" OR "transcatheter aortic valve implantation" OR "transcatheter aortic valve implantation" OR "transcatheter aortic valve implant" OR "transcatheter aortic valve implants" OR "TAVI" OR "transseptal mitral valve-in-ring" OR "TMVR" OR "LAAOC") AND ("Register" OR "registers" OR "registry" OR "registries"))

OR

(("Heart" OR "cardiac") AND ("Prosthesis" OR "Prostheses" OR "Implants" OR "Implant" OR "replacement" OR "replacements") AND ("Register" OR "registers" OR "registry" OR "registries"))

Cochrane library – Orthopaedic registries

(("Hip Replacement" OR "Hip Prosthesis" OR "hip replacement" OR "hip replacement*" OR "hip arthroplasty" OR "hip arthroplast*" OR "hip prosthesis" OR "hip prosthe*" OR "THA" OR "THR" OR "hip implant" OR "hip implants" OR "Knee Replacement" OR "Knee Prosthesis" OR "knee replacement" OR "knee replacement*" OR "knee arthroplasty" OR "knee arthroplast*" OR "knee prosthesis" OR "knee prosthe*" OR "TKA" OR "TKR" OR "knee implant" OR "knee implants" OR "Shoulder Replacement" OR "Shoulder Prosthesis" OR "shoulder replacement" OR "shoulder replacement*" OR "shoulder arthroplasty" OR "shoulder arthroplast*" OR "shoulder prosthesis" OR "shoulder prosthe*" OR "shoulder implant" OR "knee implants" OR "Ankle Replacement" OR "Ankle Prosthesis" OR "ankle replacement" OR "ankle replacement*" OR "ankle arthroplasty" OR "ankle arthroplast*" OR "ankle prosthesis" OR "ankle prosthe*" OR "ankle implant" OR "ankle implants" OR (("Hip" OR "hip" OR "hips" OR "Knee" OR "knee" OR "knees" OR "Shoulder" OR "Shoulder" OR "Shoulders" OR "Ankle" OR "ankle" OR "ankles") AND ("Prosthesis" OR "Prostheses" OR "Prosthesis" OR "Implants" OR "Implant" OR "replacement" OR "replacements" OR "arthroplasty" OR "arthroplast*"))) AND ("Register" OR "register" OR "registers" OR "registry" OR "registries") AND ("European Union" OR "European Union" OR "European Community" OR "European Coal and Steel Community" OR "Common Market" OR "EEC" OR "European Economic Community" OR "European Common Market" OR "European Economic Area"

OR "Europe" OR "Albania" OR "Andorra" OR "Armenia" OR "Armenia" OR "Austria" OR "Azerbaijan" OR "Republic of Belarus" OR "Belgium" OR "Bosnia and Herzegovina" OR "Bulgaria" OR "Croatia" OR "Czech Republic" OR "Denmark" OR "England" OR "Estonia" OR "Finland" OR "France" OR "Georgia" OR "Germany" OR "Gibraltar" OR "Greece" OR "Hungary" OR "Iceland" OR "Ireland" OR "Italy" OR "Kazakhstan" OR "Kosovo" OR "Kyrgyzstan" OR "Latvia" OR "Liechtenstein" OR "Lithuania" OR "Luxembourg" OR "Malta" OR "Moldova" OR "Monaco" OR "Montenegro" OR "Netherlands" OR "Republic of North Macedonia" OR "Northern Ireland" OR "Norway" OR "Poland" OR "Portugal" OR "Romania" OR "Russia" OR "San Marino" OR "Scotland" OR "Serbia" OR "Slovakia" OR "Slovenia" OR "Spain" OR "Sweden" OR "Switzerland" OR "Turkey" OR "Ukraine" OR "United Kingdom" OR "Uzbekistan" OR "Vatican City" OR "Wales" OR "Albanian" OR "Armenian" OR "Austrian" OR "Belgian" OR "Bosnian" OR "Bulgarian" OR "Croatian" OR "Czech" OR "Danish" OR "British" OR "Estonian" OR "Finnish" OR "French" OR "Georgian" OR "German" OR "Greek" OR "Hungarian" OR "Icelandic" OR "Irish" OR "Italian" OR "Kosovan" OR "Latvian" OR "Lithuanian" OR "Moldovan" OR "Dutch" OR "Macedonian" OR "Norwegian" OR "Polish" OR "Romanian" OR "Russian" OR "Scottish" OR "Serbian" OR "Slovakian" OR "Slovenian" OR "Spanish" OR "Swedish" OR "Swiss" OR "Turkish" OR "Ukrainian" OR "Welsh")):ti,ab,kw

AND (2013 OR 2014 OR 2015 OR 2016 OR 2017 OR 2018 OR 2019 OR 2020 OR 2021 OR 2022).yr

Cochrane library – Cardiovascular registries

(("cardiac implantable electronic device" OR "artificial heart pacemaker" OR "pacemaker" OR "pacemakers" OR "Artificial Heart" OR "artificial heart" OR "artificial hearts" OR "Heart Assist Device" OR "Artificial Heart" OR "Artificial Ventricle" OR "Artificial Ventricles" OR "Heart Assist Device" OR "Heart Assist Devices" OR "Heart Assist Pump" OR "Heart Assist Pumps" OR "Vascular Assist Device" OR "Vascular Assist Devices" OR "Ventricle Assist Device" OR "Ventricle Assist Devices" OR "Ventricular Assist Device" OR "Ventricular Assist Devices" OR "Heart Valve Prosthesis" OR "Heart Valve Prosthesis" OR "Heart Valve Prosthesis" OR "Cardiac Valve Prosthesis" OR "Cardiac Valve Prostheses" OR "Heart Prosthesis" OR "Heart Prosthesis" OR "Cardiac Prosthesis" OR "Cardiac Prostheses" OR "artificial heart valves" OR "artificial heart valve" OR "artificial valves" OR "artificial valves" OR "Implantable Defibrillator" OR "Implantable Defibrillator" OR "Implantable Defibrillators" OR "Implantable Cardioverter Defibrillator" OR "Implantable Cardioverter Defibrillators" OR "bioresorbable vascular stent" OR "bioresorbable vascular scaffold" OR "bioresorbable vascular scaffolds" OR "transcatheter aortic valve implantation" OR "transcatheter aortic valve implantation" OR

"transcatheter aortic valve implant" OR "transcatheter aortic valve implants" OR "TAVI" OR "transseptal mitral valve-in-ring" OR "TMVR" OR "LAAOC" OR (("Heart" OR "heart" OR "cardiac") AND ("Prosthesis" OR "Prostheses" OR "Prosthesis" OR "Implants" OR "Implant" OR "replacement" OR "replacements"))) AND ("Register" OR "register" OR "registers" OR "registry" OR "registries") AND ("European Union" OR "European Union" OR "European Community" OR "European Coal and Steel Community" OR "Common Market" OR "EEC" OR "European Economic Community" OR "European Common Market" OR "European Economic Area" OR "Europe" OR "Albania" OR "Andorra" OR "Armenia" OR "Armenia" OR "Austria" OR "Azerbaijan" OR "Republic of Belarus" OR "Belgium" OR "Bosnia and Herzegovina" OR "Bulgaria" OR "Croatia" OR "Czech Republic" OR "Denmark" OR "England" OR "Estonia" OR "Finland" OR "France" OR "Georgia" OR "Germany" OR "Gibraltar" OR "Greece" OR "Hungary" OR "Iceland" OR "Ireland" OR "Italy" OR "Kazakhstan" OR "Kosovo" OR "Kyrgyzstan" OR "Latvia" OR "Liechtenstein" OR "Lithuania" OR "Luxembourg" OR "Malta" OR "Moldova" OR "Monaco" OR "Montenegro" OR "Netherlands" OR "Republic of North Macedonia" OR "Northern Ireland" OR "Norway" OR "Poland" OR "Portugal" OR "Romania" OR "Russia" OR "San Marino" OR "Scotland" OR "Serbia" OR "Slovakia" OR "Slovenia" OR "Spain" OR "Sweden" OR "Switzerland" OR "Turkey" OR "Ukraine" OR "United Kingdom" OR "Uzbekistan" OR "Vatican City" OR "Wales" OR "Albanian" OR "Armenian" OR "Austrian" OR "Belgian" OR "Bosnian" OR "Bulgarian" OR "Croatian" OR "Czech" OR "Danish" OR "British" OR "Estonian" OR "Finnish" OR "French" OR "Georgian" OR "German" OR "Greek" OR "Hungarian" OR "Icelandic" OR "Irish" OR "Italian" OR "Kosovan" OR "Latvian" OR "Lithuanian" OR "Moldovan" OR "Dutch" OR "Macedonian" OR "Norwegian" OR "Polish" OR "Romanian" OR "Russian" OR "Scottish" OR "Serbian" OR "Slovakian" OR "Slovenian" OR "Spanish" OR "Swedish" OR "Swiss" OR "Turkish" OR "Ukrainian" OR "Welsh")):ti,ab,kw

AND (2013 OR 2014 OR 2015 OR 2016 OR 2017 OR 2018 OR 2019 OR 2020 OR 2021 OR 2022).yr

Embase – Orthopaedic registries

(((exp *"Hip Replacement"/ OR exp *"Hip Prosthesis"/ OR "hip replacement".ti OR "hip replacement*".ti OR "hip arthroplasty".ti OR "hip arthroplast*".ti OR "hip prosthesis".ti OR "hip prosthe*".ti OR "THA".ti OR "THR".ti OR "hip implant".ti OR "hip implants".ti OR exp *"Knee Replacement"/ OR exp *"Knee Prosthesis"/ OR "knee replacement".ti OR "knee replacement*".ti OR "knee arthroplasty".ti OR "knee arthroplast*".ti OR "knee prosthesis".ti OR "knee prosthe*".ti OR "TKA".ti OR "TKR".ti OR "knee implant".ti OR "knee implants".ti OR exp *"Shoulder Replacement"/

OR exp *"Shoulder Prosthesis"/ OR "shoulder replacement".ti OR "shoulder replacement*".ti OR "shoulder arthroplasty".ti OR "shoulder arthroplast*".ti OR "shoulder prosthesis".ti OR "shoulder prosthe*".ti OR "shoulder implant".ti OR "knee implants".ti OR exp *"Ankle Replacement"/ OR "Ankle Prosthesis"/ OR "ankle replacement".ti OR "ankle replacement*".ti OR "ankle arthroplasty".ti OR "ankle arthroplast*".ti OR "ankle prosthesis".ti OR "ankle prosthe*".ti OR "ankle implant".ti OR "ankle implants".ti OR ((exp *"Hip"/ OR "hip".ti OR "hips".ti OR exp *"Knee"/ OR "knee".ti OR "knees".ti OR exp *"Shoulder"/ OR "Shoulder".ti OR "Shoulders".ti OR exp *"Ankle"/ OR "ankle".ti OR "ankles".ti) AND (exp *"Prosthesis"/ OR "Prostheses".ti OR "Prosthesis".ti OR "Implants".ti OR "Implant".ti OR "replacement".ti OR "replacements".ti OR "arthroplasty".ti OR "arthroplast*".ti))) AND (exp "Register"/ OR "register".ti,ab OR "registers".ti,ab OR "registry".ti,ab OR "registries".ti,ab OR "register".in OR "registers".in OR "registry".in OR "registries".in) AND (exp "European Union"/ OR "European Union".ti,ab OR "European Community".ti,ab OR "European Coal and Steel Community".ti,ab OR "Common Market".ti,ab OR "EEC".ti,ab OR "European Economic Community".ti,ab OR "European Common Market".ti,ab OR "European Economic Area".ti,ab OR exp "Europe"/ OR "Albania"/ OR "Andorra"/ OR "Armenia"/ OR "Armenia"/ OR "Austria"/ OR "Azerbaijan"/ OR "Republic of Belarus"/ OR "Belgium"/ OR "Bosnia and Herzegovina"/ OR "Bulgaria"/ OR "Croatia"/ OR "Czech Republic"/ OR "Denmark"/ OR "England"/ OR "Estonia"/ OR "Finland"/ OR "France"/ OR "Georgia"/ OR "Germany"/ OR "Gibraltar"/ OR "Greece"/ OR "Hungary"/ OR "Iceland"/ OR "Ireland"/ OR "Italy"/ OR "Kazakhstan"/ OR "Kosovo"/ OR "Kyrgyzstan"/ OR "Latvia"/ OR "Liechtenstein"/ OR "Lithuania"/ OR "Luxembourg"/ OR "Malta"/ OR "Moldova"/ OR "Monaco"/ OR "Montenegro"/ OR "Netherlands"/ OR "Republic of North Macedonia"/ OR "Northern Ireland"/ OR "Norway"/ OR "Poland"/ OR "Portugal"/ OR "Romania"/ OR "Russia"/ OR "San Marino"/ OR "Scotland"/ OR "Serbia"/ OR "Slovakia"/ OR "Slovenia"/ OR "Spain"/ OR "Sweden"/ OR "Switzerland"/ OR "Turkey"/ OR "Ukraine"/ OR "United Kingdom"/ OR "Uzbekistan"/ OR "Vatican City"/ OR "Wales"/ OR "Europe".ti,ab OR "European".ti,ab OR "Albania".ti,ab OR "Andorra".ti,ab OR "Armenia".ti,ab OR "Armenia".ti,ab OR "Austria".ti,ab OR "Azerbaijan".ti,ab OR "Belarus".ti,ab OR "Belgium".ti,ab OR "Bosnia".ti,ab OR "Bulgaria".ti,ab OR "Croatia".ti,ab OR "Czech Republic".ti,ab OR "Denmark".ti,ab OR "England".ti,ab OR "Estonia".ti,ab OR "Finland".ti,ab OR "France".ti,ab OR "Georgia".ti,ab OR "Germany".ti,ab OR "Gibraltar".ti,ab OR "Greece".ti,ab OR "Herzegovina".ti,ab OR "Hungary".ti,ab OR "Iceland".ti,ab OR "Ireland".ti,ab OR "Italy".ti,ab OR "Kazakhstan".ti,ab OR "Kosovo".ti,ab OR "Kyrgyzstan".ti,ab OR "Latvia".ti,ab OR "Liechtenstein".ti,ab OR "Lithuania".ti,ab OR "Luxembourg".ti,ab OR "Malta".ti,ab OR "Moldova".ti,ab OR "Monaco".ti,ab OR "Montenegro".ti,ab OR "Netherlands".ti,ab OR "North Macedonia".ti,ab OR "Northern Ireland".ti,ab OR "Norway".ti,ab OR "Poland".ti,ab OR "Portugal".ti,ab OR "Romania".ti,ab OR "Russia".ti,ab OR "San Marino".ti,ab OR "Scotland".ti,ab OR "Serbia".ti,ab OR "Slovakia".ti,ab OR

"Slovenia".ti,ab OR "Spain".ti,ab OR "Sweden".ti,ab OR "Switzerland".ti,ab OR "Turkey".ti,ab OR "Ukraine".ti,ab OR "United Kingdom".ti,ab OR "Uzbekistan".ti,ab OR "Vatican City".ti,ab OR "Wales".ti,ab OR "Albanian".ti,ab OR "Armenian".ti,ab OR "Austrian".ti,ab OR "Belgian".ti,ab OR "Bosnian".ti,ab OR "Bulgarian".ti,ab OR "Croatian".ti,ab OR "Czech".ti,ab OR "Danish".ti,ab OR "British".ti,ab OR "Estonian".ti,ab OR "Finnish".ti,ab OR "French".ti,ab OR "Georgian".ti,ab OR "German".ti,ab OR "Greek".ti,ab OR "Hungarian".ti,ab OR "Icelandic".ti,ab OR "Irish".ti,ab OR "Italian".ti,ab OR "Kosovan".ti,ab OR "Latvian".ti,ab OR "Lithuanian".ti,ab OR "Moldovan".ti,ab OR "Dutch".ti,ab OR "Macedonian".ti,ab OR "Norwegian".ti,ab OR "Polish".ti,ab OR "Romanian".ti,ab OR "Russian".ti,ab OR "Scottish".ti,ab OR "Serbian".ti,ab OR "Slovakian".ti,ab OR "Slovenian".ti,ab OR "Spanish".ti,ab OR "Swedish".ti,ab OR "Swiss".ti,ab OR "Turkish".ti,ab OR "Ukrainian".ti,ab OR "Welsh".ti,ab)) **OR** ((exp *"Hip Replacement"/ OR exp *"Hip Prosthesis"/ OR "hip replacement".ti,ab OR "hip replacement*".ti,ab OR "hip arthroplasty".ti,ab OR "hip arthroplast*".ti,ab OR "hip prosthesis".ti,ab OR "hip prosthe*".ti,ab OR "THA".ti,ab OR "THR".ti,ab OR "hip implant".ti,ab OR "hip implants".ti,ab OR exp *"Knee Replacement"/ OR exp *"Knee Prosthesis"/ OR "knee replacement".ti,ab OR "knee replacement*".ti,ab OR "knee arthroplasty".ti,ab OR "knee arthroplast*".ti,ab OR "knee prosthesis".ti,ab OR "knee prosthe*".ti,ab OR "TKA".ti,ab OR "TKR".ti,ab OR "knee implant".ti,ab OR "knee implants".ti,ab OR exp *"Shoulder Replacement"/ OR exp *"Shoulder Prosthesis"/ OR "shoulder replacement".ti,ab OR "shoulder replacement*".ti,ab OR "shoulder arthroplasty".ti,ab OR "shoulder arthroplast*".ti,ab OR "shoulder prosthesis".ti,ab OR "shoulder prosthe*".ti,ab OR "shoulder implant".ti,ab OR "knee implants".ti,ab OR exp *"Ankle Replacement"/ OR "Ankle Prosthesis"/ OR "ankle replacement".ti,ab OR "ankle replacement*".ti,ab OR "ankle arthroplasty".ti,ab OR "ankle arthroplast*".ti,ab OR "ankle prosthesis".ti,ab OR "ankle prosthe*".ti,ab OR "ankle implant".ti,ab OR "ankle implants".ti,ab OR ((exp *"Hip"/ OR "hip".ti,ab OR "hips".ti,ab OR exp *"Knee"/ OR "knee".ti,ab OR "knees".ti,ab OR exp *"Shoulder"/ OR "Shoulder".ti,ab OR "Shoulders".ti,ab OR exp *"Ankle"/ OR "ankle".ti,ab OR "ankles".ti,ab) AND (exp *"Prosthesis"/ OR "Prostheses".ti,ab OR "Prosthesis".ti,ab OR "Implants".ti,ab OR "Implant".ti,ab OR "replacement".ti,ab OR "replacements".ti,ab OR "arthroplasty".ti,ab OR "arthroplast*".ti,ab))) AND (exp *"Register"/ OR "register".ti OR "registers".ti OR "registry".ti OR "registries".ti OR "register".in OR "registers".in OR "registry".in OR "registries".in) AND (exp "European Union"/ OR "European Union".ti,ab OR "European Community".ti,ab OR "European Coal and Steel Community".ti,ab OR "Common Market".ti,ab OR "EEC".ti,ab OR "European Economic Community".ti,ab OR "European Common Market".ti,ab OR "European Economic Area".ti,ab OR exp "Europe"/ OR "Albania"/ OR "Andorra"/ OR "Armenia"/ OR "Armenia"/ OR "Austria"/ OR "Azerbaijan"/ OR "Republic of Belarus"/ OR "Belgium"/ OR "Bosnia and Herzegovina"/ OR "Bulgaria"/ OR "Croatia"/ OR "Czech Republic"/ OR "Denmark"/ OR "England"/ OR "Estonia"/ OR "Finland"/ OR

"France"/ OR "Georgia"/ OR "Germany"/ OR "Gibraltar"/ OR "Greece"/ OR "Hungary"/ OR "Iceland"/ OR "Ireland"/ OR "Italy"/ OR "Kazakhstan"/ OR "Kosovo"/ OR "Kyrgyzstan"/ OR "Latvia"/ OR "Liechtenstein"/ OR "Lithuania"/ OR "Luxembourg"/ OR "Malta"/ OR "Moldova"/ OR "Monaco"/ OR "Montenegro"/ OR "Netherlands"/ OR "Republic of North Macedonia"/ OR "Northern Ireland"/ OR "Norway"/ OR "Poland"/ OR "Portugal"/ OR "Romania"/ OR "Russia"/ OR "San Marino"/ OR "Scotland"/ OR "Serbia"/ OR "Slovakia"/ OR "Slovenia"/ OR "Spain"/ OR "Sweden"/ OR "Switzerland"/ OR "Turkey"/ OR "Ukraine"/ OR "United Kingdom"/ OR "Uzbekistan"/ OR "Vatican City"/ OR "Wales"/ OR "Europe".ti,ab OR "European".ti,ab OR "Albania".ti,ab OR "Andorra".ti,ab OR "Armenia".ti,ab OR "Armenia".ti,ab OR "Austria".ti,ab OR "Azerbaijan".ti,ab OR "Belarus".ti,ab OR "Belgium".ti,ab OR "Bosnia".ti,ab OR "Bulgaria".ti,ab OR "Croatia".ti,ab OR "Czech Republic".ti,ab OR "Denmark".ti,ab OR "England".ti,ab OR "Estonia".ti,ab OR "Finland".ti,ab OR "France".ti,ab OR "Georgia".ti,ab OR "Germany".ti,ab OR "Gibraltar".ti,ab OR "Greece".ti,ab OR "Herzegovina".ti,ab OR "Hungary".ti,ab OR "Iceland".ti,ab OR "Ireland".ti,ab OR "Italy".ti,ab OR "Kazakhstan".ti,ab OR "Kosovo".ti,ab OR "Kyrgyzstan".ti,ab OR "Latvia".ti,ab OR "Liechtenstein".ti,ab OR "Lithuania".ti,ab OR "Luxembourg".ti,ab OR "Malta".ti,ab OR "Moldova".ti,ab OR "Monaco".ti,ab OR "Montenegro".ti,ab OR "Netherlands".ti,ab OR "North Macedonia".ti,ab OR "Northern Ireland".ti,ab OR "Norway".ti,ab OR "Poland".ti,ab OR "Portugal".ti,ab OR "Romania".ti,ab OR "Russia".ti,ab OR "San Marino".ti,ab OR "Scotland".ti,ab OR "Serbia".ti,ab OR "Slovakia".ti,ab OR "Slovenia".ti,ab OR "Spain".ti,ab OR "Sweden".ti,ab OR "Switzerland".ti,ab OR "Turkey".ti,ab OR "Ukraine".ti,ab OR "United Kingdom".ti,ab OR "Uzbekistan".ti,ab OR "Vatican City".ti,ab OR "Wales".ti,ab OR "Albanian".ti,ab OR "Armenian".ti,ab OR "Austrian".ti,ab OR "Belgian".ti,ab OR "Bosnian".ti,ab OR "Bulgarian".ti,ab OR "Croatian".ti,ab OR "Czech".ti,ab OR "Danish".ti,ab OR "British".ti,ab OR "Estonian".ti,ab OR "Finnish".ti,ab OR "French".ti,ab OR "Georgian".ti,ab OR "German".ti,ab OR "Greek".ti,ab OR "Hungarian".ti,ab OR "Icelandic".ti,ab OR "Irish".ti,ab OR "Italian".ti,ab OR "Kosovan".ti,ab OR "Latvian".ti,ab OR "Lithuanian".ti,ab OR "Moldovan".ti,ab OR "Dutch".ti,ab OR "Macedonian".ti,ab OR "Norwegian".ti,ab OR "Polish".ti,ab OR "Romanian".ti,ab OR "Russian".ti,ab OR "Scottish".ti,ab OR "Serbian".ti,ab OR "Slovakian".ti,ab OR "Slovenian".ti,ab OR "Spanish".ti,ab OR "Swedish".ti,ab OR "Swiss".ti,ab OR "Turkish".ti,ab OR "Ukrainian".ti,ab OR "Welsh".ti,ab))**)** AND (2013 OR 2014 OR 2015 OR 2016 OR 2017 OR 2018 OR 2019 OR 2020 OR 2021 OR 2022).yr NOT (conference review or conference abstract).pt

Embase – Cardiovascular registries

(((exp *"cardiac implantable electronic device"/ OR exp *"artificial heart pacemaker"/ OR "pacemaker".ti OR "pacemakers".ti OR exp *"Artificial Heart"/ OR "artificial heart".ti OR "artificial hearts".ti OR exp *"Heart Assist Device"/ OR "Artificial Heart".ti OR "Artificial Ventricle".ti OR "Artificial Ventricles".ti OR "Heart Assist Device".ti OR "Heart Assist Devices".ti OR "Heart Assist Pump".ti OR "Heart Assist Pumps".ti OR "Vascular Assist Device".ti OR "Vascular Assist Devices".ti OR "Ventricle Assist Device".ti OR "Ventricle Assist Devices".ti OR "Ventricular Assist Device".ti OR "Ventricular Assist Devices".ti OR exp *"Heart Valve Prosthesis"/ OR "Heart Valve Prosthesis".ti OR "Heart Valve Prosthesis".ti OR "Cardiac Valve Prosthesis".ti OR "Cardiac Valve Prostheses".ti OR "Heart Prosthesis".ti OR "Heart Prosthesis".ti OR "Cardiac Prosthesis".ti OR "Cardiac Prostheses".ti OR "artificial heart valves".ti OR "artificial heart valve".ti OR "artificial valves".ti OR "artificial valves".ti OR exp *"Implantable Defibrillator"/ OR "Implantable Defibrillator".ti OR "Implantable Defibrillators".ti OR "Implantable Cardioverter Defibrillator".ti OR "Implantable Cardioverter Defibrillators".ti OR exp *"bioresorbable vascular stent"/ OR "bioresorbable vascular scaffold".ti OR "bioresorbable vascular scaffolds".ti OR exp *"transcatheter aortic valve implantation"/ OR "transcatheter aortic valve implantation".ti OR "transcatheter aortic valve implant".ti OR "transcatheter aortic valve implants".ti OR "TAVI".ti OR "transseptal mitral valve-in-ring".ti OR "TMVR".ti OR "LAAOC".ti OR ((exp *"Heart"/ OR "heart".ti OR "cardiac".ti) AND (exp *"Prosthesis"/ OR "Prostheses".ti OR "Prosthesis".ti OR "Implants".ti OR "Implant".ti OR "replacement".ti OR "replacements".ti))) AND (exp "Register"/ OR "register".ti,ab OR "registers".ti,ab OR "registry".ti,ab OR "registries".ti,ab OR "register".in OR "registers".in OR "registry".in OR "registries".in) AND (exp "European Union"/ OR "European Union".ti,ab OR "European Community".ti,ab OR "European Coal and Steel Community".ti,ab OR "Common Market".ti,ab OR "EEC".ti,ab OR "European Economic Community".ti,ab OR "European Common Market".ti,ab OR "European Economic Area".ti,ab OR exp "Europe"/ OR "Albania"/ OR "Andorra"/ OR "Armenia"/ OR "Armenia"/ OR "Austria"/ OR "Azerbaijan"/ OR "Republic of Belarus"/ OR "Belgium"/ OR "Bosnia and Herzegovina"/ OR "Bulgaria"/ OR "Croatia"/ OR "Czech Republic"/ OR "Denmark"/ OR "England"/ OR "Estonia"/ OR "Finland"/ OR "France"/ OR "Georgia"/ OR "Germany"/ OR "Gibraltar"/ OR "Greece"/ OR "Hungary"/ OR "Iceland"/ OR "Ireland"/ OR "Italy"/ OR "Kazakhstan"/ OR "Kosovo"/ OR "Kyrgyzstan"/ OR "Latvia"/ OR "Liechtenstein"/ OR "Lithuania"/ OR "Luxembourg"/ OR "Malta"/ OR "Moldova"/ OR "Monaco"/ OR "Montenegro"/ OR "Netherlands"/ OR "Republic of North Macedonia"/ OR "Northern Ireland"/ OR "Norway"/ OR "Poland"/ OR "Portugal"/ OR "Romania"/ OR "Russia"/ OR "San Marino"/ OR "Scotland"/ OR "Serbia"/ OR "Slovakia"/ OR "Slovenia"/ OR "Spain"/ OR "Sweden"/ OR "Switzerland"/ OR "Turkey"/ OR "Ukraine"/ OR "United Kingdom"/ OR "Uzbekistan"/ OR "Vatican City"/ OR "Wales"/ OR "Europe".ti,ab OR "European".ti,ab OR "Albania".ti,ab OR "Andorra".ti,ab OR "Armenia".ti,ab OR "Armenia".ti,ab OR "Austria".ti,ab OR

"Azerbaijan".ti,ab OR "Belarus".ti,ab OR "Belgium".ti,ab OR "Bosnia".ti,ab OR "Bulgaria".ti,ab OR "Croatia".ti,ab OR "Czech Republic".ti,ab OR "Denmark".ti,ab OR "England".ti,ab OR "Estonia".ti,ab OR "Finland".ti,ab OR "France".ti,ab OR "Georgia".ti,ab OR "Germany".ti,ab OR "Gibraltar".ti,ab OR "Greece".ti,ab OR "Herzegovina".ti,ab OR "Hungary".ti,ab OR "Iceland".ti,ab OR "Ireland".ti,ab OR "Italy".ti,ab OR "Kazakhstan".ti,ab OR "Kosovo".ti,ab OR "Kyrgyzstan".ti,ab OR "Latvia".ti,ab OR "Liechtenstein".ti,ab OR "Lithuania".ti,ab OR "Luxembourg".ti,ab OR "Malta".ti,ab OR "Moldova".ti,ab OR "Monaco".ti,ab OR "Montenegro".ti,ab OR "Netherlands".ti,ab OR "North Macedonia".ti,ab OR "Northern Ireland".ti,ab OR "Norway".ti,ab OR "Poland".ti,ab OR "Portugal".ti,ab OR "Romania".ti,ab OR "Russia".ti,ab OR "San Marino".ti,ab OR "Scotland".ti,ab OR "Serbia".ti,ab OR "Slovakia".ti,ab OR "Slovenia".ti,ab OR "Spain".ti,ab OR "Sweden".ti,ab OR "Switzerland".ti,ab OR "Turkey".ti,ab OR "Ukraine".ti,ab OR "United Kingdom".ti,ab OR "Uzbekistan".ti,ab OR "Vatican City".ti,ab OR "Wales".ti,ab OR "Albanian".ti,ab OR "Armenian".ti,ab OR "Austrian".ti,ab OR "Belgian".ti,ab OR "Bosnian".ti,ab OR "Bulgarian".ti,ab OR "Croatian".ti,ab OR "Czech".ti,ab OR "Danish".ti,ab OR "British".ti,ab OR "Estonian".ti,ab OR "Finnish".ti,ab OR "French".ti,ab OR "Georgian".ti,ab OR "German".ti,ab OR "Greek".ti,ab OR "Hungarian".ti,ab OR "Icelandic".ti,ab OR "Irish".ti,ab OR "Italian".ti,ab OR "Kosovan".ti,ab OR "Latvian".ti,ab OR "Lithuanian".ti,ab OR "Moldovan".ti,ab OR "Dutch".ti,ab OR "Macedonian".ti,ab OR "Norwegian".ti,ab OR "Polish".ti,ab OR "Romanian".ti,ab OR "Russian".ti,ab OR "Scottish".ti,ab OR "Serbian".ti,ab OR "Slovakian".ti,ab OR "Slovenian".ti,ab OR "Spanish".ti,ab OR "Swedish".ti,ab OR "Swiss".ti,ab OR "Turkish".ti,ab OR "Ukrainian".ti,ab OR "Welsh".ti,ab)) **OR** ((exp *"cardiac implantable electronic device"/ OR exp *"artificial heart pacemaker"/ OR "pacemaker".ti,ab OR "pacemakers".ti,ab OR exp *"Artificial Heart"/ OR "artificial heart".ti,ab OR "artificial hearts".ti,ab OR exp *"Heart Assist Device"/ OR "Artificial Heart".ti,ab OR "Artificial Ventricle".ti,ab OR "Artificial Ventricles".ti,ab OR "Heart Assist Device".ti,ab OR "Heart Assist Devices".ti,ab OR "Heart Assist Pump".ti,ab OR "Heart Assist Pumps".ti,ab OR "Vascular Assist Device".ti,ab OR "Vascular Assist Devices".ti,ab OR "Ventricle Assist Device".ti,ab OR "Ventricle Assist Devices".ti,ab OR "Ventricular Assist Device".ti,ab OR "Ventricular Assist Devices".ti,ab OR exp *"Heart Valve Prosthesis"/ OR "Heart Valve Prosthesis".ti,ab OR "Heart Valve Prosthesis".ti,ab OR "Cardiac Valve Prosthesis".ti,ab OR "Cardiac Valve Prostheses".ti,ab OR "Heart Prosthesis".ti,ab OR "Heart Prosthesis".ti,ab OR "Cardiac Prosthesis".ti,ab OR "Cardiac Prostheses".ti,ab OR "artificial heart valves".ti,ab OR "artificial heart valve".ti,ab OR "artificial valves".ti,ab OR "artificial valves".ti,ab OR exp *"Implantable Defibrillator"/ OR "Implantable Defibrillator".ti,ab OR "Implantable Defibrillators".ti,ab OR "Implantable Cardioverter Defibrillator".ti,ab OR "Implantable Cardioverter Defibrillators".ti,ab OR exp *"bioresorbable vascular stent"/ OR "bioresorbable vascular scaffold".ti,ab OR "bioresorbable vascular scaffolds".ti,ab OR exp *"transcatheter aortic valve implantation"/ OR

"transcatheter aortic valve implantation".ti,ab OR "transcatheter aortic valve implant".ti,ab OR "transcatheter aortic valve implants".ti,ab OR "TAVI".ti,ab OR "transseptal mitral valve-in-ring".ti,ab OR "TMVR".ti,ab OR "LAAOC".ti,ab OR ((exp *"Heart"/ OR "heart".ti,ab OR "cardiac".ti,ab) AND (exp *"Prosthesis"/ OR "Prostheses".ti,ab OR "Prosthesis".ti,ab OR "Implants".ti,ab OR "Implant".ti,ab OR "replacement".ti,ab OR "replacements".ti,ab))) AND (exp *"Register"/ OR "register".ti OR "registers".ti OR "registry".ti OR "registries".ti OR "register".in OR "registers".in OR "registry".in OR "registries".in) AND (exp "European Union"/ OR "European Union".ti,ab OR "European Community".ti,ab OR "European Coal and Steel Community".ti,ab OR "Common Market".ti,ab OR "EEC".ti,ab OR "European Economic Community".ti,ab OR "European Common Market".ti,ab OR "European Economic Area".ti,ab OR exp "Europe"/ OR "Albania"/ OR "Andorra"/ OR "Armenia"/ OR "Armenia"/ OR "Austria"/ OR "Azerbaijan"/ OR "Republic of Belarus"/ OR "Belgium"/ OR "Bosnia and Herzegovina"/ OR "Bulgaria"/ OR "Croatia"/ OR "Czech Republic"/ OR "Denmark"/ OR "England"/ OR "Estonia"/ OR "Finland"/ OR "France"/ OR "Georgia"/ OR "Germany"/ OR "Gibraltar"/ OR "Greece"/ OR "Hungary"/ OR "Iceland"/ OR "Ireland"/ OR "Italy"/ OR "Kazakhstan"/ OR "Kosovo"/ OR "Kyrgyzstan"/ OR "Latvia"/ OR "Liechtenstein"/ OR "Lithuania"/ OR "Luxembourg"/ OR "Malta"/ OR "Moldova"/ OR "Monaco"/ OR "Montenegro"/ OR "Netherlands"/ OR "Republic of North Macedonia"/ OR "Northern Ireland"/ OR "Norway"/ OR "Poland"/ OR "Portugal"/ OR "Romania"/ OR "Russia"/ OR "San Marino"/ OR "Scotland"/ OR "Serbia"/ OR "Slovakia"/ OR "Slovenia"/ OR "Spain"/ OR "Sweden"/ OR "Switzerland"/ OR "Turkey"/ OR "Ukraine"/ OR "United Kingdom"/ OR "Uzbekistan"/ OR "Vatican City"/ OR "Wales"/ OR "Europe".ti,ab OR "European".ti,ab OR "Albania".ti,ab OR "Andorra".ti,ab OR "Armenia".ti,ab OR "Armenia".ti,ab OR "Austria".ti,ab OR "Azerbaijan".ti,ab OR "Belarus".ti,ab OR "Belgium".ti,ab OR "Bosnia".ti,ab OR "Bulgaria".ti,ab OR "Croatia".ti,ab OR "Czech Republic".ti,ab OR "Denmark".ti,ab OR "England".ti,ab OR "Estonia".ti,ab OR "Finland".ti,ab OR "France".ti,ab OR "Georgia".ti,ab OR "Germany".ti,ab OR "Gibraltar".ti,ab OR "Greece".ti,ab OR "Herzegovina".ti,ab OR "Hungary".ti,ab OR "Iceland".ti,ab OR "Ireland".ti,ab OR "Italy".ti,ab OR "Kazakhstan".ti,ab OR "Kosovo".ti,ab OR "Kyrgyzstan".ti,ab OR "Latvia".ti,ab OR "Liechtenstein".ti,ab OR "Lithuania".ti,ab OR "Luxembourg".ti,ab OR "Malta".ti,ab OR "Moldova".ti,ab OR "Monaco".ti,ab OR "Montenegro".ti,ab OR "Netherlands".ti,ab OR "North Macedonia".ti,ab OR "Northern Ireland".ti,ab OR "Norway".ti,ab OR "Poland".ti,ab OR "Portugal".ti,ab OR "Romania".ti,ab OR "Russia".ti,ab OR "San Marino".ti,ab OR "Scotland".ti,ab OR "Serbia".ti,ab OR "Slovakia".ti,ab OR "Slovenia".ti,ab OR "Spain".ti,ab OR "Sweden".ti,ab OR "Switzerland".ti,ab OR "Turkey".ti,ab OR "Ukraine".ti,ab OR "United Kingdom".ti,ab OR "Uzbekistan".ti,ab OR "Vatican City".ti,ab OR "Wales".ti,ab OR "Albanian".ti,ab OR "Armenian".ti,ab OR "Austrian".ti,ab OR "Belgian".ti,ab OR "Bosnian".ti,ab OR "Bulgarian".ti,ab OR "Croatian".ti,ab OR "Czech".ti,ab OR "Danish".ti,ab OR "British".ti,ab OR

"Estonian".ti,ab OR "Finnish".ti,ab OR "French".ti,ab OR "Georgian".ti,ab OR "German".ti,ab OR "Greek".ti,ab OR "Hungarian".ti,ab OR "Icelandic".ti,ab OR "Irish".ti,ab OR "Italian".ti,ab OR "Kosovan".ti,ab OR "Latvian".ti,ab OR "Lithuanian".ti,ab OR "Moldovan".ti,ab OR "Dutch".ti,ab OR "Macedonian".ti,ab OR "Norwegian".ti,ab OR "Polish".ti,ab OR "Romanian".ti,ab OR "Russian".ti,ab OR "Scottish".ti,ab OR "Serbian".ti,ab OR "Slovakian".ti,ab OR "Slovenian".ti,ab OR "Spanish".ti,ab OR "Swedish".ti,ab OR "Swiss".ti,ab OR "Turkish".ti,ab OR "Ukrainian".ti,ab OR "Welsh".ti,ab))) AND (2013 OR 2014 OR 2015 OR 2016 OR 2017 OR 2018 OR 2019 OR 2020 OR 2021 OR 2022).yr NOT (conference review or conference abstract).pt

Emcare – Orthopaedic registries

(((exp *"Hip Replacement"/ OR exp *"Hip Prosthesis"/ OR "hip replacement".ti OR "hip replacement*".ti OR "hip arthroplasty".ti OR "hip arthroplast*".ti OR "hip prosthesis".ti OR "hip prosthe*".ti OR "THA".ti OR "THR".ti OR "hip implant".ti OR "hip implants".ti OR exp *"Knee Replacement"/ OR exp *"Knee Prosthesis"/ OR "knee replacement".ti OR "knee replacement*".ti OR "knee arthroplasty".ti OR "knee arthroplast*".ti OR "knee prosthesis".ti OR "knee prosthe*".ti OR "TKA".ti OR "TKR".ti OR "knee implant".ti OR "knee implants".ti OR exp *"Shoulder Replacement"/ OR exp *"Shoulder Prosthesis"/ OR "shoulder replacement".ti OR "shoulder replacement*".ti OR "shoulder arthroplasty".ti OR "shoulder arthroplast*".ti OR "shoulder prosthesis".ti OR "shoulder prosthe*".ti OR "shoulder implant".ti OR "knee implants".ti OR exp *"Ankle Replacement"/ OR "Ankle Prosthesis"/ OR "ankle replacement".ti OR "ankle replacement*".ti OR "ankle arthroplasty".ti OR "ankle arthroplast*".ti OR "ankle prosthesis".ti OR "ankle prosthe*".ti OR "ankle implant".ti OR "ankle implants".ti OR ((exp *"Hip"/ OR "hip".ti OR "hips".ti OR exp *"Knee"/ OR "knee".ti OR "knees".ti OR exp *"Shoulder"/ OR "Shoulder".ti OR "Shoulders".ti OR exp *"Ankle"/ OR "ankle".ti OR "ankles".ti) AND (exp *"Prosthesis"/ OR "Prostheses".ti OR "Prosthesis".ti OR "Implants".ti OR "Implant".ti OR "replacement".ti OR "replacements".ti OR "arthroplasty".ti OR "arthroplast*".ti))) AND (exp "Register"/ OR "register".ti,ab OR "registers".ti,ab OR "registry".ti,ab OR "registries".ti,ab OR "register".in OR "registers".in OR "registry".in OR "registries".in) AND (exp "European Union"/ OR "European Union".ti,ab OR "European Community".ti,ab OR "European Coal and Steel Community".ti,ab OR "Common Market".ti,ab OR "EEC".ti,ab OR "European Economic Community".ti,ab OR "European Common Market".ti,ab OR "European Economic Area".ti,ab OR exp "Europe"/ OR "Albania"/ OR "Andorra"/ OR "Armenia"/ OR "Armenia"/ OR "Austria"/ OR "Azerbaijan"/ OR "Republic of Belarus"/ OR "Belgium"/ OR "Bosnia and Herzegovina"/ OR "Bulgaria"/ OR "Croatia"/ OR "Czech Republic"/ OR "Denmark"/ OR "England"/ OR "Estonia"/ OR "Finland"/ OR "France"/ OR "Georgia"/ OR "Germany"/

OR "Gibraltar"/ OR "Greece"/ OR "Hungary"/ OR "Iceland"/ OR "Ireland"/ OR "Italy"/ OR "Kazakhstan"/ OR "Kosovo"/ OR "Kyrgyzstan"/ OR "Latvia"/ OR "Liechtenstein"/ OR "Lithuania"/ OR "Luxembourg"/ OR "Malta"/ OR "Moldova"/ OR "Monaco"/ OR "Montenegro"/ OR "Netherlands"/ OR "Republic of North Macedonia"/ OR "Northern Ireland"/ OR "Norway"/ OR "Poland"/ OR "Portugal"/ OR "Romania"/ OR "Russia"/ OR "San Marino"/ OR "Scotland"/ OR "Serbia"/ OR "Slovakia"/ OR "Slovenia"/ OR "Spain"/ OR "Sweden"/ OR "Switzerland"/ OR "Turkey"/ OR "Ukraine"/ OR "United Kingdom"/ OR "Uzbekistan"/ OR "Vatican City"/ OR "Wales"/ OR "Europe".ti,ab OR "European".ti,ab OR "Albania".ti,ab OR "Andorra".ti,ab OR "Armenia".ti,ab OR "Armenia".ti,ab OR "Austria".ti,ab OR "Azerbaijan".ti,ab OR "Belarus".ti,ab OR "Belgium".ti,ab OR "Bosnia".ti,ab OR "Bulgaria".ti,ab OR "Croatia".ti,ab OR "Czech Republic".ti,ab OR "Denmark".ti,ab OR "England".ti,ab OR "Estonia".ti,ab OR "Finland".ti,ab OR "France".ti,ab OR "Georgia".ti,ab OR "Germany".ti,ab OR "Gibraltar".ti,ab OR "Greece".ti,ab OR "Herzegovina".ti,ab OR "Hungary".ti,ab OR "Iceland".ti,ab OR "Ireland".ti,ab OR "Italy".ti,ab OR "Kazakhstan".ti,ab OR "Kosovo".ti,ab OR "Kyrgyzstan".ti,ab OR "Latvia".ti,ab OR "Liechtenstein".ti,ab OR "Lithuania".ti,ab OR "Luxembourg".ti,ab OR "Malta".ti,ab OR "Moldova".ti,ab OR "Monaco".ti,ab OR "Montenegro".ti,ab OR "Netherlands".ti,ab OR "North Macedonia".ti,ab OR "Northern Ireland".ti,ab OR "Norway".ti,ab OR "Poland".ti,ab OR "Portugal".ti,ab OR "Romania".ti,ab OR "Russia".ti,ab OR "San Marino".ti,ab OR "Scotland".ti,ab OR "Serbia".ti,ab OR "Slovakia".ti,ab OR "Slovenia".ti,ab OR "Spain".ti,ab OR "Sweden".ti,ab OR "Switzerland".ti,ab OR "Turkey".ti,ab OR "Ukraine".ti,ab OR "United Kingdom".ti,ab OR "Uzbekistan".ti,ab OR "Vatican City".ti,ab OR "Wales".ti,ab OR "Albanian".ti,ab OR "Armenian".ti,ab OR "Austrian".ti,ab OR "Belgian".ti,ab OR "Bosnian".ti,ab OR "Bulgarian".ti,ab OR "Croatian".ti,ab OR "Czech".ti,ab OR "Danish".ti,ab OR "British".ti,ab OR "Estonian".ti,ab OR "Finnish".ti,ab OR "French".ti,ab OR "Georgian".ti,ab OR "German".ti,ab OR "Greek".ti,ab OR "Hungarian".ti,ab OR "Icelandic".ti,ab OR "Irish".ti,ab OR "Italian".ti,ab OR "Kosovan".ti,ab OR "Latvian".ti,ab OR "Lithuanian".ti,ab OR "Moldovan".ti,ab OR "Dutch".ti,ab OR "Macedonian".ti,ab OR "Norwegian".ti,ab OR "Polish".ti,ab OR "Romanian".ti,ab OR "Russian".ti,ab OR "Scottish".ti,ab OR "Serbian".ti,ab OR "Slovakian".ti,ab OR "Slovenian".ti,ab OR "Spanish".ti,ab OR "Swedish".ti,ab OR "Swiss".ti,ab OR "Turkish".ti,ab OR "Ukrainian".ti,ab OR "Welsh".ti,ab)) **OR** ((exp *"Hip Replacement"/ OR exp *"Hip Prosthesis"/ OR "hip replacement".ti,ab OR "hip replacement*".ti,ab OR "hip arthroplasty".ti,ab OR "hip arthroplast*".ti,ab OR "hip prosthesis".ti,ab OR "hip prosthe*".ti,ab OR "THA".ti,ab OR "THR".ti,ab OR "hip implant".ti,ab OR "hip implants".ti,ab OR exp *"Knee Replacement"/ OR exp *"Knee Prosthesis"/ OR "knee replacement".ti,ab OR "knee replacement*".ti,ab OR "knee arthroplasty".ti,ab OR "knee arthroplast*".ti,ab OR "knee prosthesis".ti,ab OR "knee prosthe*".ti,ab OR "TKA".ti,ab OR "TKR".ti,ab OR "knee implant".ti,ab OR "knee implants".ti,ab OR exp *"Shoulder Replacement"/ OR exp *"Shoulder Prosthesis"/ OR "shoulder

replacement".ti,ab OR "shoulder replacement*".ti,ab OR "shoulder arthroplasty".ti,ab OR "shoulder arthroplast*".ti,ab OR "shoulder prosthesis".ti,ab OR "shoulder prosthe*".ti,ab OR "shoulder implant".ti,ab OR "knee implants".ti,ab OR exp *"Ankle Replacement"/ OR "Ankle Prosthesis"/ OR "ankle replacement".ti,ab OR "ankle replacement*".ti,ab OR "ankle arthroplasty".ti,ab OR "ankle arthroplast*".ti,ab OR "ankle prosthesis".ti,ab OR "ankle prosthe*".ti,ab OR "ankle implant".ti,ab OR "ankle implants".ti,ab OR ((exp *"Hip"/ OR "hip".ti,ab OR "hips".ti,ab OR exp *"Knee"/ OR "knee".ti,ab OR "knees".ti,ab OR exp *"Shoulder"/ OR "Shoulder".ti,ab OR "Shoulders".ti,ab OR exp *"Ankle"/ OR "ankle".ti,ab OR "ankles".ti,ab) AND (exp *"Prosthesis"/ OR "Prostheses".ti,ab OR "Prosthesis".ti,ab OR "Implants".ti,ab OR "Implant".ti,ab OR "replacement".ti,ab OR "replacements".ti,ab OR "arthroplasty".ti,ab OR "arthroplast*".ti,ab))) AND (exp *"Register"/ OR "register".ti OR "registers".ti OR "registry".ti OR "registries".ti OR "register".in OR "registers".in OR "registry".in OR "registries".in) AND (exp "European Union"/ OR "European Union".ti,ab OR "European Community".ti,ab OR "European Coal and Steel Community".ti,ab OR "Common Market".ti,ab OR "EEC".ti,ab OR "European Economic Community".ti,ab OR "European Common Market".ti,ab OR "European Economic Area".ti,ab OR exp "Europe"/ OR "Albania"/ OR "Andorra"/ OR "Armenia"/ OR "Armenia"/ OR "Austria"/ OR "Azerbaijan"/ OR "Republic of Belarus"/ OR "Belgium"/ OR "Bosnia and Herzegovina"/ OR "Bulgaria"/ OR "Croatia"/ OR "Czech Republic"/ OR "Denmark"/ OR "England"/ OR "Estonia"/ OR "Finland"/ OR "France"/ OR "Georgia"/ OR "Germany"/ OR "Gibraltar"/ OR "Greece"/ OR "Hungary"/ OR "Iceland"/ OR "Ireland"/ OR "Italy"/ OR "Kazakhstan"/ OR "Kosovo"/ OR "Kyrgyzstan"/ OR "Latvia"/ OR "Liechtenstein"/ OR "Lithuania"/ OR "Luxembourg"/ OR "Malta"/ OR "Moldova"/ OR "Monaco"/ OR "Montenegro"/ OR "Netherlands"/ OR "Republic of North Macedonia"/ OR "Northern Ireland"/ OR "Norway"/ OR "Poland"/ OR "Portugal"/ OR "Romania"/ OR "Russia"/ OR "San Marino"/ OR "Scotland"/ OR "Serbia"/ OR "Slovakia"/ OR "Slovenia"/ OR "Spain"/ OR "Sweden"/ OR "Switzerland"/ OR "Turkey"/ OR "Ukraine"/ OR "United Kingdom"/ OR "Uzbekistan"/ OR "Vatican City"/ OR "Wales"/ OR "Europe".ti,ab OR "European".ti,ab OR "Albania".ti,ab OR "Andorra".ti,ab OR "Armenia".ti,ab OR "Armenia".ti,ab OR "Austria".ti,ab OR "Azerbaijan".ti,ab OR "Belarus".ti,ab OR "Belgium".ti,ab OR "Bosnia".ti,ab OR "Bulgaria".ti,ab OR "Croatia".ti,ab OR "Czech Republic".ti,ab OR "Denmark".ti,ab OR "England".ti,ab OR "Estonia".ti,ab OR "Finland".ti,ab OR "France".ti,ab OR "Georgia".ti,ab OR "Germany".ti,ab OR "Gibraltar".ti,ab OR "Greece".ti,ab OR "Herzegovina".ti,ab OR "Hungary".ti,ab OR "Iceland".ti,ab OR "Ireland".ti,ab OR "Italy".ti,ab OR "Kazakhstan".ti,ab OR "Kosovo".ti,ab OR "Kyrgyzstan".ti,ab OR "Latvia".ti,ab OR "Liechtenstein".ti,ab OR "Lithuania".ti,ab OR "Luxembourg".ti,ab OR "Malta".ti,ab OR "Moldova".ti,ab OR "Monaco".ti,ab OR "Montenegro".ti,ab OR "Netherlands".ti,ab OR "North Macedonia".ti,ab OR "Northern Ireland".ti,ab OR "Norway".ti,ab OR "Poland".ti,ab OR "Portugal".ti,ab OR "Romania".ti,ab OR "Russia".ti,ab OR "San

Marino".ti,ab OR "Scotland".ti,ab OR "Serbia".ti,ab OR "Slovakia".ti,ab OR "Slovenia".ti,ab OR "Spain".ti,ab OR "Sweden".ti,ab OR "Switzerland".ti,ab OR "Turkey".ti,ab OR "Ukraine".ti,ab OR "United Kingdom".ti,ab OR "Uzbekistan".ti,ab OR "Vatican City".ti,ab OR "Wales".ti,ab OR "Albanian".ti,ab OR "Armenian".ti,ab OR "Austrian".ti,ab OR "Belgian".ti,ab OR "Bosnian".ti,ab OR "Bulgarian".ti,ab OR "Croatian".ti,ab OR "Czech".ti,ab OR "Danish".ti,ab OR "British".ti,ab OR "Estonian".ti,ab OR "Finnish".ti,ab OR "French".ti,ab OR "Georgian".ti,ab OR "German".ti,ab OR "Greek".ti,ab OR "Hungarian".ti,ab OR "Icelandic".ti,ab OR "Irish".ti,ab OR "Italian".ti,ab OR "Kosovan".ti,ab OR "Latvian".ti,ab OR "Lithuanian".ti,ab OR "Moldovan".ti,ab OR "Dutch".ti,ab OR "Macedonian".ti,ab OR "Norwegian".ti,ab OR "Polish".ti,ab OR "Romanian".ti,ab OR "Russian".ti,ab OR "Scottish".ti,ab OR "Serbian".ti,ab OR "Slovakian".ti,ab OR "Slovenian".ti,ab OR "Spanish".ti,ab OR "Swedish".ti,ab OR "Swiss".ti,ab OR "Turkish".ti,ab OR "Ukrainian".ti,ab OR "Welsh".ti,ab))**)** AND (2013 OR 2014 OR 2015 OR 2016 OR 2017 OR 2018 OR 2019 OR 2020 OR 2021 OR 2022).yr

Emcare – Cardiovascular registries

(((exp *"cardiac implantable electronic device"/ OR exp *"artificial heart pacemaker"/ OR "pacemaker".ti OR "pacemakers".ti OR exp *"Artificial Heart"/ OR "artificial heart".ti OR "artificial hearts".ti OR exp *"Heart Assist Device"/ OR "Artificial Heart".ti OR "Artificial Ventricle".ti OR "Artificial Ventricles".ti OR "Heart Assist Device".ti OR "Heart Assist Devices".ti OR "Heart Assist Pump".ti OR "Heart Assist Pumps".ti OR "Vascular Assist Device".ti OR "Vascular Assist Devices".ti OR "Ventricle Assist Device".ti OR "Ventricle Assist Devices".ti OR "Ventricular Assist Device".ti OR "Ventricular Assist Devices".ti OR exp *"Heart Valve Prosthesis"/ OR "Heart Valve Prosthesis".ti OR "Heart Valve Prosthesis".ti OR "Cardiac Valve Prosthesis".ti OR "Cardiac Valve Prostheses".ti OR "Heart Prosthesis".ti OR "Heart Prosthesis".ti OR "Cardiac Prosthesis".ti OR "Cardiac Prostheses".ti OR "artificial heart valves".ti OR "artificial heart valve".ti OR "artificial valves".ti OR "artificial valves".ti OR exp *"Implantable Defibrillator"/ OR "Implantable Defibrillator".ti OR "Implantable Defibrillators".ti OR "Implantable Cardioverter Defibrillator".ti OR "Implantable Cardioverter Defibrillators".ti OR exp *"bioresorbable vascular stent"/ OR "bioresorbable vascular scaffold".ti OR "bioresorbable vascular scaffolds".ti OR exp *"transcatheter aortic valve implantation"/ OR "transcatheter aortic valve implantation".ti OR "transcatheter aortic valve implant".ti OR "transcatheter aortic valve implants".ti OR "TAVI".ti OR "transseptal mitral valve-in-ring".ti OR "TMVR".ti OR ("".ti) OR "LAAOC".ti OR ((exp *"Heart"/ OR "heart".ti OR "cardiac".ti) AND (exp *"Prosthesis"/ OR "Prostheses".ti OR "Prosthesis".ti OR "Implants".ti OR "Implant".ti OR "replacement".ti OR "replacements".ti))) AND (exp "Register"/ OR "register".ti,ab OR "registers".ti,ab OR "registry".ti,ab OR "registries".ti,ab OR "register".in OR

"registers".in OR "registry".in OR "registries".in) AND (exp "European Union"/ OR "European Union".ti,ab OR "European Community".ti,ab OR "European Coal and Steel Community".ti,ab OR "Common Market".ti,ab OR "EEC".ti,ab OR "European Economic Community".ti,ab OR "European Common Market".ti,ab OR "European Economic Area".ti,ab OR exp "Europe"/ OR "Albania"/ OR "Andorra"/ OR "Armenia"/ OR "Armenia"/ OR "Austria"/ OR "Azerbaijan"/ OR "Republic of Belarus"/ OR "Belgium"/ OR "Bosnia and Herzegovina"/ OR "Bulgaria"/ OR "Croatia"/ OR "Czech Republic"/ OR "Denmark"/ OR "England"/ OR "Estonia"/ OR "Finland"/ OR "France"/ OR "Georgia"/ OR "Germany"/ OR "Gibraltar"/ OR "Greece"/ OR "Hungary"/ OR "Iceland"/ OR "Ireland"/ OR "Italy"/ OR "Kazakhstan"/ OR "Kosovo"/ OR "Kyrgyzstan"/ OR "Latvia"/ OR "Liechtenstein"/ OR "Lithuania"/ OR "Luxembourg"/ OR "Malta"/ OR "Moldova"/ OR "Monaco"/ OR "Montenegro"/ OR "Netherlands"/ OR "Republic of North Macedonia"/ OR "Northern Ireland"/ OR "Norway"/ OR "Poland"/ OR "Portugal"/ OR "Romania"/ OR "Russia"/ OR "San Marino"/ OR "Scotland"/ OR "Serbia"/ OR "Slovakia"/ OR "Slovenia"/ OR "Spain"/ OR "Sweden"/ OR "Switzerland"/ OR "Turkey"/ OR "Ukraine"/ OR "United Kingdom"/ OR "Uzbekistan"/ OR "Vatican City"/ OR "Wales"/ OR "Europe".ti,ab OR "European".ti,ab OR "Albania".ti,ab OR "Andorra".ti,ab OR "Armenia".ti,ab OR "Armenia".ti,ab OR "Austria".ti,ab OR "Azerbaijan".ti,ab OR "Belarus".ti,ab OR "Belgium".ti,ab OR "Bosnia".ti,ab OR "Bulgaria".ti,ab OR "Croatia".ti,ab OR "Czech Republic".ti,ab OR "Denmark".ti,ab OR "England".ti,ab OR "Estonia".ti,ab OR "Finland".ti,ab OR "France".ti,ab OR "Georgia".ti,ab OR "Germany".ti,ab OR "Gibraltar".ti,ab OR "Greece".ti,ab OR "Herzegovina".ti,ab OR "Hungary".ti,ab OR "Iceland".ti,ab OR "Ireland".ti,ab OR "Italy".ti,ab OR "Kazakhstan".ti,ab OR "Kosovo".ti,ab OR "Kyrgyzstan".ti,ab OR "Latvia".ti,ab OR "Liechtenstein".ti,ab OR "Lithuania".ti,ab OR "Luxembourg".ti,ab OR "Malta".ti,ab OR "Moldova".ti,ab OR "Monaco".ti,ab OR "Montenegro".ti,ab OR "Netherlands".ti,ab OR "North Macedonia".ti,ab OR "Northern Ireland".ti,ab OR "Norway".ti,ab OR "Poland".ti,ab OR "Portugal".ti,ab OR "Romania".ti,ab OR "Russia".ti,ab OR "San Marino".ti,ab OR "Scotland".ti,ab OR "Serbia".ti,ab OR "Slovakia".ti,ab OR "Slovenia".ti,ab OR "Spain".ti,ab OR "Sweden".ti,ab OR "Switzerland".ti,ab OR "Turkey".ti,ab OR "Ukraine".ti,ab OR "United Kingdom".ti,ab OR "Uzbekistan".ti,ab OR "Vatican City".ti,ab OR "Wales".ti,ab OR "Albanian".ti,ab OR "Armenian".ti,ab OR "Austrian".ti,ab OR "Belgian".ti,ab OR "Bosnian".ti,ab OR "Bulgarian".ti,ab OR "Croatian".ti,ab OR "Czech".ti,ab OR "Danish".ti,ab OR "British".ti,ab OR "Estonian".ti,ab OR "Finnish".ti,ab OR "French".ti,ab OR "Georgian".ti,ab OR "German".ti,ab OR "Greek".ti,ab OR "Hungarian".ti,ab OR "Icelandic".ti,ab OR "Irish".ti,ab OR "Italian".ti,ab OR "Kosovan".ti,ab OR "Latvian".ti,ab OR "Lithuanian".ti,ab OR "Moldovan".ti,ab OR "Dutch".ti,ab OR "Macedonian".ti,ab OR "Norwegian".ti,ab OR "Polish".ti,ab OR "Romanian".ti,ab OR "Russian".ti,ab OR "Scottish".ti,ab OR "Serbian".ti,ab OR "Slovakian".ti,ab OR "Slovenian".ti,ab OR "Spanish".ti,ab OR "Swedish".ti,ab OR "Swiss".ti,ab OR "Turkish".ti,ab OR "Ukrainian".ti,ab OR

"Welsh".ti,ab)) **OR** ((exp *"cardiac implantable electronic device"/ OR exp *"artificial heart pacemaker"/ OR "pacemaker".ti,ab OR "pacemakers".ti,ab OR exp *"Artificial Heart"/ OR "artificial heart".ti,ab OR "artificial hearts".ti,ab OR exp *"Heart Assist Device"/ OR "Artificial Heart".ti,ab OR "Artificial Ventricle".ti,ab OR "Artificial Ventricles".ti,ab OR "Heart Assist Device".ti,ab OR "Heart Assist Devices".ti,ab OR "Heart Assist Pump".ti,ab OR "Heart Assist Pumps".ti,ab OR "Vascular Assist Device".ti,ab OR "Vascular Assist Devices".ti,ab OR "Ventricle Assist Device".ti,ab OR "Ventricle Assist Devices".ti,ab OR "Ventricular Assist Device".ti,ab OR "Ventricular Assist Devices".ti,ab OR exp *"Heart Valve Prosthesis"/ OR "Heart Valve Prosthesis".ti,ab OR "Heart Valve Prosthesis".ti,ab OR "Cardiac Valve Prosthesis".ti,ab OR "Cardiac Valve Prostheses".ti,ab OR "Heart Prosthesis".ti,ab OR "Heart Prosthesis".ti,ab OR "Cardiac Prosthesis".ti,ab OR "Cardiac Prostheses".ti,ab OR "artificial heart valves".ti,ab OR "artificial heart valve".ti,ab OR "artificial valves".ti,ab OR "artificial valves".ti,ab OR exp *"Implantable Defibrillator"/ OR "Implantable Defibrillator".ti,ab OR "Implantable Defibrillators".ti,ab OR "Implantable Cardioverter Defibrillator".ti,ab OR "Implantable Cardioverter Defibrillators".ti,ab OR exp *"bioresorbable vascular stent"/ OR "bioresorbable vascular scaffold".ti,ab OR "bioresorbable vascular scaffolds".ti,ab OR exp *"transcatheter aortic valve implantation"/ OR "transcatheter aortic valve implantation".ti,ab OR "transcatheter aortic valve implant".ti,ab OR "transcatheter aortic valve implants".ti,ab OR "TAVI".ti,ab OR "transseptal mitral valve-in-ring".ti,ab OR "TMVR".ti,ab OR ("".ti,ab) OR "LAAOC".ti,ab OR ((exp *"Heart"/ OR "heart".ti,ab OR "cardiac".ti,ab) AND (exp *"Prosthesis"/ OR "Prostheses".ti,ab OR "Prosthesis".ti,ab OR "Implants".ti,ab OR "Implant".ti,ab OR "replacement".ti,ab OR "replacements".ti,ab))) AND (exp *"Register"/ OR "register".ti OR "registers".ti OR "registry".ti OR "registries".ti OR "register".in OR "registers".in OR "registry".in OR "registries".in) AND (exp "European Union"/ OR "European Union".ti,ab OR "European Community".ti,ab OR "European Coal and Steel Community".ti,ab OR "Common Market".ti,ab OR "EEC".ti,ab OR "European Economic Community".ti,ab OR "European Common Market".ti,ab OR "European Economic Area".ti,ab OR exp "Europe"/ OR "Albania"/ OR "Andorra"/ OR "Armenia"/ OR "Armenia"/ OR "Austria"/ OR "Azerbaijan"/ OR "Republic of Belarus"/ OR "Belgium"/ OR "Bosnia and Herzegovina"/ OR "Bulgaria"/ OR "Croatia"/ OR "Czech Republic"/ OR "Denmark"/ OR "England"/ OR "Estonia"/ OR "Finland"/ OR "France"/ OR "Georgia"/ OR "Germany"/ OR "Gibraltar"/ OR "Greece"/ OR "Hungary"/ OR "Iceland"/ OR "Ireland"/ OR "Italy"/ OR "Kazakhstan"/ OR "Kosovo"/ OR "Kyrgyzstan"/ OR "Latvia"/ OR "Liechtenstein"/ OR "Lithuania"/ OR "Luxembourg"/ OR "Malta"/ OR "Moldova"/ OR "Monaco"/ OR "Montenegro"/ OR "Netherlands"/ OR "Republic of North Macedonia"/ OR "Northern Ireland"/ OR "Norway"/ OR "Poland"/ OR "Portugal"/ OR "Romania"/ OR "Russia"/ OR "San Marino"/ OR "Scotland"/ OR "Serbia"/ OR "Slovakia"/ OR "Slovenia"/ OR "Spain"/ OR "Sweden"/ OR "Switzerland"/ OR "Turkey"/ OR "Ukraine"/ OR "United Kingdom"/ OR "Uzbekistan"/ OR "Vatican

City"/ OR "Wales"/ OR "Europe".ti,ab OR "European".ti,ab OR "Albania".ti,ab OR "Andorra".ti,ab OR "Armenia".ti,ab OR "Armenia".ti,ab OR "Austria".ti,ab OR "Azerbaijan".ti,ab OR "Belarus".ti,ab OR "Belgium".ti,ab OR "Bosnia".ti,ab OR "Bulgaria".ti,ab OR "Croatia".ti,ab OR "Czech Republic".ti,ab OR "Denmark".ti,ab OR "England".ti,ab OR "Estonia".ti,ab OR "Finland".ti,ab OR "France".ti,ab OR "Georgia".ti,ab OR "Germany".ti,ab OR "Gibraltar".ti,ab OR "Greece".ti,ab OR "Herzegovina".ti,ab OR "Hungary".ti,ab OR "Iceland".ti,ab OR "Ireland".ti,ab OR "Italy".ti,ab OR "Kazakhstan".ti,ab OR "Kosovo".ti,ab OR "Kyrgyzstan".ti,ab OR "Latvia".ti,ab OR "Liechtenstein".ti,ab OR "Lithuania".ti,ab OR "Luxembourg".ti,ab OR "Malta".ti,ab OR "Moldova".ti,ab OR "Monaco".ti,ab OR "Montenegro".ti,ab OR "Netherlands".ti,ab OR "North Macedonia".ti,ab OR "Northern Ireland".ti,ab OR "Norway".ti,ab OR "Poland".ti,ab OR "Portugal".ti,ab OR "Romania".ti,ab OR "Russia".ti,ab OR "San Marino".ti,ab OR "Scotland".ti,ab OR "Serbia".ti,ab OR "Slovakia".ti,ab OR "Slovenia".ti,ab OR "Spain".ti,ab OR "Sweden".ti,ab OR "Switzerland".ti,ab OR "Turkey".ti,ab OR "Ukraine".ti,ab OR "United Kingdom".ti,ab OR "Uzbekistan".ti,ab OR "Vatican City".ti,ab OR "Wales".ti,ab OR "Albanian".ti,ab OR "Armenian".ti,ab OR "Austrian".ti,ab OR "Belgian".ti,ab OR "Bosnian".ti,ab OR "Bulgarian".ti,ab OR "Croatian".ti,ab OR "Czech".ti,ab OR "Danish".ti,ab OR "British".ti,ab OR "Estonian".ti,ab OR "Finnish".ti,ab OR "French".ti,ab OR "Georgian".ti,ab OR "German".ti,ab OR "Greek".ti,ab OR "Hungarian".ti,ab OR "Icelandic".ti,ab OR "Irish".ti,ab OR "Italian".ti,ab OR "Kosovan".ti,ab OR "Latvian".ti,ab OR "Lithuanian".ti,ab OR "Moldovan".ti,ab OR "Dutch".ti,ab OR "Macedonian".ti,ab OR "Norwegian".ti,ab OR "Polish".ti,ab OR "Romanian".ti,ab OR "Russian".ti,ab OR "Scottish".ti,ab OR "Serbian".ti,ab OR "Slovakian".ti,ab OR "Slovenian".ti,ab OR "Spanish".ti,ab OR "Swedish".ti,ab OR "Swiss".ti,ab OR "Turkish".ti,ab OR "Ukrainian".ti,ab OR "Welsh".ti,ab))**)** AND (2013 OR 2014 OR 2015 OR 2016 OR 2017 OR 2018 OR 2019 OR 2020 OR 2021 OR 2022).yr

Medline – Orthopaedic registries

(((exp *Arthroplasty, Replacement, Hip/ OR exp *Hip Prosthesis/ OR hip replacement.ti. OR hip replacement*.ti. OR hip arthroplasty.ti. OR hip arthroplast*.ti. OR hip prosthesis.ti. OR hip prosthe*.ti. OR THA.ti. OR THR.ti. OR hip implant.ti. OR hip implants.ti. OR exp *Arthroplasty, Replacement, Knee/ OR exp *Knee Prosthesis/ OR knee replacement.ti. OR knee replacement*.ti. OR knee arthroplasty.ti. OR knee arthroplast*.ti. OR knee prosthesis.ti. OR knee prosthe*.ti. OR TKA.ti. OR TKR.ti. OR knee implant.ti. OR knee implants.ti. OR exp *Arthroplasty, Replacement, Shoulder/ OR exp *Shoulder Prosthesis/ OR shoulder replacement.ti. OR shoulder replacement*.ti. OR shoulder arthroplasty.ti. OR shoulder arthroplast*.ti. OR shoulder prosthesis.ti. OR shoulder prosthe*.ti. OR shoulder implant.ti. OR knee implants.ti. OR exp *Arthroplasty, Replacement, Ankle/ OR exp *Ankle

Prosthesis/ OR ankle replacement.ti. OR ankle replacement*.ti. OR ankle arthroplasty.ti. OR ankle arthroplast*.ti. OR ankle prosthesis.ti. OR ankle prosthe*.ti. OR ankle implant.ti. OR ankle implants.ti. OR ((exp *Hip/ OR exp *Hip Joint/ OR hip.ti. OR hips.ti. OR exp *Knee/ OR exp *Knee Joint/ OR knee.ti. OR knees.ti. OR exp *Shoulder/ OR exp *Shoulder Joint/ OR Shoulder.ti. OR Shoulders.ti. OR exp *Ankle/ OR exp *Ankle Joint/ OR ankle.ti. OR ankles.ti.) AND (exp *Prostheses and Implants/ OR Prostheses.ti. OR Prosthesis.ti. OR Implants.ti. OR Implant.ti. OR replacement.ti. OR replacements.ti. OR arthroplasty.ti. OR arthroplast*.ti.))) AND (exp Registries/ OR register.mp. OR registers.mp. OR registry.mp. OR registries.mp. OR register.in OR registers.in OR registry.in OR registries.in) AND (exp European Union/ OR European Union.mp. OR European Community.mp. OR European Coal and Steel Community.mp. OR Common Market.mp. OR EEC.mp. OR European Economic Community.mp. OR European Common Market.mp. OR European Economic Area.mp. OR exp Europe/ OR exp Albania/ OR exp Andorra/ OR exp Armenia/ OR exp Armenia/ OR exp Austria/ OR exp Azerbaijan/ OR exp Republic of Belarus/ OR exp Belgium/ OR exp Bosnia and Herzegovina/ OR exp Bulgaria/ OR exp Croatia/ OR exp Czech Republic/ OR exp Denmark/ OR exp England/ OR exp Estonia/ OR exp Finland/ OR exp France/ OR exp Georgia/ OR exp Germany/ OR exp Gibraltar/ OR exp Greece/ OR exp Hungary/ OR exp Iceland/ OR exp Ireland/ OR exp Italy/ OR exp Kazakhstan/ OR exp Kosovo/ OR exp Kyrgyzstan/ OR exp Latvia/ OR exp Liechtenstein/ OR exp Lithuania/ OR exp Luxembourg/ OR exp Malta/ OR exp Moldova/ OR exp Monaco/ OR exp Montenegro/ OR exp Netherlands/ OR exp Republic of North Macedonia/ OR exp Northern Ireland/ OR exp Norway/ OR exp Poland/ OR exp Portugal/ OR exp Romania/ OR exp Russia/ OR exp San Marino/ OR exp Scotland/ OR exp Serbia/ OR exp Slovakia/ OR exp Slovenia/ OR exp Spain/ OR exp Sweden/ OR exp Switzerland/ OR exp Turkey/ OR exp Ukraine/ OR exp United Kingdom/ OR exp Uzbekistan/ OR exp Vatican City/ OR exp Wales/ OR Europe.mp. OR European.mp. OR Albania.mp. OR Andorra.mp. OR Armenia.mp. OR Armenia.mp. OR Austria.mp. OR Azerbaijan.mp. OR Belarus.mp. OR Belgium.mp. OR Bosnia.mp. OR Bulgaria.mp. OR Croatia.mp. OR Czech Republic.mp. OR Denmark.mp. OR England.mp. OR Estonia.mp. OR Finland.mp. OR France.mp. OR Georgia.mp. OR Germany.mp. OR Gibraltar.mp. OR Greece.mp. OR Herzegovina.mp. OR Hungary.mp. OR Iceland.mp. OR Ireland.mp. OR Italy.mp. OR Kazakhstan.mp. OR Kosovo.mp. OR Kyrgyzstan.mp. OR Latvia.mp. OR Liechtenstein.mp. OR Lithuania.mp. OR Luxembourg.mp. OR Malta.mp. OR Moldova.mp. OR Monaco.mp. OR Montenegro.mp. OR Netherlands.mp. OR North Macedonia.mp. OR Northern Ireland.mp. OR Norway.mp. OR Poland.mp. OR Portugal.mp. OR Romania.mp. OR Russia.mp. OR San Marino.mp. OR Scotland.mp. OR Serbia.mp. OR Slovakia.mp. OR Slovenia.mp. OR Spain.mp. OR Sweden.mp. OR Switzerland.mp. OR Turkey.mp. OR Ukraine.mp. OR United Kingdom.mp. OR Uzbekistan.mp. OR Vatican City.mp. OR Wales.mp. OR "Albanian".mp OR "Armenian".mp OR "Austrian".mp OR "Belgian".mp OR "Bosnian".mp OR "Bulgarian".mp OR

"Croatian".mp OR "Czech".mp OR "Danish".mp OR "British".mp OR "Estonian".mp OR "Finnish".mp OR "French".mp OR "Georgian".mp OR "German".mp OR "Greek".mp OR "Hungarian".mp OR "Icelandic".mp OR "Irish".mp OR "Italian".mp OR "Kosovan".mp OR "Latvian".mp OR "Lithuanian".mp OR "Moldovan".mp OR "Dutch".mp OR "Macedonian".mp OR "Norwegian".mp OR "Polish".mp OR "Romanian".mp OR "Russian".mp OR "Scottish".mp OR "Serbian".mp OR "Slovakian".mp OR "Slovenian".mp OR "Spanish".mp OR "Swedish".mp OR "Swiss".mp OR "Turkish".mp OR "Ukrainian".mp OR "Welsh".mp)) OR ((exp Arthroplasty, Replacement, Hip/ OR exp Hip Prosthesis/ OR hip replacement.mp. OR hip replacement*.mp. OR hip arthroplasty.mp. OR hip arthroplast*.mp. OR hip prosthesis.mp. OR hip prosthe*.mp. OR THA.mp. OR THR.mp. OR hip implant.mp. OR hip implants.mp. OR exp Arthroplasty, Replacement, Knee/ OR exp Knee Prosthesis/ OR knee replacement.mp. OR knee replacement*.mp. OR knee arthroplasty.mp. OR knee arthroplast*.mp. OR knee prosthesis.mp. OR knee prosthe*.mp. OR TKA.mp. OR TKR.mp. OR knee implant.mp. OR knee implants.mp. OR exp Arthroplasty, Replacement, Shoulder/ OR exp Shoulder Prosthesis/ OR shoulder replacement.mp. OR shoulder replacement*.mp. OR shoulder arthroplasty.mp. OR shoulder arthroplast*.mp. OR shoulder prosthesis.mp. OR shoulder prosthe*.mp. OR shoulder implant.mp. OR knee implants.mp. OR exp Arthroplasty, Replacement, Ankle/ OR exp Ankle Prosthesis/ OR ankle replacement.mp. OR ankle replacement*.mp. OR ankle arthroplasty.mp. OR ankle arthroplast*.mp. OR ankle prosthesis.mp. OR ankle prosthe*.mp. OR ankle implant.mp. OR ankle implants.mp. OR ((exp Hip/ OR exp Hip Joint/ OR hip.mp. OR hips.mp. OR exp Knee/ OR exp Knee Joint/ OR knee.mp. OR knees.mp. OR exp Shoulder/ OR exp Shoulder Joint/ OR Shoulder.mp. OR Shoulders.mp. OR exp Ankle/ OR exp Ankle Joint/ OR ankle.mp. OR ankles.mp.) AND (exp Prostheses and Implants/ OR Prostheses.mp. OR Prosthesis.mp. OR Implants.mp. OR Implant.mp. OR replacement.mp. OR replacements.mp. OR arthroplasty.mp. OR arthroplast*.mp.))) AND (exp *Registries/ OR register.ti. OR registers.ti. OR registry.ti. OR registries.ti. OR register.in OR registers.in OR registry.in OR registries.in) AND (exp European Union/ OR European Union.mp. OR European Community.mp. OR European Coal and Steel Community.mp. OR Common Market.mp. OR EEC.mp. OR European Economic Community.mp. OR European Common Market.mp. OR European Economic Area.mp. OR exp Europe/ OR exp Albania/ OR exp Andorra/ OR exp Armenia/ OR exp Armenia/ OR exp Austria/ OR exp Azerbaijan/ OR exp Republic of Belarus/ OR exp Belgium/ OR exp Bosnia and Herzegovina/ OR exp Bulgaria/ OR exp Croatia/ OR exp Czech Republic/ OR exp Denmark/ OR exp England/ OR exp Estonia/ OR exp Finland/ OR exp France/ OR exp Georgia/ OR exp Germany/ OR exp Gibraltar/ OR exp Greece/ OR exp Hungary/ OR exp Iceland/ OR exp Ireland/ OR exp Italy/ OR exp Kazakhstan/ OR exp Kosovo/ OR exp Kyrgyzstan/ OR exp Latvia/ OR exp Liechtenstein/ OR exp Lithuania/ OR exp Luxembourg/ OR exp Malta/ OR exp Moldova/ OR exp Monaco/ OR exp Montenegro/ OR exp Netherlands/ OR exp

Republic of North Macedonia/ OR exp Northern Ireland/ OR exp Norway/ OR exp Poland/ OR exp Portugal/ OR exp Romania/ OR exp Russia/ OR exp San Marino/ OR exp Scotland/ OR exp Serbia/ OR exp Slovakia/ OR exp Slovenia/ OR exp Spain/ OR exp Sweden/ OR exp Switzerland/ OR exp Turkey/ OR exp Ukraine/ OR exp United Kingdom/ OR exp Uzbekistan/ OR exp Vatican City/ OR exp Wales/ OR Europe.mp. OR European.mp. OR Albania.mp. OR Andorra.mp. OR Armenia.mp. OR Armenia.mp. OR Austria.mp. OR Azerbaijan.mp. OR Belarus.mp. OR Belgium.mp. OR Bosnia.mp. OR Bulgaria.mp. OR Croatia.mp. OR Czech Republic.mp. OR Denmark.mp. OR England.mp. OR Estonia.mp. OR Finland.mp. OR France.mp. OR Georgia.mp. OR Germany.mp. OR Gibraltar.mp. OR Greece.mp. OR Herzegovina.mp. OR Hungary.mp. OR Iceland.mp. OR Ireland.mp. OR Italy.mp. OR Kazakhstan.mp. OR Kosovo.mp. OR Kyrgyzstan.mp. OR Latvia.mp. OR Liechtenstein.mp. OR Lithuania.mp. OR Luxembourg.mp. OR Malta.mp. OR Moldova.mp. OR Monaco.mp. OR Montenegro.mp. OR Netherlands.mp. OR North Macedonia.mp. OR Northern Ireland.mp. OR Norway.mp. OR Poland.mp. OR Portugal.mp. OR Romania.mp. OR Russia.mp. OR San Marino.mp. OR Scotland.mp. OR Serbia.mp. OR Slovakia.mp. OR Slovenia.mp. OR Spain.mp. OR Sweden.mp. OR Switzerland.mp. OR Turkey.mp. OR Ukraine.mp. OR United Kingdom.mp. OR Uzbekistan.mp. OR Vatican City.mp. OR Wales.mp. OR "Albanian".mp OR "Armenian".mp OR "Austrian".mp OR "Belgian".mp OR "Bosnian".mp OR "Bulgarian".mp OR "Croatian".mp OR "Czech".mp OR "Danish".mp OR "British".mp OR "Estonian".mp OR "Finnish".mp OR "French".mp OR "Georgian".mp OR "German".mp OR "Greek".mp OR "Hungarian".mp OR "Icelandic".mp OR "Irish".mp OR "Italian".mp OR "Kosovan".mp OR "Latvian".mp OR "Lithuanian".mp OR "Moldovan".mp OR "Dutch".mp OR "Macedonian".mp OR "Norwegian".mp OR "Polish".mp OR "Romanian".mp OR "Russian".mp OR "Scottish".mp OR "Serbian".mp OR "Slovakian".mp OR "Slovenian".mp OR "Spanish".mp OR "Swedish".mp OR "Swiss".mp OR "Turkish".mp OR "Ukrainian".mp OR "Welsh".mp))) AND (2013 OR 2014 OR 2015 OR 2016 OR 2017 OR 2018 OR 2019 OR 2020 OR 2021 OR 2022).yr

Medline – Cardiovascular registries

(((exp *Pacemaker, Artificial/ OR pacemaker.ti. OR pacemakers.ti. OR exp *Heart, Artificial/ OR artificial heart.ti. OR artificial hearts.ti. OR exp *Heart-Assist Devices/ OR Artificial Heart .ti. OR Artificial Ventricle.ti. OR Artificial Ventricles.ti. OR Heart Assist Device.ti. OR Heart Assist Devices.ti. OR Heart Assist Pump.ti. OR Heart Assist Pumps.ti. OR Vascular Assist Device.ti. OR Vascular Assist Devices.ti. OR Ventricle Assist Device.ti. OR Ventricle Assist Devices.ti. OR Ventricular Assist Device.ti. OR Ventricular Assist Devices.ti. OR exp *Heart Valve Prosthesis/ OR Heart Valve Prosthesis.ti. OR Heart Valve Prosthesis.ti. OR Cardiac Valve Prosthesis.ti. OR Cardiac Valve

Prostheses.ti. OR Heart Prosthesis.ti. OR Heart Prosthesis.ti. OR Cardiac Prosthesis.ti. OR Cardiac Prostheses.ti. OR artificial heart valves.ti. OR artificial heart valve.ti. OR artificial valves.ti. OR artificial valves.ti. OR exp *Defibrillators, Implantable/ OR Implantable Defibrillator.ti. OR Implantable Defibrillators.ti. OR Implantable Cardioverter Defibrillator.ti. OR Implantable Cardioverter Defibrillators.ti. OR bioresorbable vascular scaffold.ti. OR bioresorbable vascular scaffolds.ti. OR transcatheter aortic valve implantation.ti. OR transcatheter aortic valve implant.ti. OR transcatheter aortic valve implants.ti. OR TAVI.ti. OR transseptal mitral valve-in-ring.ti. OR TMVR.ti. OR (percutaneous.ti. AND left anterior.ti. AND aortic cusp.ti.) OR LAAOC.ti. OR ((exp *Heart/ OR heart.ti. OR cardiac.ti.) AND (exp *Prostheses and Implants/ OR Prostheses.ti. OR Prosthesis.ti. OR Implants.ti. OR Implant.ti. OR replacement.ti. OR replacements.ti.))) AND (exp Registries/ OR register.mp. OR registers.mp. OR registry.mp. OR registries.mp. OR register.in OR registers.in OR registry.in OR registries.in) AND (exp European Union/ OR European Union.mp. OR European Community.mp. OR European Coal and Steel Community.mp. OR Common Market.mp. OR EEC.mp. OR European Economic Community.mp. OR European Common Market.mp. OR European Economic Area.mp. OR exp Europe/ OR exp Albania/ OR exp Andorra/ OR exp Armenia/ OR exp Armenia/ OR exp Austria/ OR exp Azerbaijan/ OR exp Republic of Belarus/ OR exp Belgium/ OR exp Bosnia and Herzegovina/ OR exp Bulgaria/ OR exp Croatia/ OR exp Czech Republic/ OR exp Denmark/ OR exp England/ OR exp Estonia/ OR exp Finland/ OR exp France/ OR exp Georgia/ OR exp Germany/ OR exp Gibraltar/ OR exp Greece/ OR exp Hungary/ OR exp Iceland/ OR exp Ireland/ OR exp Italy/ OR exp Kazakhstan/ OR exp Kosovo/ OR exp Kyrgyzstan/ OR exp Latvia/ OR exp Liechtenstein/ OR exp Lithuania/ OR exp Luxembourg/ OR exp Malta/ OR exp Moldova/ OR exp Monaco/ OR exp Montenegro/ OR exp Netherlands/ OR exp Republic of North Macedonia/ OR exp Northern Ireland/ OR exp Norway/ OR exp Poland/ OR exp Portugal/ OR exp Romania/ OR exp Russia/ OR exp San Marino/ OR exp Scotland/ OR exp Serbia/ OR exp Slovakia/ OR exp Slovenia/ OR exp Spain/ OR exp Sweden/ OR exp Switzerland/ OR exp Turkey/ OR exp Ukraine/ OR exp United Kingdom/ OR exp Uzbekistan/ OR exp Vatican City/ OR exp Wales/ OR Europe.mp. OR European.mp. OR Albania.mp. OR Andorra.mp. OR Armenia.mp. OR Armenia.mp. OR Austria.mp. OR Azerbaijan.mp. OR Belarus.mp. OR Belgium.mp. OR Bosnia.mp. OR Bulgaria.mp. OR Croatia.mp. OR Czech Republic.mp. OR Denmark.mp. OR England.mp. OR Estonia.mp. OR Finland.mp. OR France.mp. OR Georgia.mp. OR Germany.mp. OR Gibraltar.mp. OR Greece.mp. OR Herzegovina.mp. OR Hungary.mp. OR Iceland.mp. OR Ireland.mp. OR Italy.mp. OR Kazakhstan.mp. OR Kosovo.mp. OR Kyrgyzstan.mp. OR Latvia.mp. OR Liechtenstein.mp. OR Lithuania.mp. OR Luxembourg.mp. OR Malta.mp. OR Moldova.mp. OR Monaco.mp. OR Montenegro.mp. OR Netherlands.mp. OR North Macedonia.mp. OR Northern Ireland.mp. OR Norway.mp. OR Poland.mp. OR Portugal.mp. OR Romania.mp. OR Russia.mp. OR San Marino.mp. OR Scotland.mp. OR Serbia.mp. OR Slovakia.mp. OR

Slovenia.mp. OR Spain.mp. OR Sweden.mp. OR Switzerland.mp. OR Turkey.mp. OR Ukraine.mp. OR United Kingdom.mp. OR Uzbekistan.mp. OR Vatican City.mp. OR Wales.mp. OR Albanian.mp. OR Armenian.mp. OR Austrian.mp. OR Belgian.mp. OR Bosnian.mp. OR Bulgarian.mp. OR Croatian.mp. OR Czech.mp. OR Danish.mp. OR British.mp. OR Estonian.mp. OR Finnish.mp. OR French.mp. OR Georgian.mp. OR German.mp. OR Greek.mp. OR Hungarian.mp. OR Icelandic.mp. OR Irish.mp. OR Italian.mp. OR Kosovan.mp. OR Latvian.mp. OR Lithuanian.mp. OR Moldovan.mp. OR Dutch.mp. OR Macedonian.mp. OR Norwegian.mp. OR Polish.mp. OR Romanian.mp. OR Russian.mp. OR Scottish.mp. OR Serbian.mp. OR Slovakian.mp. OR Slovenian.mp. OR Spanish.mp. OR Swedish.mp. OR Swiss.mp. OR Turkish.mp. OR Ukrainian.mp. OR Welsh.mp.)) OR ((exp Pacemaker, Artificial/ OR pacemaker.mp. OR pacemakers.mp. OR exp Heart, Artificial/ OR artificial heart.mp. OR artificial hearts.mp. OR exp Heart-Assist Devices/ OR Artificial Heart .mp. OR Artificial Ventricle.mp. OR Artificial Ventricles.mp. OR Heart Assist Device.mp. OR Heart Assist Devices.mp. OR Heart Assist Pump.mp. OR Heart Assist Pumps.mp. OR Vascular Assist Device.mp. OR Vascular Assist Devices.mp. OR Ventricle Assist Device.mp. OR Ventricle Assist Devices.mp. OR Ventricular Assist Device.mp. OR Ventricular Assist Devices.mp. OR exp Heart Valve Prosthesis/ OR Heart Valve Prosthesis.mp. OR Heart Valve Prosthesis.mp. OR Cardiac Valve Prosthesis.mp. OR Cardiac Valve Prostheses.mp. OR Heart Prosthesis.mp. OR Heart Prosthesis.mp. OR Cardiac Prosthesis.mp. OR Cardiac Prostheses.mp. OR artificial heart valves.mp. OR artificial heart valve.mp. OR artificial valves.mp. OR artificial valves.mp. OR exp Defibrillators, Implantable/ OR Implantable Defibrillator.mp. OR Implantable Defibrillators.mp. OR Implantable Cardioverter Defibrillator.mp. OR Implantable Cardioverter Defibrillators.mp. OR bioresorbable vascular scaffold.mp. OR bioresorbable vascular scaffolds.mp. OR transcatheter aortic valve implantation.mp. OR transcatheter aortic valve implant.mp. OR transcatheter aortic valve implants.mp. OR TAVI.mp. OR transseptal mitral valve-in-ring.mp. OR TMVR.mp. OR (percutaneous.mp. AND left anterior.mp. AND aortic cusp.mp.) OR LAAOC.mp. OR ((exp Heart/ OR heart.mp. OR cardiac.mp.) AND (exp Prostheses and Implants/ OR Prostheses.mp. OR Prosthesis.mp. OR Implants.mp. OR Implant.mp. OR replacement.mp. OR replacements.mp.))) AND (exp *Registries/ OR register.ti. OR registers.ti. OR registry.ti. OR registries.ti. OR register.in OR registers.in OR registry.in OR registries.in) AND (exp European Union/ OR European Union.mp. OR European Community.mp. OR European Coal and Steel Community.mp. OR Common Market.mp. OR EEC.mp. OR European Economic Community.mp. OR European Common Market.mp. OR European Economic Area.mp. OR exp Europe/ OR exp Albania/ OR exp Andorra/ OR exp Armenia/ OR exp Armenia/ OR exp Austria/ OR exp Azerbaijan/ OR exp Republic of Belarus/ OR exp Belgium/ OR exp Bosnia and Herzegovina/ OR exp Bulgaria/ OR exp Croatia/ OR exp Czech Republic/ OR exp Denmark/ OR exp England/ OR exp Estonia/ OR exp Finland/ OR exp France/ OR exp Georgia/ OR exp Germany/ OR exp

Gibraltar/ OR exp Greece/ OR exp Hungary/ OR exp Iceland/ OR exp Ireland/ OR exp Italy/ OR exp Kazakhstan/ OR exp Kosovo/ OR exp Kyrgyzstan/ OR exp Latvia/ OR exp Liechtenstein/ OR exp Lithuania/ OR exp Luxembourg/ OR exp Malta/ OR exp Moldova/ OR exp Monaco/ OR exp Montenegro/ OR exp Netherlands/ OR exp Republic of North Macedonia/ OR exp Northern Ireland/ OR exp Norway/ OR exp Poland/ OR exp Portugal/ OR exp Romania/ OR exp Russia/ OR exp San Marino/ OR exp Scotland/ OR exp Serbia/ OR exp Slovakia/ OR exp Slovenia/ OR exp Spain/ OR exp Sweden/ OR exp Switzerland/ OR exp Turkey/ OR exp Ukraine/ OR exp United Kingdom/ OR exp Uzbekistan/ OR exp Vatican City/ OR exp Wales/ OR Europe.mp. OR European.mp. OR Albania.mp. OR Andorra.mp. OR Armenia.mp. OR Armenia.mp. OR Austria.mp. OR Azerbaijan.mp. OR Belarus.mp. OR Belgium.mp. OR Bosnia.mp. OR Bulgaria.mp. OR Croatia.mp. OR Czech Republic.mp. OR Denmark.mp. OR England.mp. OR Estonia.mp. OR Finland.mp. OR France.mp. OR Georgia.mp. OR Germany.mp. OR Gibraltar.mp. OR Greece.mp. OR Herzegovina.mp. OR Hungary.mp. OR Iceland.mp. OR Ireland.mp. OR Italy.mp. OR Kazakhstan.mp. OR Kosovo.mp. OR Kyrgyzstan.mp. OR Latvia.mp. OR Liechtenstein.mp. OR Lithuania.mp. OR Luxembourg.mp. OR Malta.mp. OR Moldova.mp. OR Monaco.mp. OR Montenegro.mp. OR Netherlands.mp. OR North Macedonia.mp. OR Northern Ireland.mp. OR Norway.mp. OR Poland.mp. OR Portugal.mp. OR Romania.mp. OR Russia.mp. OR San Marino.mp. OR Scotland.mp. OR Serbia.mp. OR Slovakia.mp. OR Slovenia.mp. OR Spain.mp. OR Sweden.mp. OR Switzerland.mp. OR Turkey.mp. OR Ukraine.mp. OR United Kingdom.mp. OR Uzbekistan.mp. OR Vatican City.mp. OR Wales.mp. OR Albanian.mp. OR Armenian.mp. OR Austrian.mp. OR Belgian.mp. OR Bosnian.mp. OR Bulgarian.mp. OR Croatian.mp. OR Czech.mp. OR Danish.mp. OR British.mp. OR Estonian.mp. OR Finnish.mp. OR French.mp. OR Georgian.mp. OR German.mp. OR Greek.mp. OR Hungarian.mp. OR Icelandic.mp. OR Irish.mp. OR Italian.mp. OR Kosovan.mp. OR Latvian.mp. OR Lithuanian.mp. OR Moldovan.mp. OR Dutch.mp. OR Macedonian.mp. OR Norwegian.mp. OR Polish.mp. OR Romanian.mp. OR Russian.mp. OR Scottish.mp. OR Serbian.mp. OR Slovakian.mp. OR Slovenian.mp. OR Spanish.mp. OR Swedish.mp. OR Swiss.mp. OR Turkish.mp. OR Ukrainian.mp. OR Welsh.mp.))) AND (2013 OR 2014 OR 2015 OR 2016 OR 2017 OR 2018 OR 2019 OR 2020 OR 2021 OR 2022).yr

PubMed – Orthopaedic registries

((("Arthroplasty, Replacement, Hip"[majr] OR "Hip Prosthesis"[majr] OR "hip replacement"[ti] OR "hip replacement*"[ti] OR "hip arthroplasty"[ti] OR "hip arthroplast*"[ti] OR "hip prosthesis"[ti] OR "hip prosthe*"[ti] OR "THA"[ti] OR "THR"[ti] OR "hip implant"[ti] OR "hip implants"[ti] OR "Arthroplasty, Replacement, Knee"[majr] OR "Knee Prosthesis"[majr] OR "knee replacement"[ti] OR "knee

replacement*"[ti] OR "knee arthroplasty"[ti] OR "knee arthroplast*"[ti] OR "knee prosthesis"[ti] OR "knee prosthe*"[ti] OR "TKA"[ti] OR "TKR"[ti] OR "knee implant"[ti] OR "knee implants"[ti] OR "Arthroplasty, Replacement, Shoulder"[majr] OR "Shoulder Prosthesis"[majr] OR "shoulder replacement"[ti] OR "shoulder replacement*"[ti] OR "shoulder arthroplasty"[ti] OR "shoulder arthroplast*"[ti] OR "shoulder prosthesis"[ti] OR "shoulder prosthe*"[ti] OR "shoulder implant"[ti] OR "knee implants"[ti] OR "Arthroplasty, Replacement, Ankle"[majr] OR "Ankle Prosthesis"[majr] OR "ankle replacement"[ti] OR "ankle replacement*"[ti] OR "ankle arthroplasty"[ti] OR "ankle arthroplast*"[ti] OR "ankle prosthesis"[ti] OR "ankle prosthe*"[ti] OR "ankle implant"[ti] OR "ankle implants"[ti] OR (("Hip"[majr] OR "Hip Joint"[majr] OR "hip"[ti] OR "hips"[ti] OR "Knee"[majr] OR "Knee Joint"[majr] OR "knee"[ti] OR "knees"[ti] OR "Shoulder"[majr] OR "Shoulder Joint"[majr] OR "Shoulder"[ti] OR "Shoulders"[ti] OR "Ankle"[majr] OR "Ankle Joint"[majr] OR "ankle"[ti] OR "ankles"[ti]) AND ("Prostheses and Implants"[majr] OR "Prostheses"[ti] OR "Prosthesis"[ti] OR "Implants"[ti] OR "Implant"[ti] OR "replacement"[ti] OR "replacements"[ti] OR "arthroplasty"[ti] OR "arthroplast*"[ti]))) AND ("Registries"[Mesh] OR "register"[tw] OR "registers"[tw] OR "registry"[tw] OR "registries"[tw] OR "register"[ad] OR "registers"[ad] OR "registry"[ad] OR "registries"[ad]) AND ("European Union"[Mesh] OR "European Union"[tw] OR "European Community"[tw] OR "European Coal and Steel Community"[tw] OR "Common Market"[tw] OR "EEC"[tw] OR "European Economic Community"[tw] OR "European Common Market"[tw] OR "European Economic Area"[tw] OR "Europe"[Mesh] OR "Albania"[mesh] OR "Andorra"[mesh] OR "Armenia"[mesh] OR "Armenia"[mesh] OR "Austria"[mesh] OR "Azerbaijan"[mesh] OR "Republic of Belarus"[mesh] OR "Belgium"[mesh] OR "Bosnia and Herzegovina"[mesh] OR "Bulgaria"[mesh] OR "Croatia"[mesh] OR "Czech Republic"[mesh] OR "Denmark"[mesh] OR "England"[mesh] OR "Estonia"[mesh] OR "Finland"[mesh] OR "France"[mesh] OR "Georgia"[mesh] OR "Germany"[mesh] OR "Gibraltar"[mesh] OR "Greece"[mesh] OR "Hungary"[mesh] OR "Iceland"[mesh] OR "Ireland"[mesh] OR "Italy"[mesh] OR "Kazakhstan"[mesh] OR "Kosovo"[mesh] OR "Kyrgyzstan"[mesh] OR "Latvia"[mesh] OR "Liechtenstein"[mesh] OR "Lithuania"[mesh] OR "Luxembourg"[mesh] OR "Malta"[mesh] OR "Moldova"[mesh] OR "Monaco"[mesh] OR "Montenegro"[mesh] OR "Netherlands"[mesh] OR "Republic of North Macedonia"[mesh] OR "Northern Ireland"[mesh] OR "Norway"[mesh] OR "Poland"[mesh] OR "Portugal"[mesh] OR "Romania"[mesh] OR "Russia"[mesh] OR "San Marino"[mesh] OR "Scotland"[mesh] OR "Serbia"[mesh] OR "Slovakia"[mesh] OR "Slovenia"[mesh] OR "Spain"[mesh] OR "Sweden"[mesh] OR "Switzerland"[mesh] OR "Turkey"[Mesh] OR "Ukraine"[mesh] OR "United Kingdom"[mesh] OR "Uzbekistan"[mesh] OR "Vatican City"[mesh] OR "Wales"[mesh] OR "Europe"[tw] OR "European"[tw] OR "Albania"[tw] OR "Andorra"[tw] OR "Armenia"[tw] OR "Armenia"[tw] OR "Austria"[tw] OR "Azerbaijan"[tw] OR "Belarus"[tw] OR

"Belgium"[tw] OR "Bosnia"[tw] OR "Bulgaria"[tw] OR "Croatia"[tw] OR "Czech Republic"[tw] OR "Denmark"[tw] OR "England"[tw] OR "Estonia"[tw] OR "Finland"[tw] OR "France"[tw] OR "Georgia"[tw] OR "Germany"[tw] OR "Gibraltar"[tw] OR "Greece"[tw] OR "Herzegovina"[tw] OR "Hungary"[tw] OR "Iceland"[tw] OR "Ireland"[tw] OR "Italy"[tw] OR "Kazakhstan"[tw] OR "Kosovo"[tw] OR "Kyrgyzstan"[tw] OR "Latvia"[tw] OR "Liechtenstein"[tw] OR "Lithuania"[tw] OR "Luxembourg"[tw] OR "Malta"[tw] OR "Moldova"[tw] OR "Monaco"[tw] OR "Montenegro"[tw] OR "Netherlands"[tw] OR "North Macedonia"[tw] OR "Northern Ireland"[tw] OR "Norway"[tw] OR "Poland"[tw] OR "Portugal"[tw] OR "Romania"[tw] OR "Russia"[tw] OR "San Marino"[tw] OR "Scotland"[tw] OR "Serbia"[tw] OR "Slovakia"[tw] OR "Slovenia"[tw] OR "Spain"[tw] OR "Sweden"[tw] OR "Switzerland"[tw] OR "Turkey"[tw] OR "Ukraine"[tw] OR "United Kingdom"[tw] OR "Uzbekistan"[tw] OR "Vatican City"[tw] OR "Wales"[tw] OR "Albanian"[tw] OR "Armenian"[tw] OR "Austrian"[tw] OR "Belgian"[tw] OR "Bosnian"[tw] OR "Bulgarian"[tw] OR "Croatian"[tw] OR "Czech"[tw] OR "Danish"[tw] OR "British"[tw] OR "Estonian"[tw] OR "Finnish"[tw] OR "French"[tw] OR "Georgian"[tw] OR "German"[tw] OR "Greek"[tw] OR "Hungarian"[tw] OR "Icelandic"[tw] OR "Irish"[tw] OR "Italian"[tw] OR "Kosovan"[tw] OR "Latvian"[tw] OR "Lithuanian"[tw] OR "Moldovan"[tw] OR "Dutch"[tw] OR "Macedonian"[tw] OR "Norwegian"[tw] OR "Polish"[tw] OR "Romanian"[tw] OR "Russian"[tw] OR "Scottish"[tw] OR "Serbian"[tw] OR "Slovakian"[tw] OR "Slovenian"[tw] OR "Spanish"[tw] OR "Swedish"[tw] OR "Swiss"[tw] OR "Turkish"[tw] OR "Ukrainian"[tw] OR "Welsh"[tw])) **OR** (("Arthroplasty, Replacement, Hip"[Mesh] OR "Hip Prosthesis"[Mesh] OR "hip replacement"[tw] OR "hip replacement*"[tw] OR "hip arthroplasty"[tw] OR "hip arthroplast*"[tw] OR "hip prosthesis"[tw] OR "hip prosthe*"[tw] OR "THA"[tw] OR "THR"[tw] OR "hip implant"[tw] OR "hip implants"[tw] OR "Arthroplasty, Replacement, Knee"[Mesh] OR "Knee Prosthesis"[Mesh] OR "knee replacement"[tw] OR "knee replacement*"[tw] OR "knee arthroplasty"[tw] OR "knee arthroplast*"[tw] OR "knee prosthesis"[tw] OR "knee prosthe*"[tw] OR "TKA"[tw] OR "TKR"[tw] OR "knee implant"[tw] OR "knee implants"[tw] OR "Arthroplasty, Replacement, Shoulder"[Mesh] OR "Shoulder Prosthesis"[Mesh] OR "shoulder replacement"[tw] OR "shoulder replacement*"[tw] OR "shoulder arthroplasty"[tw] OR "shoulder arthroplast*"[tw] OR "shoulder prosthesis"[tw] OR "shoulder prosthe*"[tw] OR "shoulder implant"[tw] OR "knee implants"[tw] OR "Arthroplasty, Replacement, Ankle"[Mesh] OR "Ankle Prosthesis"[Mesh] OR "ankle replacement"[tw] OR "ankle replacement*"[tw] OR "ankle arthroplasty"[tw] OR "ankle arthroplast*"[tw] OR "ankle prosthesis"[tw] OR "ankle prosthe*"[tw] OR "ankle implant"[tw] OR "ankle implants"[tw] OR (("Hip"[mesh] OR "Hip Joint"[Mesh] OR "hip"[tw] OR "hips"[tw] OR "Knee"[mesh] OR "Knee Joint"[mesh] OR "knee"[tw] OR "knees"[tw] OR "Shoulder"[Mesh] OR "Shoulder Joint"[Mesh] OR "Shoulder"[tw] OR "Shoulders"[tw] OR "Ankle"[mesh] OR "Ankle Joint"[mesh] OR "ankle"[tw] OR

"ankles"[tw]) AND ("Prostheses and Implants"[Mesh] OR "Prostheses"[tw] OR "Prosthesis"[tw] OR "Implants"[tw] OR "Implant"[tw] OR "replacement"[tw] OR "replacements"[tw] OR "arthroplasty"[tw] OR "arthroplast*"[tw]))) AND ("Registries"[majr] OR "register"[ti] OR "registers"[ti] OR "registry"[ti] OR "registries"[ti] OR "register"[ad] OR "registers"[ad] OR "registry"[ad] OR "registries"[ad]) AND ("European Union"[Mesh] OR "European Union"[tw] OR "European Community"[tw] OR "European Coal and Steel Community"[tw] OR "Common Market"[tw] OR "EEC"[tw] OR "European Economic Community"[tw] OR "European Common Market"[tw] OR "European Economic Area"[tw] OR "Europe"[Mesh] OR "Albania"[mesh] OR "Andorra"[mesh] OR "Armenia"[mesh] OR "Armenia"[mesh] OR "Austria"[mesh] OR "Azerbaijan"[mesh] OR "Republic of Belarus"[mesh] OR "Belgium"[mesh] OR "Bosnia and Herzegovina"[mesh] OR "Bulgaria"[mesh] OR "Croatia"[mesh] OR "Czech Republic"[mesh] OR "Denmark"[mesh] OR "England"[mesh] OR "Estonia"[mesh] OR "Finland"[mesh] OR "France"[mesh] OR "Georgia"[mesh] OR "Germany"[mesh] OR "Gibraltar"[mesh] OR "Greece"[mesh] OR "Hungary"[mesh] OR "Iceland"[mesh] OR "Ireland"[mesh] OR "Italy"[mesh] OR "Kazakhstan"[mesh] OR "Kosovo"[mesh] OR "Kyrgyzstan"[mesh] OR "Latvia"[mesh] OR "Liechtenstein"[mesh] OR "Lithuania"[mesh] OR "Luxembourg"[mesh] OR "Malta"[mesh] OR "Moldova"[mesh] OR "Monaco"[mesh] OR "Montenegro"[mesh] OR "Netherlands"[mesh] OR "Republic of North Macedonia"[mesh] OR "Northern Ireland"[mesh] OR "Norway"[mesh] OR "Poland"[mesh] OR "Portugal"[mesh] OR "Romania"[mesh] OR "Russia"[mesh] OR "San Marino"[mesh] OR "Scotland"[mesh] OR "Serbia"[mesh] OR "Slovakia"[mesh] OR "Slovenia"[mesh] OR "Spain"[mesh] OR "Sweden"[mesh] OR "Switzerland"[mesh] OR "Turkey"[Mesh] OR "Ukraine"[mesh] OR "United Kingdom"[mesh] OR "Uzbekistan"[mesh] OR "Vatican City"[mesh] OR "Wales"[mesh] OR "Europe"[tw] OR "European"[tw] OR "Albania"[tw] OR "Andorra"[tw] OR "Armenia"[tw] OR "Armenia"[tw] OR "Austria"[tw] OR "Azerbaijan"[tw] OR "Belarus"[tw] OR "Belgium"[tw] OR "Bosnia"[tw] OR "Bulgaria"[tw] OR "Croatia"[tw] OR "Czech Republic"[tw] OR "Denmark"[tw] OR "England"[tw] OR "Estonia"[tw] OR "Finland"[tw] OR "France"[tw] OR "Georgia"[tw] OR "Germany"[tw] OR "Gibraltar"[tw] OR "Greece"[tw] OR "Herzegovina"[tw] OR "Hungary"[tw] OR "Iceland"[tw] OR "Ireland"[tw] OR "Italy"[tw] OR "Kazakhstan"[tw] OR "Kosovo"[tw] OR "Kyrgyzstan"[tw] OR "Latvia"[tw] OR "Liechtenstein"[tw] OR "Lithuania"[tw] OR "Luxembourg"[tw] OR "Malta"[tw] OR "Moldova"[tw] OR "Monaco"[tw] OR "Montenegro"[tw] OR "Netherlands"[tw] OR "North Macedonia"[tw] OR "Northern Ireland"[tw] OR "Norway"[tw] OR "Poland"[tw] OR "Portugal"[tw] OR "Romania"[tw] OR "Russia"[tw] OR "San Marino"[tw] OR "Scotland"[tw] OR "Serbia"[tw] OR "Slovakia"[tw] OR "Slovenia"[tw] OR "Spain"[tw] OR "Sweden"[tw] OR "Switzerland"[tw] OR "Turkey"[tw] OR "Ukraine"[tw] OR "United Kingdom"[tw] OR "Uzbekistan"[tw] OR "Vatican City"[tw] OR "Wales"[tw] OR "Albanian"[tw] OR "Armenian"[tw] OR

"Austrian"[tw] OR "Belgian"[tw] OR "Bosnian"[tw] OR "Bulgarian"[tw] OR "Croatian"[tw] OR "Czech"[tw] OR "Danish"[tw] OR "British"[tw] OR "Estonian"[tw] OR "Finnish"[tw] OR "French"[tw] OR "Georgian"[tw] OR "German"[tw] OR "Greek"[tw] OR "Hungarian"[tw] OR "Icelandic"[tw] OR "Irish"[tw] OR "Italian"[tw] OR "Kosovan"[tw] OR "Latvian"[tw] OR "Lithuanian"[tw] OR "Moldovan"[tw] OR "Dutch"[tw] OR "Macedonian"[tw] OR "Norwegian"[tw] OR "Polish"[tw] OR "Romanian"[tw] OR "Russian"[tw] OR "Scottish"[tw] OR "Serbian"[tw] OR "Slovakian"[tw] OR "Slovenian"[tw] OR "Spanish"[tw] OR "Swedish"[tw] OR "Swiss"[tw] OR "Turkish"[tw] OR "Ukrainian"[tw] OR "Welsh"[tw]))) AND ("2013/01/01"[PDAT] : "3000/12/31"[PDAT])

PubMed – Cardiovascular registries

((("Pacemaker, Artificial"[majr] OR "pacemaker"[ti] OR "pacemakers"[ti] OR "Heart, Artificial"[majr] OR "artificial heart"[ti] OR "artificial hearts"[ti] OR "Heart-Assist Devices"[majr] OR "Artificial Heart"[ti] OR "Artificial Ventricle"[ti] OR "Artificial Ventricles"[ti] OR "Heart Assist Device"[ti] OR "Heart Assist Devices"[ti] OR "Heart Assist Pump"[ti] OR "Heart Assist Pumps"[ti] OR "Vascular Assist Device"[ti] OR "Vascular Assist Devices"[ti] OR "Ventricle Assist Device"[ti] OR "Ventricle Assist Devices"[ti] OR "Ventricular Assist Device"[ti] OR "Ventricular Assist Devices"[ti] OR "Heart Valve Prosthesis"[majr] OR "Heart Valve Prosthesis"[ti] OR "Heart Valve Prosthesis"[ti] OR "Cardiac Valve Prosthesis"[ti] OR "Cardiac Valve Prostheses"[ti] OR "Heart Prosthesis"[ti] OR "Heart Prosthesis"[ti] OR "Cardiac Prosthesis"[ti] OR "Cardiac Prostheses"[ti] OR "artificial heart valves"[ti] OR "artificial heart valve"[ti] OR "artificial valves"[ti] OR "artificial valves"[ti] OR "Defibrillators, Implantable"[majr] OR "Implantable Defibrillator"[ti] OR "Implantable Defibrillators"[ti] OR "Implantable Cardioverter Defibrillator"[ti] OR "Implantable Cardioverter Defibrillators"[ti] OR "bioresorbable vascular scaffold"[ti] OR "bioresorbable vascular scaffolds"[ti] OR "transcatheter aortic valve implantation"[ti] OR "transcatheter aortic valve implant"[ti] OR "transcatheter aortic valve implants"[ti] OR "TAVI"[ti] OR "transseptal mitral valve-in-ring"[ti] OR "TMVR"[ti] OR ("percutaneous"[ti] AND "left anterior"[ti] AND "aortic cusp"[ti]) OR "LAAOC"[ti] OR (("Heart"[majr] OR "heart"[ti] OR "cardiac"[ti]) AND ("Prostheses and Implants"[majr] OR "Prostheses"[ti] OR "Prosthesis"[ti] OR "Implants"[ti] OR "Implant"[ti] OR "replacement"[ti] OR "replacements"[ti]))) AND ("Registries"[Mesh] OR "register"[tw] OR "registers"[tw] OR "registry"[tw] OR "registries"[tw] OR "register"[ad] OR "registers"[ad] OR "registry"[ad] OR "registries"[ad]) AND ("European Union"[Mesh] OR "European Union"[tw] OR "European Community"[tw] OR "European Coal and Steel Community"[tw] OR "Common Market"[tw] OR "EEC"[tw] OR "European Economic Community"[tw] OR "European Common Market"[tw] OR "European Economic Area"[tw] OR "Europe"[Mesh] OR "Albania"[mesh] OR "Andorra"[mesh] OR

"Armenia"[mesh] OR "Armenia"[mesh] OR "Austria"[mesh] OR "Azerbaijan"[mesh] OR "Republic of Belarus"[mesh] OR "Belgium"[mesh] OR "Bosnia and Herzegovina"[mesh] OR "Bulgaria"[mesh] OR "Croatia"[mesh] OR "Czech Republic"[mesh] OR "Denmark"[mesh] OR "England"[mesh] OR "Estonia"[mesh] OR "Finland"[mesh] OR "France"[mesh] OR "Georgia"[mesh] OR "Germany"[mesh] OR "Gibraltar"[mesh] OR "Greece"[mesh] OR "Hungary"[mesh] OR "Iceland"[mesh] OR "Ireland"[mesh] OR "Italy"[mesh] OR "Kazakhstan"[mesh] OR "Kosovo"[mesh] OR "Kyrgyzstan"[mesh] OR "Latvia"[mesh] OR "Liechtenstein"[mesh] OR "Lithuania"[mesh] OR "Luxembourg"[mesh] OR "Malta"[mesh] OR "Moldova"[mesh] OR "Monaco"[mesh] OR "Montenegro"[mesh] OR "Netherlands"[mesh] OR "Republic of North Macedonia"[mesh] OR "Northern Ireland"[mesh] OR "Norway"[mesh] OR "Poland"[mesh] OR "Portugal"[mesh] OR "Romania"[mesh] OR "Russia"[mesh] OR "San Marino"[mesh] OR "Scotland"[mesh] OR "Serbia"[mesh] OR "Slovakia"[mesh] OR "Slovenia"[mesh] OR "Spain"[mesh] OR "Sweden"[mesh] OR "Switzerland"[mesh] OR "Turkey"[mesh] OR "Ukraine"[mesh] OR "United Kingdom"[mesh] OR "Uzbekistan"[mesh] OR "Vatican City"[mesh] OR "Wales"[mesh] OR "Europe"[tw] OR "European"[tw] OR "Albania"[tw] OR "Andorra"[tw] OR "Armenia"[tw] OR "Armenia"[tw] OR "Austria"[tw] OR "Azerbaijan"[tw] OR "Belarus"[tw] OR "Belgium"[tw] OR "Bosnia"[tw] OR "Bulgaria"[tw] OR "Croatia"[tw] OR "Czech Republic"[tw] OR "Denmark"[tw] OR "England"[tw] OR "Estonia"[tw] OR "Finland"[tw] OR "France"[tw] OR "Georgia"[tw] OR "Germany"[tw] OR "Gibraltar"[tw] OR "Greece"[tw] OR "Herzegovina"[tw] OR "Hungary"[tw] OR "Iceland"[tw] OR "Ireland"[tw] OR "Italy"[tw] OR "Kazakhstan"[tw] OR "Kosovo"[tw] OR "Kyrgyzstan"[tw] OR "Latvia"[tw] OR "Liechtenstein"[tw] OR "Lithuania"[tw] OR "Luxembourg"[tw] OR "Malta"[tw] OR "Moldova"[tw] OR "Monaco"[tw] OR "Montenegro"[tw] OR "Netherlands"[tw] OR "North Macedonia"[tw] OR "Northern Ireland"[tw] OR "Norway"[tw] OR "Poland"[tw] OR "Portugal"[tw] OR "Romania"[tw] OR "Russia"[tw] OR "San Marino"[tw] OR "Scotland"[tw] OR "Serbia"[tw] OR "Slovakia"[tw] OR "Slovenia"[tw] OR "Spain"[tw] OR "Sweden"[tw] OR "Switzerland"[tw] OR "Turkey"[tw] OR "Ukraine"[tw] OR "United Kingdom"[tw] OR "Uzbekistan"[tw] OR "Vatican City"[tw] OR "Wales"[tw] OR "Albanian"[tw] OR "Armenian"[tw] OR "Austrian"[tw] OR "Belgian"[tw] OR "Bosnian"[tw] OR "Bulgarian"[tw] OR "Croatian"[tw] OR "Czech"[tw] OR "Danish"[tw] OR "British"[tw] OR "Estonian"[tw] OR "Finnish"[tw] OR "French"[tw] OR "Georgian"[tw] OR "German"[tw] OR "Greek"[tw] OR "Hungarian"[tw] OR "Icelandic"[tw] OR "Irish"[tw] OR "Italian"[tw] OR "Kosovan"[tw] OR "Latvian"[tw] OR "Lithuanian"[tw] OR "Moldovan"[tw] OR "Dutch"[tw] OR "Macedonian"[tw] OR "Norwegian"[tw] OR "Polish"[tw] OR "Romanian"[tw] OR "Russian"[tw] OR "Scottish"[tw] OR "Serbian"[tw] OR "Slovakian"[tw] OR "Slovenian"[tw] OR "Spanish"[tw] OR "Swedish"[tw] OR "Swiss"[tw] OR "Turkish"[tw] OR "Ukrainian"[tw] OR "Welsh"[tw])) **OR** (("Pacemaker, Artificial"[Mesh] OR

"pacemaker"[tw] OR "pacemakers"[tw] OR "Heart, Artificial"[Mesh] OR "artificial heart"[tw] OR "artificial hearts"[tw] OR "Heart-Assist Devices"[mesh] OR "Artificial Heart"[tw] OR "Artificial Ventricle"[tw] OR "Artificial Ventricles"[tw] OR "Heart Assist Device"[tw] OR "Heart Assist Devices"[tw] OR "Heart Assist Pump"[tw] OR "Heart Assist Pumps"[tw] OR "Vascular Assist Device"[tw] OR "Vascular Assist Devices"[tw] OR "Ventricle Assist Device"[tw] OR "Ventricle Assist Devices"[tw] OR "Ventricular Assist Device"[tw] OR "Ventricular Assist Devices"[tw] OR "Heart Valve Prosthesis"[Mesh] OR "Heart Valve Prosthesis"[tw] OR "Heart Valve Prosthesis"[tw] OR "Cardiac Valve Prosthesis"[tw] OR "Cardiac Valve Prostheses"[tw] OR "Heart Prosthesis"[tw] OR "Heart Prosthesis"[tw] OR "Cardiac Prosthesis"[tw] OR "Cardiac Prostheses"[tw] OR "artificial heart valves"[tw] OR "artificial heart valve"[tw] OR "artificial valves"[tw] OR "artificial valves"[tw] OR "Defibrillators, Implantable"[Mesh] OR "Implantable Defibrillator"[tw] OR "Implantable Defibrillators"[tw] OR "Implantable Cardioverter Defibrillator"[tw] OR "Implantable Cardioverter Defibrillators"[tw] OR "bioresorbable vascular scaffold"[tw] OR "bioresorbable vascular scaffolds"[tw] OR "transcatheter aortic valve implantation"[tw] OR "transcatheter aortic valve implant"[tw] OR "transcatheter aortic valve implants"[tw] OR "TAVI"[tw] OR "transseptal mitral valve-in-ring"[tw] OR "TMVR"[tw] OR ("percutaneous"[tw] AND "left anterior"[tw] AND "aortic cusp"[tw]) OR "LAAOC"[tw] OR (("Heart"[mesh] OR "heart"[tw] OR "cardiac"[tw]) AND ("Prostheses and Implants"[Mesh] OR "Prostheses"[tw] OR "Prosthesis"[tw] OR "Implants"[tw] OR "Implant"[tw] OR "replacement"[tw] OR "replacements"[tw]))) AND ("Registries"[majr] OR "register"[ti] OR "registers"[ti] OR "registry"[ti] OR "registries"[ti] OR "register"[ad] OR "registers"[ad] OR "registry"[ad] OR "registries"[ad]) AND ("European Union"[Mesh] OR "European Union"[tw] OR "European Community"[tw] OR "European Coal and Steel Community"[tw] OR "Common Market"[tw] OR "EEC"[tw] OR "European Economic Community"[tw] OR "European Common Market"[tw] OR "European Economic Area"[tw] OR "Europe"[Mesh] OR "Albania"[mesh] OR "Andorra"[mesh] OR "Armenia"[mesh] OR "Armenia"[mesh] OR "Austria"[mesh] OR "Azerbaijan"[mesh] OR "Republic of Belarus"[mesh] OR "Belgium"[mesh] OR "Bosnia and Herzegovina"[mesh] OR "Bulgaria"[mesh] OR "Croatia"[mesh] OR "Czech Republic"[mesh] OR "Denmark"[mesh] OR "England"[mesh] OR "Estonia"[mesh] OR "Finland"[mesh] OR "France"[mesh] OR "Georgia"[mesh] OR "Germany"[mesh] OR "Gibraltar"[mesh] OR "Greece"[mesh] OR "Hungary"[mesh] OR "Iceland"[mesh] OR "Ireland"[mesh] OR "Italy"[mesh] OR "Kazakhstan"[mesh] OR "Kosovo"[mesh] OR "Kyrgyzstan"[mesh] OR "Latvia"[mesh] OR "Liechtenstein"[mesh] OR "Lithuania"[mesh] OR "Luxembourg"[mesh] OR "Malta"[mesh] OR "Moldova"[mesh] OR "Monaco"[mesh] OR "Montenegro"[mesh] OR "Netherlands"[mesh] OR "Republic of North Macedonia"[mesh] OR "Northern Ireland"[mesh] OR "Norway"[mesh] OR "Poland"[mesh] OR "Portugal"[mesh] OR "Romania"[mesh] OR "Russia"[mesh] OR "San

Marino"[mesh] OR "Scotland"[mesh] OR "Serbia"[mesh] OR "Slovakia"[mesh] OR "Slovenia"[mesh] OR "Spain"[mesh] OR "Sweden"[mesh] OR "Switzerland"[mesh] OR "Turkey"[mesh] OR "Ukraine"[mesh] OR "United Kingdom"[mesh] OR "Uzbekistan"[mesh] OR "Vatican City"[mesh] OR "Wales"[mesh] OR "Europe"[tw] OR "European"[tw] OR "Albania"[tw] OR "Andorra"[tw] OR "Armenia"[tw] OR "Armenia"[tw] OR "Austria"[tw] OR "Azerbaijan"[tw] OR "Belarus"[tw] OR "Belgium"[tw] OR "Bosnia"[tw] OR "Bulgaria"[tw] OR "Croatia"[tw] OR "Czech Republic"[tw] OR "Denmark"[tw] OR "England"[tw] OR "Estonia"[tw] OR "Finland"[tw] OR "France"[tw] OR "Georgia"[tw] OR "Germany"[tw] OR "Gibraltar"[tw] OR "Greece"[tw] OR "Herzegovina"[tw] OR "Hungary"[tw] OR "Iceland"[tw] OR "Ireland"[tw] OR "Italy"[tw] OR "Kazakhstan"[tw] OR "Kosovo"[tw] OR "Kyrgyzstan"[tw] OR "Latvia"[tw] OR "Liechtenstein"[tw] OR "Lithuania"[tw] OR "Luxemfcrdbourg"[tw] OR "Malta"[tw] OR "Moldova"[tw] OR "Monaco"[tw] OR "Montenegro"[tw] OR "Netherlands"[tw] OR "North Macedonia"[tw] OR "Northern Ireland"[tw] OR "Norway"[tw] OR "Poland"[tw] OR "Portugal"[tw] OR "Romania"[tw] OR "Russia"[tw] OR "San Marino"[tw] OR "Scotland"[tw] OR "Serbia"[tw] OR "Slovakia"[tw] OR "Slovenia"[tw] OR "Spain"[tw] OR "Sweden"[tw] OR "Switzerland"[tw] OR "Turkey"[tw] OR "Ukraine"[tw] OR "United Kingdom"[tw] OR "Uzbekistan"[tw] OR "Vatican City"[tw] OR "Wales"[tw] OR "Albanian"[tw] OR "Armenian"[tw] OR "Austrian"[tw] OR "Belgian"[tw] OR "Bosnian"[tw] OR "Bulgarian"[tw] OR "Croatian"[tw] OR "Czech"[tw] OR "Danish"[tw] OR "British"[tw] OR "Estonian"[tw] OR "Finnish"[tw] OR "French"[tw] OR "Georgian"[tw] OR "German"[tw] OR "Greek"[tw] OR "Hungarian"[tw] OR "Icelandic"[tw] OR "Irish"[tw] OR "Italian"[tw] OR "Kosovan"[tw] OR "Latvian"[tw] OR "Lithuanian"[tw] OR "Moldovan"[tw] OR "Dutch"[tw] OR "Macedonian"[tw] OR "Norwegian"[tw] OR "Polish"[tw] OR "Romanian"[tw] OR "Russian"[tw] OR "Scottish"[tw] OR "Serbian"[tw] OR "Slovakian"[tw] OR "Slovenian"[tw] OR "Spanish"[tw] OR "Swedish"[tw] OR "Swiss"[tw] OR "Turkish"[tw] OR "Ukrainian"[tw] OR "Welsh"[tw]))) AND ("2013/01/01"[PDAT] : "3000/12/31"[PDAT])

Web of Science – Orthopaedic registries

((ti=("Hip Replacement" OR "Hip Prosthesis" OR "hip replacement" OR "hip replacement*" OR "hip arthroplasty" OR "hip arthroplast*" OR "hip prosthesis" OR "hip prosthe*" OR "THA" OR "THR" OR "hip implant" OR "hip implants" OR "Knee Replacement" OR "Knee Prosthesis" OR "knee replacement" OR "knee replacement*" OR "knee arthroplasty" OR "knee arthroplast*" OR "knee prosthesis" OR "knee prosthe*" OR "TKA" OR "TKR" OR "knee implant" OR "knee implants" OR "Shoulder Replacement" OR "Shoulder Prosthesis" OR "shoulder replacement" OR "shoulder replacement*" OR "shoulder arthroplasty" OR "shoulder arthroplast*" OR "shoulder prosthesis" OR "shoulder prosthe*" OR "shoulder

implant" OR "knee implants" OR "Ankle Replacement" OR "Ankle Prosthesis" OR "ankle replacement" OR "ankle replacement*" OR "ankle arthroplasty" OR "ankle arthroplast*" OR "ankle prosthesis" OR "ankle prosthe*" OR "ankle implant" OR "ankle implants" OR (("Hip" OR "hip" OR "hips" OR "Knee" OR "knee" OR "knees" OR "Shoulder" OR "Shoulder" OR "Shoulders" OR "Ankle" OR "ankle" OR "ankles") AND ("Prosthesis" OR "Prostheses" OR "Prosthesis" OR "Implants" OR "Implant" OR "replacement" OR "replacements" OR "arthroplasty" OR "arthroplast*"))) AND (ts=("Register" OR "register" OR "registers" OR "registry" OR "registries") OR ad=("register" OR "registers" OR "registry" OR "registries")) AND TS=("European Union" OR "European Union" OR "European Community" OR "European Coal and Steel Community" OR "Common Market" OR "EEC" OR "European Economic Community" OR "European Common Market" OR "European Economic Area" OR "Europe" OR "Albania" OR "Andorra" OR "Armenia" OR "Armenia" OR "Austria" OR "Azerbaijan" OR "Republic of Belarus" OR "Belgium" OR "Bosnia and Herzegovina" OR "Bulgaria" OR "Croatia" OR "Czech Republic" OR "Denmark" OR "England" OR "Estonia" OR "Finland" OR "France" OR "Georgia" OR "Germany" OR "Gibraltar" OR "Greece" OR "Hungary" OR "Iceland" OR "Ireland" OR "Italy" OR "Kazakhstan" OR "Kosovo" OR "Kyrgyzstan" OR "Latvia" OR "Liechtenstein" OR "Lithuania" OR "Luxembourg" OR "Malta" OR "Moldova" OR "Monaco" OR "Montenegro" OR "Netherlands" OR "Republic of North Macedonia" OR "Northern Ireland" OR "Norway" OR "Poland" OR "Portugal" OR "Romania" OR "Russia" OR "San Marino" OR "Scotland" OR "Serbia" OR "Slovakia" OR "Slovenia" OR "Spain" OR "Sweden" OR "Switzerland" OR "Turkey" OR "Ukraine" OR "United Kingdom" OR "Uzbekistan" OR "Vatican City" OR "Wales" OR "Albanian" OR "Armenian" OR "Austrian" OR "Belgian" OR "Bosnian" OR "Bulgarian" OR "Croatian" OR "Czech" OR "Danish" OR "British" OR "Estonian" OR "Finnish" OR "French" OR "Georgian" OR "German" OR "Greek" OR "Hungarian" OR "Icelandic" OR "Irish" OR "Italian" OR "Kosovan" OR "Latvian" OR "Lithuanian" OR "Moldovan" OR "Dutch" OR "Macedonian" OR "Norwegian" OR "Polish" OR "Romanian" OR "Russian" OR "Scottish" OR "Serbian" OR "Slovakian" OR "Slovenian" OR "Spanish" OR "Swedish" OR "Swiss" OR "Turkish" OR "Ukrainian" OR "Welsh")) **OR** (ts=("Hip Replacement" OR "Hip Prosthesis" OR "hip replacement" OR "hip replacement*" OR "hip arthroplasty" OR "hip arthroplast*" OR "hip prosthesis" OR "hip prosthe*" OR "THA" OR "THR" OR "hip implant" OR "hip implants" OR "Knee Replacement" OR "Knee Prosthesis" OR "knee replacement" OR "knee replacement*" OR "knee arthroplasty" OR "knee arthroplast*" OR "knee prosthesis" OR "knee prosthe*" OR "TKA" OR "TKR" OR "knee implant" OR "knee implants" OR "Shoulder Replacement" OR "Shoulder Prosthesis" OR "shoulder replacement" OR "shoulder replacement*" OR "shoulder arthroplasty" OR "shoulder arthroplast*" OR "shoulder prosthesis" OR "shoulder prosthe*" OR "shoulder implant" OR "knee implants" OR "Ankle Replacement" OR "Ankle Prosthesis" OR "ankle replacement" OR "ankle replacement*" OR "ankle

arthroplasty" OR "ankle arthroplast*" OR "ankle prosthesis" OR "ankle prosthe*" OR "ankle implant" OR "ankle implants" OR (("Hip" OR "hip" OR "hips" OR "Knee" OR "knee" OR "knees" OR "Shoulder" OR "Shoulder" OR "Shoulders" OR "Ankle" OR "ankle" OR "ankles") AND ("Prosthesis" OR "Prostheses" OR "Prosthesis" OR "Implants" OR "Implant" OR "replacement" OR "replacements" OR "arthroplasty" OR "arthroplast*"))) AND ti=("Register" OR "register" OR "registers" OR "registry" OR "registries" OR "register" OR "registers" OR "registry" OR "registries") AND ts=("European Union" OR "European Union" OR "European Community" OR "European Coal and Steel Community" OR "Common Market" OR "EEC" OR "European Economic Community" OR "European Common Market" OR "European Economic Area" OR "Europe" OR "Albania" OR "Andorra" OR "Armenia" OR "Armenia" OR "Austria" OR "Azerbaijan" OR "Republic of Belarus" OR "Belgium" OR "Bosnia and Herzegovina" OR "Bulgaria" OR "Croatia" OR "Czech Republic" OR "Denmark" OR "England" OR "Estonia" OR "Finland" OR "France" OR "Georgia" OR "Germany" OR "Gibraltar" OR "Greece" OR "Hungary" OR "Iceland" OR "Ireland" OR "Italy" OR "Kazakhstan" OR "Kosovo" OR "Kyrgyzstan" OR "Latvia" OR "Liechtenstein" OR "Lithuania" OR "Luxembourg" OR "Malta" OR "Moldova" OR "Monaco" OR "Montenegro" OR "Netherlands" OR "Republic of North Macedonia" OR "Northern Ireland" OR "Norway" OR "Poland" OR "Portugal" OR "Romania" OR "Russia" OR "San Marino" OR "Scotland" OR "Serbia" OR "Slovakia" OR "Slovenia" OR "Spain" OR "Sweden" OR "Switzerland" OR "Turkey" OR "Ukraine" OR "United Kingdom" OR "Uzbekistan" OR "Vatican City" OR "Wales" OR "Albanian" OR "Armenian" OR "Austrian" OR "Belgian" OR "Bosnian" OR "Bulgarian" OR "Croatian" OR "Czech" OR "Danish" OR "British" OR "Estonian" OR "Finnish" OR "French" OR "Georgian" OR "German" OR "Greek" OR "Hungarian" OR "Icelandic" OR "Irish" OR "Italian" OR "Kosovan" OR "Latvian" OR "Lithuanian" OR "Moldovan" OR "Dutch" OR "Macedonian" OR "Norwegian" OR "Polish" OR "Romanian" OR "Russian" OR "Scottish" OR "Serbian" OR "Slovakian" OR "Slovenian" OR "Spanish" OR "Swedish" OR "Swiss" OR "Turkish" OR "Ukrainian" OR "Welsh"))**)** AND py=(2013 OR 2014 OR 2015 OR 2016 OR 2017 OR 2018 OR 2019 OR 2020 OR 2021 OR 2022) NOT dt=(meeting abstract)

Web of Science – Cardiovascular registries

((ti=("cardiac implantable electronic device" OR "artificial heart pacemaker" OR "pacemaker" OR "pacemakers" OR "Artificial Heart" OR "artificial heart" OR "artificial hearts" OR "Heart Assist Device" OR "Artificial Heart" OR "Artificial Ventricle" OR "Artificial Ventricles" OR "Heart Assist Device" OR "Heart Assist Devices" OR "Heart Assist Pump" OR "Heart Assist Pumps" OR "Vascular Assist Device" OR "Vascular Assist Devices" OR "Ventricle Assist Device" OR "Ventricle Assist Devices" OR

"Ventricular Assist Device" OR "Ventricular Assist Devices" OR "Heart Valve Prosthesis" OR "Heart Valve Prosthesis" OR "Heart Valve Prosthesis" OR "Cardiac Valve Prosthesis" OR "Cardiac Valve Prostheses" OR "Heart Prosthesis" OR "Heart Prosthesis" OR "Cardiac Prosthesis" OR "Cardiac Prostheses" OR "artificial heart valves" OR "artificial heart valve" OR "artificial valves" OR "artificial valves" OR "Implantable Defibrillator" OR "Implantable Defibrillator" OR "Implantable Defibrillators" OR "Implantable Cardioverter Defibrillator" OR "Implantable Cardioverter Defibrillators" OR "bioresorbable vascular stent" OR "bioresorbable vascular scaffold" OR "bioresorbable vascular scaffolds" OR "transcatheter aortic valve implantation" OR "transcatheter aortic valve implantation" OR "transcatheter aortic valve implant" OR "transcatheter aortic valve implants" OR "TAVI" OR "transseptal mitral valve-in-ring" OR "TMVR" OR "LAAOC" OR (("Heart" OR "heart" OR "cardiac") AND ("Prosthesis" OR "Prostheses" OR "Prosthesis" OR "Implants" OR "Implant" OR "replacement" OR "replacements"))) AND (ts=("Register" OR "register" OR "registers" OR "registry" OR "registries") OR ad=("register" OR "registers" OR "registry" OR "registries")) AND TS=("European Union" OR "European Union" OR "European Community" OR "European Coal and Steel Community" OR "Common Market" OR "EEC" OR "European Economic Community" OR "European Common Market" OR "European Economic Area" OR "Europe" OR "Albania" OR "Andorra" OR "Armenia" OR "Armenia" OR "Austria" OR "Azerbaijan" OR "Republic of Belarus" OR "Belgium" OR "Bosnia and Herzegovina" OR "Bulgaria" OR "Croatia" OR "Czech Republic" OR "Denmark" OR "England" OR "Estonia" OR "Finland" OR "France" OR "Georgia" OR "Germany" OR "Gibraltar" OR "Greece" OR "Hungary" OR "Iceland" OR "Ireland" OR "Italy" OR "Kazakhstan" OR "Kosovo" OR "Kyrgyzstan" OR "Latvia" OR "Liechtenstein" OR "Lithuania" OR "Luxembourg" OR "Malta" OR "Moldova" OR "Monaco" OR "Montenegro" OR "Netherlands" OR "Republic of North Macedonia" OR "Northern Ireland" OR "Norway" OR "Poland" OR "Portugal" OR "Romania" OR "Russia" OR "San Marino" OR "Scotland" OR "Serbia" OR "Slovakia" OR "Slovenia" OR "Spain" OR "Sweden" OR "Switzerland" OR "Turkey" OR "Ukraine" OR "United Kingdom" OR "Uzbekistan" OR "Vatican City" OR "Wales" OR "Albanian" OR "Armenian" OR "Austrian" OR "Belgian" OR "Bosnian" OR "Bulgarian" OR "Croatian" OR "Czech" OR "Danish" OR "British" OR "Estonian" OR "Finnish" OR "French" OR "Georgian" OR "German" OR "Greek" OR "Hungarian" OR "Icelandic" OR "Irish" OR "Italian" OR "Kosovan" OR "Latvian" OR "Lithuanian" OR "Moldovan" OR "Dutch" OR "Macedonian" OR "Norwegian" OR "Polish" OR "Romanian" OR "Russian" OR "Scottish" OR "Serbian" OR "Slovakian" OR "Slovenian" OR "Spanish" OR "Swedish" OR "Swiss" OR "Turkish" OR "Ukrainian" OR "Welsh")) **OR** (ts=("cardiac implantable electronic device" OR "artificial heart pacemaker" OR "pacemaker" OR "pacemakers" OR "Artificial Heart" OR "artificial heart" OR "artificial hearts" OR "Heart Assist Device" OR "Artificial Heart" OR "Artificial Ventricle" OR "Artificial Ventricles" OR "Heart Assist Device" OR "Heart Assist

Devices" OR "Heart Assist Pump" OR "Heart Assist Pumps" OR "Vascular Assist Device" OR "Vascular Assist Devices" OR "Ventricle Assist Device" OR "Ventricle Assist Devices" OR "Ventricular Assist Device" OR "Ventricular Assist Devices" OR "Heart Valve Prosthesis" OR "Heart Valve Prosthesis" OR "Heart Valve Prosthesis" OR "Cardiac Valve Prosthesis" OR "Cardiac Valve Prostheses" OR "Heart Prosthesis" OR "Heart Prosthesis" OR "Cardiac Prosthesis" OR "Cardiac Prostheses" OR "artificial heart valves" OR "artificial heart valve" OR "artificial valves" OR "artificial valves" OR "Implantable Defibrillator" OR "Implantable Defibrillator" OR "Implantable Defibrillators" OR "Implantable Cardioverter Defibrillator" OR "Implantable Cardioverter Defibrillators" OR "bioresorbable vascular stent" OR "bioresorbable vascular scaffold" OR "bioresorbable vascular scaffolds" OR "transcatheter aortic valve implantation" OR "transcatheter aortic valve implantation" OR "transcatheter aortic valve implant" OR "transcatheter aortic valve implants" OR "TAVI" OR "transseptal mitral valve-in-ring" OR "TMVR" OR "LAAOC" OR (("Heart" OR "heart" OR "cardiac") NEAR/4 ("Prosthesis" OR "Prostheses" OR "Prosthesis" OR "Implants" OR "Implant" OR "replacement" OR "replacements"))) AND ti=("Register" OR "register" OR "registers" OR "registry" OR "registries" OR "register" OR "registers" OR "registry" OR "registries") AND ts=("European Union" OR "European Union" OR "European Community" OR "European Coal and Steel Community" OR "Common Market" OR "EEC" OR "European Economic Community" OR "European Common Market" OR "European Economic Area" OR "Europe" OR "Albania" OR "Andorra" OR "Armenia" OR "Armenia" OR "Austria" OR "Azerbaijan" OR "Republic of Belarus" OR "Belgium" OR "Bosnia and Herzegovina" OR "Bulgaria" OR "Croatia" OR "Czech Republic" OR "Denmark" OR "England" OR "Estonia" OR "Finland" OR "France" OR "Georgia" OR "Germany" OR "Gibraltar" OR "Greece" OR "Hungary" OR "Iceland" OR "Ireland" OR "Italy" OR "Kazakhstan" OR "Kosovo" OR "Kyrgyzstan" OR "Latvia" OR "Liechtenstein" OR "Lithuania" OR "Luxembourg" OR "Malta" OR "Moldova" OR "Monaco" OR "Montenegro" OR "Netherlands" OR "Republic of North Macedonia" OR "Northern Ireland" OR "Norway" OR "Poland" OR "Portugal" OR "Romania" OR "Russia" OR "San Marino" OR "Scotland" OR "Serbia" OR "Slovakia" OR "Slovenia" OR "Spain" OR "Sweden" OR "Switzerland" OR "Turkey" OR "Ukraine" OR "United Kingdom" OR "Uzbekistan" OR "Vatican City" OR "Wales" OR "Albanian" OR "Armenian" OR "Austrian" OR "Belgian" OR "Bosnian" OR "Bulgarian" OR "Croatian" OR "Czech" OR "Danish" OR "British" OR "Estonian" OR "Finnish" OR "French" OR "Georgian" OR "German" OR "Greek" OR "Hungarian" OR "Icelandic" OR "Irish" OR "Italian" OR "Kosovan" OR "Latvian" OR "Lithuanian" OR "Moldovan" OR "Dutch" OR "Macedonian" OR "Norwegian" OR "Polish" OR "Romanian" OR "Russian" OR "Scottish" OR "Serbian" OR "Slovakian" OR "Slovenian" OR "Spanish" OR "Swedish" OR "Swiss" OR "Turkish" OR "Ukrainian" OR "Welsh"))) AND py=(2013 OR 2014 OR 2015 OR 2016 OR 2017 OR 2018 OR 2019 OR 2020 OR 2021 OR 2022) NOT dt=(meeting abstract)

Google Scholar – Orthopaedic registries

allintitle: "Hip"|"Knee"|"Shoulder"|"Ankle" "Prosthesis"|"Implant"|"replacement"|"arthroplasty" "Register"|"registers"|"registry"|"registries" -"american" -"australian" -"canadian"

Google Scholar – Cardiovascular Registries

allintitle: "Heart"|"cardiac" "Prosthesis"|"Implant"|"replacement" "Register"|"registers"|"registry"|"registries" -"american" -"australian" -"canadian"

allintitle: "cardiac implant"|"pacemaker"|"Artificial Heart"|"Implantable Defibrillator" "Register"|"registers"|"registry"|"registries" -"american" -"australian" -"canadian"

allintitle: "TAVI"|"TMVR"|"LAAOC" "Register"|"registers"|"registry"|"registries" -"american" -"australian" -"canadian"

Publication date limit: (2013 OR 2014 OR 2015 OR 2016 OR 2017 OR 2018 OR 2019 OR 2020 OR 2021 OR 2022)
