# Supplementary material for: Quality and Utility of European Cardiovascular and Orthopaedic Registries for the Regulatory Evaluation of Medical Device Safety and Performance Across the Implant Lifecycle: A Systematic Review
Source: Int J Health Policy Manag. 2023 Jul 18;12:7648. doi: 10.34172/ijhpm.2023.7648 (PMC10702370; doi:10.34172/ijhpm.2023.7648)
Supplement: Supplementary file 2 — Tables S1A-S8A. [file ijhpm-12-7648-s002.pdf]

Article title: Quality and Utility of European Cardiovascular and Orthopaedic Registries for the Regulatory Evaluation of Medical Device Safety and Performance Across the Implant Lifecycle: A Systematic Review

Journal name: International Journal of Health Policy and Management (IJHPM)

Authors' information: Lotje A. Hoogervorst[1,2]*, Timon H. Geurkink[1], Anne Lübbeke[3,4], Sergio Buccheri[5], Jan W. Schoones[6], Marina Torre[7], Paola Laricchiuta[7], Paul Piscoi[8], Alma B. Pedersen[9,10], Chris P. Gale[11,12,13], James A. Smith[14,15], Aldo P. Maggioni[16], Stefan James[5,17,18], Alan G. Fraser[19], Rob G.H.H. Nelissen[1], Perla J. Marang-van de Mheen[2]

*Correspondence to: Lotje A. Hoogervorst, Email: l.a.hoogervorst@lumc.nl

Citation: Hoogervorst LA, Geurkink TH, Lübbeke A, et al. Quality and utility of European cardiovascular and orthopaedic registries for the regulatory evaluation of medical device safety and performance across the implant lifecycle: a systematic review. Int J Health Policy Manag. 2023;12:7648. doi:10.34172/ijhpm.2023.7648

Supplementary file 2

Table S1A: Cardiovascular registries – Domain 'Identification'				
	**Country**	**Design**	**Website**	**Initial motivation / goal**
**Cardiovascular registries – combined**				
British Cardiovascular Intervention Society	The UK	National	https://www.bcis.org.uk/	To promote education, training and research in cardiovascular intervention and develops and upholds clinical and professional standards[1]
East Denmark Heart Registry	Denmark	National	N/R	N/R
German Society for Thoracic and Cardiovascular Surgery	Germany	National	https://www.dgthg.de/	To promote the science and further development of therapies in the field of thoracic, cardiac, and vascular surgery[465]
Polish National Database of Cardiac Surgery Procedures	Poland	National	https://krok.csioz.gov.pl/krok/	N/R
Portuguese National Registry of Intervention Cardiology	Portugal	National	https://www.apic.pt/	To study, investigate and promote other scientific activities within the scope of medical, surgical, technological, and organizational aspects of cardiovascular intervention[466]
Spanish Cardiac Catheterization and Coronary Intervention Registry	Spain	National	N/R	To report the activity recorded in interventional cardiology laboratories in Spain[7]
Western Denmark Heart Registry	Denmark	Regional	N/R	To promote clinical and health services research on use of cardiovascular procedures and their outcomes[8]
**Cardiovascular registries – stents**				
Polish National Percutaneous Coronary Intervention Registry	Poland	National	https://www.orpki.cm-uj.krakow.pl/	N/R
Swedish Coronary Angiography and Angioplasty Registry	Sweden	National	https://www.ucr.uu.se/swedeheart/start-scaar/	To collect relevant information regarding disease severity, medical and medical-technical treatment from the time of intervention on all performed coronary angiograms and PCI treatments[9]
**Cardiovascular registries – valves**				
Quality Assurance Registry on Aortic Valve Replacement	Germany	National	N/R	N/R
Austrian-TAVI Registry	Austria	National	https://www.tavi.at/	N/R
Belgian TAVI Registry	Belgium	National	N/R	N/R
Czech TAVI Registry	Czechia	National	N/R	To investigate 1) the clinical impact of the relative high rate of paravalvular leaks, and 2) the function of implanted valves in real long-term follow-up exceeding 5 years[194]
FinnValve Registry	Finland	National	N/R	N/R
FRANCE-TAVI Registry	France	National	N/R	To identify all patients with a change of valves implanted catheter meets the selection criteria of the technical accepting the scheduled evaluations in the context of this disease and who have agreed to participate in the study[477]
German Aortic Valve Registry	Germany	National	https://www.aortenklappenregister.de/	1) to present the structure, process, and result quality of the various techniques of aortic valve therapy; 2) to determine

				criteria for indications (e.g. through scoring systems); 3) to record quality and safety of specific medical devices; 4) to assess the quality of care of participating centres with the aim to improve healthcare quality, and 5) to evaluate health economical statuses of treatments[478]
Polish Registry of Transcatheter Aortic Valve Implantation	Poland	National	N/R	1) to monitor TAVI indications and procedural strategy; 2) to supervise adherence to the guidelines and compliance with indications/contraindications to TAVI; 3) to assess objectively and non-commercially peri-procedural device success; 4) to monitor and improve safety, quality and efficacy of treatment; 5) to assess early- and long-term results of this novel method of treatment in order to develop the most optimal therapeutic follow-up strategy, and 6) to monitor and evaluate cost-effectiveness of TAVI in Poland[16]
Spanish Registry of Heart Valves Repair	Spain	National	N/R	N/R
Swedish Transcatheter Cardiac Intervention Registry	Sweden	National	https://www.ucr.uu.se/swedeheart/start-swentry/	To evaluate the new method for aortic valve intervention both with regard to acute results and long-term follow-up[479]
Swiss TAVI Registry	Switzerland	National	https://www.swisstavi.ch/	To assess the clinical outcomes of consecutive patients undergoing TAVI in Switzerland[252]

Table S2A: Cardiovascular registries – Domain 'Maturity'			
	**Starting year**	**First annual report (publishing year)**	**Most recent/last annual report (publishing year)**
**Cardiovascular registries – combined**			
British Cardiovascular Intervention Society	1988[1]	1991[1]	2020, data till 2020[1]
East Denmark Heart Registry	2005[2]	N/R	N/R
German Society for Thoracic and Cardiovascular Surgery	1978[3]	1989[3]	2021, data till 2020[4]
Polish National Database of Cardiac Surgery Procedures	2006[5]	N/R	N/R
Portuguese National Registry of Intervention Cardiology	2002[6]	N/R	N/R
Spanish Cardiac Catheterization and Coronary Intervention Registry	1990[7]	1990[7]	2020, data till 2019[7]
Western Denmark Heart Registry	1999[8]	N/R	N/R
**Cardiovascular registries – stents**			
Polish National Percutaneous Coronary Intervention Registry	N/R	N/R	N/R
Swedish Coronary Angiography and Angioplasty Registry	1998[9]	2007[10]	2021, data till 2020[9, 10]
**Cardiovascular registries – valves**			
Quality Assurance Registry on Aortic Valve Replacement	N/R	N/R	N/R
Austrian-TAVI Registry	2011[11]	N/R	N/R
Belgian TAVI Registry	2007[12]	N/R	N/R
Czech TAVI Registry	2009[13]	N/R	N/R
FinnValve Registry	N/R	N/R	N/R
FRANCE-TAVI Registry	2013[14]	N/R	N/R
German Aortic Valve Registry	2010[15]	N/R	N/R
Polish Registry of Transcatheter Aortic Valve Implantation	2013[16]	N/R	N/R
Spanish Registry of Heart Valves Repair	N/R	2013[17]	2019, data till 2017[18]
Swedish Transcatheter Cardiac Intervention Registry	2010[19]	2010[10]	2021, data till 2020[10]
Swiss TAVI Registry	2011[20]	N/R	N/R

Table S3A: Cardiovascular registries – Domain ‘Governance’					
	**Mandatory**	**Patients' consent**	**Funding**	**Who can access the data and see results?**	**Privacy regulation for patients identifiable information**
**Cardiovascular registries – combined**					
British Cardiovascular Intervention Society	N/R	N/R	Public[21]	N/R	N/R
East Denmark Heart Registry	Yes[22]	N/R	N/R	N/R	N/R
German Society for Thoracic and Cardiovascular Surgery	No[3]	N/R	N/R	N/R	N/R
Polish National Database of Cardiac Surgery Procedures	Yes[5]	Not required[5]	Public[5]	N/R	N/R
Portuguese National Registry of Intervention Cardiology	No[6]	Required[23]	N/R	N/R	N/R
Spanish Cardiac Catheterization and Coronary Intervention Registry	No[7]	N/R	N/R	N/R	N/R
Western Denmark Heart Registry	Yes[22]	Not required[24]	Public[25]	N/R	Serial numbers are generated when uploading data[8]
**Cardiovascular registries – stents**					
Polish National Percutaneous Coronary Intervention Registry	N/R	N/R	N/R	N/R	N/R
Swedish Coronary Angiography and Angioplasty Registry	Yes[26]	Not required[26]	Public[27]	N/R	Unique personal ID number[28]
**Cardiovascular registries – valves**					
Quality Assurance Registry on Aortic Valve Replacement	Yes[29]	N/R	N/R	N/R	N/R
Austrian-TAVI Registry	N/R	Required[11]	N/R	N/R	N/R
Belgian TAVI Registry	N/R	N/R	N/R	N/R	N/R
Czech TAVI Registry	N/R	Not required[13]	N/R	N/R	All data are anonymous[13]
FinnValve Registry	N/R	Not required[30]	N/R	N/R	N/R
FRANCE-TAVI Registry	No[14]	Required[14]	Private[14]	N/R	N/R
German Aortic Valve Registry	No[31]	Required[32]	Private[15]	N/R	All data are anonymous[33]
Polish Registry of Transcatheter Aortic Valve Implantation	Yes[16]	Required[16]	Public[34]	Limited to authorized representatives of TAVI-centers[16]	N/R
Spanish Registry of Heart Valves Repair	No[35]	N/R	N/R	N/R	N/R
Swedish Transcatheter Cardiac Intervention Registry	Yes[26]	Not required[26]	Public[27]	N/R	Unique personal ID number[36]
Swiss TAVI Registry	Yes[9]	Required[20]	Private[20]	N/R	N/R

Table S4A: Cardiovascular registries – Domain 'Coverage, design & organisation'								
	**No. of hospitals (% of coverage)**	**Number of patients/ procedures (total)**	**Number of patients/ procedures (selected)**	**Annual number of patients/ procedures (last year)**	**Data capture and collection**	**Access to registry for users/members**	**Type of information provided, for whom and at which level**	**Data linkage with other sources**
**Cardiovascular registries – combined**								
British Cardiovascular Intervention Society	Minimal 118 (exact number unknown) (N/R)[1]	28,622 TAVI procedures (2007-2020)[1]	N/A	6,076 TAVI procedures and 100,112 PCI procedures (2019)[1]	Web-based[1]	Through website[1]	Hospital- and surgeon-level[1]	National health service[37]
East Denmark Heart Registry	N/R (N/R)	N/R	3 studies (range: 944-50,460)[2, 38, 39]	N/R	Web-based[25]	Through website[25]	N/R	N/R
German Society for Thoracic and Cardiovascular Surgery	78 (N/R)[3]	N/R	N/A	35,469 valves (2020)[40]	Web-based[41]	N/R	N/R	N/R
Polish National Database of Cardiac Surgery Procedures	37 (100%)[5]	N/R	15 studies (range: 3,057-188,972)[5, 42-55]	N/R	Web-based[56]	N/R	N/R	National health fund (health insurance institution)[53]
Portuguese National Registry of Intervention Cardiology	25 (N/R)[57]	73,977 PCI (2010-2015)[58]	N/A	13,891 PCI (2015)[58]	Web-based[6]	N/R	N/R	N/R
Spanish Cardiac Catheterization and Coronary Intervention Registry	123 (97.6%)[59]	N/R	N/A	4,692 valves and 92,771 stents (2020)[59]	Web-based[60]	N/R	N/R	N/R
Western Denmark Heart Registry	13 (100%)[8]	N/R	74 studies (range: 68-1,200,472)[8, 24, 25, 61-131]	N/R	Web-based[8]	Through website[8]	Hospital- and medical device-level[8]	National patient register[130]
**Cardiovascular registries – stents**								
Polish National Percutaneous Coronary Intervention Registry	161 (28.2%)[132]	N/R	46 studies (range: 591 - 1,436,546)[133-178]	N/R	Web-based[166]	N/R	N/R	N/R

Swedish Coronary Angiography and Angioplasty Registry	29 (N/R)[179]	380,220 (2007-2020)[180]	N/A	24,657 PCI patients (2020)[180]	Web-based[9]	Through website[9]	Hospital- and medical device-level[10]	National patient register[28]
**Cardiovascular registries – valves**								
Quality Assurance Registry on Aortic Valve Replacement	95 (N/R)[181]	N/R	4 studies (range: 6,972-120,280)[29, 182-184]	N/R	Web-based[29]	N/R	N/R	N/R
Austrian-TAVI Registry	11 (100%)[11]	N/R	2 studies (range: 959-1,822)[11, 185]	N/R	Web-based[185]	Through website[185]	N/R	N/R
Belgian TAVI Registry	23 (N/R)[12]	N/R	3 studies (range: 328-861)[12, 186, 187]	N/R	Web-based[188]	N/R	N/R	N/R
Czech TAVI Registry	N/R	N/R	6 studies (range: 58-1,532)[13, 189-194]	N/R	Web-based[194]	N/R	N/R	N/R
FinnValve Registry	5 (100%)[195, 196]	6,463 (2008-2017)[30]	N/R	N/R	Web-based[197]	N/R	N/R	National Statistical Institution[195], Finnish Population Register Centre[197], Finnish National Institute for Health and Welfare[197]
FRANCE-TAVI Registry	50 (N/R)[198]	N/R	27 studies (range: 287-30,913)[14, 198-223]	N/R	Web-based[14]	N/R	N/R	N/R
German Aortic Valve Registry	92 (N/R)[15]	N/R	23 studies (range: 1,118-142,435)[15, 31-33, 224-242]	N/R	Web-based[33]	Through website[33]	Hospital- and medical device-level[33]	German external quality assurance plan[33]

Polish Registry of Transcatheter Aortic Valve Implantation	21 (N/R)[16]	N/R	11 studies (range: 19-5,043)[16, 34, 243-251]	N/R	Web-based[16]	N/R	N/R	Civil registries[248]
Spanish Registry of Heart Valves Repair	27 (N/R)[18]	N/R	N/R	1,607 mitral valve, 4,289 aortic valve, and 98 tricuspid valve replacements (2017)[18]	Web-based[18]	N/R	N/R	N/R
Swedish Transcatheter Cardiac Intervention Registry	8 (N/R)[10]	N/R	N/A	1,299 TAVI procedures (2020)[10]	Web-based[19]	Through website[19]	Hospital- and medical device-level[10]	National patient register[36]
Swiss TAVI Registry	16 (N/R)[252]	N/R	40 studies (range: 113-9,478)[20, 253-291]	N/R	Web-based[282]	Through website[282]	N/R	N/R

Table S5A: Cardiovascular registries – Manufacturers mentioned in annual reports, peer-reviewed publications & websites

Stents	Valves
Abbott Laboratories[110, 292-307]	Abbott Laboratories[5, 15, 30, 38, 42, 59, 185, 197, 228, 231, 259, 260, 263, 266, 267, 278, 282, 285, 286, 288, 308]
B. Braun[298, 301, 303-305]	Baxter International[5]
Biosensors International[292, 299, 301, 302]	Biosensors International[59, 253]
Biotronik AG[292-294, 299, 301, 302]	Boston Scientific[15, 38, 59, 185, 228, 250, 253, 258-260, 262, 263, 265-267, 274, 278, 281, 282, 285-288, 290, 308-311]
Boston Scientific[104, 110, 117, 119, 292-299, 301-304, 306, 307, 312, 313]	CryoLife[5, 42]
CID S.p.A[298, 304]	Edwards Lifesciences[5, 11, 12, 14, 15, 24, 30-32, 34, 38, 42, 59, 185, 186, 189, 191, 197, 199-202, 204-212, 214, 216-220, 223, 228-231, 233, 237, 243, 245, 246, 251, 253-255, 258-267, 271, 272, 274, 278, 282, 285-290, 308-311]

Cordis[71, 104, 110, 117, 119, 295-298, 301, 303-307, 312, 313]	JenaValve[15, 32, 228, 253, 282, 285, 286]
LivaNova[303, 305]	Labcor[5, 42, 228]
Medtronic[71, 110, 292-299, 301-307, 312]	LivaNova[5, 15, 30, 31, 42, 228, 231]
Terumo Corporation[292-294, 299, 301, 302]	Medtronic[2, 5, 11, 12, 14, 15, 20, 30-32, 34, 38, 42, 59, 185, 186, 189, 199-202, 204-212, 214, 216-220, 223, 228-231, 233, 237, 245, 246, 251, 253-255, 258-260, 262-267, 271, 272, 274, 278, 282, 285-290, 308-311]
	Meril Life[59]

Table S6A: Cardiovascular registries – Domain 'Data quality & completeness'

	Quality assurance system defined/quality check of data	Missing data for patients' characteristics	Methods for handling missing data	Data completeness on patient/ procedure-level
**Cardiovascular registries – combined**				
British Cardiovascular Intervention Society	Data platform has error checking for range and consistency and a validation cycle provides every operator to read their report so that corrections can be made prior to data publication[37]	N/R	In case of a completeness of <95% of a specific variable; the risk adjusted outcomes are considered to be inadequate[314]	N/R
East Denmark Heart Registry	N/R	N/R	N/R	N/R
German Society for Thoracic and Cardiovascular Surgery	After entering the data, it will be checked for completeness[3]	N/R	N/R	N/R
Polish National Database of Cardiac Surgery Procedures	N/R	N/R	N/R	N/R
Portuguese National Registry of Intervention Cardiology	N/R	N/R	N/R	N/R
Spanish Cardiac Catheterization and Coronary Intervention Registry	The steering committee and the working group perform data cleaning[60]	N/R	N/R	N/R
Western Denmark Heart Registry	Systematic validation procedures and random spot checks after data entry and variable levels are restricted within pre-specified limits[8]	N/R	N/R	N/R
**Cardiovascular registries – stents**				

Polish National Percutaneous Coronary Intervention Registry	N/R	N/R	N/R	N/R
Swedish Coronary Angiography and Angioplasty Registry	Data platform has error checking for range and consistency, definitions are displayed on screen when data is entered, data entered in the registry of 20 hospitals will be annually compared information in the patients' records from 30-<br>40 randomly chosen patients in each hospital and the majority of variables are mandatory[315]	N/R	N/R	N/R
**Cardiovascular registries – valves**				
Quality Assurance Registry on Aortic Valve Replacement	Controlled by validated system[29]	N/R	N/R	N/R
Austrian-TAVI Registry	N/R	N/R	N/R	N/R
Belgian TAVI Registry	N/R	N/R	N/R	N/R
Czech TAVI Registry	N/R	N/R	N/R	N/R
FinnValve Registry	Robust checking of completeness and data quality[310]	N/R	N/R	N/R
FRANCE-TAVI Registry	Regular data checks, data platform has error checking for range and consistency[14]	N/R	N/R	N/R
German Aortic Valve Registry	Data completeness is verified by an electronic tool which analyses reimbursement, data validity is monitored by a multistage plausibility check combined with an on-site data verification on a randomly selected 3% of the samples[33]	N/R	N/R	N/R
Polish Registry of Transcatheter Aortic Valve Implantation	Credibility and completeness of data will be verified through internal and external<br>regular audits[16]	N/R	N/R	N/R
Spanish Registry of Heart Valves Repair	N/R	N/R	N/R	N/R
Swedish Transcatheter Cardiac Intervention Registry	N/R	N/R	N/R	N/R
Swiss TAVI Registry	The Clinical Trials Unit of Bern performs data monitoring (e.g., completeness of data and plausibility checks)[286]	N/R	N/R	N/R

Table S7A: Cardiovascular registries – Outcomes reported, definition & duration of follow-up			
	**Mortality**	**MACE**	**Other**
**Cardiovascular registries – combined**			
British Cardiovascular Intervention Society	In-hospital[316-326]; 5-day[327]; 1-week[307]; 10-day[324, 327]; 15-day[327]; 20-day[324, 327]; 25-day[327]; 1-month[307, 318, 320, 323-336]; 100-day[324]; 6-month[307, 323, 330]; 200-day[324]; 10-month[324]; 1-year[307, 318, 322-325, 328, 330-332, 334, 337, 338]; 500-day[307]; 2-year[322, 337]; 1000-day[307]; 3-year[322, 323, 334, 337]; 4-year[322, 337]; 1500-day[307]; 5-year[322, 334]; 2000-day[307]; 6-year[319]; 2500-day[307].	In-hospital (including death, myocardial infarction, and re-intervention[320, 339]); In-hospital (including death, myocardial infarction, and revascularization[331]); In-hospital (including death, emergency coronary artery bypass grafting, myocardial infarction, and re-intervention[334]); In-hospital (unspecified[340])	Access site complication[331], aortic dissection[319, 323], arterial complication[326], bleeding[307, 317, 318, 321, 324-326, 331, 334, 339-341], blood transfusion[307, 318, 321, 330], cardiac complication[325], cardiogenic shock[317], cardioversion[319, 330], complication (unspecified)[325, 326], coronary dissection[307, 323], coronary complication[318, 322], coronary perforation[307, 323, 330], CVA[319, 321, 323, 326], ECMO usage[317], heart failure[317, 341], ICU stay (length)[317], in-hospital stay (length)[317, 321, 323, 339, 341], myocardial infarction[307, 316-319, 321-324, 326, 332, 341], no flow[307, 319, 323], pacemaker implantation[309], pericardial tamponade[323, 330], procedural success[318, 323, 337, 341], rehospitalization[341], re-intervention[318, 319, 322-324, 326, 330], renal failure[307, 323, 330], revascularization[307, 341], side branch occlusion[307, 319, 323, 330], slow flow[307, 319, 323], stent thrombosis[317, 341], stroke[307, 317, 322, 324, 325, 332, 341], vascular complication[322, 323, 325, 339]
East Denmark Heart Registry	1-month[2]; 1-year[2, 38]; 2-year[38]; 3-year[38]; 4-year[38]; 5-year[38, 39]; 6-year[38]; 7-year[38]; 8-year[38]; 9-year[38].	N/R	In-hospital stay (length)[2], myocardial infarction[39], revascularization[39], vascular complication[2]
German Society for Thoracic	In-hospital[3, 41, 342-379]	N/R	Complication (unspecified)[380], infection[381], re-intervention[378, 379]

and Cardiovascular Surgery			
Polish National Database of Cardiac Surgery Procedures	In-hospital[5, 42-44, 49, 50, 53];<br>Early (<24hr) post-operative[45, 48, 52, 53];<br>10-day[50];<br>20-day[50];<br>1-month[45, 48, 50, 52, 54];<br>1-year[43-45, 48, 49, 52-54];<br>2-year[43-45, 48-50, 52-54];<br>3-year[44, 45, 48, 49, 52-54];<br>4-year[43-45, 48-50, 52-54];<br>5-year[45, 48, 49, 52-54];<br>6-year[43, 45, 48-50, 52-54];<br>7-year[45, 48, 49, 52-54];<br>8-year[43, 45, 48-50, 52-54];<br>9-year[45, 48, 49, 52-54];<br>10-year[43, 45, 48-50, 52-55];<br>11-year[45, 48, 49, 52-54];<br>12-year[45, 48-50, 52, 53];<br>13-year[52].	N/R	Cardiogenic shock[53], ECMO usage[45, 48, 50, 52], gastro-intestinal complication[44, 45, 48-50, 52, 53], ICU readmission[49], ICU stay (length)[5, 42, 45, 48, 50, 52, 53], infection[45, 48-50, 52, 53], in-hospital stay (length)[5, 42], left ventricular support[43, 45, 49], multi-organ failure[45, 48, 50, 52, 53], myocardial infarction[43, 45, 49, 50, 52, 53], neurological complication[44, 45, 48-50, 52, 53], pacemaker implantation[48, 50, 52], pericardial tamponade[45, 49, 50, 52, 53], pulmonary embolism[45], re-intervention[43, 45, 49, 50, 52, 53], renal failure[43-45, 48-50, 52, 53], respiratory failure[44, 45, 49, 50, 52, 53]
Portuguese National Registry of Intervention Cardiology	N/R	N/R	N/R
Spanish Cardiac Catheterization and Coronary Intervention Registry	In-hospital[382];<br>6-month[383].	N/R	In-hospital stay (length)[382], mitral regurgitation[382], procedural success[382]
Western Denmark Heart Registry	In-hospital[24, 76, 82, 111, 123];<br>10-day[74];<br>20-day[74];<br>1-month[8, 24, 63, 67, 69, 70, 74, 76, 79, 82, 84, 86, 88, 95, 96, 101, 103, 110, 122, 126, 127, 130];<br>4-month[82];<br>6-month[67, 71, 86, 93, 122, 127];	2-year (including death, myocardial infarction, and revascularization[115]);<br>3-year (including cardiovascular-related death, myocardial infarction, revascularization, and stent thrombosis[100]);	Allergic reaction[8], arrhythmia[8, 79, 84, 127], atrial fibrillation[73, 107, 118, 129], bleeding[8, 25, 79, 86, 111, 121, 123, 128], blood transfusion[63, 102, 107, 118, 121], contrast-fluid reaction[79], EuroSCORE[124], infection[8, 25, 96, 102, 123], ICU stay (length)[8, 25, 67, 86, 96, 123, 125], in-hospital stay (length)[67, 79, 86, 96, 102, 109, 125], limb amputation[106], malignancy[108], myocardial infarction[8, 25, 61-63, 65, 67, 69, 71, 74, 77, 80, 81, 84, 86, 88-90, 92, 98, 100, 101, 105, 110, 117, 120, 122, 123, 128, 130], obstructive coronary artery disease[65], pericardial tamponade[86, 121], peripheral artery disease[106], rehospitalization[83, 102, 109], re-intervention[8, 24, 25, 61, 63, 67, 79, 86, 113, 121, 122], renal

	1-year[8, 24, 92, 93, 95-98, 101-103, 117, 126, 127]; 15-month[117]; 18-month[71, 101]; 2-year[62, 69, 71, 72, 76, 78, 93, 98, 102, 103, 126]; 30-month[24]; 3-year[72, 92, 93, 97, 102-104]; 4-year[72, 93, 103]; 5-year[24, 66, 72, 80, 93, 103, 127]; 6-year[93]; 7-year[93, 105]; 90-month[24]; 9-year[89]; 10-year[24, 90, 120, 127]; 11-year[77, 99]; 150-month[24, 128]; 15-year[24].	3-year (including death, myocardial infarction, revascularization, and stroke[102]); 5-year (including cardiovascular-related death, cardiac arrest, myocardial infarction, and revascularization[66]); 5-year (including cardiovascular-related death, myocardial infarction, and stroke[80]); 5-year (unspecified[85])	failure[25, 63, 67, 84, 86, 88, 96, 102, 116, 127, 128], revascularization[62, 69, 71, 75, 77, 87, 91, 92, 97, 98, 100-102, 119, 127], stent failure[97], stent restenosis[71, 91], stent thrombosis[62, 69, 71, 91, 92, 97, 98, 100, 101, 104, 111, 117, 126], stroke[8, 25, 61, 63, 67, 81, 84, 86, 88-90, 102, 112, 120, 122, 123, 127], TIA[8, 112]
**Cardiovascular registries – stents**			
Polish National Percutaneous Coronary Intervention Registry	Procedural[135, 137-140, 147, 149, 153, 154, 158, 159, 161, 162, 165-170, 174, 175]; In-hospital[141, 142, 150]; 5-day[150]; 10-day[150]; 15-day[150, 155]; 20-day[150]; 25-day[150]; 1-month[141, 150, 155, 157]; 45-day[155]; 2-month[155, 157]; 75-day[155]; 3-month[155, 157]; 4-month[157]; 5-month[157]; 6-month[142, 150, 155, 157]; 7-month[157]; 8-month[157]; 9-month[157]; 10-month[157]; 11-month[157]; 1-year[141, 142, 150, 155-157, 172, 176, 178];	Procedural (including death, myocardial infarction, and stroke[138, 161]); 1-year (including death, myocardial infarction, revascularization, and urgent PCI/CABG[156]); 3-year (unspecified[141, 150]); 3-year (including cardiac arrest, death, rehospitalization for heart failure, myocardial infarction, and stroke[142]); 3-year (including cardiac arrest, death, myocardial infarction, and stroke[155])	Allergic reaction[133-136, 138, 143, 144, 146-148, 151, 159, 161, 162, 165-167, 170, 175], angiographic findings[136, 144, 146-148, 153, 156, 161, 162, 168, 172, 178], bleeding[133-136, 138-140, 143, 144, 148, 149, 151, 161, 162, 167, 168, 170, 174, 175], cardiac arrest[140, 142-144, 148], complication (unspecified)[146, 175], coronary dissection[133, 134, 136, 138, 143, 144, 146, 147, 151, 154, 161, 166-168, 170, 175], coronary perforation[135, 136, 138-140, 143, 146, 148, 149, 154, 162, 164-166, 168, 170, 175], heart failure[142, 155], in-hospital stay (length)[142], myocardial infarction[138, 139, 142-144, 146, 151, 155, 156, 159, 161, 162, 165-168, 170, 172, 176, 178], no-reflow[133-135, 138-140, 143, 144, 146-148, 151, 153, 154, 159, 161, 162, 164-168, 170, 174, 175], rehospitalization[142, 155], re-intervention[142, 155, 156, 172, 176, 178], restenosis[144, 146, 162, 176], revascularization[156, 172, 178], stent thrombosis[146, 156, 162, 172, 176, 178], stroke[133, 134, 136, 138, 140, 142-144, 146, 151, 152, 154, 155, 159, 161, 162, 165-168, 170, 175], vessel perforation[147]

	13-month[157];<br>14-month[157];<br>15-month[157];<br>16-month[157];<br>17-month[157];<br>18-month[142, 150, 155, 157];<br>19-month[157];<br>20-month[157];<br>21-month[157];<br>22-month[157];<br>23-month[157];<br>2-year[142, 150, 155, 157];<br>30-month[142, 150, 155];<br>3-year[141, 142, 150, 155].		
Swedish Coronary Angiography and Angioplasty Registry	Procedural[384, 385];<br>In-hospital[385-391];<br>1-day[392, 393];<br>3-day[393];<br>5-day[391, 393, 394];<br>1-week[392, 393, 395];<br>10-day[315, 391, 396-400];<br>15-day[391];<br>20-day[315, 391, 394, 396-400];<br>25-day[391, 394];<br>1-month[302, 304, 315, 330, 388, 390-392, 394, 396-421];<br>2-month[292, 411, 422, 423];<br>50-day[424];<br>3-month[293, 294, 415, 425, 426];<br>100-day[315, 406, 407, 424];<br>4-month[292, 411, 422, 423];<br>5-month[424];<br>6-month[292-294, 298, 301, 302, 330, 388, 411, 415, 422, 423, 425-430];<br>200-day[315, 406, 407, 424];<br>8-month[292, 411, 422, 423];<br>250-day[424];<br>9-month[293, 294, 415, 425, 426];<br>10-month[292, 315, 406, 407, 411, 422-424];<br>1-year[292-294, 297-302, 304, 312, 315, 330, 385, 388, 389, 391, 392, 400, 403-407, 409, 410, 414, 415, 418, 420-422, 424-449];	1-year (including death, myocardial infarction, re-intervention, restenosis, and revascularization[421]);<br>4-year (including death, myocardial infarction, and revascularization[448])	Allergic reaction[385, 390], anaphylactic reaction[384], anemia[454], arrhythmia[330, 385, 390], bleeding[384, 385, 399, 400, 403, 405-408, 413, 418-420, 425, 443, 454, 455], bradycardia[387], cardiogenic shock[398, 400, 413, 414], cerebrovascular event[454], complication (unspecified)[384-386, 390, 391, 408, 414, 455, 456], coronary flow reserve[457], coronary occlusion[390], coronary perforation[330, 385, 390, 391], gastro-intestinal bleeding[454], heart failure[394, 400, 406, 416, 426, 434, 435, 441, 443, 447, 449], hemodynamic complication[385, 390, 414], in-hospital stay (length)[394], in-stent thrombosis[292-294, 297-302, 304, 306, 313, 315, 385, 391, 394, 402, 403, 405, 407, 411, 413, 414, 418, 420-423, 436, 441, 445, 447, 448, 458], intra-aortic balloon pump therapy[414], myocardial infarction[292-294, 299-303, 312, 384, 385, 389, 391, 399, 401, 406, 407, 410, 411, 421, 422, 424, 425, 427, 428, 430, 433, 435, 437, 441, 443, 447-450, 452, 459], neurological complication[315, 385, 390, 394, 413, 414, 418, 455], left ventricular ejection fraction[394], left ventricular dysfunction[411], pericardial tamponade[330, 384, 385, 390, 391, 411], procedural success[315, 384, 392, 409, 444], pseudoaneurysm[385], rehospitalization[391, 394, 416, 421, 426, 443, 450, 460], re-intervention[299, 330, 384, 385, 390, 391, 393, 403, 412, 421, 424, 447], renal failure[330, 384, 385, 447, 460], restenosis[292-294, 296-299, 301-303, 312, 385, 391, 414, 421-423, 429, 430, 433, 436, 445, 447, 448, 461], re-restenosis[295], resuscitation[389], revascularization[294, 385, 391, 394, 411, 414, 421, 422, 426, 433, 435, 441, 448, 452], side branch occlusion[330, 385, 391], stent loss[385, 390], stent thrombosis[292-294, 297-302, 304-306, 313, 315, 385, 391, 394, 402, 403, 405, 407, 411, 413, 414, 418, 420-423, 436, 441, 445, 447, 448, 458], stroke[315, 384, 394, 399, 401, 406, 411, 412, 416, 435, 443, 447, 449, 452], TIA[412], vascular complication[385, 390], ventricular fibrillation[387], ventricular tachycardia[387]

	14-month[292];<br>15-month[293, 294];<br>16-month[292];<br>18-month[292-294, 298, 301, 388, 427, 430];<br>20-month[292];<br>21-month[293, 294];<br>22-month[292];<br>2-year[292-294, 297-299, 301, 304, 312, 385, 388, 391, 392, 400, 412, 414, 418, 421, 423, 427, 428, 431, 432, 435, 436, 438-441, 444, 446-451];<br>730-day[438];<br>30-month[301, 388, 427, 430, 452];<br>3-year[297, 299, 301, 304, 385, 388, 391, 392, 400, 410, 412, 414, 416, 418, 421, 427, 428, 430-432, 434-436, 438, 439, 441, 444-449, 451];<br>42-month[388, 430];<br>4-year[297, 299, 385, 391, 400, 414, 418, 421, 423, 428, 430-432, 435, 438-441, 444, 446-449, 451, 453];<br>1460-day[438];<br>5-year[297, 299, 385, 391, 400, 406, 414, 418, 421, 428, 432, 435, 438, 439, 441, 444, 446, 447, 449, 451];<br>66-month[432];<br>6-year[299, 385, 391, 414, 418, 421, 423, 438-441, 443, 444, 446, 451];<br>2195-day[438];<br>7-year[299, 385, 391, 414, 418, 421, 438, 439, 446];<br>8-year[299, 385, 391, 414, 418, 421, 423, 438-440, 446, 451];<br>2920-day[438];<br>9-year[299, 385, 391, 418, 421, 438, 439];<br>10-year[299, 313, 385, 391, 418, 421, 423, 438-440, 451];<br>11-year[385, 391, 421];<br>12-year[421];<br>21-year[442].		
**Cardiovascular registries – valves**			
Quality Assurance Registry on Aortic Valve Replacement	In-hospital[29, 182-184];<br>1-month[184].	N/R	Aortic regurgitation[183], cerebrovascular event[29], complication (unspecified)[183, 184], delirium[182], in-hospital stay (length)[29, 182, 184], low cardiac output[29], myocardial infarction[29, 183], neurological complication[183], pacemaker implantation[182, 184], renal failure[29, 182, 184], resuscitation[29], stroke[182], TIA[182], vascular complication[183, 184]
Austrian TAVI Registry	1-month[11, 185];<br>6-month[185];<br>1-year[11, 185];	N/R	Complication (unspecified)[11], echographic findings[11], procedural success[11], quality-of-life[11], rehospitalization[11]

	18-month[185];<br>2-year[185];<br>30-month[185];<br>3-year[185].		
Belgian TAVI Registry	Procedural [186];<br>1-month[12, 186];<br>3-month[12];<br>6-month[12, 186];<br>1-year[12, 186];<br>18-month[12];<br>2-year[12, 187];<br>30-month[12];<br>3-year[12].	30-day (including death, myocardial infarction, pacemaker implantation, stroke, and TIA[12]); Unknown (including pacemaker implantation, renal failure, and stroke[187])	Echographic findings[186], pacemaker implantation[186], procedural success[12, 186], renal failure[186, 187], stroke[186], TIA[186], valve migration[12]
Czech TAVI Registry	5-day[462];<br>10-day[462];<br>15-day[462];<br>20-day[462];<br>25-day[462];<br>1-month [462].	N/R	Aortic regurgitation[13, 462], bleeding[462], cerebrovascular event[462], complication (unspecified)[191], coronary obstruction[462], in-hospital stay (length)[191], mitral regurgitation[462], NYHA classification[462], procedural success[462], rehospitalization[191]
FinnValve Registry	1-month[196, 308, 311];<br>1-year[30, 195-197, 308, 310, 311];<br>2-year[30, 195-197, 308, 310, 311];<br>3-year[30, 195, 197, 308, 310, 311];<br>4-year[30, 195, 197, 310, 311];<br>5-year[30, 195, 197, 311];<br>6-year[30, 195, 197];<br>7-year[195, 197];<br>8-year[30];<br>10-year[30].	N/R	Aortic annulus rupture[196], aortic dissection[196, 308], aortic peak gradient[30], atrial fibrillation[30, 196, 308, 310, 311], bleeding[30, 196, 308, 310, 311], blood transfusion[196, 308, 310, 311], coronary occlusion[196, 308], ECMO usage[196], ICU stay (length)[30], in-hospital stay (length)[30, 196, 308, 311], infection[30, 197, 308, 310], intra-aortic balloon pump therapy[196], pacemaker implantation[30, 196, 308], paravalvular regurgitation[196, 308], re-intervention[30, 196, 197, 308, 310, 311], renal failure[30, 196, 308, 310, 311], revascularization[308], stroke[30, 196, 308, 310, 311], vascular complication[196, 308, 310]
FRANCE TAVI Registry	Procedural[210];<br>In-hospital[198, 200, 203, 211, 218];<br>1-month[198, 199, 201, 202, 204, 205, 207, 209, 210, 212-214, 216, 218-220];<br>2-month[216];<br>3-month[200, 216];<br>100-day[205, 214, 219];<br>4-month[202, 210, 216];<br>5-month[216];<br>6-month[200, 201, 204, 205, 216, 220];	N/R	Acute coronary syndrome[222], acute occlusion (unspecified artery/vessel)[219], annulus rupture[14, 198, 204, 209, 211, 217], aortic dissection[14, 198, 211, 217], aortic regurgitation[201, 204, 205, 207, 212, 219, 220, 223], atrial fibrillation[214], blood transfusion[213], bleeding[198, 200-205, 207, 209, 213, 214, 216, 219, 220, 222, 223], echographic findings[14, 200, 208, 217], heart failure[201, 208], hemorrhagic shock[198], ICU stay (length)[204, 205, 207, 209, 213, 214, 219], infection[198, 199, 203, 223], in-hospital stay (length)[199, 204, 205, 207, 209, 211, 213, 214, 217, 219, 220], left ventricular ejection fraction[212], life threatening event[205, 214], mitral regurgitation[204, 205, 207, 219], myocardial infarction[14, 198-205, 207, 209, 214, 219, 220], NYHA classification[202, 216], pacemaker implantation[14, 198-201, 203-205, 207, 209, 211, 214, 217, 219, 220, 223], pericardial tamponade[14, 198, 204, 205, 207, 209, 211, 214, 217], peri-

	200-day[205, 214, 219];<br>7-month[216];<br>8-month[202, 210, 216];<br>9-month[200, 213, 216];<br>10-month[205, 214, 216, 219];<br>11-month[216];<br>1-year[200-205, 207, 209, 210, 212, 214-217, 219, 220];<br>400-day[205, 214, 219];<br>18-month[200, 212];<br>2-year[200, 203, 215, 217];<br>3-year[203, 215, 217];<br>4-year[208, 217];<br>5-year[217].		procedural complication (unspecified)[203, 223], procedural success[14, 199, 201, 204, 205, 207, 209-214, 218-220], pulmonary embolism[14, 198, 211], rehospitalization[199, 202], re-intervention[198, 200, 201, 204, 205, 207, 209, 214, 219], renal failure[14, 198, 201-205, 207, 209, 211, 213, 214, 219], stroke[14, 198-205, 207-209, 211, 213, 214, 216, 217, 219, 222], valve dysfunction[201, 202], valve migration[14, 198, 199, 211, 217, 220], vascular complication[198-205, 207, 209, 213, 214, 219, 220]
German Aortic Valve Registry	In-hospital[15, 31, 32, 224, 225, 228, 230-233, 235-237, 241];<br>5-day[240];<br>10-day[240];<br>15-day[232, 240];<br>20-day[240];<br>25-day[240];<br>1-month[32, 224, 228, 229, 232, 234, 238-240];<br>2-month[224, 228, 229, 232, 240];<br>3-month[230];<br>4-month[224, 228, 229, 232, 234, 240];<br>6-month[32, 224, 225, 228-230, 232, 238, 240];<br>8-month[224, 228, 229, 232, 234, 240];<br>9-month[230];<br>10-month[224, 228, 229, 240];<br>1-year[15, 32, 224, 225, 228-232, 234, 238-240];<br>2-year[15];<br>3-year[15];<br>4-year[15];<br>5-year[15].	1-year (including death, myocardial infarction, and stroke[32])	Aortic dissection[233], aortic regurgitation[31, 228, 230, 231, 233, 234, 237, 239-241], atrial fibrillation[31, 225, 228, 230, 231, 235, 240, 241], bleeding[32, 228, 230, 231, 233, 236, 241], blood transfusion[31, 234, 235, 237, 240], cerebrovascular event[237], conversion sternotomy[224, 230, 237], coronary occlusion[237], delirium[239], device malposition[224], echographic findings[236, 241], EQ-5D-3L scores[235], ICD implantation[32, 225, 231, 235, 236], ICU stay (length)[224, 225, 228, 231, 234-236, 241], infection[231, 234, 239, 241], in-hospital stay (length)[31, 224, 225, 228, 231, 235, 236, 240, 241], left ventricular decompensation[230, 237, 239], low cardiac output[32, 224, 231], mechanical ventilation (duration)[237], myocardial infarction[15, 31, 32, 224, 225, 228, 230, 231, 233-236, 238-240], NYHA classification[225, 231, 238], pacemaker implantation[15, 31, 32, 224, 225, 228, 230, 231, 233, 235-241], pericardial tamponade[231, 233, 237, 239, 240], procedural success[224, 230, 234], rehospitalization[225, 230, 231, 235], re-intervention[31, 32, 225, 233, 235-238, 241], renal failure[15, 31, 32, 224, 225, 228, 231, 233-237, 239, 241], respiratory failure[224, 239], resuscitation[32, 224], sepsis[230, 236, 239, 241], stroke[15, 31, 32, 224, 225, 228, 230, 231, 233-236, 238-241], thromboembolic event[230, 237], TIA[225, 228, 231, 235, 240, 241], vascular complication[32, 224, 228, 230, 231, 233, 234, 237, 239, 240]
Polish Registry of Transcatheter Aortic Valve Implantation	In-hospital[34, 243, 251];<br>1-month[248, 250, 251];<br>50-day[251];<br>100-day[251];<br>5-month[251];<br>6-month[246].<br>200-day[251];	30-day (including cardiovascular-related death, myocardial infarction, and stroke[249])	Bleeding[34, 244], mitral regurgitation[251], myocardial infarction[34], NYHA classification[243], paravalvular leakage[246], procedural success[34, 243, 250], quality of life[245], thromboembolic event[34], vascular access site complication[247], vascular complication[34]

	250-day[251];<br>10-month[251];<br>350-day[251];<br>400-day[251];<br>15-month[251];<br>500-day[251];<br>550-day[251].		
Spanish Registry of Heart Valves Repair	N/R	N/R	N/R
Swedish Transcatheter Cardiac Intervention Registry	In-hospital[36];<br>1-month[28];<br>6-month[36];<br>1-year[28, 36];<br>2-year[28, 36];<br>3-year[28, 36];<br>4-year[28, 36];<br>5-year[28, 36];<br>6-year[28, 36];<br>7-year[28];<br>8-year[28, 36].	N/R	In-hospital stay (length)[36], pacemaker implantation[36], re-intervention[36], stroke[28, 36]
Swiss TAVI Registry	Procedural[253, 274];<br>In-hospital[289];<br>5-day[255, 270, 282, 284];<br>10-day[255, 268, 270, 282, 284];<br>15-day[255, 270, 282, 284];<br>20-day[255, 268, 270, 282, 284];<br>25-day[255, 270, 282, 284];<br>1-month[20, 253, 255-258, 260, 262-266, 268-270, 272, 274, 275, 278, 280-282, 284-287, 291];<br>2-month[256-259, 262, 263, 266, 267, 269, 274, 275, 280, 283, 285-287, 291];<br>3-month[253, 256-259, 262, 263, 266, 267, 269, 275, 278, 280, 283, 285, 286, 291];<br>100-day[268];<br>4-month[256-259, 262, 263, 266, 267, 269, 274, 275, 278, 280, 283, 285-287, 291];<br>5-month[253, 256-259, 262, 263, 266, 267, 269, 275, 278, 280, 283, 285, 286, 291];	1-year (including cardiovascular-related death, myocardial infarction, and stroke[257])	Access site complication[20, 253, 255, 257, 258, 264, 272], annulus rupture[263, 266], aortic dissection[289], aortic regurgitation[20, 253, 263, 266, 272, 282, 290], aortic rupture[289], arrhythmia[260], atrial fibrillation[290], bleeding[20, 253, 255-257, 259, 260, 263-267, 272, 274, 278, 281, 282, 284-287, 290, 291], blood transfusion[253, 259], central venous pressure[276], cerebrovascular event[253, 259, 262, 267, 291], coronary occlusion[263, 266, 284, 289], effective orifice area[260], ICU stay (length)[253, 259, 282, 286, 289], in-hospital stay (length)[253, 257, 259, 281, 282, 285, 286, 289, 290], left bundle branch block[284], left ventricular function[281, 287], leg ischemia[289], mitral regurgitation[287], myocardial infarction[20, 253, 255-260, 262, 264, 266, 267, 271, 272, 274, 278, 282, 285-287, 291], NYHA classification[265, 275, 280], pacemaker implantation[20, 253, 259, 263-266, 272, 281, 282, 284-286, 289, 290], pericardial tamponade[263, 266, 284, 289], procedural success[263, 274], rehospitalization[265, 279], re-intervention[20, 260, 263, 264, 285, 289], renal failure[20, 253-255, 258, 260, 263, 264, 266, 267, 272, 278, 282, 285-287, 289], stroke[20, 253, 256-260, 263-266, 269, 271, 272, 274, 278, 281, 282, 285-287, 289, 291], TIA[256, 260, 271, 272, 274, 282, 285-287, 291], transvalvular gradient[260, 281], transvalvular pressure[288], valve dislocation[266, 284], valve dysfunction[265], valve migration[263, 289], valvular leakage[289], vascular complication[20, 263, 265, 266, 274, 281, 282, 284-287, 289)

	6-month[256-259, 262-264, 266, 267, 269, 274, 275, 278, 280, 281, 283, 285-287, 291];<br>200-day[268];<br>7-month[253, 256-259, 262, 263, 266, 267, 269, 275, 278, 280, 283, 285, 286, 291];<br>8-month[256-259, 262, 263, 266, 267, 269, 274, 275, 278, 280, 283, 285-287, 291];<br>9-month[253, 256-259, 262, 263, 266, 267, 269, 275, 278, 280, 283, 285, 286, 291];<br>10-month[256-259, 262, 263, 266-269, 274, 275, 278, 280, 283, 285-287, 291];<br>11-month[253, 256-259, 262, 263, 266, 269, 275, 278, 280, 283, 285, 286, 291];<br>1-year[253, 256-259, 262-269, 271, 272, 274-276, 278, 280, 281, 283, 285-287, 290, 291];<br>14-month[274, 287];<br>16-month[274, 287;]<br>18-month[264, 274, 287];<br>20-month[274, 287];<br>22-month[274, 287];<br>2-year[264, 271, 272, 274, 277, 287];<br>3-year[271, 272];<br>4-year[271, 272];<br>5-year[271, 272].		

Table S8A: Cardiovascular registries – Domain 'Safety & performance'

	Frequency of feedback	Level of feedback provided	Feedback (time period)	Outlier reports/procedures	Accessibility of results	Definition of outlier	Number of outliers identified
**Cardiovascular registries – combined**							
British Cardiovascular Intervention Society	Annually[1]	Hospital level[314]	N/R	Call to treatment time, door to treatment time, adverse outcome (CVA/needing emergency cardiac surgery), survival (only individual hospitals)[314]	Publicly available, and individual hospitals (survival data)[314]	Funnel plots using 3SD and 2SD (call to treatment, door to treatment, and survival)[314]	N/R
East Denmark Heart Registry	N/R	N/R	N/R	N/R	N/R	N/R	N/R
German Society for Thoracic and Cardiovascular Surgery	N/R	N/R	N/R	N/R	N/R	N/R	N/R
Polish National Database of Cardiac Surgery Procedures	N/R	N/R	N/R	N/R	N/R	N/R	N/R
Portuguese National Registry of Intervention Cardiology	N/R	N/R	N/R	N/R	N/R	N/R	N/R
Spanish Cardiac Catheterization and Coronary Intervention Registry	Annually[463]	N/R	N/R	N/R	N/R	N/R	N/R
Western Denmark Heart Registry	Annually and quarterly[25]	N/R	N/R	N/R	N/R	N/R	N/R
**Cardiovascular registries – stents**							
Polish National Percutaneous Coronary Intervention Registry	N/R	N/R	N/R	N/R	N/R	N/R	N/R
Swedish Coronary Angiography and Angioplasty Registry	Annually[10]	Hospital- and medical device-level[10, 464]	30-day (hospital level) and 1-year (medical device level)[10, 464]	N/R (30-day mortality after PCI in STEMI patients per hospital including mean and 95% and waiting time per hospital are reported (statistical testing unknown), stent thrombosis and restenosis in most used stents are reported (statistical testing unknown)[10, 464]	Publicly available[10, 464]	N/R	N/R
**Cardiovascular registries – valves**							
Quality Assurance Registry on Aortic Valve Replacement	N/R	N/R	N/R	N/R	N/R	N/R	N/R

Austrian-TAVI Registry	N/R	N/R	N/R	N/R	N/R	N/R	N/R
Belgian TAVI Registry	N/R	N/R	N/R	N/R	N/R	N/R	N/R
Czech TAVI Registry	N/R	N/R	N/R	N/R	N/R	N/R	N/R
FinnValve Registry	N/R	N/R	N/R	N/R	N/R	N/R	N/R
FRANCE-TAVI Registry	N/R	N/R	N/R	N/R	N/R	N/R	N/R
German Aortic Valve Registry	Annually[(33)]	Hospital level[(33)]	N/R	N/R (Individual hospitals' results are compared with the entire registry data; statistical testing unknown)[(33)]	Individual hospitals[(33)]	N/R	N/R
Polish Registry of Transcatheter Aortic Valve Implantation	N/R	N/R	N/R	N/R	N/R	N/R	N/R
Spanish Registry of Heart Valves Repair	N/R	N/R	N/R	N/R	N/R	N/R	N/R
Swedish Transcatheter Cardiac Intervention Registry	N/R	N/R	N/R	N/R	N/R	N/R	N/R
Swiss TAVI Registry	N/R	N/R	N/R	N/R	N/R	N/R	N/R

References, cardiovascular

1. The British Cardiovascular Intervention Society (BCIS). https://www.bcis.org.uk/ (accessed March 21. 2022)
2. De Backer O, Luk NH, Olsen NT, Olsen PS, Søndergaard L. Choice of Treatment for Aortic Valve Stenosis in the Era of Transcatheter Aortic Valve Replacement in Eastern Denmark (2005 to 2015). JACC Cardiovasc Interv. 2016;9(11):1152-8.
3. Beckmann A, Funkat AK, Lewandowski J, Frie M, Ernst M, Hekmat K, et al. German Heart Surgery Report 2016: The Annual Updated Registry of the German Society for Thoracic and Cardiovascular Surgery. Thorac Cardiovasc Surg. 2017;65(7):505-18.
4. Deutsche Gesellschaft für Thorax-, Herz- und Gefäßchirurgie (DGTHG). www.dgthg.de/ (accessed March 21. 2022)
5. Bartus K, Litwinowicz R, Sadowski J, Filip G, Kowalewski M, Suwalski P, et al. Bioprosthetic or mechanical heart valves: prosthesis choice for borderline patients?-Results from 9,616 cases recorded in Polish national cardiac surgery registry. J Thorac Dis. 2020;12(10):5869-78.
6. Sousa P, Uva AS, Pinto F. Risk-adjustment model in health outcomes evaluation: a contribution to strengthen assessment towards quality improvement in interventional cardiology. Int J Qual Health Care. 2008;20(5):324-30.
7. Cid Álvarez AB, Rodríguez Leor O, Moreno R, Pérez de Prado A. Spanish Cardiac Catheterization and Coronary Intervention Registry. 27th Official Report of the Spanish Society of Cardiology Working Group on Cardiac Catheterization and Interventional Cardiology (1990-2017). Rev Esp Cardiol (Engl Ed). 2018;71(12):1036-46.
8. Schmidt M, Maeng M, Madsen M, Sørensen HT, Jensen LO, Jakobsen CJ. The Western Denmark Heart Registry: Its Influence on Cardiovascular Patient Care. J Am Coll Cardiol. 2018;71(11):1259-72.
9. Swedish Coronary Angiography and Angioplasty Registry (SCAAR). https://www.ucr.uu.se/swedeheart/start-scaar/ (accessed February 27. 2023)
10. SWEDEHEART. SWEDEHEART Annual report 2020. 2021. Accessible: https://www.ucr.uu.se/swedeheart/dokument-sh/arsrapporter-sh/arsrapport-2020/1-swedeheart-annual-report-2020-english-2
11. Zweiker D, Maier R, Lamm G, Maurer E, Heigert M, Neunteufl T, et al. The Austrian transcatheter aortic valve implantation (TAVI) Registry--3 years' data. Int J Cardiol. 2014;177(1):114-6.
12. Collas VM, Dubois C, Legrand V, Kefer J, De Bruyne B, Dens J, et al. Midterm clinical outcome following Edwards SAPIEN or Medtronic Corevalve transcatheter aortic valve implantation (TAVI): Results of the Belgian TAVI registry. Catheter Cardiovasc Interv. 2015;86(3):528-35.
13. Kala P, Blaha M, Mates M, Želízko M, Branny M, Kočka V, et al. Czech TAVI registry – Hospital outcome. Cor et Vasa. 2017;59(1):e51-e6.
14. Auffret V, Lefevre T, Van Belle E, Eltchaninoff H, Iung B, Koning R, et al. Temporal Trends in Transcatheter Aortic Valve Replacement in France: FRANCE 2 to FRANCE TAVI. J Am Coll Cardiol. 2017;70(1):42-55.

15. Beyersdorf F, Bauer T, Freemantle N, Walther T, Frerker C, Herrmann E, et al. Five-year outcome in 18 010 patients from the German Aortic Valve Registry. Eur J Cardiothorac Surg. 2021;60(5):1139-46.
16. Zembala-John J, Wilczek K, Tobota Z, Chodór P, Cieśla D, Jaźwiec T, et al. POL-TAVI - Polish Registry of Transcatheter Aortic Valve Implantation - simple tool, great value, rationale and design. Kardiochir Torakochirurgia Pol. 2016;13(4):309-15.
17. Sáez de Ibarra JI, Sitges M, Silva J, García-Fuster R, Evangelista A, Borras X, et al. Registro Español de Reparación Valvular 2009-2011. Cirugía Cardiovascular. 2013;20(3):144-9.
18. Jorge Rodríguez-Rodaa JISdI, Javier Gualisc, Pedro Limad, Carmen Iglesiase, Rafael García-Fusterf, Carlos Porrasg, Delfina Fletcherh, Francisco Gutiérrez-Garcíai, Manel Castellàj, Yolanda Carrascalk, Eduardo Bernabeul,v, Luis Delgadom, Tomás Darocan, Carlos Moraleso, Fabrizio Sbragap, José M. González-Santosq, Carlos E. Martínr, Juan J. Oteros, Alejandro Adsuart, Rafael Rodríguezu, Rafael Llorensw, Ana M. Belx, Miguel Gomez-Vidaly. Registro español de reparación valvular 2016-2017. Cirugía Cardiovascular. 2019;26:147-52.
19. Swedish Transcatheter Cardiac Intervention Registry (SWENTRY). https://www.ucr.uu.se/swedeheart/start-swentry (accessed March 21. 2022)
20. Noble S, Stortecky S, Heg D, Tueller D, Jeger R, Toggweiler S, et al. Comparison of procedural and clinical outcomes with Evolut R versus Medtronic CoreValve: a Swiss TAVI registry analysis. EuroIntervention. 2017;12(18):e2170-e6.
21. Ludman PF. UK TAVI registry. Heart. 2019;105(Suppl 2):s2-s5.
22. Jakobsen L, Terkelsen CJ, Søndergaard L, De Backer O, Aarøe J, Nissen H, et al. Short- and Long-Term Mortality and Stroke Risk After Transcatheter Aortic Valve Implantation. Am J Cardiol. 2018;121(1):78-85.
23. Pereira H, Caldeira D, Teles RC, Costa M, da Silva PC, da Gama Ribeiro V, et al. Thrombus aspiration in patients with ST-elevation myocardial infarction: results of a national registry of interventional cardiology. BMC Cardiovasc Disord. 2018;18(1):69.
24. Krasniqi L, Kronby MP, Riber LPS. Long-term survival after Carpentier-Edwards Perimount aortic valve replacement in Western Denmark: a multi-centre observational study. J Cardiothorac Surg. 2021;16(1):130.
25. Rasmussen LA, Bøtker HE, Jensen LO, Ravkilde J, Riber L, Nielsen PH, et al. Quality assurance of the Western Denmark Heart Registry, a population-based healthcare register. Dan Med J. 2017;64(10).
26. Glaser N, Jackson V, Holzmann MJ, Franco-Cereceda A, Sartipy U. Late Survival After Aortic Valve Replacement in Patients With Moderately Reduced Kidney Function. J Am Heart Assoc. 2016;5(12).
27. SWEDEHEART. https://www.ucr.uu.se/swedeheart/ (accessed March 21. 2022)
28. Bjursten H, Norrving B, Ragnarsson S. Late stroke after transcatheter aortic valve replacement: a nationwide study. Sci Rep. 2021;11(1):9593.
29. Bestehorn K, Eggebrecht H, Fleck E, Bestehorn M, Mehta RH, Kuck KH. Volume-outcome relationship with transfemoral transcatheter aortic valve implantation (TAVI): insights from the compulsory German Quality Assurance Registry on Aortic Valve Replacement (AQUA). EuroIntervention. 2017;13(8):914-20.

30. Dahlbacka S, Laakso T, Kinnunen EM, Moriyama N, Laine M, Virtanen M, et al. Patient-Prosthesis Mismatch Worsens Long-Term Survival: Insights From the FinnValve Registry. Ann Thorac Surg. 2021;111(4):1284-90.
31. Abdel-Wahab M, Fujita B, Frerker C, Bauer T, Beckmann A, Bekeredjian R, et al. Transcatheter Versus Rapid-Deployment Aortic Valve Replacement: A Propensity-Matched Analysis From the German Aortic Valve Registry. JACC Cardiovasc Interv. 2020;13(22):2642-54.
32. Lauten A, Figulla HR, Möllmann H, Holzhey D, Kötting J, Beckmann A, et al. TAVI for low-flow, low-gradient severe aortic stenosis with preserved or reduced ejection fraction: a subgroup analysis from the German Aortic Valve Registry (GARY). EuroIntervention. 2014;10(7):850-9.
33. Beckmann A, Hamm C, Figulla HR, Cremer J, Kuck KH, Lange R, et al. The German Aortic Valve Registry (GARY): a nationwide registry for patients undergoing invasive therapy for severe aortic valve stenosis. Thorac Cardiovasc Surg. 2012;60(5):319-25.
34. Czerwińska-Jelonkiewicz K, Zembala M, Dąbrowski M, Witkowski A, Ochała A, Kochman J, et al. Can TAVI patients receive aspirin monotherapy as patients after surgical aortic bioprosthesis implantation? Data from the Polish Registry - POL-TAVI. Int J Cardiol. 2017;227:305-11.
35. Carrascal Y, Ibarra J, Lecoq R, González Santos J, Salazar Á, Daroca T, et al. Registro Español de Reparación Valvular 2014. Cirugía Cardiovascular. 2016;23.
36. Bjursten H, Rasmussen M, Nozohoor S, Götberg M, Olaison L, Rück A, et al. Infective endocarditis after transcatheter aortic valve implantation: a nationwide study. Eur Heart J. 2019;40(39):3263-9.
37. Ludman P. British Cardiovascular Intervention Society database: insights into interventional cardiology in the United Kingdom. Heart. 2019;105(16):1289.
38. Millan-Iturbe O, Sawaya FJ, Lønborg J, Chow DHF, Bieliauskas G, Engstrøm T, et al. Coronary artery disease, revascularization, and clinical outcomes in transcatheter aortic valve replacement: Real-world results from the East Denmark Heart Registry. Catheter Cardiovasc Interv. 2018;92(4):818-26.
39. Joshi FR, Biasco L, Pedersen F, Holmvang L, Helqvist S, Tilsted HH, et al. Invasive angiography and revascularization in patients with stable angina following prior coronary artery bypass grafting: Results from the East Denmark heart registry. Catheter Cardiovasc Interv. 2017;89(3):341-9.
40. Beckmann A, Meyer R, Lewandowski J, Markewitz A, Gummert J. German Heart Surgery Report 2020: The Annual Updated Registry of the German Society for Thoracic and Cardiovascular Surgery. Thorac Cardiovasc Surg. 2021;69(4):294-307.
41. Beckmann A, Funkat A-K, Lewandowski J, Frie M, Ernst M, Hekmat K, et al. Cardiac Surgery in Germany during 2014: A Report on Behalf of the German Society for Thoracic and Cardiovascular Surgery. Thorac Cardiovasc Surg. 2015;63(04):258-69.
42. Bartus K, Sadowski J, Litwinowicz R, Filip G, Jasinski M, Deja M, et al. Changing trends in aortic valve procedures over the past ten years-from mechanical prosthesis via stented bioprosthesis to TAVI procedures-analysis of 50,846 aortic valve cases based on a Polish National Cardiac Surgery Database. J Thorac Dis. 2019;11(6):2340-9.

43. Trzeciak P, Karolak W, Gąsior M, Zembala M. In-hospital and long-term outcomes of coronary artery bypass graft surgery in patients ≤ 45 years of age and older (from the KROK registry). Kardiol Pol. 2017;75(9):884-92.
44. Knapik P, Knapik M, Zembala MO, Przybyłowski P, Nadziakiewicz P, Hrapkowicz T, et al. In-hospital and mid-term outcomes in patients reoperated on due to bleeding following coronary artery surgery (from the KROK Registry). Interact Cardiovasc Thorac Surg. 2019.
45. Kowalewski M, Jasiński M, Staromłyński J, Zembala M, Widenka K, Zembala MO, et al. Long-Term Survival Following Surgical Ablation for Atrial Fibrillation Concomitant to Isolated and Combined Coronary Artery Bypass Surgery-Analysis from the Polish National Registry of Cardiac Surgery Procedures (KROK). J Clin Med. 2020;9(5).
46. Main cardiac surgery procedures performed in Poland in 2013 (according to the National Registry of Cardiac Surgery Procedures - KROK, Warsaw, Poland 2014). Kardiochir Torakochirurgia Pol. 2014;11(3):349-52.
47. Main cardiac surgery procedures performed in Poland in 2014 (according to the National Registry of Cardiac Surgery Procedures - KROK, Warsaw, Poland 2015). Kardiochir Torakochirurgia Pol. 2015;12(3):288-91.
48. Kowalewski M, Pasierski M, Litwinowicz R, Zembala M, Piekuś-Słomka N, Tobota Z, et al. Multiple Versus Single Arterial Coronary Arterial Bypass Grafting Surgery for Multivessel Disease in Atrial Fibrillation. Semin Thorac Cardiovasc Surg. 2021;33(4):974-83.
49. Knapik P, Hirnle G, Kowalczuk-Wieteska A, M OZ, Pawlak S, Hrapkowicz T, et al. Off-pump versus on-pump coronary artery surgery in octogenarians (from the KROK Registry). PLoS One. 2020;15(9):e0238880.
50. Kowalewski M, Jasiński M, Staromłyński J, Zembala M, Widenka K, Brykczyński M, et al. On-Pump vs Off-Pump coronary artery bypass surgery in atrial fibrillation. Analysis from the polish national registry of cardiac surgery procedures (KROK). PLoS One. 2020;15(4):e0231950.
51. Knapik P, Cieśla D, Saucha W, Knapik M, Zembala MO, Przybyłowski P, et al. Outcome Prediction After Coronary Surgery and Redo Surgery for Bleeding (From the KROK Registry). J Cardiothorac Vasc Anesth. 2019;33(11):2930-7.
52. Suwalski P, Kowalewski M, Jasiński M, Staromłyński J, Zembala M, Widenka K, et al. Surgical ablation for atrial fibrillation during isolated coronary artery bypass surgery. Eur J Cardiothorac Surg. 2020;57(4):691-700.
53. Suwalski P, Kowalewski M, Jasiński M, Staromłyński J, Zembala M, Widenka K, et al. Survival after surgical ablation for atrial fibrillation in mitral valve surgery: Analysis from the Polish National Registry of Cardiac Surgery Procedures (KROK). J Thorac Cardiovasc Surg. 2019;157(3):1007-18.e4.
54. Deja MA, Malinowski M, Widenka K, Stożyński N, Bartuś K, Kapelak B, et al. Repair or Replacement for Secondary Mitral Regurgitation: Results From Polish National Registry. Ann Thorac Surg. 2022;113(1):146-56.
55. Kowalczuk-Wieteska A, Parys M, Pawlaczyk R, Filipiak K, Cisowski M, Bochenek A, et al. Prediction of complications and death in octogenarians with left main coronary

artery disease after coronary artery bypass implantation - off-pump, on-pump and minimally invasive techniques comparison. Postepy Kardiol Interwencyjnej. 2019;15(2):218-25.
56. Krajowego Rejestru Operacji Kardiochirurgicznych (KROK). https://krok.csioz.gov.pl/krok/ (accessed March 21. 2022)
57. Caldeira D, Pereira H, Marques A, Alegria S, Calisto J, Silva PCD, et al. Adjuvant antithrombotic therapy in ST-elevation myocardial infarction: Contemporaneous Portuguese cross-sectional data. Rev Port Cardiol (Engl Ed). 2019;38(11):809-14.
58. Teles RC, Pires-Morais G, da Silva PC, Ferreira RC, de Sousa Almeida M, Seixo F, et al. Portugal: coronary and structural heart interventions from 2010 to 2015. EuroIntervention. 2017;13(Z):Z55-z8.
59. Romaguera R, Ojeda S, Cruz-González I, Moreno R. Spanish Cardiac Catheterization and Coronary Intervention Registry. 30th Official Report of the Interventional Cardiology Association of the Spanish Society of Cardiology (1990-2020) in the year of the COVID-19 pandemic. Rev Esp Cardiol (Engl Ed). 2021;74(12):1095-105.
60. Cid Álvarez AB, Rodríguez Leor O, Moreno R, Pérez de Prado A. Spanish Cardiac Catheterization and Coronary Intervention Registry. 28th Official Report of the Spanish Society of Cardiology Working Group on Cardiac Catheterization and Interventional Cardiology (1990-2018). Rev Esp Cardiol (Engl Ed). 2019;72(12):1043-53.
61. de Thurah A, Andersen IT, Tinggaard AB, Riis AH, Therkildsen J, Bøtker HE, et al. Risk of major adverse cardiovascular events among patients with rheumatoid arthritis after initial CT-based diagnosis and treatment. RMD Open. 2020;6(1).
62. Kaltoft A, Jensen LO, Maeng M, Tilsted HH, Thayssen P, Bøttcher M, et al. 2-year clinical outcomes after implantation of sirolimus-eluting, paclitaxel-eluting, and bare-metal coronary stents: results from the WDHR (Western Denmark Heart Registry). J Am Coll Cardiol. 2009;53(8):658-64.
63. Kremke M, Tang M, Bak M, Kristensen KL, Hindsholm K, Andreasen JJ, et al. Antiplatelet therapy at the time of coronary artery bypass grafting: a multicentre cohort study. Eur J Cardiothorac Surg. 2013;44(2):e133-40.
64. Rasmussen LD, Bøttcher M, Ivarsen P, Jørgensen HS, Nyegaard M, Buttenschøn H, et al. Association between circulating proprotein convertase subtilisin/kexin type 9 levels and prognosis in patients with severe chronic kidney disease. Nephrol Dial Transplant. 2020;35(4):632-9.
65. Mortensen MB, Gaur S, Frimmer A, Bøtker HE, Sørensen HT, Kragholm KH, et al. Association of Age With the Diagnostic Value of Coronary Artery Calcium Score for Ruling Out Coronary Stenosis in Symptomatic Patients. JAMA Cardiol. 2022;7(1):36-44.
66. Dupont L, Winther S, Jørgensen HS, Bomholt P, Bøtker HE, Bøttcher M, et al. Atrial function, atrial volume and cardiovascular clinical outcomes in patients with end-stage renal disease - A study of cardiac computed tomography. J Cardiovasc Comput Tomogr. 2017;11(5):389-96.
67. Fedosova M, Kimose HH, Greisen JR, Fast P, Gissel MS, Jakobsen CJ. Blood cardioplegia benefits only patients with a long cross-clamp time. Perfusion. 2019;34(1):42-9.
68. Mortensen MB, Steffensen FH, Bøtker HE, Jensen JM, Rønnow Sand NP, Kragholm KH, et al. CAD Severity on Cardiac CTA Identifies Patients With Most Benefit of Treating

LDL-Cholesterol to ACC/AHA and ESC/EAS Targets. JACC Cardiovasc Imaging. 2020;13(9):1961-72.
69. Jensen LO, Maeng M, Thayssen P, Kaltoft A, Tilsted HH, Bøttcher M, et al. Clinical outcome after primary percutaneous coronary intervention with drug-eluting and bare metal stents in patients with ST-segment elevation myocardial infarction. Circ Cardiovasc Interv. 2008;1(3):176-84.
70. Antonsen L, Jensen LO, Thayssen P, Christiansen EH, Junker A, Tilsted HH, et al. Comparison of outcomes of patients ≥ 80 years of age having percutaneous coronary intervention according to presentation (stable vs unstable angina pectoris/non-ST-segment elevation myocardial infarction vs ST-segment elevation myocardial infarction). Am J Cardiol. 2011;108(10):1395-400.
71. Maeng M, Jensen LO, Kaltoft A, Tilsted HH, Christiansen EH, Thayssen P, et al. Comparison of zotarolimus-eluting and sirolimus-eluting coronary stents: a study from the Western Denmark Heart Registry. BMC Cardiovasc Disord. 2012;12:84.
72. Kugathasan P, Johansen MB, Jensen MB, Aagaard J, Nielsen RE, Jensen SE. Coronary Artery Calcification and Mortality Risk in Patients With Severe Mental Illness. Circ Cardiovasc Imaging. 2019;12(3):e008236.
73. Vinter N, Christesen AMS, Mortensen LS, Urbonaviciene G, Lindholt J, Johnsen SP, et al. Coronary artery calcium score and the long-term risk of atrial fibrillation in patients undergoing non-contrast cardiac computed tomography for suspected coronary artery disease: a Danish registry-based cohort study. Eur Heart J Cardiovasc Imaging. 2018;19(8):926-32.
74. Thim T, Egholm G, Olesen KKW, Madsen M, Jensen SE, Jensen LO, et al. Coronary stent implantation and adverse cardiac events after surgery. Eur J Clin Invest. 2018;48(12):e13030.
75. Nørgaard KS, Isaksen C, Buhl JS, Nielsen AH, Nørgaard A, Urbonaviciene G, et al. CT coronary angiography in low- to intermediate-risk patients: less radiation, less invasive angiography, and less revascularisation. Scand Cardiovasc J. 2014;48(5):265-70.
76. Jensen LO, Thayssen P, Farkas DK, Hougaard M, Terkelsen CJ, Tilsted HH, et al. Culprit only or multivessel percutaneous coronary interventions in patients with ST-segment elevation myocardial infarction and multivessel disease. EuroIntervention. 2012;8(4):456-64.
77. Gyldenkerne C, Olesen KK, Thrane PG, Madsen M, Thim T, Würtz M, et al. Diabetes is not a risk factor for myocardial infarction in patients without coronary artery disease: A study from the Western Denmark Heart Registry. Diab Vasc Dis Res. 2020;17(4):1479164120941809.
78. Sørensen JT, Stengaard C, Sørensen CA, Thygesen K, Bøtker HE, Thuesen L, et al. Diagnosis and outcome in a prehospital cohort of patients with bundle branch block and suspected acute myocardial infarction. Eur Heart J Acute Cardiovasc Care. 2013;2(2):176-81.
79. Schmidt M, Maeng M, Jakobsen CJ, Madsen M, Thuesen L, Nielsen PH, et al. Existing data sources for clinical epidemiology: The Western Denmark Heart Registry. Clin Epidemiol. 2010;2:137-44.
80. Gyldenkerne C, Olesen KKW, Madsen M, Thim T, Jensen LO, Raungaard B, et al. Extent of coronary artery disease is associated with myocardial infarction and mortality in patients with diabetes mellitus. Clin Epidemiol. 2019;11:419-28.

81. Würtz M, Olesen KKW, Thim T, Kristensen SD, Eikelboom JW, Maeng M. External applicability of the COMPASS trial: the Western Denmark Heart Registry. Eur Heart J Cardiovasc Pharmacother. 2019;5(4):192-9.
82. Hansen LS, Sloth E, Hjortdal VE, Jakobsen CJ. Follow-Up After Cardiac Surgery Should be Extended to at Least 120 Days When Benchmarking Cardiac Surgery Centers. J Cardiothorac Vasc Anesth. 2015;29(4):984-9.
83. Terkelsen CJ, Jensen LO, Tilsted HH, Trautner S, Johnsen SP, Vach W, et al. Health care system delay and heart failure in patients with ST-segment elevation myocardial infarction treated with primary percutaneous coronary intervention: follow-up of population-based medical registry data. Ann Intern Med. 2011;155(6):361-7.
84. Nielsen DV, Hansen MK, Johnsen SP, Hansen M, Hindsholm K, Jakobsen CJ. Health outcomes with and without use of inotropic therapy in cardiac surgery: results of a propensity score-matched analysis. Anesthesiology. 2014;120(5):1098-108.
85. Mortensen MB, Steffensen FH, Bøtker HE, Jensen JM, Rønnow Sand NP, Kragholm KH, et al. Heterogenous Distribution of Risk for Cardiovascular Disease Events in Patients With Stable Ischemic Heart Disease. JACC Cardiovasc Imaging. 2021;14(2):442-50.
86. Kann SH, Thomassen SA, Abromaitiene V, Jakobsen CJ. ICU Nurses-An Impact Factor on Patient Turnover in Cardiac Surgery in Western Denmark? J Cardiothorac Vasc Anesth. 2021.
87. Jensen LO, Maeng M, Thayssen P, Lassen JF, Bøtker HE, Rasmussen K, et al. Impact of drug eluting stents on target vessel revascularization. A report from the Western Denmark heart registry from 2000 to 2004. EuroIntervention. 2006;1(4):391-5.
88. Atladottir HO, Modrau IS, Jakobsen CJ, Torp-Pedersen CT, Gissel MS, Nielsen DV. Impact of perioperative course during cardiac surgery on outcomes in patients 80 years and older. J Thorac Cardiovasc Surg. 2021;162(5):1568-77.
89. Mortensen MB, Dzaye O, Steffensen FH, Bøtker HE, Jensen JM, Rønnow Sand NP, et al. Impact of Plaque Burden Versus Stenosis on Ischemic Events in Patients With Coronary Atherosclerosis. J Am Coll Cardiol. 2020;76(24):2803-13.
90. Løgstrup BB, Olesen KKW, Masic D, Gyldenkerne C, Thrane PG, Ellingsen T, et al. Impact of rheumatoid arthritis on major cardiovascular events in patients with and without coronary artery disease. Ann Rheum Dis. 2020;79(9):1182-8.
91. Jensen JK, Jensen LO, Terkelsen CJ, Lassen JF, Tilsted HH, Hansen KN, et al. Incidence of definite stent thrombosis or in-stent restenosis after drug-eluting stent implantation for treatment of coronary in-stent restenosis: from Western Denmark Heart Registry. Catheter Cardiovasc Interv. 2013;81(2):260-5.
92. Jensen LO, Maeng M, Thayssen P, Tilsted HH, Terkelsen CJ, Kaltoft A, et al. Influence of diabetes mellitus on clinical outcomes following primary percutaneous coronary intervention in patients with ST-segment elevation myocardial infarction. Am J Cardiol. 2012;109(5):629-35.
93. Jensen LO, Terkelsen CJ, Horváth-Puhó E, Tilsted HH, Maeng M, Junker A, et al. Influence of multivessel disease with or without additional revascularization on mortality in patients with ST-segment elevation myocardial infarction. Am Heart J. 2015;170(1):70-8.

94. Mortensen MB, Dzaye O, Bødtker H, Steffensen FH, Bøtker HE, Jensen JM, et al. Interplay of Risk Factors and Coronary Artery Calcium for CHD Risk in Young Patients. JACC Cardiovasc Imaging. 2021;14(12):2387-96.
95. Nielsen DV, Torp-Pedersen C, Skals RK, Gerds TA, Karaliunaite Z, Jakobsen CJ. Intraoperative milrinone versus dobutamine in cardiac surgery patients: a retrospective cohort study on mortality. Crit Care. 2018;22(1):51.
96. Nielsen DV, Fedosova M, Hjortdal V, Jakobsen CJ. Is single-dose prophylactic gentamicin associated with acute kidney injury in patients undergoing cardiac surgery? A matched-pair analysis. J Thorac Cardiovasc Surg. 2014;148(4):1634-9.
97. Hougaard M, Thayssen P, Kaltoft A, Tilsted HH, Maeng M, Lassen JF, et al. Long-term outcome following percutaneous coronary intervention with drug-eluting stents compared with bare-metal stents in saphenous vein graft lesions: from Western Denmark Heart Registry. Catheter Cardiovasc Interv. 2014;83(7):1035-42.
98. Jensen LO, Maeng M, Thayssen P, Kaltoft A, Tilsted HH, Lassen JF, et al. Long-term outcomes after percutaneous coronary intervention in patients with and without diabetes mellitus in Western Denmark. Am J Cardiol. 2010;105(11):1513-9.
99. Azimi A, Charlot MG, Torp-Pedersen C, Gislason GH, Køber L, Jensen LO, et al. Moderate overweight is beneficial and severe obesity detrimental for patients with documented atherosclerotic heart disease. Heart. 2013;99(9):655-60.
100. Schmidt M, Pedersen L, Maeng M, Lassen JF, Lash TL, Nielsen TT, et al. Nonsteroidal antiinflammatory drug use and cardiovascular risks after coronary stent implantation. Pharmacotherapy. 2011;31(5):458-68.
101. Jensen LO, Kaltoft A, Thayssen P, Tilsted HH, Christiansen EH, Mikkelsen KV, et al. Outcome in high risk patients with unprotected left main coronary artery stenosis treated with percutaneous coronary intervention. Catheter Cardiovasc Interv. 2010;75(1):101-8.
102. Modrau IS, Nielsen PH, Nielsen DV, Christiansen EH, Hoffmann T, Parner ET, et al. Outcome of hybrid compared to conventional revascularization in multivessel coronary artery disease. Scand Cardiovasc J. 2020;54(6):376-82.
103. Antonsen L, Jensen LO, Terkelsen CJ, Tilsted HH, Junker A, Maeng M, et al. Outcomes after primary percutaneous coronary intervention in octogenarians and nonagenarians with ST-segment elevation myocardial infarction: from the Western Denmark heart registry. Catheter Cardiovasc Interv. 2013;81(6):912-9.
104. Jensen LO, Tilsted HH, Thayssen P, Kaltoft A, Maeng M, Lassen JF, et al. Paclitaxel and sirolimus eluting stents versus bare metal stents: long-term risk of stent thrombosis and other outcomes. From the Western Denmark Heart Registry. EuroIntervention. 2010;5(8):898-905.
105. Olesen KKW, Madsen M, Egholm G, Thim T, Jensen LO, Raungaard B, et al. Patients With Diabetes Without Significant Angiographic Coronary Artery Disease Have the Same Risk of Myocardial Infarction as Patients Without Diabetes in a Real-World Population Receiving Appropriate Prophylactic Treatment. Diabetes Care. 2017;40(8):1103-10.
106. Olesen KKW, Gyldenkerne C, Thim T, Thomsen RW, Maeng M. Peripheral artery disease, lower limb revascularization, and amputation in diabetes patients with and without coronary artery disease: a cohort study from the Western Denmark Heart Registry. BMJ Open Diabetes Res Care. 2021;9(1).

107. Gu J, Andreasen JJ, Melgaard J, Lundbye-Christensen S, Hansen J, Schmidt EB, et al. Preoperative Electrocardiogram Score for Predicting New-Onset Postoperative Atrial Fibrillation in Patients Undergoing Cardiac Surgery. J Cardiothorac Vasc Anesth. 2017;31(1):69-76.
108. Vinter N, Christesen AMS, Mortensen LS, Lindholt JS, Johnsen SP, Tjønneland A, et al. Relation of Coronary Artery Calcium Score and Risk of Cancer (from a Danish Population-Based Follow-up Study in Patients Who Underwent Cardiac Computed Tomography). Am J Cardiol. 2017;120(4):542-9.
109. Hansen LS, Hjortdal VE, Jakobsen CJ. Relocation of patients after cardiac surgery: is it worth the effort? Acta Anaesthesiol Scand. 2016;60(4):441-9.
110. Egholm G, Kristensen SD, Thim T, Olesen KK, Madsen M, Jensen SE, et al. Risk Associated With Surgery Within 12 Months After Coronary Drug-Eluting Stent Implantation. J Am Coll Cardiol. 2016;68(24):2622-32.
111. Chisholm GE, Grejs A, Thim T, Christiansen EH, Kaltoft A, Lassen JF, et al. Safety of therapeutic hypothermia combined with primary percutaneous coronary intervention after out-of-hospital cardiac arrest. Eur Heart J Acute Cardiovasc Care. 2015;4(1):60-3.
112. Steensig K, Olesen KKW, Thim T, Nielsen JC, Jensen SE, Jensen LO, et al. Should the Presence or Extent of Coronary Artery Disease be Quantified in the CHA2DS2-VASc Score in Atrial Fibrillation? A Report from the Western Denmark Heart Registry. Thromb Haemost. 2018;118(12):2162-70.
113. Nørgaard KS, Isaksen C, Buhl JS, Kirk Johansen J, Nielsen AH, Nørgaard A, et al. Single-centre cohort study of gender influence in coronary CT angiography in patients with a low to intermediate pretest probability of coronary heart disease. Open Heart. 2015;2(1):e000233.
114. Gleerup HB, Dahm CC, Thim T, Jensen SE, Jensen LO, Kristensen SD, et al. Smoking is the dominating modifiable risk factor in younger patients with STEMI. Eur Heart J Acute Cardiovasc Care. 2020;9(1):70-5.
115. Mortensen KH, Thuesen L, Kristensen IB, Christiansen EH. Spontaneous coronary artery dissection: a Western Denmark Heart Registry study. Catheter Cardiovasc Interv. 2009;74(5):710-7.
116. Layton JB, Hansen MK, Jakobsen CJ, Kshirsagar AV, Andreasen JJ, Hjortdal VE, et al. Statin initiation and acute kidney injury following elective cardiovascular surgery: a population cohort study in Denmark†. Eur J Cardiothorac Surg. 2016;49(3):995-1000.
117. Jensen LO, Maeng M, Kaltoft A, Thayssen P, Hansen HH, Bottcher M, et al. Stent thrombosis, myocardial infarction, and death after drug-eluting and bare-metal stent coronary interventions. J Am Coll Cardiol. 2007;50(5):463-70.
118. Gu J, Skals RK, Torp-Pedersen C, Lundbye-Christensen S, Jakobsen CJ, Bæch J, et al. Storage time of intraoperative transfused allogeneic red blood cells is not associated with new-onset postoperative atrial fibrillation in cardiac surgery. PLoS One. 2017;12(2):e0172726.
119. Maeng M, Okkels Jensen L, Rasmussen K, Flensted Lassen J, Romer Krusell L, Thayssen P, et al. Target lesion revascularisation in patients treated with a sirolimus-eluting or paclitaxel-eluting stent. Heart. 2007;93(6):694-7.

120. Olesen KKW, Madsen M, Gyldenkerne C, Thrane PG, Thim T, Jensen LO, et al. Ten-year cardiovascular risk in diabetes patients without obstructive coronary artery disease: a retrospective Western Denmark cohort study. Cardiovasc Diabetol. 2021;20(1):23.
121. Kremke M, Gissel MS, Jensen MJ, Thomassen SA, Jakobsen CJ. The association between a three-day ticagrelor discontinuation and perioperative bleeding complications. Eur J Cardiothorac Surg. 2019;55(4):714-20.
122. Kremke M, Hansen MK, Christensen S, Tang M, Andreasen JJ, Jakobsen CJ. The association between platelet transfusion and adverse outcomes after coronary artery bypass surgery. Eur J Cardiothorac Surg. 2015;48(5):e102-9.
123. Özcan C, Juel K, Flensted Lassen J, von Kappelgaard LM, Mortensen PE, Gislason G. The Danish Heart Registry. Clin Epidemiol. 2016;8:503-8.
124. Mikkelsen MM, Johnsen SP, Nielsen PH, Jakobsen CJ. The EuroSCORE in western Denmark: a population-based study. J Cardiothorac Vasc Anesth. 2012;26(2):258-64.
125. Bhavsar R, Jakobsen CJ. The Major Decrease in Resource Utilization in Recent Decades Seems Guided by Demographic Changes: Fast Tracking-Real Concept or Demographics. J Cardiothorac Vasc Anesth. 2020;34(6):1476-84.
126. Thayssen P, Jensen LO, Lassen JF, Tilsted HH, Kaltoft A, Christiansen EH, et al. The risk and prognostic impact of definite stent thrombosis or in-stent restenosis after coronary stent implantation. EuroIntervention. 2012;8(5):591-8.
127. Skov JK, Kimose HH, Greisen J, Jakobsen CJ. To jump or not to jump? A multicentre propensity-matched study of sequential vein grafting of the heart†. Interact Cardiovasc Thorac Surg. 2019.
128. Jakobsen CJ, Ryhammer PK, Tang M, Andreasen JJ, Mortensen PE. Transfusion of blood during cardiac surgery is associated with higher long-term mortality in low-risk patients. Eur J Cardiothorac Surg. 2012;42(1):114-20.
129. Munkholm SB, Jakobsen CJ, Mortensen PE, Lundbye-Christensen S, Andreasen JJ. Validation of post-operative atrial fibrillation in the Western Denmark Heart Registry. Dan Med J. 2015;62(12):A5162.
130. Norderud K, Egholm G, Thim T, Olesen KKW, Madsen M, Jensen LO, et al. Validation of the European Society of Cardiology and European Society of Anaesthesiology non-cardiac surgery risk score in patients treated with coronary drug-eluting stent implantation. Eur Heart J Qual Care Clin Outcomes. 2019;5(1):22-7.
131. Nielsen DV, Johnsen SP, Madsen M, Jakobsen CJ. Variation in use of peroperative inotropic support therapy in cardiac surgery: time for reflection? Acta Anaesthesiol Scand. 2011;55(3):352-8.
132. Dudek D, Siudak Z, Legutko J, Araszkiewicz A, Bil J, Dąbrowski M, et al. Percutaneous interventions in cardiology in Poland in the year 2017. Summary report of the Association of Cardiovascular Interventions of the Polish Cardiac Society AISN PTK and Jagiellonian University Medical College. Postepy Kardiol Interwencyjnej. 2018;14(4):422-4.
133. Zasada W, Bobrowska B, Plens K, Dziewierz A, Siudak Z, Surdacki A, et al. Acute myocardial infarction in young patients. Kardiol Pol. 2021;79(10):1093-8.
134. Januszek R, Siudak Z, Malinowski KP, Wojdyła R, Mika P, Wańha W, et al. Aspiration Thrombectomy in Patients with Acute Myocardial Infarction-5-Year Analysis Based on a Large National Registry (ORPKI). J Clin Med. 2020;9(11).

135. Zabojszcz M, Januszek R, Siudak Z, Janion-Sadowska A, Jędrychowska M, Pawlik A, et al. Association between the mortality rate and operator volume in patients undergoing emergency or elective percutaneous coronary interventions. Kardiol Pol. 2020;78(2):138-46.
136. Januszek R, Siudak Z, Dziewierz A, Rakowski T, Legutko J, Dudek D, et al. Bailout rotational atherectomy in patients with myocardial infarction is not associated with an increased periprocedural complication rate or poorer angiographic outcomes in comparison to elective procedures (from the ORPKI Polish National Registry 2015-2016). Postepy Kardiol Interwencyjnej. 2018;14(2):135-43.
137. Rakowski T, De Luca G, Siudak Z, Plens K, Dziewierz A, Kleczyński P, et al. Characteristics of patients presenting with myocardial infarction with non-obstructive coronary arteries (MINOCA) in Poland: data from the ORPKI national registry. J Thromb Thrombolysis. 2019;47(3):462-6.
138. Januszek R, Dziewierz A, Siudak Z, Rakowski T, Dudek D, Bartuś S. Chronic obstructive pulmonary disease and periprocedural complications in patients undergoing percutaneous coronary interventions. PLoS One. 2018;13(10):e0204257.
139. Siudak Z, Grygier M, Wojakowski W, Malinowski KP, Witkowski A, Gąsior M, et al. Clinical and procedural characteristics of COVID-19 patients treated with percutaneous coronary interventions. Catheter Cardiovasc Interv. 2020;96(6):E568-e75.
140. Tokarek T, Siudak Z, Dziewierz A, Rakowski T, Krycińska R, Siwiec A, et al. Clinical outcomes in nonagenarians undergoing a percutaneous coronary intervention: data from the ORPKI Polish National Registry 2014-2016. Coron Artery Dis. 2018;29(7):573-8.
141. Staszczak B, Siudak Z, Malinowski KP, Jędrychowska M, Zabojszcz M, Dolecka-Ślusarczyk M, et al. Clinical outcomes in patients with acute myocardial infarction treated with primary percutaneous coronary intervention stratified according to duration of pain-to-balloon time and type of myocardial infarction. Cardiol J. 2021.
142. Zandecki L, Janion-Sadowska A, Kurzawski J, Piatek L, Zabojszcz M, Plens K, et al. Clinical presentation and 3-year outcomes of patients with acute coronary syndromes and non-obstructive coronary arteries on angiography. PLoS One. 2020;15(6):e0234735.
143. Tokarek T, Dziewierz A, Plens K, Rakowski T, Januszek R, Zabojszcz M, et al. Comparison of safety and effectiveness between the right and left radial artery approach in percutaneous coronary intervention. Rev Esp Cardiol (Engl Ed). 2022;75(2):119-28.
144. Januszek R, Dziewierz A, Siudak Z, Rakowski T, Kameczura T, Tokarek T, et al. Concomitant multi-vessel disease is associated with a lower procedural death rate in patients treated with percutaneous coronary interventions within the left main coronary artery (from the ORPKI registry). Arch Med Sci. 2021;17(4):881-90.
145. Rakowski T, Siudak Z, Dziewierz A, Plens K, Kleczyński P, Dudek D. Contemporary use of P2Y(12) inhibitors in patients with ST-segment elevation myocardial infarction referred to primary percutaneous coronary interventions in Poland: Data from ORPKI national registry. J Thromb Thrombolysis. 2018;45(1):151-7.
146. Januszek R, Siudak Z, Reczuch K, Dobrzycki S, Lesiak M, Legutko J, et al. Current trends and procedural outcomes in the era of rotational atherectomy expansion in Poland in the period 2014-2017 (based on the nationwide ORPKI registry). Postepy Kardiol Interwencyjnej. 2019;15(2):158-66.

147. Dziewierz A, Siudak Z, Tokarek T, Rakowski T, Dudek D. Determinants of stroke following percutaneous coronary intervention in acute myocardial infarction (from ORPKI Polish National Registry). Int J Cardiol. 2016;223:236-8.
148. Januszek RA, Dziewierz A, Siudak Z, Rakowski T, Legutko J, Rzeszutko Ł, et al. Diabetes and periprocedural outcomes in patients treated with rotablation during percutaneous coronary interventions. Cardiol J. 2018;27(2):152-61.
149. Lanocha M, Lansky A, Siudak Z, Włodarczak A, Tarchalski J, Malinowski KP, et al. Does the operator's sex matter? An analysis based on the national interventional cardiology registry. Kardiol Pol. 2020;78(12):1221-6.
150. Januszek R, Siudak Z, Janion-Sadowska A, Jędrychowska M, Staszczak B, Bartuś J, et al. Effect of day- and night-time admissions on long-term clinical outcomes of patients with acute myocardial infarction treated with percutaneous coronary intervention. Pol Arch Intern Med. 2020;130(7-8):570-81.
151. Januszek R, Bryniarski L, Siudak Z, Malinowski KP, Bryniarski KL, Surdacki A, et al. Five-year report from the Polish national registry on percutaneous coronary interventions with a focus on coronary artery perforations within chronic total occlusions. Postepy Kardiol Interwencyjnej. 2020;16(4):399-409.
152. Staszczak B, Malinowski KP, Wańha W, Siudak Z, Jędrychowska M, Susuł M, et al. Frequency and predictors of diagnostic coronary angiography and percutaneous coronary intervention related to stroke. Kardiol Pol. 2021;79(10):1099-106.
153. Terlecki M, Wojciechowska W, Dudek D, Siudak Z, Plens K, Guzik TJ, et al. Impact of acute total occlusion of the culprit artery on outcome in NSTEMI based on the results of a large national registry. BMC Cardiovasc Disord. 2021;21(1):297.
154. Dziewierz A, Brener SJ, Siudak Z, Plens K, Rakowski T, Zasada W, et al. Impact of On-Site Surgical Backup on Periprocedural Outcomes of Primary Percutaneous Interventions in Patients Presenting With ST-Segment Elevation Myocardial Infarction (From the ORPKI Polish National Registry). Am J Cardiol. 2018;122(6):929-35.
155. Piątek Ł, Janion-Sadowska A, Piątek K, Zandecki Ł, Zabojszcz M, Siudak Z, et al. Long-term clinical outcomes in patients with unstable angina undergoing percutaneous coronary interventions in a contemporary registry data from Poland. Coron Artery Dis. 2020;31(3):215-21.
156. Siudak Z, Dziewierz A, Rakowski T, Tokarek T, Mielecki W, Żabówka A, et al. No clinical benefit from manual thrombus aspiration in patients with non-ST-elevation myocardial infarction. Postepy Kardiol Interwencyjnej. 2016;12(1):32-40.
157. Sielski J, Kaziród-Wolski K, Siudak Z. Out-of-hospital cardiac arrest: data from the National Registry of Invasive Cardiology Procedures (ORPKI) in a long-term survival analysis of patients with acute coronary syndromes in a Polish region. Kardiol Pol. 2020;78(5):412-9.
158. Rzeszutko Ł, Tokarek T, Siudak Z, Dziewierz A, Żmudka K, Dudek D. Patient profile and periprocedural outcomes of bioresorbable vascular scaffold implantation in comparison with drug-eluting and bare-metal stent implantation. Experience from ORPKI Polish National Registry 2014-2015. Postepy Kardiol Interwencyjnej. 2016;12(4):321-8.
159. Ochała A, Siudak Z, Legutko J, Parma R, Chmielak Z, Bartuś S, et al. Percutaneous interventions in cardiology in Poland in the year 2014. Summary report of the Association of

Cardiovascular Interventions of the Polish Cardiac Society AISN PTK. Postepy Kardiol Interwencyjnej. 2015;11(3):177-81.
160. Januszek RA, Bryniarski L, Siudak Z, Malinowski KP, Surowiec S, Bryniarski K, et al. Predictors and trends of contrast use and radiation exposure in a large cohort of patients treated with percutaneous coronary interventions: Chronic total occlusion analysis based on a national registry. Cardiol J. 2021.
161. Januszek R, Siudak Z, Dziewierz A, Dudek D, Bartuś S. Predictors of in-hospital effectiveness and complications of rotational atherectomy (from the ORPKI Polish National Registry 2014-2016). Catheter Cardiovasc Interv. 2018;92(4):E278-e87.
162. Januszek RA, Dziewierz A, Siudak Z, Rakowski T, Dudek D, Bartuś S. Predictors of periprocedural complications in patients undergoing percutaneous coronary interventions within coronary artery bypass grafts. Cardiol J. 2019;26(6):633-44.
163. Podolec J, Wiewiórka Ł, Siudak Z, Malinowski K, Dudek D, Gackowski A, et al. Prevalence and clinical presentation of myocardial bridge on the basis of the National Polish Percutaneous Interventions Registry and the Classification of Rare Cardiovascular Diseases. Kardiol Pol. 2018;77(4):465-70.
164. Rakowski T, Węgiel M, Siudak Z, Plens K, Dziewierz A, Birkemeyer R, et al. Prevalence and Predictors of Coronary Artery Perforation During Percutaneous Coronary Interventions (from the ORPKI National Registry in Poland). Am J Cardiol. 2019;124(8):1186-9.
165. Siudak Z, Wysocka-Dubielecka K, Malinowski K, Dziewierz A, Tokarek T, Plens K, et al. Psoriasis is an independent predictor of increased risk of allergic reaction during percutaneous coronary interventions. Big data analysis from the Polish National PCI Registry (ORPKI). Cardiol J. 2020;27(3):278-84.
166. Tokarek T, Dziewierz A, Plens K, Rakowski T, Dudek D, Siudak Z. Radial approach reduces mortality in patients with ST-segment elevation myocardial infarction and cardiogenic shock. Pol Arch Intern Med. 2021;131(5):421-8.
167. Januszek R, Siudak Z, Malinowski KP, Reczuch K, Dobrzycki S, Lesiak M, et al. Radial versus femoral access in patients treated with percutaneous coronary intervention and rotational atherectomy. Kardiol Pol. 2020;78(6):529-36.
168. Siudak Z, Tokarek T, Dziewierz A, Wysocki T, Wiktorowicz A, Legutko J, et al. Reduced periprocedural mortality and bleeding rates of radial approach in ST-segment elevation myocardial infarction. Propensity score analysis of data from the ORPKI Polish National Registry. EuroIntervention. 2017;13(7):843-50.
169. Sielski J, Kaziród-Wolski K, Siudak Z. Risk of perioperative death and sudden cardiac arrest: A study of 113 456 cases from the National Registry of Invasive Cardiology Procedures (ORPKI) for estimation of the perioperative prognosis. Kardiol Pol. 2021;79(12):1328-34.
170. Sabatowski K, Malinowski KP, Siudak Z, Reczuch K, Dobrzycki S, Lesiak M, et al. Sex-related differences and rotational atherectomy: Analysis of 5 177 percutaneous coronary interventions based on a large national registry from 2014 to 2020. Kardiol Pol. 2021;79(12):1320-7.

171. Siudak Z, Ochała A, Lesiak M, Witkowski A, Gil RJ, Legutko J, et al. Temporal trends and patterns in percutaneous treatment of coronary artery disease in Poland in the years 2005-2011. Kardiol Pol. 2015;73(7):485-92.
172. Dziewierz A, Siudak Z, Rakowski T, Zasada W, Legutko J, Żabówka A, et al. The impact of multiple stent implantation in the infarct-related artery on one-year clinical outcomes of patients with ST-elevation myocardial infarction undergoing primary percutaneous coronary intervention. Data from the Polish NRDES Registry. Kardiol Pol. 2016;74(8):717-25.
173. Kleczyński P, Siudak Z, Dziewierz A, Tokarek T, Rakowski T, Legutko J, et al. The network of invasive cardiology facilities in Poland in 2016 (data from the ORPKI Polish National Registry). Kardiol Pol. 2018;76(4):805-7.
174. Januszek R, Siudak Z, Malinowski KP, Wańha W, Wojakowski W, Gąsior M, et al. Transradial and Transfemoral Approach in Patients with Prior Coronary Artery Bypass Grafting. J Clin Med. 2020;9(3).
175. Tokarek T, Dziewierz A, Malinowski KP, Rakowski T, Bartuś S, Dudek D, et al. Treatment Delay and Clinical Outcomes in Patients with ST-Segment Elevation Myocardial Infarction during the COVID-19 Pandemic. J Clin Med. 2021;10(17).
176. Rzeszutko Ł, Siudak Z, Tokarek T, Plens K, Włodarczak A, Lekston A, et al. Twelve months clinical outcome after bioresorbable vascular scaffold implantation in patients with stable angina and acute coronary syndrome. Data from the Polish National Registry. Postepy Kardiol Interwencyjnej. 2016;12(2):108-15.
177. Rzeszutko Ł, Siudak Z, Włodarczak A, Lekston A, Depukat R, Ochała A, et al. Use of bioresorbable vascular scaffolds in patients with stable angina and acute coronary syndromes. Polish National Registry. Kardiol Pol. 2014;72(12):1394-9.
178. Siudak Z, Dziewierz A, Rakowski T, Żmudka K, Legutko J, Bartuś S, et al. Borderline trend towards long-term mortality benefit from drug eluting stents implantation in ST-elevation myocardial infarction patients in Poland-data from NRDES registry. Catheter Cardiovasc Interv. 2014;83(3):436-42.
179. SWEDEHEART. Methodological remarks. https://www.ucr.uu.se/swedeheart/index.php?option=com_content&view=article&id=220&Itemid=538 (accessed March 21. 2022)
180. Swedish Coronary Angiography and Angioplasty Registry (SCAAR). Different stents in SCAAR. https://www.ucr.uu.se/swedeheart/forskning-scaar/in-english/stent-reports (accessed March 21. 2022)
181. Bestehorn K, Bestehorn M, Fleck E. Influence of different approaches of aortic valve replacement on the incidence of post-operative delirium in intermediate risk patients - a matched pair analysis. Curr Med Res Opin. 2015;31(12):2157-63.
182. Eggebrecht H, Bestehorn K, Rassaf T, Bestehorn M, Voigtländer T, Fleck E, et al. In-hospital outcomes after transcatheter or surgical aortic valve replacement in younger patients less than 75 years old: a propensity-matched comparison. EuroIntervention. 2018;14(1):50-7.
183. Eggebrecht H, Bestehorn M, Haude M, Schmermund A, Bestehorn K, Voigtländer T, et al. Outcomes of transfemoral transcatheter aortic valve implantation at hospitals with and without on-site cardiac surgery department: insights from the prospective German aortic

valve replacement quality assurance registry (AQUA) in 17 919 patients. Eur Heart J. 2016;37(28):2240-8.
184. Eggebrecht H, Mehta RH. Transcatheter aortic valve implantation (TAVI) in Germany 2008-2014: on its way to standard therapy for aortic valve stenosis in the elderly? EuroIntervention. 2016;11(9):1029-33.
185. Egger F, Zweiker D, Freynhofer MK, Löffler V, Rohla M, Geppert A, et al. Impact of On-Site Cardiac Surgery on Clinical Outcomes After Transfemoral Transcatheter Aortic Valve Replacement. JACC Cardiovasc Interv. 2018;11(21):2160-7.
186. Bosmans JM, Kefer J, De Bruyne B, Herijgers P, Dubois C, Legrand V, et al. Procedural, 30-day and one year outcome following CoreValve or Edwards transcatheter aortic valve implantation: results of the Belgian national registry. Interact Cardiovasc Thorac Surg. 2011;12(5):762-7.
187. Van Gestel R, Müller T, Bosmans J. Does my high blood pressure improve your survival? Overall and subgroup learning curves in health. Health Econ. 2017;26(9):1094-109.
188. Delta cardio. Focus op aortaklep interventie. 18 November 2017. Available: https://www.azdelta.be/sites/default/files/deltacardio_brochure_focus_op_aortaklepinterventie_lr_deel1.pdf
189. Branny M, Branny P, Januska J, Hudec M, Kluzova K, Kufova P, et al. Kratkodobe klinicke vysledky nemocnych po katetrove implantaci aortalni chlopne: TAVI registr Kardiocentra Nemocnice Podlesi Trinec. Interventional Cardiology. 2014;13(4):177-81.
190. Bláha M, Kala P, Klimes D, Jarkovský J, Branny M, !aeásek J, et al., editors. Czech TAVI Registry - Technical background of the multicentre parametric data collection | Czech TAVI Registry - Technické zajiau ní multicentrického sc ru parametrických dat2014.
191. Kala P, Němec P, Malík P, Ondrášek J, Třetina M, Pokorný P, et al. [The role of learning curve on the results of aortic valve implantation for severe aortic stenosis]. Vnitr Lek. 2014;60(4):316-21.
192. Fridrich V. [Czech TAVI Registry. Do we need registeries?]. Vnitr Lek. 2014;60(10):817-21.
193. Bláha M, Kala P, Klimeš D, Bernat I, Branny M, Cervinka P, et al. [The user´s reporting from the national registry of catheter aortic valve implantations (Czech TAVI Registry): the possibilities of the analytical reports based on the database system TrialDB2]. Vnitr Lek. 2014;60(10):837-45.
194. Kala P, Blaha M. Czech TAVI Registry—rationale and design. Cor et Vasa. 2012;54(3):e143-e50.
195. Biancari F, Dahlbacka S, Juvonen T, Virtanen MPO, Maaranen P, Jaakkola J, et al. Favorable outcome of cancer patients undergoing transcatheter aortic valve replacement. Int J Cardiol. 2020;315:86-9.
196. Mäkikallio T, Jalava MP, Husso A, Virtanen M, Laakso T, Ahvenvaara T, et al. Ten-year experience with transcatheter and surgical aortic valve replacement in Finland. Ann Med. 2019;51(3-4):270-9.
197. Biancari F, Valtola A, Juvonen T, Husso A, Dahlbacka S, Laakso T, et al. Trifecta Versus Perimount Magna Ease Aortic Valve Prostheses. Ann Thorac Surg. 2020;110(3):879-88.

198. Beurtheret S, Karam N, Resseguier N, Houel R, Modine T, Folliguet T, et al. Femoral Versus Nonfemoral Peripheral Access for Transcatheter Aortic Valve Replacement. J Am Coll Cardiol. 2019;74(22):2728-39.
199. Durand E, Avinée G, Gillibert A, Tron C, Bettinger N, Bouhzam N, et al. Analysis of length of stay after transfemoral transcatheter aortic valve replacement: results from the FRANCE TAVI registry. Clin Res Cardiol. 2021;110(1):40-9.
200. Van Belle E, Vincent F, Labreuche J, Auffret V, Debry N, Lefèvre T, et al. Balloon-Expandable Versus Self-Expanding Transcatheter Aortic Valve Replacement: A Propensity-Matched Comparison From the FRANCE-TAVI Registry. Circulation. 2020;141(4):243-59.
201. Bière L, Durfort A, Fouquet O, Hamel JF, Leprince P, Chevreul K, et al. Baseline characteristics and outcomes after transcatheter aortic-valve implantation in patients with or without previous balloon aortic valvuloplasty: Insights from the FRANCE 2 registry. Arch Cardiovasc Dis. 2017;110(10):534-42.
202. Chopard R, Teiger E, Meneveau N, Chocron S, Gilard M, Laskar M, et al. Baseline Characteristics and Prognostic Implications of Pre-Existing and New-Onset Atrial Fibrillation After Transcatheter Aortic Valve Implantation: Results From the FRANCE-2 Registry. JACC Cardiovasc Interv. 2015;8(10):1346-55.
203. Folliguet TA, Teiger E, Beurtheret S, Modine T, Lefevre T, Van Belle E, et al. Carotid versus femoral access for transcatheter aortic valve implantation: a propensity score inverse probability weighting study. Eur J Cardiothorac Surg. 2019;56(6):1140-6.
204. Oguri A, Yamamoto M, Mouillet G, Gilard M, Laskar M, Eltchaninoff H, et al. Clinical outcomes and safety of transfemoral aortic valve implantation under general versus local anesthesia: subanalysis of the French Aortic National CoreValve and Edwards 2 registry. Circ Cardiovasc Interv. 2014;7(4):602-10.
205. Castellant P, Didier R, Bezon E, Couturaud F, Eltchaninoff H, Iung B, et al. Comparison of Outcome of Transcatheter Aortic Valve Implantation With Versus Without Previous Coronary Artery Bypass Grafting (from the FRANCE 2 Registry). Am J Cardiol. 2015;116(3):420-5.
206. Chevreul K, Brunn M, Cadier B, Haour G, Eltchaninoff H, Prat A, et al. Cost of transcatheter aortic valve implantation and factors associated with higher hospital stay cost in patients of the FRANCE (FRench Aortic National CoreValve and Edwards) registry. Arch Cardiovasc Dis. 2013;106(4):209-19.
207. Yamamoto M, Mouillet G, Oguri A, Gilard M, Laskar M, Eltchaninoff H, et al. Effect of body mass index on 30- and 365-day complication and survival rates of transcatheter aortic valve implantation (from the FRench Aortic National CoreValve and Edwards 2 [FRANCE 2] registry). Am J Cardiol. 2013;112(12):1932-7.
208. Didier R, Benic C, Nasr B, Le Ven F, Hannachi S, Eltchaninoff H, et al. High Post-Procedural Transvalvular Gradient or Delayed Mean Gradient Increase after Transcatheter Aortic Valve Implantation: Incidence, Prognosis and Associated Variables. The FRANCE-2 Registry. J Clin Med. 2021;10(15).
209. Oguri A, Yamamoto M, Mouillet G, Gilard M, Laskar M, Eltchaninoff H, et al. Impact of chronic kidney disease on the outcomes of transcatheter aortic valve implantation: results from the FRANCE 2 registry. EuroIntervention. 2015;10(9):e1-9.

210. Chopard R, Meneveau N, Chocron S, Gilard M, Laskar M, Eltchaninoff H, et al. Impact of chronic obstructive pulmonary disease on Valve Academic Research Consortium-defined outcomes after transcatheter aortic valve implantation (from the FRANCE 2 Registry). Am J Cardiol. 2014;113(9):1543-9.
211. Deharo P, Jaussaud N, Grisoli D, Camus O, Resseguier N, Le Breton H, et al. Impact of Direct Transcatheter Aortic Valve Replacement Without Balloon Aortic Valvuloplasty on Procedural and Clinical Outcomes: Insights From the FRANCE TAVI Registry. JACC Cardiovasc Interv. 2018;11(19):1956-65.
212. Amabile N, Agostini H, Gilard M, Eltchaninoff H, Iung B, Donzeau-Gouge P, et al. Impact of low preprocedural transvalvular gradient on cardiovascular mortality following TAVI: an analysis from the FRANCE 2 registry. EuroIntervention. 2014;10(7):842-9.
213. Arai T, Morice MC, O'Connor SA, Yamamoto M, Eltchaninoff H, Leguerrier A, et al. Impact of pre- and post-procedural anemia on the incidence of acute kidney injury and 1-year mortality in patients undergoing transcatheter aortic valve implantation (from the French Aortic National CoreValve and Edwards 2 [FRANCE 2] Registry). Catheter Cardiovasc Interv. 2015;85(7):1231-9.
214. Furuta A, Lellouche N, Mouillet G, Gilard M, Laskar M, Eltchaninoff H, et al. Impact of previous acute pulmonary oedema after transcatheter aortic valve implantation: insight from French Aortic National CoreValve and Edwards 2 [FRANCE 2]registry. Int J Cardiol. 2015;183:98-104.
215. Overtchouk P, Guedeney P, Rouanet S, Verhoye JP, Lefevre T, Van Belle E, et al. Long-Term Mortality and Early Valve Dysfunction According to Anticoagulation Use: The FRANCE TAVI Registry. J Am Coll Cardiol. 2019;73(1):13-21.
216. Mouillet G, Lellouche N, Yamamoto M, Oguri A, Dubois-Rande JL, Van Belle E, et al. Outcomes following pacemaker implantation after transcatheter aortic valve implantation with CoreValve(®) devices: Results from the FRANCE 2 Registry. Catheter Cardiovasc Interv. 2015;86(3):E158-66.
217. Benamer H, Auffret V, Cayla G, Chevalier B, Dupouy P, Eltchaninoff H, et al. Position paper of French Interventional Group (GACI) for TAVI in France in 2018. Ann Cardiol Angeiol (Paris). 2018;67(6):455-65.
218. Iung B, Laouénan C, Himbert D, Eltchaninoff H, Chevreul K, Donzeau-Gouge P, et al. Predictive factors of early mortality after transcatheter aortic valve implantation: individual risk assessment using a simple score. Heart. 2014;100(13):1016-23.
219. Furuta A, Lellouche N, Mouillet G, Dhanjal T, Gilard M, Laskar M, et al. Prognostic value of new onset atrial fibrillation after transcatheter aortic valve implantation: A FRANCE 2 registry substudy. Int J Cardiol. 2016;210:72-9.
220. Gilard M, Eltchaninoff H, Iung B, Donzeau-Gouge P, Chevreul K, Fajadet J, et al. Registry of transcatheter aortic-valve implantation in high-risk patients. N Engl J Med. 2012;366(18):1705-15.
221. Didier R, Gouysse M, Eltchaninoff H, Le Breton H, Commeau P, Cayla G, et al. Successful linkage of French large-scale national registry populations to national reimbursement data: Improved data completeness and minimized loss to follow-up. Arch Cardiovasc Dis. 2020;113(8-9):534-41.

222. Didier R, Lhermusier T, Auffret V, Eltchaninoff H, Le Breton H, Cayla G, et al. TAVR Patients Requiring Anticoagulation: Direct Oral Anticoagulant or Vitamin K Antagonist? JACC Cardiovasc Interv. 2021;14(15):1704-13.
223. Spaziano M, Lefèvre T, Romano M, Eltchaninoff H, Leprince P, Motreff P, et al. Transcatheter Aortic Valve Replacement in the Catheterization Laboratory Versus Hybrid Operating Room: Insights From the FRANCE TAVI Registry. JACC Cardiovasc Interv. 2018;11(21):2195-203.
224. Husser O, Fujita B, Hengstenberg C, Frerker C, Beckmann A, Möllmann H, et al. Conscious Sedation Versus General Anesthesia in Transcatheter Aortic Valve Replacement: The German Aortic Valve Registry. JACC Cardiovasc Interv. 2018;11(6):567-78.
225. Holzhey D, Mohr FW, Walther T, Möllmann H, Beckmann A, Kötting J, et al. Current Results of Surgical Aortic Valve Replacement: Insights From the German Aortic Valve Registry. Ann Thorac Surg. 2016;101(2):658-66.
226. Paradis JM, Rodés-Cabau J. Expanding the Transcatheter Aortic Valve Replacement Field Under Continued Surveillance: The GARY Registry. J Am Coll Cardiol. 2015;65(20):2181-3.
227. Hamm CW, Beyersdorf F. GARY-The Largest Registry of Aortic Stenosis Treatment Worldwide. Eur Heart J. 2020;41(6):733-5.
228. Mas-Peiro S, Faerber G, Bon D, Herrmann E, Bauer T, Bleiziffer S, et al. Impact of chronic kidney disease in 29 893 patients undergoing transcatheter or surgical aortic valve replacement from the German Aortic Valve Registry. Eur J Cardiothorac Surg. 2021;59(3):532-44.
229. Fujita B, Schmidt T, Bleiziffer S, Bauer T, Beckmann A, Bekeredjian R, et al. Impact of new pacemaker implantation following surgical and transcatheter aortic valve replacement on 1-year outcome. Eur J Cardiothorac Surg. 2020;57(1):151-9.
230. Bauer T, Möllmann H, Beckmann A, Ensminger S, Frerker C, Holzhey D, et al. Left ventricular function determines the survival benefit for women over men after transcatheter aortic valve implantation (TAVI). EuroIntervention. 2017;13(4):467-74.
231. Werner N, Zahn R, Beckmann A, Bauer T, Bleiziffer S, Hamm CW, et al. Patients at Intermediate Surgical Risk Undergoing Isolated Interventional or Surgical Aortic Valve Implantation for Severe Symptomatic Aortic Valve Stenosis. Circulation. 2018;138(23):2611-23.
232. Bekeredjian R, Szabo G, Balaban Ü, Bleiziffer S, Bauer T, Ensminger S, et al. Patients at low surgical risk as defined by the Society of Thoracic Surgeons Score undergoing isolated interventional or surgical aortic valve implantation: in-hospital data and 1-year results from the German Aortic Valve Registry (GARY). Eur Heart J. 2019;40(17):1323-30.
233. Walther T, Hamm CW, Schuler G, Berkowitsch A, Kötting J, Mangner N, et al. Perioperative Results and Complications in 15,964 Transcatheter Aortic Valve Replacements: Prospective Data From the GARY Registry. J Am Coll Cardiol. 2015;65(20):2173-80.
234. Voigtländer L, Twerenbold R, Schäfer U, Conradi L, Balaban Ü, Bekeredjian R, et al. Prognostic Impact of Underweight (Body Mass Index <20 kg/m(2)) in Patients With Severe Aortic Valve Stenosis Undergoing Transcatheter Aortic Valve Implantation or Surgical Aortic Valve Replacement (from the German Aortic Valve Registry [GARY]). Am J Cardiol. 2020;129:79-86.

235. Lange R, Beckmann A, Neumann T, Krane M, Deutsch MA, Landwehr S, et al. Quality of Life After Transcatheter Aortic Valve Replacement: Prospective Data From GARY (German Aortic Valve Registry). JACC Cardiovasc Interv. 2016;9(24):2541-54.
236. Ensminger S, Fujita B, Bauer T, Möllmann H, Beckmann A, Bekeredjian R, et al. Rapid Deployment Versus Conventional Bioprosthetic Valve Replacement for Aortic Stenosis. J Am Coll Cardiol. 2018;71(13):1417-28.
237. Hamm CW, Möllmann H, Holzhey D, Beckmann A, Veit C, Figulla HR, et al. The German Aortic Valve Registry (GARY): in-hospital outcome. Eur Heart J. 2014;35(24):1588-98.
238. Mohr FW, Holzhey D, Möllmann H, Beckmann A, Veit C, Figulla HR, et al. The German Aortic Valve Registry: 1-year results from 13,680 patients with aortic valve disease. Eur J Cardiothorac Surg. 2014;46(5):808-16.
239. Blumenstein J, Möllmann H, Bleiziffer S, Bauer T, Ensminger S, Bekeredjian R, et al. Transcatheter aortic valve implantation in nonagenarians: insights from the German Aortic Valve Registry (GARY). Clin Res Cardiol. 2020;109(9):1099-106.
240. Färber G, Bleiziffer S, Doenst T, Bon D, Böning A, Weiler H, et al. Transcatheter or surgical aortic valve implantation in chronic dialysis patients: a German Aortic Valve Registry analysis. Clin Res Cardiol. 2021;110(3):357-67.
241. Fujita B, Ensminger S, Bauer T, Möllmann H, Beckmann A, Bekeredjian R, et al. Trends in practice and outcomes from 2011 to 2015 for surgical aortic valve replacement: an update from the German Aortic Valve Registry on 42 776 patients. Eur J Cardiothorac Surg. 2018;53(3):552-9.
242. Hamm CW, Mohr F, Heusch G. Lessons Learned From the German Aortic Valve Registry. J Am Coll Cardiol. 2018;72(6):689-92.
243. Zembala M, Wilczek K, Przybylski R, Chodór P, Nadziakiewicz P, Krasoń M, et al. [POL-TAVI First--Polish report on transcatheter aortic valve implantation (TAVI) of Edwards-Sapien prosthesis in the first 19 high risk patients with severe aortic stenosis and comorbidities]. Kardiol Pol. 2009;67(8):936-40.
244. Navarese EP, Zhang Z, Kubica J, Andreotti F, Farinaccio A, Bartorelli AL, et al. Development and Validation of a Practical Model to Identify Patients at Risk of Bleeding After TAVR. JACC Cardiovasc Interv. 2021;14(11):1196-206.
245. Stańska A, Jagielak D, Brzeziński M, Zembala M, Wojakowski W, Kochman J, et al. Improvement of quality of life following transcatheter aortic valve implantation in the elderly: a multi-centre study based on the Polish national TAVI registry. Kardiol Pol. 2017;75(1):13-20.
246. Kleczyński P, Zasada W, Bagieński M, Rzeszutko Ł, Sorysz D, Sobczyński R, et al. Paravalvular leak after transcatheter aortic valve implantation (TAVI): Short-term results. Data from Polish national POL-TAVI registry. Cardiol J. 2016;23(2):163-8.
247. Walas RL, Kukulski L, Rychter J, Jaźwiec T, Gąska M, Hawranek M, et al. Vascular access site complications after transfemoral transcatheter aortic valve implantation in the POL-TAVI Registry: surgical versus percutaneous approach. J Cardiovasc Surg (Torino). 2020;61(1):117-22.
248. Huczek Z, Zbroński K, Grodecki K, Scisło P, Rymuza B, Kochman J, et al. Concomitant coronary artery disease and its management in patients referred to transcatheter

aortic valve implantation: Insights from the POL-TAVI Registry. Catheter Cardiovasc Interv. 2018;91(1):115-23.
249. Grodecki K, Tamarappoo BK, Huczek Z, Jedrzejczyk S, Cadet S, Kwiecinski J, et al. Non-calcific aortic tissue quantified from computed tomography angiography improves diagnosis and prognostication of patients referred for transcatheter aortic valve implantation. Eur Heart J Cardiovasc Imaging. 2021;22(6):626-35.
250. Huczek Z, Grodecki K, Scisło P, Wilczek K, Jagielak D, Fil W, et al. Transcatheter aortic valve-in-valve implantation in failed stentless bioprostheses. J Interv Cardiol. 2018;31(6):861-9.
251. Szymański P, Hryniewiecki T, Dąbrowski M, Sorysz D, Kochman J, Jastrzębski J, et al. Mitral and aortic regurgitation following transcatheter aortic valve replacement. Heart. 2016;102(9):701-6.
252. Swiss TAVI. https://www.swisstavi.ch/ (accessed February 28. 2023)
253. Attinger-Toller A, Ferrari E, Tueller D, Templin C, Muller O, Nietlispach F, et al. Age-Related Outcomes After Transcatheter Aortic Valve Replacement: Insights From the SwissTAVI Registry. JACC Cardiovasc Interv. 2021;14(9):952-60.
254. Langfritz M, Shahin M, Nietlispach F, Taramasso M, Denegri A, Moccetti M, et al. Baseline Predictors of Renal Failure in Transcatheter Aortic Valve Implantation. J Invasive Cardiol. 2019;31(10):E289-e97.
255. Stortecky S, Windecker S, Pilgrim T, Heg D, Buellesfeld L, Khattab AA, et al. Cerebrovascular accidents complicating transcatheter aortic valve implantation: frequency, timing and impact on outcomes. EuroIntervention. 2012;8(1):62-70.
256. Okuno T, Lanz J, Stortecky S, Heg D, Bernhard B, Gräni C, et al. Clinical impact of left atrial appendage filling defects in patients undergoing transcatheter aortic valve implantation. Eur Heart J Cardiovasc Imaging. 2021.
257. Asami M, Bernhard B, Demirel C, Okuno T, Stortecky S, Heg D, et al. Clinical outcomes following transcatheter aortic valve implantation in patients with porcelain aorta. J Cardiovasc Comput Tomogr. 2021.
258. Stefanini GG, Stortecky S, Cao D, Rat-Wirtzler J, O'Sullivan CJ, Gloekler S, et al. Coronary artery disease severity and aortic stenosis: clinical outcomes according to SYNTAX score in patients undergoing transcatheter aortic valve implantation. Eur Heart J. 2014;35(37):2530-40.
259. Sweda R, Dobner S, Heg D, Lanz J, Malebranche D, Langhammer B, et al. Discharge Location and Outcomes After Transcatheter Aortic Valve Implantation. Am J Cardiol. 2021;140:95-102.
260. Vontobel J, Possner M, Schütz P, Müller B, Taramasso M, Binder RK, et al. Early safety outcome following transcatheter aortic valve implantation: is the amount of contrast media used a matter of concern? Swiss Med Wkly. 2015;145:w14238.
261. Külling M, Külling J, Wyss C, Hürlimann D, Reho I, Salzberg S, et al. Effective orifice area and hemodynamic performance of the transcatheter Edwards Sapien 3 prosthesis: short-term and 1-year follow-up. Eur Heart J Cardiovasc Imaging. 2018;19(1):23-30.
262. Franzone A, Stortecky S, Räber L, Heg D, Yamaji K, Piccolo R, et al. Effects of coronary artery disease in patients undergoing transcatheter aortic valve implantation: A study of age- and gender-matched cohorts. Int J Cardiol. 2017;243:150-5.

263. Okuno T, Heg D, Lanz J, Praz F, Gräni C, Langhammer B, et al. Heart valve sizing and clinical outcomes in patients undergoing transcatheter aortic valve implantation. Catheter Cardiovasc Interv. 2021;98(5):E768-e79.
264. Wenaweser P, Pilgrim T, Guerios E, Stortecky S, Huber C, Khattab AA, et al. Impact of coronary artery disease and percutaneous coronary intervention on outcomes in patients with severe aortic stenosis undergoing transcatheter aortic valve implantation. EuroIntervention. 2011;7(5):541-8.
265. Yousif N, Obeid S, Binder R, Denegri A, Shahin M, Templin C, et al. Impact of gender on outcomes after transcatheter aortic valve implantation. J Geriatr Cardiol. 2018;15(6):394-400.
266. Okuno T, Asami M, Heg D, Lanz J, Praz F, Hagemeyer D, et al. Impact of Left Ventricular Outflow Tract Calcification on Procedural Outcomes After Transcatheter Aortic Valve Replacement. JACC Cardiovasc Interv. 2020;13(15):1789-99.
267. Franzone A, Stortecky S, Pilgrim T, Asami M, Lanz J, Heg D, et al. Incidence and impact of renal dysfunction on clinical outcomes after transcatheter aortic valve implantation. Int J Cardiol. 2018;250:73-9.
268. Eberhard M, Hinzpeter R, Schönenberger ALN, Euler A, Kuzo N, Reeve K, et al. Incremental Prognostic Value of Coronary Artery Calcium Score for Predicting All-Cause Mortality after Transcatheter Aortic Valve Replacement. Radiology. 2021;301(1):105-12.
269. Stortecky S, Heg D, Tueller D, Pilgrim T, Muller O, Noble S, et al. Infective Endocarditis After Transcatheter Aortic Valve Replacement. J Am Coll Cardiol. 2020;75(24):3020-30.
270. Muller O, Fournier S, Pilgrim T, Heg D, Noble S, Jeger R, et al. Local Versus General Anesthesia for Transcatheter Aortic Valve Replacement: A SwissTAVI Registry Analysis. JACC Cardiovasc Interv. 2019;12(18):1874-6.
271. Pilgrim T, Englberger L, Rothenbühler M, Stortecky S, Ceylan O, O'Sullivan CJ, et al. Long-term outcome of elderly patients with severe aortic stenosis as a function of treatment modality. Heart. 2015;101(1):30-6.
272. Vollenbroich R, Wenaweser P, Macht A, Stortecky S, Praz F, Rothenbühler M, et al. Long-term outcomes with balloon-expandable and self-expandable prostheses in patients undergoing transfemoral transcatheter aortic valve implantation for severe aortic stenosis. Int J Cardiol. 2019;290:45-51.
273. Elchinova E, Nozica N, Bartkowiak J, Ryffel C, Bernhard B, Elsmaan M, et al. Permanent pacemaker implantation late after transcatheter aortic valve implantation. Heart Rhythm. 2021;18(12):2033-9.
274. Koskinas KC, Stortecky S, Franzone A, O'Sullivan CJ, Praz F, Zuk K, et al. Post-Procedural Troponin Elevation and Clinical Outcomes Following Transcatheter Aortic Valve Implantation. J Am Heart Assoc. 2016;5(2).
275. Tomii D, Okuno T, Praz F, Heg D, Wild MG, Lanz J, et al. Potential Candidates for Transcatheter Tricuspid Valve Intervention After Transcatheter Aortic Valve Replacement: Predictors and Prognosis. JACC Cardiovasc Interv. 2021;14(20):2246-56.
276. Eberhard M, Milanese G, Ho M, Zimmermann S, Frauenfelder T, Nietlispach F, et al. Pre-procedural CT angiography inferior vena cava measurements: a predictor of mortality in patients undergoing transcatheter aortic valve implantation. Eur Radiol. 2019;29(2):975-84.

277. Franzone A, O'Sullivan CJ, Stortecky S, Heg D, Lanz J, Vollenbroich R, et al. Prognostic impact of invasive haemodynamic measurements in combination with clinical and echocardiographic characteristics on two-year clinical outcomes of patients undergoing transcatheter aortic valve implantation. EuroIntervention. 2017;12(18):e2186-e93.
278. Asami M, Stortecky S, Praz F, Lanz J, Räber L, Franzone A, et al. Prognostic Value of Right Ventricular Dysfunction on Clinical Outcomes After Transcatheter Aortic Valve Replacement. JACC Cardiovasc Imaging. 2019;12(4):577-87.
279. Franzone A, Pilgrim T, Arnold N, Heg D, Langhammer B, Piccolo R, et al. Rates and predictors of hospital readmission after transcatheter aortic valve implantation. Eur Heart J. 2017;38(28):2211-7.
280. Okuno T, Heg D, Lanz J, Praz F, Brugger N, Stortecky S, et al. Refined staging classification of cardiac damage associated with aortic stenosis and outcomes after transcatheter aortic valve implantation. Eur Heart J Qual Care Clin Outcomes. 2021;7(6):532-41.
281. Brinkert M, Wolfrum M, Moccetti F, Bossard M, Berte B, Cuculi F, et al. Relevance of New Conduction Disorders After Implantation of the ACURATE Neo Transcatheter Heart Valve in the Aortic Valve Position. Am J Cardiol. 2020;125(5):783-7.
282. Wenaweser P, Stortecky S, Heg D, Tueller D, Nietlispach F, Falk V, et al. Short-term clinical outcomes among patients undergoing transcatheter aortic valve implantation in Switzerland: the Swiss TAVI registry. EuroIntervention. 2014;10(8):982-9.
283. Okuno T, Heg D, Lanz J, Stortecky S, Praz F, Windecker S, et al. Staging cardiac damage associated with aortic stenosis in patients undergoing transcatheter aortic valve implantation. Int J Cardiol Heart Vasc. 2021;33:100768.
284. Bernhard B, Okuno T, Cicovic A, Stortecky S, Reichlin T, Lanz J, et al. Systemic corticosteroid exposure and atrioventricular conductance delays after transcatheter aortic valve implantation. Cardiovasc Revasc Med. 2021.
285. Stortecky S, Franzone A, Heg D, Tueller D, Noble S, Pilgrim T, et al. Temporal trends in adoption and outcomes of transcatheter aortic valve implantation: a SwissTAVI Registry analysis. Eur Heart J Qual Care Clin Outcomes. 2019;5(3):242-51.
286. Ferrari E, Stortecky S, Heg D, Muller O, Nietlispach F, Tueller D, et al. The hospital results and 1-year outcomes of transcatheter aortic valve-in-valve procedures and transcatheter aortic valve implantations in the native valves: the results from the Swiss-TAVI Registry. Eur J Cardiothorac Surg. 2019;56(1):55-63.
287. Vollenbroich R, Stortecky S, Praz F, Lanz J, Franzone A, Zuk K, et al. The impact of functional vs degenerative mitral regurgitation on clinical outcomes among patients undergoing transcatheter aortic valve implantation. Am Heart J. 2017;184:71-80.
288. Franzone A, Pilgrim T, Haynes AG, Lanz J, Asami M, Praz F, et al. Transcatheter aortic valve thrombosis: incidence, clinical presentation and long-term outcomes. Eur Heart J Cardiovasc Imaging. 2018;19(4):398-404.
289. Ferrari E, Eeckhout E, Keller S, Muller O, Tozzi P, Berdajs D, et al. Transfemoral versus transapical approach for transcatheter aortic valve implantation: hospital outcome and risk factor analysis. J Cardiothorac Surg. 2017;12(1):78.
290. Stortecky S, Stefanini GG, Pilgrim T, Heg D, Praz F, Luterbacher F, et al. Validation of the Valve Academic Research Consortium Bleeding Definition in Patients With Severe

Aortic Stenosis Undergoing Transcatheter Aortic Valve Implantation. J Am Heart Assoc. 2015;4(10):e002135.
291. Okuno T, Hagemeyer D, Brugger N, Ryffel C, Heg D, Lanz J, et al. Valvular and Nonvalvular Atrial Fibrillation in Patients Undergoing Transcatheter Aortic Valve Replacement. JACC Cardiovasc Interv. 2020;13(18):2124-33.
292. Buccheri S, Sarno G, Lagerqvist B, Olivecrona G, Hambraeus K, Witt N, et al. Bioabsorbable polymer everolimus-eluting stents in patients with acute myocardial infarction: a report from the Swedish Coronary Angiography and Angioplasty Registry. EuroIntervention. 2018;14(5):e562-e9.
293. Buccheri S, James S, Lindholm D, Fröbert O, Olivecrona GK, Persson J, et al. Clinical and angiographic outcomes of bioabsorbable vs. permanent polymer drug-eluting stents in Sweden: a report from the Swedish Coronary and Angioplasty Registry (SCAAR). Eur Heart J. 2019;40(31):2607-15.
294. Buccheri S, Sarno G, Erlinge D, Renlund H, Lagerqvist B, Grimfjärd P, et al. Clinical outcomes with unselected use of an ultrathin-strut sirolimus-eluting stent: a report from the Swedish Coronary Angiography and Angioplasty Registry (SCAAR). EuroIntervention. 2021;16(17):1413-21.
295. Schwalm T, Carlsson J, Meissner A, Lagerqvist B, James S. Current treatment and outcome of coronary in-stent restenosis in Sweden: a report from the Swedish Coronary Angiography and Angioplasty Registry (SCAAR). EuroIntervention. 2013;9(5):564-72.
296. Sarno G, Lagerqvist B, Carlsson J, Olivecrona G, Nilsson J, Calais F, et al. Initial clinical experience with an everolimus eluting platinum chromium stent (Promus Element) in unselected patients from the Swedish Coronary Angiography and Angioplasty Registry (SCAAR). Int J Cardiol. 2013;167(1):146-50.
297. Fröbert O, Scherstén F, James SK, Carlsson J, Lagerqvist B. Long-term safety and efficacy of drug-eluting and bare metal stents in saphenous vein grafts. Am Heart J. 2012;164(1):87-93.
298. Sarno G, Lagerqvist B, Fröbert O, Nilsson J, Olivecrona G, Omerovic E, et al. Lower risk of stent thrombosis and restenosis with unrestricted use of 'new-generation' drug-eluting stents: a report from the nationwide Swedish Coronary Angiography and Angioplasty Registry (SCAAR). Eur Heart J. 2012;33(5):606-13.
299. Grimfjärd P, Bergman E, Buccheri S, Erlinge D, Lagerqvist B, Svennblad B, et al. Outcome of PCI with Xience versus other commonly used modern drug eluting stents: A SCAAR report. Catheter Cardiovasc Interv. 2021;98(2):E197-e204.
300. Grimfjärd P, James S, Persson J, Angerås O, Koul S, Omerovic E, et al. Outcome of percutaneous coronary intervention with the Absorb bioresorbable scaffold: data from the Swedish Coronary Angiography and Angioplasty Registry (SCAAR). EuroIntervention. 2017;13(11):1303-10.
301. Silverio A, Buccheri S, Venetsanos D, Alfredsson J, Lagerqvist B, Persson J, et al. Percutaneous Treatment and Outcomes of Small Coronary Vessels: A SCAAR Report. JACC Cardiovasc Interv. 2020;13(7):793-804.
302. Sarno G, Lagerqvist B, Olivecrona G, Varenhorst C, Danielewicz M, Hambraeus K, et al. Real-life clinical outcomes with everolimus eluting platinum chromium stent with an

abluminal biodegradable polymer in patients from the Swedish Coronary Angiography and Angioplasty Registry (SCAAR). Catheter Cardiovasc Interv. 2017;90(6):881-7.
303. Stenestrand U, James SK, Lindbäck J, Fröbert O, Carlsson J, Scherstén F, et al. Safety and efficacy of drug-eluting vs. bare metal stents in patients with diabetes mellitus: long-term follow-up in the Swedish Coronary Angiography and Angioplasty Registry (SCAAR). Eur Heart J. 2010;31(2):177-86.
304. Sarno G, Lagerqvist B, Nilsson J, Frobert O, Hambraeus K, Varenhorst C, et al. Stent thrombosis in new-generation drug-eluting stents in patients with STEMI undergoing primary PCI: a report from SCAAR. J Am Coll Cardiol. 2014;64(1):16-24.
305. Lagerqvist B, Carlsson J, Fröbert O, Lindbäck J, Scherstén F, Stenestrand U, et al. Stent thrombosis in Sweden: a report from the Swedish Coronary Angiography and Angioplasty Registry. Circ Cardiovasc Interv. 2009;2(5):401-8.
306. Varenhorst C, Lindholm M, Sarno G, Olivecrona G, Jensen U, Nilsson J, et al. Stent thrombosis rates the first year and beyond with new- and old-generation drug-eluting stents compared to bare metal stents. Clin Res Cardiol. 2018;107(9):816-23.
307. Almudarra SS, Gale CP, Baxter PD, Fleming SJ, Brogan RA, Ludman PF, et al. Comparative outcomes after unprotected left main stem percutaneous coronary intervention: a national linked cohort study of 5,065 acute and elective cases from the BCIS Registry (British Cardiovascular Intervention Society). JACC Cardiovasc Interv. 2014;7(7):717-30.
308. Virtanen MPO, Airaksinen J, Niemelä M, Laakso T, Husso A, Jalava MP, et al. Comparison of Survival of Transfemoral Transcatheter Aortic Valve Implantation Versus Surgical Aortic Valve Replacement for Aortic Stenosis in Low-Risk Patients Without Coronary Artery Disease. Am J Cardiol. 2020;125(4):589-96.
309. Hilling-Smith R, Smethurst J, Cockburn J, Williams T, Trivedi U, Banning A, et al. Pre-procedural pacing bias among transcatheter aortic valves with higher post-procedure pacing rates: evidence from the UK TAVI Registry. Heart Vessels. 2021;36(3):408-13.
310. Laakso T, Laine M, Moriyama N, Dahlbacka S, Airaksinen J, Virtanen M, et al. Impact of paravalvular regurgitation on the mid-term outcome after transcatheter and surgical aortic valve replacement. Eur J Cardiothorac Surg. 2020;58(6):1145-52.
311. Maaranen P, Husso A, Tauriainen T, Lahtinen A, Valtola A, Ahvenvaara T, et al. Blood Transfusion and Outcome After Transfemoral Transcatheter Aortic Valve Replacement. J Cardiothorac Vasc Anesth. 2019;33(11):2949-59.
312. Fröbert O, Lagerqvist B, Carlsson J, Lindbäck J, Stenestrand U, James SK. Differences in restenosis rate with different drug-eluting stents in patients with and without diabetes mellitus: a report from the SCAAR (Swedish Angiography and Angioplasty Registry). J Am Coll Cardiol. 2009;53(18):1660-7.
313. Völz S, Angerås O, Odenstedt J, Ioanes D, Haraldsson I, Dworeck C, et al. Sustained risk of stent thrombosis and restenosis in first generation drug-eluting Stents after One Decade of Follow-up: A Report from the Swedish Coronary Angiography and Angioplasty Registry (SCAAR). Catheter Cardiovasc Interv. 2018;92(6):E403-e9.
314. The British Cardiovascular Intervention Society (BCIS). BCIS National Audit Adult Interventional Procedures 2019. Accessible: http://bcis.org.uk/wp-content/uploads/2021/01/BCIS-Audit-2018-19-data-ALL-4-5-2020-for-web.pdf

315. Angerås O, Haraldsson I, Redfors B, Fröbert O, Petursson P, Albertsson P, et al. Impact of Thrombus Aspiration on Mortality, Stent Thrombosis, and Stroke in Patients With ST-Segment-Elevation Myocardial Infarction: A Report From the Swedish Coronary Angiography and Angioplasty Registry. J Am Heart Assoc. 2018;7(1).
316. Rashid Hons M, Gale Hons CP, Curzen Hons N, Ludman Hons P, De Belder Hons M, Timmis Hons A, et al. Impact of Coronavirus Disease 2019 Pandemic on the Incidence and Management of Out-of-Hospital Cardiac Arrest in Patients Presenting With Acute Myocardial Infarction in England. J Am Heart Assoc. 2020;9(22):e018379.
317. Kite TA, Ludman PF, Gale CP, Wu J, Caixeta A, Mansourati J, et al. International Prospective Registry of Acute Coronary Syndromes in Patients With COVID-19. J Am Coll Cardiol. 2021;77(20):2466-76.
318. Kinnaird T, Johnson T, Anderson R, Gallagher S, Sirker A, Ludman P, et al. Intravascular Imaging and 12-Month Mortality After Unprotected Left Main Stem PCI: An Analysis From the British Cardiovascular Intervention Society Database. JACC Cardiovasc Interv. 2020;13(3):346-57.
319. Jones DA, Gallagher S, Rathod KS, Redwood S, de Belder MA, Mathur A, et al. Mortality in South Asians and Caucasians after percutaneous coronary intervention in the United Kingdom: an observational cohort study of 279,256 patients from the BCIS (British Cardiovascular Intervention Society) National Database. JACC Cardiovasc Interv. 2014;7(4):362-71.
320. Hulme W, Sperrin M, Curzen N, Kinnaird T, De Belder MA, Ludman P, et al. Operator volume is not associated with mortality following percutaneous coronary intervention: insights from the British Cardiovascular Intervention Society registry. Eur Heart J. 2018;39(18):1623-34.
321. Kinnaird T, Gallagher S, Sharp A, Protty M, Salim T, Ludman P, et al. Operator Volumes and In-Hospital Outcomes: An Analysis of 7,740 Rotational Atherectomy Procedures From the BCIS National Database. JACC Cardiovasc Interv. 2021;14(13):1423-30.
322. Rathod KS, Jain AK, Firoozi S, Lim P, Boyle R, Nevett J, et al. Outcome of inter-hospital transfer versus direct admission for primary percutaneous coronary intervention: An observational study of 25,315 patients with ST-elevation myocardial infarction from the London Heart Attack Group. Eur Heart J Acute Cardiovasc Care. 2020;9(8):948-57.
323. Patel N, De Maria GL, Kassimis G, Rahimi K, Bennett D, Ludman P, et al. Outcomes after emergency percutaneous coronary intervention in patients with unprotected left main stem occlusion: the BCIS national audit of percutaneous coronary intervention 6-year experience. JACC Cardiovasc Interv. 2014;7(9):969-80.
324. Shoaib A, Kinnaird T, Curzen N, Kontopantelis E, Ludman P, de Belder M, et al. Outcomes Following Percutaneous Coronary Intervention in Non-ST-Segment-Elevation Myocardial Infarction Patients With Coronary Artery Bypass Grafts. Circ Cardiovasc Interv. 2018;11(11):e006824.
325. Nagaraja V, Rashid M, Fischmann DL, Anderson HV, Kinnaird T, Ludman P, et al. Outcomes of Percutaneous Coronary Intervention in Cardiac Transplant Patients: A Binational Analysis Derived From the United Kingdom and United States. J Invasive Cardiol. 2020;32(9):321-9.

326. Jones DA, Rathod KS, Koganti S, Lim P, Firoozi S, Bogle R, et al. The association between the public reporting of individual operator outcomes with patient profiles, procedural management, and mortality after percutaneous coronary intervention: an observational study from the Pan-London PCI (BCIS) Registry using an interrupted time series analysis. Eur Heart J. 2019;40(31):2620-9.
327. Farooq V, Goedhart D, Ludman P, de Belder MA, Harcombe A, El-Omar M. Relationship Between Femoral Vascular Closure Devices and Short-Term Mortality From 271 845 Percutaneous Coronary Intervention Procedures Performed in the United Kingdom Between 2006 and 2011: A Propensity Score-Corrected Analysis From the British Cardiovascular Intervention Society. Circ Cardiovasc Interv. 2016;9(6).
328. McAllister KS, Ludman PF, Hulme W, de Belder MA, Stables R, Chowdhary S, et al. A contemporary risk model for predicting 30-day mortality following percutaneous coronary intervention in England and Wales. Int J Cardiol. 2016;210:125-32.
329. Mamas MA, Nolan J, de Belder MA, Zaman A, Kinnaird T, Curzen N, et al. Changes in Arterial Access Site and Association With Mortality in the United Kingdom: Observations From a National Percutaneous Coronary Intervention Database. Circulation. 2016;133(17):1655-67.
330. Kunadian V, Qiu W, Lagerqvist B, Johnston N, Sinclair H, Tan Y, et al. Gender Differences in Outcomes and Predictors of All-Cause Mortality After Percutaneous Coronary Intervention (Data from United Kingdom and Sweden). Am J Cardiol. 2017;119(2):210-6.
331. Rashid M, Rushton CA, Kwok CS, Kinnaird T, Kontopantelis E, Olier I, et al. Impact of Access Site Practice on Clinical Outcomes in Patients Undergoing Percutaneous Coronary Intervention Following Thrombolysis for ST-Segment Elevation Myocardial Infarction in the United Kingdom: An Insight From the British Cardiovascular Intervention Society Dataset. JACC Cardiovasc Interv. 2017;10(22):2258-65.
332. Sirker A, Mamas M, Kwok CS, Kontopantelis E, Ludman P, Hildick-Smith D. Outcomes From Selective Use of Thrombectomy in Patients Undergoing Primary Percutaneous Coronary Intervention for ST-Segment Elevation Myocardial Infarction: An Analysis of the British Cardiovascular Intervention Society/National Institute for Cardiovascular Outcomes Research (BCIS-NICOR) Registry, 2006-2013. JACC Cardiovasc Interv. 2016;9(2):126-34.
333. Taxiarchi P, Kontopantelis E, Martin GP, Kinnaird T, Curzen N, Banning AP, et al. Same-Day Discharge After Elective Percutaneous Coronary Intervention: Insights From the British Cardiovascular Intervention Society. JACC Cardiovasc Interv. 2019;12(15):1479-94.
334. Holroyd EW, Sirker A, Kwok CS, Kontopantelis E, Ludman PF, De Belder MA, et al. The Relationship of Body Mass Index to Percutaneous Coronary Intervention Outcomes: Does the Obesity Paradox Exist in Contemporary Percutaneous Coronary Intervention Cohorts? Insights From the British Cardiovascular Intervention Society Registry. JACC Cardiovasc Interv. 2017;10(13):1283-92.
335. O'Neill D, Nicholas O, Gale CP, Ludman P, de Belder MA, Timmis A, et al. Total Center Percutaneous Coronary Intervention Volume and 30-Day Mortality: A Contemporary National Cohort Study of 427 467 Elective, Urgent, and Emergency Cases. Circ Cardiovasc Qual Outcomes. 2017;10(3).

336. Ludman PF, de Belder MA, Redwood S, Banning A. United Kingdom: coronary and structural heart interventions from 2010 to 2015. EuroIntervention. 2017;13(Z):Z83-z8.
337. George S, Cockburn J, Clayton TC, Ludman P, Cotton J, Spratt J, et al. Long-term follow-up of elective chronic total coronary occlusion angioplasty: analysis from the U.K. Central Cardiac Audit Database. J Am Coll Cardiol. 2014;64(3):235-43.
338. Hulme WJ, Sperrin M, Martin GP, Curzen N, Ludman P, Kontopantelis E, et al. Temporal trends in relative survival following percutaneous coronary intervention. BMJ Open. 2019;9(2):e024627.
339. Mamas MA, Tosh J, Hulme W, Hoskins N, Bungey G, Ludman P, et al. Health Economic Analysis of Access Site Practice in England During Changes in Practice: Insights From the British Cardiovascular Interventional Society. Circ Cardiovasc Qual Outcomes. 2018;11(5):e004482.
340. Sirker A, Kwok CS, Kontopantelis E, Johnson T, Freeman P, de Belder MA, et al. Antiplatelet drug selection in PCI to vein grafts in patients with acute coronary syndrome and adverse clinical outcomes: Insights from the British Cardiovascular Intervention Society database. Catheter Cardiovasc Interv. 2018;92(4):659-65.
341. Kite TA, Ladwiniec A, Owens CG, Chase A, Shaukat A, Mozid AM, et al. Outcomes following PCI in CABG candidates during the COVID-19 pandemic: The prospective multicentre UK-ReVasc registry. Catheter Cardiovasc Interv. 2022;99(2):305-13.
342. Kalmar P, Irrgang E. Cardiac surgery in Germany during 1991. A report by the German Society for Thoracic and Cardiovascular Surgery. Thorac Cardiovasc Surg. 1992;40(3):163-5.
343. Kalmar P, Irrgang E. Cardiac surgery in Germany during 1992. A report by the German Society for Thoracic and Cardiovascular Surgery. Thorac Cardiovasc Surg. 1993;41(3):202-4.
344. Kalmar P, Irrgang E. Cardiac surgery in Germany during 1993. A report by the German Society for Thoracic and Cardiovascular Surgery. Thorac Cardiovasc Surg. 1994;42(3):194-6.
345. Kalmar P, Irrgang E. Cardiac surgery in Germany during 1994. A report by the German Society for Thoracic and Cardiovascular Surgery. Thorac Cardiovasc Surg. 1995;43(3):181-3.
346. Kalmár P, Irrgang E. Cardiac surgery in Germany during 1995. A report by the German Society for Thoracic and Cardiovascular Surgery. Thorac Cardiovasc Surg. 1996;44(3):161-4.
347. Kalmár P, Irrgang E. Cardiac surgery in Germany during 1996. A report by the German Society for Thoracic and Cardiovascular Surgery. Thorac Cardiovasc Surg. 1997;45(3):134-7.
348. Kalmár P, Irrgang E. Cardiac surgery in Germany during 2001: a report by the German Society for Thoracic and Cardiovascular Surgery. Thorac Cardiovasc Surg. 2002;50(6):30-5.
349. Kalmár P, Irrgang E. Cardiac surgery in Germany during 2003: a report by the German Society for Thoracic and Cardiovascular Surgery. Thorac Cardiovasc Surg. 2004;52(5):312-7.

350. Gummert JF, Funkat A, Krian A. Cardiac surgery in Germany during 2004: a report on behalf of the German Society for Thoracic and Cardiovascular Surgery. Thorac Cardiovasc Surg. 2005;53(6):391-9.
351. Gummert JF, Funkat A, Beckmann A, Hekmat K, Ernst M, Krian A. Cardiac surgery in Germany during 2005: a report on behalf of the German Society for Thoracic and Cardiovascular Surgery. Thorac Cardiovasc Surg. 2006;54(5):362-71.
352. Gummert JF, Funkat A, Beckmann A, Schiller W, Hekmat K, Ernst M, et al. Cardiac surgery in Germany during 2006: a report on behalf of the German Society for Thoracic and Cardiovascular Surgery. Thorac Cardiovasc Surg. 2007;55(6):343-50.
353. Gummert JF, Funkat A, Beckmann A, Schiller W, Hekmat K, Ernst M, et al. Cardiac surgery in Germany during 2007: a report on behalf of the German Society for Thoracic and Cardiovascular Surgery. Thorac Cardiovasc Surg. 2008;56(6):328-36.
354. Gummert JF, Funkat A, Beckmann A, Schiller W, Hekmat K, Ernst M, et al. Cardiac surgery in Germany during 2008. A report on behalf of the German Society for Thoracic and Cardiovascular Surgery. Thorac Cardiovasc Surg. 2009;57(6):315-23.
355. Gummert JF, Funkat A, Beckmann A, Schiller W, Hekmat K, Ernst M, et al. Cardiac surgery in Germany during 2009. A report on behalf of the German Society for Thoracic and Cardiovascular Surgery. Thorac Cardiovasc Surg. 2010;58(7):379-86.
356. Gummert JF, Funkat AK, Beckmann A, Ernst M, Hekmat K, Beyersdorf F, et al. Cardiac surgery in Germany during 2010: a report on behalf of the German Society for Thoracic and Cardiovascular Surgery. Thorac Cardiovasc Surg. 2011;59(5):259-67.
357. Funkat AK, Beckmann A, Lewandowski J, Frie M, Schiller W, Ernst M, et al. Cardiac surgery in Germany during 2011: a report on behalf of the German Society for Thoracic and Cardiovascular Surgery. Thorac Cardiovasc Surg. 2012;60(6):371-82.
358. Beckmann A, Funkat AK, Lewandowski J, Frie M, Schiller W, Hekmat K, et al. Cardiac surgery in Germany during 2012: a report on behalf of the German Society for Thoracic and Cardiovascular Surgery. Thorac Cardiovasc Surg. 2014;62(1):5-17.
359. Funkat A, Beckmann A, Lewandowski J, Frie M, Ernst M, Schiller W, et al. Cardiac surgery in Germany during 2013: a report on behalf of the German Society for Thoracic and Cardiovascular Surgery. Thorac Cardiovasc Surg. 2014;62(5):380-92.
360. Rodewald G, Polonius MJ. Cardiac surgery in the Federal Republic of Germany during 1978 and 1979. A report by the German Society for Thoracic and Cardiovascular Surgery. Thorac Cardiovasc Surg. 1980;28(6):373-7.
361. Rodewald G, Polonius MJ. Cardiac surgery in the Federal Republic of Germany during 1981. A report by the German Society for Thoracic and Cardiovascular Surgery. Thorac Cardiovasc Surg. 1982;30(3):127-9.
362. Rodewald G, Polonius MJ. Cardiac surgery in the Federal Republic of Germany during 1982. A report by the German Society for Thoracic and Cardiovascular surgery. Thorac Cardiovasc Surg. 1983;31(3):188-90.
363. Rodewald G, Polonius MJ. Cardiac surgery in the Federal Republic of Germany during 1983. A report by the German Society for Thoracic and Cardiovascular Surgery. Thorac Cardiovasc Surg. 1984;32(6):395-7.

364. Rodewald G, Kalmar P. Cardiac surgery in the Federal Republic of Germany during 1984. A report by the German Society for Thoracic and Cardiovascular Surgery. Thorac Cardiovasc Surg. 1985;33(6):397-9.
365. Rodewald G, Kalmar P. Cardiac surgery in the Federal Republic of Germany during 1985. A report by the German Society for Thoracic and Cardiovascular Surgery. Thorac Cardiovasc Surg. 1986;34(6):406-8.
366. Rodewald G, Kalmar P. Cardiac surgery in the Federal Republic of Germany during 1986. A report by the German Society for Thoracic and Cardiovascular Surgery. Thorac Cardiovasc Surg. 1987;35(4):259-61.
367. Rodewald G, Kalmar P. Cardiac surgery in the Federal Republic of Germany during 1987. A report by the German Society for Thoracic and Cardiovascular Surgery. Thorac Cardiovasc Surg. 1988;36(3):180-2.
368. Kalmar P. Cardiac surgery in the Federal Republic of Germany during 1988. A report by the German Society for Thoracic and Cardiovascular Surgery. Thorac Cardiovasc Surg. 1989;37(3):193-5.
369. Kalmar P, Irrgang E. Cardiac surgery in the Federal Republic of Germany during 1989. A report by the German Society for Thoracic and Cardiovascular Surgery. Thorac Cardiovasc Surg. 1990;38(3):198-200.
370. Kalmar P, Irrgang E. Cardiac surgery in the Federal Republic of Germany during 1990. A report by the German Society for Thoracic and Cardiovascular Surgery. Thorac Cardiovasc Surg. 1991;39(3):167-9.
371. Beckmann A, Funkat AK, Lewandowski J, Frie M, Ernst M, Hekmat K, et al. German Heart Surgery Report 2015: The Annual Updated Registry of the German Society for Thoracic and Cardiovascular Surgery. Thorac Cardiovasc Surg. 2016;64(6):462-74.
372. Beckmann A, Meyer R, Lewandowski J, Frie M, Markewitz A, Harringer W. German Heart Surgery Report 2017: The Annual Updated Registry of the German Society for Thoracic and Cardiovascular Surgery. Thorac Cardiovasc Surg. 2018;66(8):608-21.
373. Beckmann A, Meyer R, Lewandowski J, Markewitz A, Harringer W. German Heart Surgery Report 2018: The Annual Updated Registry of the German Society for Thoracic and Cardiovascular Surgery. Thorac Cardiovasc Surg. 2019;67(5):331-44.
374. Beckmann A, Meyer R, Lewandowski J, Markewitz A, Gummert J. German Heart Surgery Report 2019: The Annual Updated Registry of the German Society for Thoracic and Cardiovascular Surgery. Thorac Cardiovasc Surg. 2020;68(4):263-76.
375. Beckmann A, Dittrich S, Arenz C, Krogmann ON, Horke A, Tengler A, et al. German Registry for Cardiac Operations and Interventions in Patients with Congenital Heart Disease: Report 2020-Comprehensive Data from 6 Years of Experience. Thorac Cardiovasc Surg. 2021;69(S 03):e21-e31.
376. Meinertz T, Diegeler A, Stiller B, Fleck E, Heinemann MK, Schmaltz AA, et al. German Heart Report 2013. Clin Res Cardiol. 2015;104(2):112-23.
377. Gummert JF, Funkat A, Osswald B, Beckmann A, Schiller W, Krian A, et al. EuroSCORE overestimates the risk of cardiac surgery: results from the national registry of the German Society of Thoracic and Cardiovascular Surgery. Clin Res Cardiol. 2009;98(6):363-9.

378. Kalmár P, Irrgang E. Cardiac surgery in Germany during 1997. A report by the German Society for Thoracic and Cardiovascular Surgery. Thorac Cardiovasc Surg. 1998;46(5):307-10.
379. Kalmàr P, Irrgang E. Cardiac surgery in Germany during 1998. A report by the German Society for Thoracic and Cardiovascular Surgery. Thorac Cardiovasc Surg. 1999;47(4):260-3.
380. Struck E, De Vivie ER, Hehrlein F, Hügel W, Kalmar P, Sebening F, et al. Multicentric quality assurance in cardiac surgery. QUADRA study of the German Society for Thoracic and Cardiovascular Surgery (QUADRA: quality data retrospective analysis). Thorac Cardiovasc Surg. 1990;38(2):123-34.
381. Beckmann A, Doebler K, Schaefer E, Koetting J, Gastmeier P, Graf K. Sternal surgical site infection prevention - is there any room for improvement? Eur J Cardiothorac Surg. 2011;40(2):347-51.
382. Benito-González T, Carrasco-Chinchilla F, Estévez-Loureiro R, Pascual I, Arzamendi D, Garrote-Coloma C, et al. Clinical and echocardiographic outcomes of transcatheter mitral valve repair in atrial functional mitral regurgitation. Int J Cardiol. 2021;345:29-35.
383. Pascual I, Carrasco-Chinchilla F, Benito-Gonzalez T, Li CH, Avanzas P, Nombela-Franco L, et al. Transcatheter Mitral Repair for Functional Mitral Regurgitation According to Left Ventricular Function: A Real-Life Propensity-Score Matched Study. J Clin Med. 2020;9(6).
384. Råmunddal T, Hoebers LP, Henriques JP, Dworeck C, Angerås O, Odenstedt J, et al. Chronic total occlusions in Sweden--a report from the Swedish Coronary Angiography and Angioplasty Registry (SCAAR). PLoS One. 2014;9(8):e103850.
385. Völz S, Dworeck C, Redfors B, Pétursson P, Angerås O, Gan LM, et al. Survival of Patients With Angina Pectoris Undergoing Percutaneous Coronary Intervention With Intracoronary Pressure Wire Guidance. J Am Coll Cardiol. 2020;75(22):2785-99.
386. Gudnason T, Gudnadottir GS, Lagerqvist B, Eyjolfsson K, Nilsson T, Thorgeirsson G, et al. Comparison of interventional cardiology in two European countries: a nationwide Internet based registry study. Int J Cardiol. 2013;168(2):1237-42.
387. Jha S, Zeijlon R, Enabtawi I, Espinosa AS, Chamat J, Omerovic E, et al. Electrocardiographic predictors of adverse in-hospital outcomes in the Takotsubo syndrome. Int J Cardiol. 2020;299:43-8.
388. Angerås O, Albertsson P, Karason K, Råmunddal T, Matejka G, James S, et al. Evidence for obesity paradox in patients with acute coronary syndromes: a report from the Swedish Coronary Angiography and Angioplasty Registry. Eur Heart J. 2013;34(5):345-53.
389. Lawesson SS, Stenestrand U, Lagerqvist B, Wallentin L, Swahn E. Gender perspective on risk factors, coronary lesions and long-term outcome in young patients with ST-elevation myocardial infarction. Heart. 2010;96(6):453-9.
390. SCAAR Annual Report 2011. Scand Cardiovasc J. 2013;47 Suppl 62:55-76.
391. Harnek J, James SK, Lagerqvist B. Very long-term outcome of coronary covered stents: a report from the SCAAR registry. EuroIntervention. 2019;14(16):1660-7.
392. Redfors B, Råmunddal T, Angerås O, Dworeck C, Haraldsson I, Ioanes D, et al. Angiographic findings and survival in patients undergoing coronary angiography due to sudden cardiac arrest in western Sweden. Resuscitation. 2015;90:13-20.

393. Mohammad MA, Koul S, Olivecrona GK, Götberg M, Tydén P, Rydberg E, et al. Incidence and outcome of myocardial infarction treated with percutaneous coronary intervention during COVID-19 pandemic. Heart. 2020;106(23):1812-8.
394. Fröbert O, Lagerqvist B, Olivecrona GK, Omerovic E, Gudnason T, Maeng M, et al. Thrombus aspiration during ST-segment elevation myocardial infarction. N Engl J Med. 2013;369(17):1587-97.
395. Kreutzer M, Magnuson A, Lagerqvist B, Fröbert O. Patent coronary artery and myocardial infarction in the era of primary angioplasty: assessment of an old problem in a new setting with data from the Swedish Coronary Angiography and Angioplasty Registry (SCAAR). EuroIntervention. 2010;6(5):590-5.
396. Wester A, Mohammad MA, Andell P, Rylance R, Dankiewicz J, Friberg H, et al. Coronary angiographic findings and outcomes in patients with sudden cardiac arrest without ST-elevation myocardial infarction: A SWEDEHEART study. Resuscitation. 2018;126:172-8.
397. Bergh N, Angerås O, Albertsson P, Dworeck C, Matejka G, Haraldsson I, et al. Does the timing of treatment with intra-aortic balloon counterpulsation in cardiogenic shock due to ST-elevation myocardial infarction affect survival? Acute Card Care. 2014;16(2):57-62.
398. Redfors B, Vedad R, Angerås O, Råmunddal T, Petursson P, Haraldsson I, et al. Mortality in takotsubo syndrome is similar to mortality in myocardial infarction - A report from the SWEDEHEART registry. Int J Cardiol. 2015;185:282-9.
399. Jurga J, Szummer KE, Lewinter C, Mellbin L, Götberg M, Zwackman S, et al. Pretreatment With P2Y12 Inhibitors in Patients With Chronic Coronary Syndrome Undergoing Percutaneous Coronary Intervention: A Report From the Swedish Coronary Angiography and Angioplasty Registry. Circ Cardiovasc Interv. 2021;14(11):e010849.
400. Redfors B, Jha S, Thorleifsson S, Jernberg T, Angerås O, Frobert O, et al. Short- and Long-Term Clinical Outcomes for Patients With Takotsubo Syndrome and Patients With Myocardial Infarction: A Report From the Swedish Coronary Angiography and Angioplasty Registry. J Am Heart Assoc. 2021;10(17):e017290.
401. Henriksson M, Palmer S, Chen R, Damant J, Fitzpatrick NK, Abrams K, et al. Assessing the cost effectiveness of using prognostic biomarkers with decision models: case study in prioritising patients waiting for coronary artery surgery. Bmj. 2010;340:b5606.
402. Buccheri S, Sarno G, Fröbert O, Gudnason T, Lagerqvist B, Lindholm D, et al. Assessing the Nationwide Impact of a Registry-Based Randomized Clinical Trial on Cardiovascular Practice. Circ Cardiovasc Interv. 2019;12(3):e007381.
403. Dworeck C, Redfors B, Angerås O, Haraldsson I, Odenstedt J, Ioanes D, et al. Association of Pretreatment With P2Y12 Receptor Antagonists Preceding Percutaneous Coronary Intervention in Non-ST-Segment Elevation Acute Coronary Syndromes With Outcomes. JAMA Netw Open. 2020;3(10):e2018735.
404. Gjesdal G, Braun O, Smith JG, Scherstén F, Tydén P. Blood lactate is a predictor of short-term mortality in patients with myocardial infarction complicated by heart failure but without cardiogenic shock. BMC Cardiovasc Disord. 2018;18(1):8.
405. Grimfjärd P, Lagerqvist B, Erlinge D, Varenhorst C, James S. Clinical use of cangrelor: nationwide experience from the Swedish Coronary Angiography and Angioplasty Registry (SCAAR). Eur Heart J Cardiovasc Pharmacother. 2019;5(3):151-7.

406. Entezarjou A, Mohammad MA, Andell P, Koul S. Culprit vessel: impact on short-term and long-term prognosis in patients with ST-elevation myocardial infarction. Open Heart. 2018;5(2):e000852.
407. Koul S, Smith JG, Scherstén F, James S, Lagerqvist B, Erlinge D. Effect of upstream clopidogrel treatment in patients with ST-segment elevation myocardial infarction undergoing primary percutaneous coronary intervention. Eur Heart J. 2011;32(23):2989-97.
408. Gudnadottir GS, Andersen K, Thrainsdottir IS, James SK, Lagerqvist B, Gudnason T. Gender differences in coronary angiography, subsequent interventions, and outcomes among patients with acute coronary syndromes. Am Heart J. 2017;191:65-74.
409. Omerovic E, Råmunddal T, Albertsson P, Holmberg M, Hallgren P, Boren J, et al. Levosimendan neither improves nor worsens mortality in patients with cardiogenic shock due to ST-elevation myocardial infarction. Vasc Health Risk Manag. 2010;6:657-63.
410. Kuhl J, Jörneskog G, Wemminger M, Bengtsson M, Lundman P, Kalani M. Long-term clinical outcome in patients with acute coronary syndrome and dysglycaemia. Cardiovasc Diabetol. 2015;14:120.
411. Wachtell K, Lagerqvist B, Olivecrona GK, James SK, Fröbert O. Novel Trial Designs: Lessons Learned from Thrombus Aspiration During ST-Segment Elevation Myocardial Infarction in Scandinavia (TASTE) Trial. Curr Cardiol Rep. 2016;18(1):11.
412. Carlsson J, James SN, Ståhle E, Höfer S, Lagerqvist B. Outcome of percutaneous coronary intervention in hospitals with and without on-site cardiac surgery standby. Heart. 2007;93(3):335-8.
413. Redfors B, Dworeck C, Haraldsson I, Angerås O, Odenstedt J, Ioanes D, et al. Pretreatment with P2Y12 receptor antagonists in ST-elevation myocardial infarction: a report from the Swedish Coronary Angiography and Angioplasty Registry. Eur Heart J. 2019;40(15):1202-10.
414. Redfors B, Dworeck C, Angerås O, Haraldsson I, Petursson P, Odenstedt J, et al. Prognosis is similar for patients who undergo primary PCI during regular-hours and off-hours: A report from SCAAR. Catheter Cardiovasc Interv. 2018;91(7):1240-9.
415. Jamaly S, Redfors B, Omerovic E, Carlsson L, Karason K. Prognostic significance of BMI after PCI treatment in ST-elevation myocardial infarction: a cohort study from the Swedish Coronary Angiography and Angioplasty Registry. Open Heart. 2021;8(1).
416. Lingman M, Albertsson P, Herlitz J, Bergfeldt L, Lagerqvist B. The impact of hypertension and diabetes on outcome in patients undergoing percutaneous coronary intervention. Am J Med. 2011;124(3):265-75.
417. Fröbert O, Lagerqvist B, Gudnason T, Thuesen L, Svensson R, Olivecrona GK, et al. Thrombus Aspiration in ST-Elevation myocardial infarction in Scandinavia (TASTE trial). A multicenter, prospective, randomized, controlled clinical registry trial based on the Swedish angiography and angioplasty registry (SCAAR) platform. Study design and rationale. Am Heart J. 2010;160(6):1042-8.
418. Völz S, Petursson P, Odenstedt J, Ioanes D, Haraldsson I, Angerås O, et al. Ticagrelor is Not Superior to Clopidogrel in Patients With Acute Coronary Syndromes Undergoing PCI: A Report from Swedish Coronary Angiography and Angioplasty Registry. J Am Heart Assoc. 2020;9(14):e015990.

419. Damman P, Varenhorst C, Koul S, Eriksson P, Erlinge D, Lagerqvist B, et al. Treatment patterns and outcomes in patients undergoing percutaneous coronary intervention treated with prasugrel or clopidogrel (from the Swedish Coronary Angiography and Angioplasty Registry [SCAAR]). Am J Cardiol. 2014;113(1):64-9.
420. Koutouzis M, Lagerqvist B, James S, Omerovic E, Matejka G, Grip L, et al. Unfractionated heparin administration in patients treated with bivalirudin during primary percutaneous coronary intervention is associated lower mortality and target lesion thrombosis: a report from the Swedish Coronary Angiography and Angioplasty Registry (SCAAR). Heart. 2011;97(18):1484-8.
421. Harnek J, James S, Lagerqvist B. Coronary Artery Perforation and Tamponade - Incidence, Risk Factors, Predictors and Outcomes From 12 Years' Data of the SCAAR Registry. Circ J. 2019;84(1):43-53.
422. Götberg M, Christiansen EH, Gudmundsdottir IJ, Sandhall L, Danielewicz M, Jakobsen L, et al. Instantaneous Wave-free Ratio versus Fractional Flow Reserve to Guide PCI. N Engl J Med. 2017;376(19):1813-23.
423. Andell P, Karlsson S, Mohammad MA, Götberg M, James S, Jensen J, et al. Intravascular Ultrasound Guidance Is Associated With Better Outcome in Patients Undergoing Unprotected Left Main Coronary Artery Stenting Compared With Angiography Guidance Alone. Circ Cardiovasc Interv. 2017;10(5).
424. Hambraeus K, Jensevik K, Lagerqvist B, Lindahl B, Carlsson R, Farzaneh-Far R, et al. Long-Term Outcome of Incomplete Revascularization After Percutaneous Coronary Intervention in SCAAR (Swedish Coronary Angiography and Angioplasty Registry). JACC Cardiovasc Interv. 2016;9(3):207-15.
425. Akerblom A, James SK, Koutouzis M, Lagerqvist B, Stenestrand U, Svennblad B, et al. Eptifibatide is noninferior to abciximab in primary percutaneous coronary intervention: results from the SCAAR (Swedish Coronary Angiography and Angioplasty Registry). J Am Coll Cardiol. 2010;56(6):470-5.
426. Frobert O, Reitan C, Hatsukami DK, Pernow J, Omerovic E, Andell P. Smokeless tobacco, snus, at admission for percutaneous coronary intervention and future risk for cardiac events. Open Heart. 2019;6(2):e001109.
427. Lagerqvist B, James SK, Stenestrand U, Lindbäck J, Nilsson T, Wallentin L. Long-term outcomes with drug-eluting stents versus bare-metal stents in Sweden. N Engl J Med. 2007;356(10):1009-19.
428. James SK, Stenestrand U, Lindbäck J, Carlsson J, Scherstén F, Nilsson T, et al. Long-term safety and efficacy of drug-eluting versus bare-metal stents in Sweden. N Engl J Med. 2009;360(19):1933-45.
429. Kedhi E, Gomes ME, Lagerqvist B, Smith JG, Omerovic E, James S, et al. Clinical impact of second-generation everolimus-eluting stent compared with first-generation drug-eluting stents in diabetes mellitus patients: insights from a nationwide coronary intervention register. JACC Cardiovasc Interv. 2012;5(11):1141-9.
430. Carlsson J, James SK, Lindbäck J, Scherstén F, Nilsson T, Stenestrand U, et al. Outcome of drug-eluting versus bare-metal stenting used according to on- and off-label criteria. J Am Coll Cardiol. 2009;53(16):1389-98.

431. Tornvall P, Collste O, Ehrenborg E, Järnbert-Petterson H. A Case-Control Study of Risk Markers and Mortality in Takotsubo Stress Cardiomyopathy. J Am Coll Cardiol. 2016;67(16):1931-6.
432. Arinell K, Josefsson J, Magnuson A, Fröbert O. Angiographic morphology impacts outcomes in STEMI patients with LAD occlusion. Catheter Cardiovasc Interv. 2011;77(1):29-34.
433. Jensen J, Lagerqvist B, Aasa M, Särev T, Nilsson T, Tornvall P. Clinical and angiographic follow-up after coronary drug-eluting and bare metal stent implantation. Do drug-eluting stents hold the promise? J Intern Med. 2006;260(2):118-24.
434. Koul S, Andell P, Martinsson A, Gustav Smith J, van der Pals J, Scherstén F, et al. Delay from first medical contact to primary PCI and all-cause mortality: a nationwide study of patients with ST-elevation myocardial infarction. J Am Heart Assoc. 2014;3(2):e000486.
435. Johnston N, Jönelid B, Christersson C, Kero T, Renlund H, Schenck-Gustafsson K, et al. Effect of Gender on Patients With ST-Elevation and Non-ST-Elevation Myocardial Infarction Without Obstructive Coronary Artery Disease. Am J Cardiol. 2015;115(12):1661-6.
436. Fröbert O, Sarno G, James SK, Saleh N, Lagerqvist B. Effect of stent inflation pressure and post-dilatation on the outcome of coronary artery intervention. A report of more than 90,000 stent implantations. PLoS One. 2013;8(2):e56348.
437. Persson J, Lindbäck J, Hofman-Bang C, Lagerqvist B, Stenestrand U, Samnegard A. Efficacy and safety of clopidogrel after PCI with stenting in patients on oral anticoagulants with acute coronary syndrome. EuroIntervention. 2011;6(9):1046-52, 8-9.
438. Saluveer O, Redfors B, Angerås O, Dworeck C, Haraldsson I, Ljungman C, et al. Hypertension is associated with increased mortality in patients with ischaemic heart disease after revascularization with percutaneous coronary intervention - a report from SCAAR. Blood Press. 2017;26(3):166-73.
439. Völz S, Redfors B, Angerås O, Ioanes D, Odenstedt J, Koul S, et al. Long-term mortality in patients with ischaemic heart failure revascularized with coronary artery bypass grafting or percutaneous coronary intervention: insights from the Swedish Coronary Angiography and Angioplasty Registry (SCAAR). Eur Heart J. 2021;42(27):2657-64.
440. Ritsinger V, Hero C, Svensson AM, Saleh N, Lagerqvist B, Eeg-Olofsson K, et al. Mortality and extent of coronary artery disease in 2776 patients with type 1 diabetes undergoing coronary angiography: A nationwide study. Eur J Prev Cardiol. 2017;24(8):848-57.
441. Fokkema ML, James SK, Albertsson P, Aasa M, Åkerblom A, Calais F, et al. Outcome after percutaneous coronary intervention for different indications: long-term results from the Swedish Coronary Angiography and Angioplasty Registry (SCAAR). EuroIntervention. 2016;12(3):303-11.
442. Fokkema ML, James SK, Albertsson P, Akerblom A, Calais F, Eriksson P, et al. Population trends in percutaneous coronary intervention: 20-year results from the SCAAR (Swedish Coronary Angiography and Angioplasty Registry). J Am Coll Cardiol. 2013;61(12):1222-30.
443. Velders MA, James SK, Libungan B, Sarno G, Fröbert O, Carlsson J, et al. Prognosis of elderly patients with ST-elevation myocardial infarction treated with primary percutaneous

coronary intervention in 2001 to 2011: A report from the Swedish Coronary Angiography and Angioplasty Registry (SCAAR) registry. Am Heart J. 2014;167(5):666-73.
444. Råmunddal T, Hoebers LP, Henriques JP, Dworeck C, Angerås O, Odenstedt J, et al. Prognostic Impact of Chronic Total Occlusions: A Report From SCAAR (Swedish Coronary Angiography and Angioplasty Registry). JACC Cardiovasc Interv. 2016;9(15):1535-44.
445. Calais F, Lagerqvist B, Leppert J, James SK, Fröbert O. Proximal coronary artery intervention: stent thrombosis, restenosis and death. Int J Cardiol. 2013;170(2):227-32.
446. Waziri H, Jørgensen E, Kelbæk H, Stagmo M, Pedersen F, Lagerqvist B, et al. Short and long-term survival after primary percutaneous coronary intervention in young patients with ST-elevation myocardial infarction. Int J Cardiol. 2016;203:697-701.
447. Ritsinger V, Saleh N, Lagerqvist B, Norhammar A. High event rate after a first percutaneous coronary intervention in patients with diabetes mellitus: results from the Swedish coronary angiography and angioplasty registry. Circ Cardiovasc Interv. 2015;8(6):e002328.
448. Venetsanos D, Omerovic E, Sarno G, Pagonis C, Witt N, Calais F, et al. Long term outcome after treatment of de novo coronary artery lesions using three different drug coated balloons. Int J Cardiol. 2021;325:30-6.
449. Edfors R, Szummer K, Evans M, Carrero JJ, Spaak J, James S, et al. Renal function is associated with long-term outcomes independent of degree of atherosclerosis: 6-year data from the Swedish Coronary Angiography and Angioplasty Registry. Eur Heart J Qual Care Clin Outcomes. 2016;2(2):91-8.
450. Johnston N, Schenck-Gustafsson K, Lagerqvist B. Are we using cardiovascular medications and coronary angiography appropriately in men and women with chest pain? Eur Heart J. 2011;32(11):1331-6.
451. Ritsinger V, Hero C, Svensson AM, Saleh N, Lagerqvist B, Eeg-Olofsson K, et al. Characteristics and Prognosis in Women and Men With Type 1 Diabetes Undergoing Coronary Angiography: A Nationwide Registry Report. Diabetes Care. 2018;41(4):876-83.
452. Koutouzis M, Lagerqvist B, Oldgren J, Akerblom A, Wahlin M, Karlsson T, et al. Long-term results following switch from abciximab to eptifibatide during percutaneous coronary intervention. Clin Cardiol. 2010;33(11):686-92.
453. Norhammar A, Lagerqvist B, Saleh N. Long-term mortality after PCI in patients with diabetes mellitus: results from the Swedish Coronary Angiography and Angioplasty Registry. EuroIntervention. 2010;5(8):891-7.
454. Wadell D, Jensen J, Englund E, Själander A. Triple therapy after PCI - Warfarin treatment quality and bleeding risk. PLoS One. 2018;13(12):e0209187.
455. Nilsson T, Lagerqvist B, Tornvall P. Coronary angiography of patients with a previous coronary artery by-pass operation is associated with a three times increased risk for neurological complications. A report from the Swedish Coronary Angiography and Angioplasty Registry (SCAAR). Scand Cardiovasc J. 2009;43(6):374-9.
456. Jensen UJ, Jensen J, Olivecrona G, Ahlberg G, Lagerquist B, Tornvall P. The role of a simulator-based course in coronary angiography on performance in real life cath lab. BMC Med Educ. 2014;14:49.
457. Haraldsson I, Gan LM, Svedlund S, Torngren K, Westergren HU, Redfors B, et al. PROspective evaluation of coronary FLOW reserve and molecular biomarkers in patients

with established coronary artery disease the PROFLOW-trial: cross-sectional evaluation of coronary flow reserve. Vasc Health Risk Manag. 2019;15:375-84.
458. Varenhorst C, Koul S, Erlinge D, Lagerqvist B, Siegbahn A, Wallentin L, et al. Relationship between clopidogrel-induced platelet P2Y12 inhibition and stent thrombosis or myocardial infarction after percutaneous coronary intervention-a case-control study. Am Heart J. 2011;162(2):363-71.
459. Tornvall P, Göransson A, Ekman J, Järnbert-Pettersson H. Myocardial Infarction in Systemic Lupus Erythematosus: Incidence and Coronary Angiography Findings. Angiology. 2021;72(5):459-64.
460. Liss P, Persson PB, Hansell P, Lagerqvist B. Renal failure in 57 925 patients undergoing coronary procedures using iso-osmolar or low-osmolar contrast media. Kidney Int. 2006;70(10):1811-7.
461. Bondesson P, Lagerqvist B, James SK, Olivecrona GK, Venetsanos D, Harnek J. Comparison of two drug-eluting balloons: a report from the SCAAR registry. EuroIntervention. 2012;8(4):444-9.
462. Branny M, Branny P, Januska J, Hudec M, Kluzova K, Kufova P, et al. Short-term clinical results among the patients undergoing transcatheter aortic valve implantation. Interventional Cardiology. 2014;13(4):177-81.
463. Ojeda S, Romaguera R, Cruz-González I, Moreno R. Spanish Cardiac Catheterization and Coronary Intervention Registry. 29th Official Report of the Interventional Cardiology Association of the Spanish Society of Cardiology (1990-2019). Rev Esp Cardiol (Engl Ed). 2020;73(11):927-36.
464. Swedish Coronary Angiography and Angioplasty Registry (SCAAR). Stent reports https://www.ucr.uu.se/swedeheart/forskning-scaar/in-english/stent-reports (accessed March 21. 2022)
465. German Society for Thoracic and Cardiovascular Surgery. Willkommen bei den Herzchirurgen. https://www.dgthg.de/ (accessed February 27. 2023)
466. Portuguese National Registry of Intervention Cardiology. https://www.apic.pt/ (accessed February 27. 2023)
477. ClinicalTrials.gov. Registry of Aortic Valve Bioprostheses Established by Catheter (FRANCE-TAVI). https://www.clinicaltrials.gov/ct2/show/NCT01777828 (accessed February 27. 2023)
478. German Aortic Valve Registry. Ziele. https://www.aortenklappenregister.de/ (accessed February 28. 2023)
479. Swedish Transcatheter Cardiac Intervention Registry. https://www.ucr.uu.se/swedeheart/start-swentry/ (accessed February 28. 2023)
