# Supplementary material for: Quality and Utility of European Cardiovascular and Orthopaedic Registries for the Regulatory Evaluation of Medical Device Safety and Performance Across the Implant Lifecycle: A Systematic Review
Source: Int J Health Policy Manag. 2023 Jul 18;12:7648. doi: 10.34172/ijhpm.2023.7648 (PMC10702370; doi:10.34172/ijhpm.2023.7648)
Supplement: Supplementary file 3 — Tables S1B-S8B. [file ijhpm-12-7648-s003.pdf]

Article title: Quality and Utility of European Cardiovascular and Orthopaedic Registries for the Regulatory Evaluation of Medical Device Safety and Performance Across the Implant Lifecycle: A Systematic Review

Journal name: International Journal of Health Policy and Management (IJHPM)

Authors' information: Lotje A. Hoogervorst[1,2]*, Timon H. Geurkink[1], Anne Lübbeke[3,4], Sergio Buccheri[5], Jan W. Schoones[6], Marina Torre[7], Paola Laricchiuta[7], Paul Piscoi[8], Alma B. Pedersen[9,10], Chris P. Gale[11,12,13], James A. Smith[14,15], Aldo P. Maggioni[16], Stefan James[5,17,18], Alan G. Fraser[19], Rob G.H.H. Nelissen[1], Perla J. Marang-van de Mheen[2]

*Correspondence to: Lotje A. Hoogervorst, Email: l.a.hoogervorst@lumc.nl

Citation: Hoogervorst LA, Geurkink TH, Lübbeke A, et al. Quality and utility of European cardiovascular and orthopaedic registries for the regulatory evaluation of medical device safety and performance across the implant lifecycle: a systematic review. Int J Health Policy Manag. 2023;12:7648. doi:10.34172/ijhpm.2023.7648

Supplementary file 3

Table S1B: Orthopaedic registries – Domain 'Identification'				
	**Country**	**Design**	**Website**	**Initial motivation / goal**
**Orthopaedic arthroplasty registries – combined**				
Croatian Register of endoprothesis	Croatia	National	N/R	N/R
German Arthroplasty Register	Germany	National	https://www.eprd.de/de/	To create a robust framework for the assessment of hip and knee arthroplasties[(45)]
Finnish Arthroplasty Register	Finland	National	https://www.thl.fi/far/	To study and ensure the quality of prostheses for the safety of patients[(81)]
Irish National Orthopaedic Register	Ireland	National	https://www.noca.ie/audits/irish-national-orthopaedic-register/	1) to improve the quality of services and care provided to patients having joint replacement surgery, and 2) to monitor the safety of implants and support hospitals should an implant recall occur[(4)]
Lithuanian Arthroplasty Register	Lithuania	National	https://lsed.lt/	N/R
Dutch Arthroplasty Register	The Netherlands	National	https://www.lroi.nl/	To provide insight into and feedback on the results of arthroplasties in the Netherlands and the related orthopaedic Care[(82)]
Hungarian Arthroplasty Register	Hungary	National	https://www.ortopedtarsasag.hu/	To collect the quality indicators of domestic hip and knee prosthesis implants with a user-friendly, internet-based system that provides real-time data processing[(7)]
Norwegian Arthroplasty Register	Norway	National	https://nrlweb.ihelse.net/	1) to prevent the use of poor prostheses in patients, and 2) to provide an overview of the products and surgical procedures in use at any time and the patient groups who need arthroplasty[(8)]
Nordic Arthroplasty Register Association	Denmark, Finland, Norway, Sweden	Multi-country	N/R	To improve the quality of research and understanding of the clinical course of patients undergoing joint replacement surgery and thereby improve the results after joint replacement surgery[(83)]
National Joint Registry for England, Wales, Northern Ireland, the Isle of Man, and the States of Guernsey	England, Wales, Northern Ireland, the Isle of Man, and the states of Guernsey	Multi-country	https://www.njrcentre.org.uk/	To record patient information and provide data on: the performance and longevity of replacement joint implants; the surgical outcomes for the hospitals where these operations are carried out; and on the performance outcomes of the surgeons who conduct the procedures[(56)]
Belgian National Arthroplasty Register	Belgium	National	https://www.ehealth.fgov.be/nl/egezondheid/beroepsbeoefenaars-in-de-gezondheidszorg/qermidorthopride/	1) to collect data to enable professionals to examine the quality of care during hip prosthesis placement; 2) to provide information about the type of prostheses placed on a patient when he or she comes for a consultation, so

				that accessories can be appropriately ordered if necessary, and 3) to determine the lifespan of the prostheses[84]
Catalan Arthroplasty Register	Catalonia (Spain)	Regional	N/R	To assess the clinical effectiveness of hip and knee arthroplasties in Catalonia[13]
National Arthroplasty Registry of Slovenia	Slovenia	National	https://www.ob-valdoltra.si/	To support quality and safe health care for the patients, as well as to improve the orthopaedic profession[34]
Italian Arthroplasty Registry	Italy	National	https://riap.iss.it/riap/	To monitor the long-term effectiveness of hip, knee, shoulder and ankle prostheses (measured as implant survival), and support regions and hospitals when recall of patients is needed because of problems reported on specific implants[36]
Emilia-Romagna Region Arthroplasty Register	Emilia-Romagna (Italy)	Regional	https://www.ior.it/en/curarsi-al-rizzoli/register-orthopaedic-prosthetic-implants/	1) to determine the demographic characteristics and the diagnostic categories of patients who have undergone replacement surgery; 2) to gather detailed information on the use of the different prostheses used in primary and revision surgery; 3) to assess the effectiveness of the different types of prostheses; 4) supply orthopaedic surgeons with a very useful tool to give the patient timely information; 5) to collaborate in post-marketing surveillance, allowing surgeons to easily identify patients implanted with a recalled implant; 6) compare the regional situation with other national and international situations; 7) to inform the Regional Orthopaedic Commission about those implants that show an abnormal failure rate, and 8) to answer questions coming from the Regional Orthopaedic Commission or from other National or European Institutions[63]
Romanian National Arthroplasty Register	Romania	National	https://www.rne.ro/	To keep track of the revision surgery, in order to compare the quality of different types of endoprosthesis, cement and surgical techniques to detect low quality implants as soon as possible, by comparing the results of different medical devices and treatments used[77]
Portuguese National Arthroplasty Register	Portugal	National	https://www.rpa.spot.pt/	To investigate what kind of arthroplasty is better than the rest, or what are the best joint pairs[85]

Scottish Arthroplasty Project Joint Registry	Scotland	National	https://www.arthro.scot.nhs.uk/	To encourage continual improvement in the quality of care provided to joint replacement surgery (arthroplasty) patients[20]
Slovakian National Arthroplasty Register	Slovakia	National	https://sar.mfn.sk/	1) to provide an epidemiological analysis of performed artificial joint replacements; 2) to identify risk factors of primary and revision implantations that result in arthroplasty failure, taking into account the age and gender of the patient, the type of implant and the method of its fixation, the surgical procedure used; 3) to reduce the number of revision operations by analyzing and eliminating risk factors; 4) to create a standard algorithm for regular checks of patients with an artificial joint replacement, thereby eliminating the occurrence of large-scale destruction during the release of the endoprosthesis, and 5) to improve the quality of patient care after joint replacement[86]
Swiss Arthroplasty Register	Switzerland	National	https://www.siris-implant.ch/	To improve quality in implant medicine by means of a continuous learning process based on systematically collected data[22]
**Orthopaedic arthroplasty registries – hips**				
Czech Republic Arthroplasty Register	Czechia	National	https://www.ksrzis.cz/	To register data on treatment with the use of an endoprosthesis and specific information specifying this treatment in more detail[87]
French Arthroplasty Register	France	National	https://www.sofcot.fr/	1) to promote and develop knowledge of French orthopaedic and trauma surgery; 2) to strengthen the links between orthopaedic and trauma surgeons in order to facilitate exchanges and to be able to reach consensus beneficial to the development of the specialty; 3) to foster relationships with other disciplines and countries concerned with the musculoskeletal system; 4) 4o organize any international, European, national or local scientific event related to its purpose, and 5) 5o coordinate the monitoring of the evolution of the specialty's practices and its environment[24]
Danish Hip Arthroplasty Register	Denmark	National	https://www.dhr.dk/	To continuously monitor and improve the quality of treatment of primary and revision THA in Denmark[88]
Swedish Hip Arthroplasty Register	Sweden	National	https://shpr.registercentrum.se/	To analyze the entire process surrounding hip replacement surgery – that is, to identify predictors of

				both good and poor outcomes in a multidimensional and individual-based manner[89]
**Orthopaedic arthroplasty registries – knees**				
Danish Knee Arthroplasty Register	Denmark	National	https://www.danishhealthdata.com/find-health-data/Dansk-Knaealloplastik-Register/	1) to examine the epidemiology of knee replacement procedures in Denmark, and 2) to monitor and facilitate continuous improvement of knee replacement surgery outcomes on both local and national levels[44]
Swedish Knee Arthroplasty Register	Sweden	National	https://www.myknee.se/	To collect, analyze, and render information that could warn against suboptimal techniques and implants[72]

Table S2B: Orthopaedic registries – Domain 'Maturity'			
	**Starting year**	**First annual report (publishing year)**	**Most recent/last annual report (publishing year)**
**Orthopaedic arthroplasty registries – combined**			
Croatian Register of endoprothesis	2006[1]	N/R	N/R
German Arthroplasty Register	2012[2]	2019[2]	2020, data till 2019[2]
Finnish Arthroplasty Register	1980[3]	1997[3]	Only updates on website[3]
Irish National Orthopaedic Register	2014[4]	2020[4]	2021, data till 2020[4]
Lithuanian Arthroplasty Register	2010[5]	N/R	N/R
Dutch Arthroplasty Register	2007[6]	2016[6]	2021, data till 2020[6]
Hungarian Arthroplasty Register	2007[7]	N/R	N/R
Norwegian Arthroplasty Register	1987[8]	1999[8]	2021, data till 2020[8]
Nordic Arthroplasty Register Association	2007[9]	N/R	N/R
National Joint Registry for England, Wales, Northern Ireland, the Isle of Man, and the States of Guernsey	2002[10]	2004[10]	2021, data till 2020[10]
Belgian National Arthroplasty Register	2009[11]	2011[11]	2019, data till 2018[11]
Catalan Arthroplasty Register	2005[12]	N/R	2017, data till 2014[13]
National Arthroplasty Registry of Slovenia	2019[14]	2019[14]	2021, data till 2020[14]
Italian Arthroplasty Registry	2006[15]	2014[15]	2020, data till 2019[15]
Emilia-Romagna Region Arthroplasty Register	2000[16]	2001[16]	2020, data till 2018[16]
Romanian National Arthroplasty Register	2001[17]	2010[17]	Data till 2011 but nowadays updates on website[17]
Portuguese National Arthroplasty Register	2009[18]	2010[18]	Data till 2013[18]
Scottish Arthroplasty Project Joint Registry	1996[19]	2002[20]	2021, data till 2020[20]
Slovakian National Arthroplasty Register	2003[21]	2003[21]	2013, data till 2011[21]
Swiss Arthroplasty Register	2012[22]	2015[22]	2020, data till 2019[22]
**Orthopaedic arthroplasty registries – hips**			
Czech Republic Arthroplasty Register	2003[23]	N/R	N/R
French Arthroplasty Register	2006[24]	2014[24]	2020, data till 2019[24]
Danish Hip Arthroplasty Register	1995[25]	2004[25]	2020, data till 2019[25]
Swedish Hip Arthroplasty Register	1979[26]	2002[27]	2020, data till 2019[26]
**Orthopaedic arthroplasty registries – knees**			
Danish Knee Arthroplasty Register	1997[28]	N/R	N/R
Swedish Knee Arthroplasty Register	1975[29]	1999[29]	2020 data till 2019[29]

Table S3B: Orthopaedic registries – Domain 'Governance'

	Mandatory	Patients' consent	Funding	Who can access the data and see results?	Privacy regulation for patients identifiable information
**Orthopaedic arthroplasty registries – combined**					
Croatian Register of endoprothesis	N/R	N/R	N/R	N/R	N/R
German Arthroplasty Register	No[30]	Required[2]	Private and public[2]	N/R	N/R
Finnish Arthroplasty Register	Yes[31]	N/R	Public[3]	N/R	N/R
Irish National Orthopaedic Register	N/R	Required[4]	Private[4]	Only hospitals' own data are accessible[4]	N/R
Lithuanian Arthroplasty Register	No[32]	N/R	N/R	N/R	N/R
Dutch Arthroplasty Register	Yes[6]	Not required[6]	Private[6]	N/R	Privacy at hospital level[6]
Hungarian Arthroplasty Register	N/R	N/R	N/R	Only orthopaedic departments' own data are accessible[7]	N/R
Norwegian Arthroplasty Register	Yes[8]	Required[8]	N/R	N/R	No privacy at hospital level[33]
Nordic Arthroplasty Register Association	N/R	N/R	N/R	N/R	Personal identification number is deleted[9]
National Joint Registry for England, Wales, Northern Ireland, the Isle of Man, and the States of Guernsey	Yes[10]	Required[10]	N/R	Patients personal data is only available for treating surgeons[10]	No privacy at hospital- and surgeon-level[10]
Belgian National Arthroplasty Register	Yes[11]	N/R	N/R	N/R	N/R
Catalan Arthroplasty Register	No[12]	N/R	N/R	N/R	N/R
National Arthroplasty Registry of Slovenia	N/R	N/R	Private and public[34]	N/R	N/R
Italian Arthroplasty Registry	No[15]	Required[35]	Public[15]	N/R	Before data are transmitted, a pseudonym is assigned to every patient[36]
Emilia-Romagna Region Arthroplasty Register	Yes[37]	N/R	N/R	N/R	N/R
Romanian National Arthroplasty Register	Yes[17]	N/R	N/R	N/R	N/R
Portuguese National Arthroplasty Register	No[38]	N/R	N/R	N/R	N/R
Scottish Arthroplasty Project Joint Registry	N/R	N/R	N/R	N/R	N/R
Slovakian National Arthroplasty Register	Yes[21]	N/R	N/R	N/R	N/R
Swiss Arthroplasty Register	Yes[22]	Required[22]	N/R	N/R	Patient personal data and clinical data are stored separately[39]
**Orthopaedic arthroplasty registries – hips**					
Czech Republic Arthroplasty Register	N/R	N/R	Public[23]	N/R	N/R
French Arthroplasty Register	No[40]	N/R	N/R	N/R	N/R
Danish Hip Arthroplasty Register	Yes[25]	N/R	Public[25]	N/R	N/R
Swedish Hip Arthroplasty Register	No[41]	Not required[26]	Private and public[26]	Data is only available for Swedish hip arthroplasty register researches[42]	N/R
**Orthopaedic arthroplasty registries – knees**					
Danish Knee Arthroplasty Register	Yes[43]	Not required[44]	N/R	N/R	N/R
Swedish Knee Arthroplasty Register	No[29]	N/R	Private and public[29]	N/R	N/R

Table S4B: Orthopaedic registries – Domain 'Coverage, design & organisation'

	No. of hospitals (% of coverage)	Number of patients/procedures (total)	Annual number of patients/procedures (last year)	Data capture and collection	Access to registry for users/members	Type of information provided, for whom and at which level	Data linkage with other sources
**Orthopaedic arthroplasty registries – combined**							
Croatian Register of endoprothesis	N/R	N/R	N/R	N/R	N/R	N/R	N/R
German Arthroplasty Register	723 (N/R)[2]	1,381,355 hip and knee arthroplasties (2012-2019)[45]	157,681 primary hip arthroplasties and 124,677 primary knee arthroplasties (2019)[45]	Web-based and barcode scanning[30, 46]	N/R	Medical device level[45]	Health insurers[2]
Finnish Arthroplasty Register	36 (N/R)[3]	229,172 primary THA and 247,068 primary knee arthroplasties (1980-2021)[3]	1,920 primary THA and 2,596 primary knee arthroplasties (2021)[3]	Web-based and barcode scanning[47]	N/R	Hospital- and medical device-level[3]	N/R
Irish National Orthopaedic Register	7 (58.3%)[48]	3,344 primary hip arthroplasties and 2,677 primary knee arthroplasties (2014-2019)[48]	1,013 primary hip arthroplasties and 781 primary knee arthroplasties (2019)[48]	Web-based and barcode scanning[48]	N/R	N/R	National database on discharges from acute public hospitals[48]
Lithuanian Arthroplasty Register	24 (100%)[49]	N/R	N/R	Web-based[32]	N/R	N/R	Implant usage database[32]
Dutch Arthroplasty Register	89 (100%)[50, 51]	353,668 primary THA and 287,777 primary TKA (2007-2020)[6]	27,205 primary THA and 19,615 primary TKA (2020)[6]	Web-based or by paper and barcode scanning[6]	Through website[6]	Hospital- and medical device-level (hospital level not public available)[6, 51]	Hospitals information systems and the Dutch national insurance database[52]
Hungarian Arthroplasty Register	N/R	N/R	N/R	N/R	N/R	N/R	N/R
Norwegian Arthroplasty Register	70 (hip arthroplasties)[53] and 82 (knee arthroplasties)[54] (100%, 2000)[55]	218,445 primary hip arthroplasties (excluded hemi prosthesis for hip fractures; 1987-2020) and 102,649 primary knee arthroplasties (1994-2020)[33]	8,538 primary hip arthroplasties (excluded hemi prosthesis for hip fractures) and 6,587 primary knee arthroplasties (2020)[33]	N/R	N/R	Hospital- and medical device-level[33]	National patient register[33]
Nordic Arthroplasty Register Association	N/R	N/R	N/R	N/R	N/R	N/R	N/R

National Joint Registry for England, Wales, Northern Ireland, the Isle of Man, and the States of Guernsey	433 (N/R)[10]	1,251,164 primary hip arthroplasties and 1,357,077 primary knee arthroplasties (2003-2020)[56]	54,858 primary hip arthroplasties and 50,904 primary knee arthroplasties (2020)[56]	Web-based[10]	Through website[10]	Hospital-, medical device-, and surgeon-level[10]	Hospital episode statistics[56]
Belgian National Arthroplasty Register	N/R	102,665 primary THA and 97,138 primary TKA (2009-2018)[57]	24,704 primary THA and 22,027 primary TKA (2018)[57]	Web-based[58]	Through website[58]	N/R	N/R
Catalan Arthroplasty Register	52 (94.5%)[59]	46,488 primary hip arthroplasties and 60,192 primary knee arthroplasties (2005-2014)[13]	11,038 primary hip arthroplasties and 12,798 primary knee arthroplasties (2014)[13]	Web-based[12]	N/R	N/R	Central register of insured persons, minimum basic data set at hospital discharge, and prosthesis catalogue[13]
National Arthroplasty Registry of Slovenia	N/R	N/R	3,075 primary THA and 2,192 primary TKA (2020)[60, 61]	Web-based and barcode scanning[34]	N/R	Hospital-, medical device-, and surgeon-level[60, 61]	N/R
Italian Arthroplasty Registry	277 (35%)[36]	N/R	27,329 primary THA and 27,588 primary TKA (2018)[62]	Web-based[35]	N/R	N/R	Hospital discharge databases[35]
Emilia-Romagna Region Arthroplasty Register	63 (N/R)[63]	120,408 primary THA and 102,786 primary knee arthroplasties (2000-2018)[63]	8,533 primary THA and 7,881 primary knee arthroplasties (2018)[63]	Data is provided in paper forms, but transferred by registry staff to an electronically database[63]	Through website[63]	Medical device level[63]	Hospitals discharge databases[63]
Romanian National Arthroplasty Register	125 (N/R)[17]	117,923 primary THA and 43,208 primary TKA (2001-2021)[17]	7,016 primary THA and 4,009 primary TKA (2021)[17]	Web-based or by paper and barcode scanning[64]	N/R	N/R	N/R
Portuguese National Arthroplasty Register	141 (N/R)[18]	20,860 primary hip arthroplasties and 20,110 primary knee arthroplasties (2009-2013)[38]	4,440 primary hip arthroplasties and 4,234 primary knee arthroplasties (2013)[38]	N/R	N/R	Hospital- and medical device-level[38]	N/R

Scottish Arthroplasty Project Joint Registry	16 (N/R)[20]	132,180 primary hip arthroplasties and 123,246 primary knee arthroplasties (2001-2020)[20]	4,034 primary hip arthroplasties and 3,199 primary knee arthroplasties (2020)[20]	N/R	N/R	N/R	N/R
Slovakian National Arthroplasty Register	40 (hip arthroplasties) and 31 (knee arthroplasties) (N/R)[65]	45,350 primary THA and 36,943 primary TKA (2003-2020)[90]	5,347 primary THA and 3,754 primary TKA (2020)[90]	N/R	N/R	Hospital- and medical device-level[65]	N/R
Swiss Arthroplasty Register	186 (100%)[39]	134,673 primary THA and 102,638 primary TKA (2012-2019)[39]	19,897 primary THA and 15,378 primary TKA (2019)[39]	Web-based or by paper and barcode scanning[39]	N/R	Medical device level[39]	The federal office of public health and implant sales[39]
**Orthopaedic arthroplasty registries – hips**							
Czech Republic Arthroplasty Register	72 (N/R)[23]	101,734 primary hip arthroplasties (2003-2012)[23]	N/R	N/R	N/R	Medical device-level[23]	N/R
French Arthroplasty Register	N/R	52,391 primary hip arthroplasties (2006-2021)[66]	3,146 primary hip arthroplasties (september 2020-september 2021)[66]	N/R	N/R	N/R	N/R
Danish Hip Arthroplasty Register	47 (N/R)[67]	191,946 primary THA (1995-2019)[67]	11,193 primary THA (2019)[67]	Web-based or by paper[25]	N/R	Hospital- and medical device-level[67]	All the Danish medical databases and the administrative registers[68]
Swedish Hip Arthroplasty Register	75 (N/R)[26, 69]	306,075 primary THA (2000-2019)[26, 69]	19,942 primary THA (2019)[26, 69]	Web-based[26, 69]	N/R	Hospital- and medical device-level[26, 69]	National patient register[41]
**Orthopaedic arthroplasty registries – knees**							
Danish Knee Arthroplasty Register	57 (95%)[44]	141,085 primary knee arthroplasties (1997-2019)[70]	10,184 primary knee arthroplasties (2019)[70]	Web-based[44]	N/R	Hospital level[71]	National patient register[70]
Swedish Knee Arthroplasty Register	72 (100%)[72]	302,589 primary knee arthroplasties (1975-2019)[72]	16,929 primary knee arthroplasties (2019)[72]	Paper forms, expect for PROMs (web-based)[72]	N/R	Hospital- and medical device-level[72]	National patient register[72]

**Table S5B: Orthopaedic registries – Manufacturers mentioned in annual reports, peer-reviewed publications & websites**	
**Hip arthroplasties**	**Knee arthroplasties**
aap Implantate AG[77]	Adler Ortho[63]
Adler Ortho[13, 33, 63, 77]	Aesculap AG[45, 48, 65]
Aesculap AG[13, 23, 45, 77]	Amplitude[39, 63]
Amplitude[51, 63, 67, 79]	Anika[48]
Anika[79]	Arthrex[39, 51, 63]
Argomedical[77]	B. Braun[38, 39, 56, 63]
AristoTech Industries GmbH[49]	CERAVER[63, 65]
ARTIQO[45]	Citieffe[63]
ASCO Medical[77]	Corin[39, 45, 51, 56, 63]
Aston-Med[79]	DEDIENNE Sante[63]
ASTON-SEM[38, 79]	DePuy Synthes[3, 13, 38, 39, 45, 48, 51, 56, 63, 65]
Atesos Medical AG[45]	Endoplant GmbH[65]
Auxein Medical[77]	Endoplus Orthopedics[63]
B. Braun[38, 39, 65, 67, 79]	Exactech[13, 38, 51, 56, 63]
Biotechni[33, 77]	Finceramica[63]
Citieffe[63]	Groupe Lepine[13]
Corin[13, 26, 45, 56, 79]	Implantcast GmbH[45, 56]
DEDIENNE Sante[79]	Lafitt[38]
DePuy Synthes[3, 13, 26, 33, 38, 39, 45, 48, 51, 56, 63, 65, 67, 77, 79]	LimaCorporate[38, 39, 45, 51, 63, 65]
Endoplant GmbH[63]	Mathys Ltd Bettlach[39, 45, 51, 63, 65]
Endoplus Orthopedics[63]	MatOrtho Limited[33, 51, 56]
Evolutis[33, 38, 79]	MBA[38]
FH Orthopedics[38, 79]	Medacta International[39, 45, 56]
Finsbury Orthopaedics[63]	MEDIN[65]
FOURNITURES HOSPITALIERES[77]	MicroPort Orthopedics[13, 39, 45, 56]
Groupe Lepine[33, 38, 49, 63, 77, 79]	OHST[45]
Gruppo Bioimpianti[63, 77]	Permedica S.p.A.[63]
Hipokrat[77]	SAMO[63]
Implantcast GmbH[33, 45, 51, 63]	SERF[65]
ImplanTec[39]	Smith+Nephew[13, 38, 39, 45, 48, 51, 56, 63]
IMPOL[38]	Speetec Implantate GmbH[45]

Joint Medica[33]	STRYKER[3, 13, 38, 39, 45, 48, 51, 56, 63, 65]
JRI Orthopaedics[13, 33, 56]	Surgival[13]
Lafitt[38]	Symbios[63]
LimaCorporate[33, 38, 39, 48, 51, 63, 65, 67, 79]	Waldemar LINK[13, 38, 39, 45, 56, 63, 65]
Mathys Ltd Bettlach[39, 45, 51, 63, 79]	Wright Medical UK[38, 63]
MatOrtho Limited[13, 26]	Zimmer Biomet[3, 13, 38, 39, 45, 48, 51, 56, 63, 65]
MBA[38]	
Medacta International[38, 39, 45, 48, 63, 79]	
Medcomtech[38]	
MEDIN[23]	
Merete GmbH[38]	
MicroPort Orthopedics[13, 33, 45, 56, 79]	
Narang Medical Limited[77]	
Permedica S.p.A.[38, 63, 77]	
Peter Brehm[38, 45]	
Plus Orthopedics[39]	
Protetim[77]	
REDA Instrumente GmbH[77]	
SAMO[38, 63]	
SERF[13, 48, 63, 65, 67, 79]	
Shakti Orthopaedic[77]	
Signature Orthopaedics[65]	
Smith+Nephew[3, 13, 26, 33, 38, 39, 45, 48, 51, 56, 63, 67, 77, 79]	
STRYKER[3, 13, 26, 33, 38, 39, 45, 48, 49, 51, 56, 63, 65, 67, 77, 79]	
Surgival[63, 77]	
Symbios[39, 45, 63, 79]	
TIPMED[77]	
TST Medical Devices[77]	
V2-EVREN[77]	
Waldemar LINK[3, 13, 26, 33, 45, 51, 63, 65, 67, 77]	
Wright Medical UK[38, 63]	
Zimmer Biomet[3, 13, 23, 26, 33, 38, 39, 45, 48, 51, 56, 63, 65, 67, 77, 79]	

Table S6B: Orthopaedic registries – Domain 'Data quality & completeness'

	Quality assurance system defined/quality check of data	Missing data for patients' characteristics	Methods for handling missing data	Data completeness on patients/procedure-level
**Orthopaedic arthroplasty registries – combined**				
Croatian Register of endoprothesis	N/R	N/R	N/R	N/R
German Arthroplasty Register	The registry thoroughly reviews incoming data sets to identify inconsistencies[45]	N/R	N/R	70% hip and knee arthroplasties combined (2019)[45]
Finnish Arthroplasty Register	Annual database check (comparing data with the nationwide hospital discharge registry)[73]	7% ASA score and 12% BMI (hip arthroplasties 2014-2021)[3]	N/R	95% primary hip arthroplasties and 95% primary knee arthroplasties (2021)[3]
Irish National Orthopaedic Register	N/R	N/R	N/R	19% hip arthroplasties and 24% knee arthroplasties (2018)[48]
Lithuanian Arthroplasty Register	N/R	N/R	N/R	86% primary THA (2011-2013)[32] and 95% primary TKA (2016)[74]
Dutch Arthroplasty Register	Annual database check, automatic implant library (if the entered product does not correspond with the current target joint, a warning message on display will appear)[51]	Ranging 0.0-4.6% for hip arthroplasties and 0.0-2.1% for knee arthroplasties (2020)[51]	N/R	96.5% hip arthroplasties and 99.2% for knee arthroplasties (2020)[51]
Hungarian Arthroplasty Register	N/R	N/R	N/R	N/R
Norwegian Arthroplasty Register	N/R	N/R	N/R	97,5% primary hip arthroplasties and 97,6% primary knee arthroplasties (2017)[33]
Nordic Arthroplasty Register Association	N/R	N/R	N/R	N/R
National Joint Registry for England, Wales, Northern Ireland, the Isle of Man, and the States of Guernsey	Monthly or quarterly data quality checks (not further specified)[56]	N/R	Missing data is considered as missing completely at random (not further specified)[56]	97,6% primary hip arthroplasties and 98,5% for primary knee arthroplasties (2018)[56]
Belgian National Arthroplasty Register	N/R	N/R	N/R	N/R
Catalan Arthroplasty Register	Data quality check twice a year (not further specified)[12]	N/R	N/R	69,4% primary hip arthroplasties and 69,6% primary knee arthroplasties (2014)[75]
National Arthroplasty Registry of Slovenia	Data quality checks are often performed (not further specified)[34]	N/R	N/R	93% hip arthroplasties and 99,4% knee arthroplasties (2020)[60, 61]
Italian Arthroplasty Registry	Syntactic and semantic data quality checks first on procedure and then on device data[62]	Records not passing quality checks[62]:	Records not passing quality checks are not	65,8% hip arthroplasties and 63,7% knee arthroplasties (2018)[62]

		Procedures: hip (3.8%), knee (3.7%)<br>Devices: hip (7.3%), knee (6.0%)	included in the analyses[62]	
Emilia-Romagna Region Arthroplasty Register	Checking data on coverage & correct classification of implanted components, matching towards regional databases, and continuous check of data entry[37]	N/R	N/R	96% of hip, knee and shoulder arthroplasties (2018)[63]
Romanian National Arthroplasty Register	N/R	N/R	N/R	N/R
Portuguese National Arthroplasty Register	N/R	N/R	N/R	N/R
Scottish Arthroplasty Project Joint Registry	N/R	N/R	N/R	N/R
Slovakian National Arthroplasty Register	N/R	N/R	N/R	N/R
Swiss Arthroplasty Register	The plausibility of the data is checked as closely as possible at the time it is entered in order to obtain “valid values” for each data record using an automatically analysis script, and if necessary the user will be contacted to correct the data[22]	11% ASA score and 15% BMI for hip arthroplasties and 10% ASA score and 15% BMI for knee arthroplasties (2019)[39]	N/R	91,7% primary THA (for all reasons excluding trauma) and 94,1% primary knee arthroplasties (for all reasons excluding trauma) (2018)[39]
**Orthopaedic arthroplasty registries – hips**				
Czech Republic Arthroplasty Register	N/R	N/R	N/R	N/R
French Arthroplasty Register	N/R	N/R	N/R	N/R
Danish Hip Arthroplasty Register	N/R	8% ASA score and 11% BMI (2020)[67]	N/R	96,5% primary THA (2019)[67]
Swedish Hip Arthroplasty Register	During registration there are compulsory entries that cannot be left blank if the data is to be saved, the web input module comes with automatically generated controls, control reports are automatically generated if operation-data for one or more variables is missing or if the data is inconsistent, then the hospital in question is contacted and corrects the data itself or a medical record is sent to the register for follow-up, contact secretaries and doctors receive a balancing report twice per year in order to be able to check that the reported operations balances with the real production unit is requested to control its register-balance with the local patient administrative system[26]	0.4% ASA score, 0.7% BMI, 0.1% fixation method, 0.2% articulation (2018)[26]	N/R	98% hip arthroplasties (2018)[26]
**Orthopaedic arthroplasty registries – knees**				

Danish Knee Arthroplasty Register	Checked every 3 months (comparing data with the national patient registry), the entered data are regularly subject to missing value control for all variables included in the dataset, checks for coding errors are continuously performed for several of the most important variables (e.g., date of surgery and implant design)[44]	N/R	Missing procedures will be sent every 3 months to each orthopaedic department and request for data entry[44]	98-99% primary knee arthroplasties (2019)[76]
Swedish Knee Arthroplasty Register	Randomly selected hospitals with >50 annual procedures are asked to produce patient records for 25 primary knee arthroplasties and these will be compared to data which is entered in the register. Staff from the Swedish Knee Arthroplasty Register are visiting hospitals to gather patient data and will compared to data entered in the register[72]	Reported in most variables (ranging 0.0-5.1%)[72]	N/R	97,1% knee arthroplasties (2018)[72]

Table S7B: Orthopaedic registries – Outcomes reported, definition & duration of follow-up					
	**Revision**	**Reasons for revision (hip)**	**Reasons for revision (knee)**	**PROMs**	**Other**
**Orthopaedic arthroplasty registries – combined**					
Croatian Register of endo prothesis	N/R	N/R	N/R	N/R	N/R
German Arthroplasty Register	Cumulative revisions[45]; 6-month[45]; 1-year[45]; 18-month[45]; 2-year[45]; 30-month[45]; 3-year[45]; 42-month[45]; 4-year[45]; 54-month[45]; 5-year[45].	Infection, loosening (cup/stem/cup & stem), osteolysis with fixed component (cup/stem/cup & stem), periprosthetic fracture, dislocation, wear, component failure, malalignment, progression of arthrosis, condition after removal, and 'other'[45]	Infection, loosening (femoral component/tibial tray/patellar component/several components), osteolysis with fixed component (femoral component/tibial tray/patellar component/several components), periprosthetic fracture, ligament instability, wear, component failure, malalignment, restricted mobility, progression of arthrosis, condition after removal, and 'other'[45]	N/R	N/R
Finnish Arthroplasty Register	Revision risks[3]; 1-year[3]; 3-year[3]; 5-year[3]; 7-year[3]; 10-year[3]; 15-year[3]; 20-year[3]; 25-year[3].	Acute femoral neck fracture, adverse reaction to metal debris, aseptic loosening of the acetabular component, aseptic loosening of the femoral component, acetabular osteolysis, avascular necrosis of femoral head, breakage of the acetabular component, breakage of the femoral head, breakage of the liner, breakage of the modular neck, breakage of the stem, correction of leg length discrepancy, developmental dysplasia of the hip, dislocation, failed osteosynthesis (pertrochanteric femoral fracture), femoral osteolysis,	Primary OA, acute fracture (femur), aseptic loosening of femoral component, aseptic loosening of patellar component, aseptic loosening of tibial component, breakage of femoral component, breakage of insert, breakage of patellar component, breakage of tibial component, dislocation of insert, failed osteosynthesis (tibia), femoral osteolysis, infection, instability of PF joint, instability of TF joint, malposition of femur component, malposition of patellar component, malposition of tibia component, other disease, periprosthetic femoral	N/R	N/R

		infection, inflammatory psoriatic arthritis, lack of osseointegration (cup), lack of osseointegration (stem), malposition of the acetabular component, malposition of the femoral component, metastasis, other disease, other inflammatory disease, periprosthetic acetabular fracture, periprosthetic femoral fracture, posttraumatic secondary OA, primary OA, trunnion problem, unspecific pain, unusual noise of implant, and 'other'[3]	fracture, periprosthetic patellar fracture, periprosthetic tibial fracture, status post septic arthritis, stiffness, tibial osteolysis, unspecific pain, wear of insert, wound necrosis, and 'other'[3]		
Irish National Orthopaedic Register	Revision rates[48]; <1 year[48].	Aseptic loosening, component failure, infection, instability, pain of unknown origin, periprosthetic fracture, and 'other'[48]	Aseptic loosening femur, aseptic loosening tibia, infection, instability, malalignment, pain of unknown origin, and 'other'[48]	EQ-5D-5L hip/knee, Oxford Hip Score, and Oxford Knee Score[48]; Pre-operatively[48]; 6-month post-operatively[48]; 2-year post-operatively[48]; 5-year post-operatively)[48].	Cardiopulmonary complications within 30-days of hip/knee surgery, dislocation within 30-days of hip surgery, infections within 30-days of hip/knee surgery, instability within 30-days of knee surgery, mortality within 30-days of hip/knee surgery, periprosthetic fracture within 30-days of hip/knee surgery, thromboembolic events within 90-days of hip/knee surgery, wound hematoma within 30-days of hip/knee surgery[48]
Lithuanian Arthroplasty Register	N/R	N/R	N/R	N/R	N/R
Dutch Arthroplasty Register	Cumulative revision percentages[6]; 1-year[6]; 3-year[6]; 5-year[6]; 7-year[6]; 10-year[6]; 12-year[6].	Dislocation, girdlestone situation, infection, inlay wear, loosening of acetabular component, loosening of femur component, peri-articular ossification, peri-prosthetic fracture, symptomatic MoM bearing, and 'other'[6]	Arthrofibrosis, infection, insert wear, instability, loosening of femur component, loosening of patella component, loosening of tibia component, malalignment, patellar dislocation, patellar pain, periprosthetic fracture, progression of OA, revision after knee removal, and 'other'[6]	Anchor question: daily functioning hip/knee, anchor question: pain knee, NRS rest scores hip/knee, NRS activity scores hip/knee, NRS satisfaction scores knee, EQ-5D index scores hip/knee, EQ-5D thermometer scores hip/knee, HOOS-PS scores hip, Oxford hip scores, Oxford knee scores, KOOS-PS scores knee[6]; Pre-operatively[6];	Re-revision hip (dislocation/infection/inlay wear/loosening of acetabulum component/loosening of femur component/peri-articular ossification/peri-prosthetic fracture/symptomatic MoM bearing/other), re-revision knee (arthrofibrosis/infection/insert wear/instability/loosening of femur component/loosening of patella component/loosening of tibia component/malalignment/patellar dislocation/patellar pain/periprosthetic fracture/progression OA/other)[6]

				3-month post-operatively[6]; 1-year post-operatively[6].	
Hungarian Arthroplasty Register	N/R	N/R	N/R	N/R	N/R
Norwegian Arthroplasty Register	Revision rates[33]; Unknown FU.	Deep infection, dislocation, gluteal failure, implant failure, loosening of acetabular component, loosening of femoral component, osteolysis acetabular (no loosening), osteolysis femur (no loosening), pain, periprosthetic fracture, polyethylene wear, previous girdlestone, and 'other'[33]	Deep infection, defect polyethylene, dislocation (no patella), dislocation of patella, fracture near implant, instability, loose distal component, loose patella component, loose proximal component, malalignment, pain, and 'other'[33]	EQ-5D scores hip/knee, HOOS scores hip, KOOS scores knee[33]; Pre-operatively[33]; 1-year post-operatively[33].	Per-operative complications hip (not specified), peri-operative complications knee (administrative failure/anesthesia problems/avulsion fractures/blood torniquet failing/failure of instruments/fracture/ligament rupture/patella tendon rupture/problem difficulty due to anatomy/rupture of damage MCL/technical problem with cement/tendon injury/violation of sterility tourines/other)[33]
Nordic Arthroplasty Register Association	N/R	N/R	N/R	N/R	N/R
National Joint Registry for England, Wales, Northern Ireland, the Isle of Man, and the States of Guernsey	Cumulative revisions[56]; 1-year[56]; 2-year[56]; 3-year[56]; 4-year[56]; 5-year[56]; 6-year[56]; 7-year[56]; 8-year[56]; 9-year (only knees)[56]; 10-year[56]; 11-year (only knees)[56]; 12-year[56]; 13-year[56]; 14-year (only knees)[56]; 15-year[56];	Adverse reaction to particulate debris, aseptic loosening, dislocation & subluxation, head of socket size mismatch, implant fracture, infection, lysis, malalignment, pain, and periprosthetic fracture[56]	Aseptic loosening or lysis, dislocation & subluxation, implant wear, infection, instability, malalignment, pain, and 'other'[56]	N/R	30-days, 90-days, 1-year, 5-years, 10-years, 15-years mortality hip/knee, 17-years mortality hip, re-revisions hip (adverse reaction to particulate debris/aseptic loosening/dislocation & subluxation/head of socket size mismatch/implant fracture/infection/lysis/malalignment/pain/periprosthetic fracture), re-revision knee (aseptic loosening or lysis/dislocation & subluxation/implant wear/infection/instability/malalignment/pain/periprosthetic fracture/progressive arthritis/stiffness/other)[56]

	16-year[56];<br>17-year[56].				
Belgian National Arthroplasty Register	Revision rates[57];<br>1-year[57];<br>2-year[57];<br>3-year[57];<br>4-year[57];<br>5-year[57];<br>6-year[57];<br>7-year[57];<br>8-year[57];<br>9-year[57];<br>10-year (only hips)[57].	Aseptic loosening, infection, instability, pain, periprosthetic fracture, wear, and 'other'[57]	Aseptic loosening, implant failure, infection, instability, malalignment, pain, periprosthetic fracture, progressive OS in nonreplaced component, stiffness, wear of polyethylene component, and 'other'[57]	N/R	90-days mortality hip/knee[57]
Catalan Arthroplasty Register	Cumulative revision rates[75];<br>1-month[75];<br>3-month[75];<br>1-year[75];<br>3-year[75];<br>5-year[75];<br>7-year[75];<br>9-year[75].	Infection, mechanical complications, and 'other'[75]	Infection, mechanical complications, and 'other'[75]	N/R	N/R
National Arthroplasty Registry of Slovenia	Revision rates[60, 61];<br>Unknown FU.	Chronic infection, condition after girdlestone, dislocation, early infection, implant broken, loosening, metallosis, NP, osteolysis, pain, paraarticular ossification, periprosthetic fracture of acetabulum, periprosthetic fracture of femur, wear of inlay, and 'other'[60]	2-stage revision, chronic infection (>3 months), early infection (<3 months), femoral component loosening, implant broken, inequality, instability, instability of cruciate ligaments, instability of lateral ligaments, malimplantation, non-diagnosis, necrosis, OA of other component, pain, patellar dislocation, periprosthetic fracture, poor ROM, tibial component removal, total prosthesis loosening, and 'other'[61]	N/R	N/R

Italian Arthroplasty Registry	Revision rates[62]; Unknown FU.	Aseptic loosening (cup), aseptic loosening (stem), aseptic loosening (total), disease progression, implant fracture, infection, lysis, pain, periprosthetic fracture, previous prosthesis removal, prosthesis dislocation, wear, and 'other'[62]	Aseptic loosening of femur, aseptic loosening of patella, aseptic loosening of several components, aseptic loosening of tibia, disease progression, dislocation, fractured spacer, implant fracture, infection, instability, pain, periprosthetic fracture, stiffness, wear, and 'other'[62]	N/R	Discharge destination hip/knee (deceased/discharge against medical advice/discharge to a nursing home/discharge to a residential health care/discharge to hospital at home/ordinary discharge/transfer in the same hospital/transfer to an acute admission unit of a different hospital/transfer to an inpatient rehabilitation hospital)[62]
Emilia Romagna Region Arthroplasty Register	Revision rates[63]; 5-year[63]; 10-year[63] 15-year[63]; 18-year (only hips)[63].	Acetabulum fracture, aseptic loosening (cup), aseptic loosening (stem), aseptic loosening (total), bone fracture, heterotopic bone, metallosis, pain without loosening, poly wear, primary instability, prosthesis breakage, prosthesis dislocation, septic loosening, trauma, two steps prosthesis removal, and 'other'[63]	Aseptic loosening of femoral component, aseptic loosening of tibial component, breakage prosthesis, insert wear, instability, pain without loosening, periprosthetic bone fracture, progression of disease, prosthesis dislocation, septic loosening, stiffness, total aseptic loosening, trauma, two steps prosthesis removal, and 'other'[63]	N/R	Deaths during hospitalization hip/knee, deaths within 90-days after procedure hip, intra-operative complications hip (acetabulum fracture/anesthesiologic/calcar fracture/diaphysis fracture/greater trochanter fracture/hemorragia/instability/other), intra-operative complications knee (anesthesiologic/femoral fracture/hemorragia/ligament lesion/rupture patellar tendon/tibial fracture/tibial tuberosity fracture/vascular lesion/other), post-operative complications hip/knee (early infection/deep venous thrombosis), re-revision hip (aseptic loosening (cup)/aseptic loosening (stem)/breakage prosthesis/global aseptic loosening/pain without loosening/periprosthetic bone fracture/poly wear/primary instability/recurrent prosthesis dislocation/septic loosening/unknown/other)[63]
Romanian National Arthroplasty Register	Revision rates[77]; 1-year (only hips)[77]; 2-year (only hips)[77]; 3-year (only hips)[77]; 4-year (only hips)[77]; 5-year (only hips)[77]; 6-year (only hips)[77]; 7-year (only hips)[77]; 8-year (only hips)[77]; 9-year (only hips)[77]; 10-year (only hips)[77].	Acetabular loosening, acetabular osteolysis, acetabular protrusion, broken implant, cotiloiditis, early infection, femoral osteolysis, late infection, luxation, paraarticular ossification, periprosthetic fracture, wear, and 'other'[77]	N/R	N/R	90-days and 1-year mortality after primary hip procedure[77]
Portuguese National	Number of revisions[38]; Unknown FU.	Aseptic loosening, deficient implantation stem, dislocation, dissociation, fracture implant,	Aseptic loosening, deficient implantation, fracture of the implant, infection, luxation,	N/R	N/R

Arthroplasty Register		infection, osteolysis stem, pain, PE wear, and 'other'[38]	osteolysis, pain, polyethylene wear, and periprosthetic fracture[38]		
Scottish Arthroplasty Project Joint Registry	Revision rates[20]; Unknown FU.	N/R	N/R	N/R	Acute renal failure/acute myocardial infarction/CVA/ within 30-days after hip/knee procedure, mortality/deep venous thrombosis/pulmonary embolism within 90-days after hip/knee procedure, dislocation/infection withing 1-year after hip/knee procedure[20]
Slovakian National Arthroplasty Register	Cumulative revision risks[65]; 1-month[65]; 3-month[65]; 1-year[65]; 2-year[65]; 3-year[65]; 4-year[65]; 5-year[65]; 6-year[65]; 7-year (only hips)[65]; 8-year (only hips)[65]; 9-year (only hips)[65].	Acetabular protrusis, aseptic loosening of acetabular component, aseptic loosening of both components, aseptic loosening of femur component, big bone defect of acetabulum, big bone defect of femur, chronic infection, early infection, fracture of implant, girdlestone to THA, luxation, osteolysis of acetabulum, osteolysis of femur, paraarticular ossifications, periprosthetic fracture, polyethylene wear, spacer to THA, and 'other'[65]	Aseptic loosening of femoral component, aseptic loosening of patellar component, aseptic loosening of tibial component, chronic infection, collateral ligament instability, early infection, fracture of the implant, instability of PCL, knee pain without loosening, luxation, malposition, patellar luxation, patellar pain, PE wear, periprosthetic fracture, spacer to TKA, stiffness, and 'other'[65]	N/R	N/R
Swiss Arthroplasty Register	Cumulative revision rates[78]; 1-year[78]; 2-year[78]; 3-year[78]; 4-year[78]; 5-year[78]; 6-year[78]; 7-year[78].	Acetabular osteolysis, acetabular protrusion, blood ion level, dislocation, femoral osteolysis, impingement, implant breakage, infection, loosening acetabular component, loosening femoral component, metallosis, periprosthetic fracture, position orientation of stem, position orientation of the cup, squeaking, status after spacer, trochanter pathology, wear, and 'other'[78]	Component malposition femur, component malposition tibia, femorotibial instability, infection, joint stiffness or arthrofibrosis, loosening femur, loosening patella, loosening tibia, pain, patella problems, patellar instability, periprosthetic fracture femur, periprosthetic fracture patella, periprosthetic fracture tibia, sizing femoral component, sizing tibial component, wear of inlay, and 'other'[78]	N/R	30-days, 90-days, 1-year, 2-years, 3-years, 4-years, 5-years, and 6-years mortality after hip procedure[78]
**Orthopaedic arthroplasty registries – hips**					

Czech Republic Arthroplasty Register	N/R	N/R	N/A	N/R	N/R
French Arthroplasty Register	Cumulative revision risks & revision rate per 100 observed component years[79];<br>1-year[79];<br>2-year[79];<br>3-year[79];<br>4-year[79];<br>5-year[79];<br>6-year[79].	Aseptic loosening, calcifications, deep acute infection, dislocation, head and neck resection, implant fracture, pain, peri-operative fracture, periprosthetic fracture, removal of material, septic loosening due to chronic infection, wear and/or osteolysis, and 'other'[79]	N/A	N/R	N/R
Danish Hip Arthroplasty Register	Revision rate per 100 observed component years[67];<br>Up to 25-year[67].	Aseptic loosening acetabular component, aseptic loosening femoral component, aseptic loosening of femoral & acetabular component, component failure, dislocation, femoral fracture, infection, osteolysis without loosening, pain, PE wear without loosening, and 'other'[67]	N/A	N/R	5-years mortality after primary THA, 5-years mortality after primary THA due to OA, blood transfusion within 7-days after primary THA due to OA, rehospitalization after primary THA due to OA, rehospitalization after primary THA due to fracture, reprocedures within 1-year after primary THA due to infection, reprocedures within 2-years after primary THA, reprocedures within 2-years after primary THA due to OA, reprocedures within 2-years after primary THA due to fracture, re-revision (aseptic loosening (all)/component failure/dislocation/femoral fracture/infection/osteolysis without loosening/pain/Polyethylene wear without loosening/'other')[67]
Swedish Hip Arthroplasty Register	Survival rates[26];<br>2-year[26];<br>5-year[26];<br><10-year[26];<br>10-15year[26].	Dislocation, implant fracture, infection, instability, loosening, periprosthetic fracture, and 'other'[26]	N/A	Pre-operatively, 1-year, 6-years, and 10-years post-operatively (EQ VAS/EQ-5D-5L index/pain VAS/satisfaction VAS)[26]	2nd and 3rd revision (dislocation/extraction without registered insertion (yet)/infection/loosening/periprosthetic fracture/other), 9-years patient survival, 30-days and 90-days mortality after hip procedure, adverse events within 30- and 90-days after hip procedure (i.e., all kinds of readmissions that can be assumed to have a connection with the operation that has been carried out, divided into: cardiovascular/medical/surgical complications), reoperation in 1st/2nd/3rd year after hip procedure (allergy/ALVAL and/or pseudotumor/bleeding, hematoma/cup and/or liner wear/cyst and/or bursa/delayed fracture healing/difference in bone length/dislocation

					and/or instability/dislocation/fracture of spacer/faulty inserted implant/fracture acetabulum/fracture femur/fracture under resurfacing prosthesis/heightened metal ion concentrations/heterotopic bone formation/implant rupture inclusive plate rupture/inadequate cementation/loose piece of cement/infection/loose implant part/loosening/malignant or benign tumor/material left behind (not cement)/nerve or vascular injury/osteolysis acetabulum and/or femur/per operative fracture (previous op.)/trochanteric problems, limb/unclear pain/wound complication (rupture, granuloma)/other)[26]
**Orthopaedic arthroplasty registries – knees**					
Danish Knee Arthroplasty Register	N/R	N/A	N/R	N/R	N/R
Swedish Knee Arthroplasty Register	Cumulative revision rates[72];<br>Up to 10-years[72].	N/A	Fracture, infection, instability, loosening, patella, progress, wear, and 'other'[72]	Pre-operatively and 1 year post-operatively (EQ5D/EQ-VAS/KOOS/OMERACT-OARSI/VAS-knee pain/VAS-satisfaction with the surgery)[72]	Adverse events within 90-days after primary knee replacement (adverse surgical events/all/cardiovascular events/death within 90-days/other)[72]

Table S8B: Orthopaedic registries – Domain 'Safety & performance'

	Frequency of feedback	Level of feedback provided	Feedback (time period)	Outlier reports/procedures	Accessibility of results	Definition of outlier	Number of outliers identified
**Orthopaedic arthroplasty registries – combined**							
Croatian Register of endoprothesis	N/R	N/R	N/R	N/R	N/R	N/R	N/R
German Arthroplasty Register	Annually[45]	Medical device level[45]	1-, 2-, 3-, 4-, 5-years[45]	N/R (revisions per implant are reported; statistical testing unknown)[45]	Publicly available[45]	N/R	N/R
Finnish Arthroplasty Register	Annually[3]	Hospital- and medical device level[3]	1-, 3-, 5-years (hospital level) and 1-, 3-, 5-, 7-, 10 years (medical device level)[3]	N/R (revisions per implant and hospital are reported; statistical testing unknown)[3]	Publicly available[3]	N/R	N/R
Irish National Orthopaedic Register	Annually and quarterly[48]	N/R	N/R	N/R	N/R	N/R	N/R
Lithuanian Arthroplasty Register	N/R	N/R	N/R	N/R	N/R	N/R	N/R
Dutch Arthroplasty Register	Annually[51]	Hospital- and medical device/ level[51]	1- and 5-years[51]	Revision outlier procedures for hospitals and implants[6, 51]	Members and individual hospitals[6]	N/R	N/R
Hungarian Arthroplasty Register	N/R	N/R	N/R	N/R	N/R	N/R	N/R
Norwegian Arthroplasty Register	Annually[33]	Hospital- and medical device level[33]	10-years (hospital level) and 3- and 10-years (medical device level)[33]	N/R (durability of replacements per hip implant and percentage non-revised standard hip patients per hospital are reported; statistical testing unknown)[33]	Publicly available[33]	N/R	N/R
Nordic Arthroplasty Register Association	N/R	N/R	N/R	N/R	N/R	N/R	N/R

National Joint Registry for England, Wales, Northern Ireland, the Isle of Man, and the States of Guernsey	Annually[56]	Hospital-, medical device-, and surgeon-level[56]	Medical devices: 1-, 3-, 5-, 10-, 15-, 17 years[56], Hospitals and surgeons: 1- and 3-year[90]	Revision outlier performances for hospitals, implants, and surgeons[90]	Publicly available[56, 90]	- Hospitals: outside the range based on the expected range of performance for hospitals[90]<br><br>- Implants: having a more than twice prothesis time incident rate when compared to the group, allowing for confidence intervals[90]<br><br>- Surgeon: Outside 99.8% control limits of funnel plot (analysing 90-day mortality following THA and TKA)[90]	Hospitals performing both THA and TKA:<br>- 201 positive outlier hospitals and 7 negative outlier hospitals on compliance;<br>- 218 positive outlier hospitals and 6 negative outlier hospitals on revision compliance;<br>- 257 positive outlier hospitals and 38 negative outlier hospitals on consent;<br>- 319 positive outlier hospitals and 8 negative outlier hospitals on valid NHS number;<br>- 198 positive outlier hospitals and 79 negative outlier hospitals on time taken to enter data[90]<br><br>Hospitals performing THA:<br>- 7 positive outlier hospitals on compliance;<br>- 6 positive outlier hospitals on revision compliance;<br>- 5 positive outlier hospitals and 3 negative outlier hospitals on consent;<br>- 5 positive outlier hospitals and 2 negative outlier hospitals on valid NHS number;<br>- 3 positive outlier hospitals and 3 negative outlier hospitals on time taken to enter data[90]

							Hospitals performing TKA:<br>- 2 positive outlier hospitals on compliance;<br>- 2 positive outlier hospitals on revision compliance;<br>- 1 negative outlier hospital on consent;<br>- 1 positive outlier hospitals on valid NHS number;<br>- 1 positive outlier hospitals and 1 negative outlier hospital on time taken to enter data[90]<br><br>- 31 component combinations (THA); 12 THA cups; 13 THA stems, and 17 TKA implants<br><br>- No outlier surgeons identified[90]
Belgian National Arthroplasty Register	N/R	N/R	N/R	N/R	N/R	N/R	N/R
Catalan Arthroplasty Register	N/R	N/R	N/R	N/R	N/R	N/R	N/R
National Arthroplasty Registry of Slovenia	Annually[60, 61]	Medical device level[60, 61]	N/R	N/R (revisions per implant are reported; only numbers are shown, no statistical testing)[60, 61]	Publicly available[60, 61]	N/R	N/R
Italian Arthroplasty Registry	N/R	N/R	N/R	N/R	N/R	N/R	N/R
Emilia-Romagna Region Arthroplasty Register	Annually[63]	Medical device level[63]	5-years[63]	N/R (revisions per implant are reported; statistical testing unknown)[63]	Publicly available[63]	N/R	N/R
Romanian National Arthroplasty Register	N/R	Hospital- and medical device level[77]	2-, 5- and 10-years (hospital level) and range ±8- to	N/R (implant survival & reoperations per hospital and implant are reported; statistical testing unknown)[77]	Publicly available[77]	N/R	N/R

			13-years (medical device level)[77]				
Portuguese National Arthroplasty Register	N/R	Hospital level[38]	N/R	N/R (list of hospitals in which revisions were performed; statistical testing unknown)[38]	Publicly available[38]	N/R	N/R
Scottish Arthroplasty Project Joint Registry	Annually[20]	Hospital level[80]	30- and 90 days, 1-, 3-, and 5 years[80]	Hospitals between 2-3SD are alerted to their position and advised to investigate this internally, hospitals that have exceeded >3 SD above the mean are alerted as well and required to conduct investigations on this issue[80]	N/R	2-3SD above the mean and >3SD above the mean[80]	2 hospitals (hip AMI within 30-days), 1 hospital (hip ARF within 30-days), 2 hospitals (hip CVA within 30-days), 2 hospitals (hip infection within 1 year), 2 hospitals (hip mortality within 90-days), 2 hospitals (hip revision within 1-year), 1 hospital (hip revision within 5-years), 3 hospitals (knee ARF within 30-days), 1 hospital (knee infection within 1-year), 2 hospitals (knee mortality within 90-days), 1 hospital (knee revision within 1-year), 2 hospitals (knee revision within 5-years)[80]
Slovakian National Arthroplasty Register	N/R	Hospital- and medical device level[65]	N/R	Revisions per implant are reported including relative risks on revision; RR >5% are marked in orange and >10% are marked in red, revisions per hospital are reported[65]	Publicly available[65]	RR >5% and RR >10%[65]	49 component combinations (THA) of which 20 uncemented, 13 cemented, 8 hybrids, and 8 reverse hybrids[65]
Swiss Arthroplasty Register	Annually and quarterly[22]	Medical device level[39]	2-years[39]	Revision per implant are reported outlier status is set at	Publicly available[39]	Revision rates of more than twice	12 component combinations (THA) of which 9 uncemented, 3

				more than twice than the relevant group average[39]		compared to the relevant group[39]	hybrids, and 3 TKA implants (all component fixations)[39]
**Orthopaedic arthroplasty registries – hips**							
Czech Republic Arthroplasty Register	N/R	N/R	N/R	N/R	N/R	N/R	N/R
French Arthroplasty Register	Annually[79]	Medical device level[79]	N/R	Revision rate per 100 observed component years per implants are reported, implants >1.3 revision rate per 100 observed component years are considered to raise concern[79]	Publicly available[79]	Revision rates of >1.3 per 100 observed component years[79]	3 cups (THA); 1 uncemented cup; 1 cemented cup; 1 double mobility cup uncemented, and 2 uncemented stems (THA)[79]
Danish Hip Arthroplasty Register	Annually[25]	Hospital level[67]	7-days, 2- and 5-years[67]	Blood transfusion rates within 7-days after primary THA due to OA & rehospitalization after primary THA & rehospitalization after primary THA due to OA & rehospitalization after primary THA due to fracture & reprocedures within 2-years after primary THA & reprocedures within 2-years after primary THA due to OA & reprocedures within 2-years after primary THA due to fracture & 5-years mortality after primary THA & 5-years mortality after primary THA due to OA per hospital are reported (funnel plots)[67]	Publicly available[67]	Outside 95% control limits of funnel plot[67]	2 hospitals (rehospitalization after primary THA), 3 hospitals (rehospitalization after primary THA due to fracture), 4 hospitals (reprocedures within 2-years after primary THA), 3 hospitals (reprocedures within 2-years after primary THA due to OA), 4 hospitals (5-years mortality after primary THA), 3 hospitals (5 years mortality after primary THA due to OA)[67]
Swedish Hip Arthroplasty Register	Annually[26]	Hospital-, medical device- and surgeon level[26]	90-days and 2-years (surgeon level), 2-, 5-,	Revision outlier procedures for surgeons (e.g., above the 95% CI for adverse events within 90-	Publicly available as well as	Above the 95% CI for adverse events	1 surgeon for reoperations within 2-years (data 2016), 8 hospitals on 5-years implant survival, 7

			10-years (hospital level) and <2-, 2-, and 5-years (medical device level)[26]	days & reoperations within 2-years after hip procedure), 30-days adverse events, 90-days adverse events, 5- and 10-years implant survival per hospital (95% CI interval for each hospital above the average 30- and 90-days adverse events rates and survival rate), N/R (reoperations within 2-years after primary procedure per hospital are reported; statistical testing unknown), revision on 10-year survival (based on log-rank tests with significance set at p<0,0005 compared with control group)[26]	individual reports to hospitals[26]	within 90-days & reoperations within 2-years after hip procedure based on the average during that period (surgeon level) and 95% CI per hospital above the average 5- and 10-years survival rates[26]	hospitals on 10-years implant survival, 14 hospitals on 30-days adverse events (elective patients), 16 hospitals on 90-days adverse events (elective patients), 5 hospitals on 30-days adverse events (standard patients), 6 hospitals on 90-days adverse events (standard patients), 5 hospitals on 30-days adverse events (fracture patients), 2 hospitals on 90-days adverse events (fracture patients), 7 hospitals on 30-days adverse events (after first reoperation), 6 hospitals on 90-days adverse events (after first operation), 4 hospitals on 30-days adverse events (second or later operation), 2 hospitals on 90-days adverse events (second or later operation), 6 hospitals on 30-days adverse events (after first revision), 6 hospitals on 90-days adverse events (after first revision), 3 hospitals on 30-days adverse events (after second or later revision), 3 hospitals on 90-days adverse events (after second or later revision), 7 THA cups, and 1 THA stem[26]
Danish Knee Arthroplasty Register	N/R	N/R	N/R	N/R	N/R	N/R	N/R

Swedish Knee Arthroplasty Register	Annually[72]	Hospital- and medical device level[72]	90-days (hospital level), N/R (medical device level)[72]	N/R (all events/adverse surgical events/cardiovascular events/death/other events within 90-days per hospital (risk/1000) are reported; statistical testing unknown), risk of revision per implant are reported (marked in red with higher risk ratio, based on risk of revision (RR); the PFC-Sigma MBT implant is used as reference), relative risk of revision per hospital are reported (marked in red with higher risk ratio; statistical testing unknown)[72]	Publicly available as well as individual reports to hospitals[72]	N/R	3 TKA implants (general risk of revision), 4 TKA implants (risk of revision when infection is not considered to be a revision), 10 hospitals (relative risk of revision), 9 hospitals (relative risk of revision when infection is not considered to be a revision)[72]

References, orthopaedic

1. Niederländer C, Wahlster P, Kriza C, Kolominsky-Rabas P. Registries of implantable medical devices in Europe. Health Policy. 2013;113(1-2):20-37.

2. Endoprothesenregister Deutschland. https://www.eprd.de/de/ (accessed on March 24. 2022)

3. Finnish Arthroplasty Register. https://www.thl.fi/far/ (accessed on March 24. 2022)

4. Irish National Orthopaedic Register. https://www.noca.ie/audits/irish-national-orthopaedic-register (accessed on March 24. 2022)

5. Sąnarių endoptrotezuotojų asociacija. http://lsed.lt/ (accessed on March 24. 2022)

6. Landelijke Registratie Orthopedische Implantaten. https://www.lroi.nl/ (accessed on March 24. 2022)

7. Magyar Orthoped Tarsasag. http://www.ortopedtarsasag.hu/ (accessed on March 24. 2022)

8. Helse Bergen Haukeland Universitetssjukehus. https://nrlweb.ihelse.net/ (accessed on March 24. 2022)

9. Badawy M, Fenstad AM, Bartz-Johannessen CA, Indrekvam K, Havelin LI, Robertsson O, et al. Hospital volume and the risk of revision in Oxford unicompartmental knee arthroplasty in the Nordic countries -an observational study of 14,496 cases. BMC Musculoskelet Disord. 2017;18(1):388.

10. National Joint Registry. https://www.njrcentre.org.uk/ (accessed on March 24. 2022)

11. ORTHOpride. https://www.ehealth.fgov.be/nl/egezondheid/beroepsbeoefenaars-in-de-gezondheidszorg/qermidorthopride (accessed on March 24. 2022)

12. Allepuz A, Martínez O, Tebé C, Nardi J, Portabella F, Espallargues M. Joint registries as continuous surveillance systems: the experience of the Catalan Arthroplasty Register (RACat). J Arthroplasty. 2014;29(3):484-90.

13. Catalan Arthroplasty Register. Catalan Arthroplasty Register: Third report (2005-2014). January 2017. Accessible: https://scientiasalut.gencat.cat/bitstream/handle/11351/3388/catalan_arthroplasty_register_third_report_2005-2014_2017.pdf;jsessionid=4F83828F62E3CB0B7B2B672561062C94?sequence=1

14. Ortopedska bolnisnica Valdoltra. https://www.ob-valdoltra.si/ (accessed on March 24. 2022)

15. Italian Arthroplasty Registry. https://riap.iss.it/riap/ (accessed on March 24. 2022)

16. Register of the Orthopaedic Prosthetic Implant. http://www.ior.it/en/curarsi-al-rizzoli/register-orthopaedic-prosthetic-implants (accessed on March 24. 2022)

17. Romanian Arthroplasty Register. https://www.rne.ro/ (accessed on March 24. 2022)

18. Registo Portugues de Artroplastias. http://www.rpa.spot.pt/ (accessed on March 24. 2022)

19. Makaram N, Woods L, Beattie N, Roberts SB, Macpherson GJ. Long-term outcomes following total hip and total knee arthroplasty in patients with Paget's disease of bone (PDB) - A national study. Surgeon. 2020;18(6):335-43.

20. The Scottish Arthroplasty Project. https://www.arthro.scot.nhs.uk/ (accessed on March 24. 2022)

21. Slovenský artroplastický register. https://sar.mfn.sk/ (accessed on March 24. 2022)

22. Schweizerisches implantat-register. https://www.siris-implant.ch/ (accessed on March 24. 2022)

23. Vavřík P, Landor I, Popelka S, Fialka R, Hach J. The National Register of Joint Replacements of the Czech Republic. Acta Chir Orthop Traumatol Cech. 2014;81 Suppl:3-68.
24. SOFCOT. https://www.sofcot.fr/ (accessed on March 24. 2022)
25. The Danish Hip Arthroplasty Register. www.dhr.dk (accessed on March 24. 2022)
26. Swedish Hip Arthroplasty Register. Swedish Hip Arthroplasty Register Annual report 2019. 2021. Accessible: https://registercentrum.blob.core.windows.net/shpr/r/VGR_Annual-report_SHAR_2019_EN_Digital-pages_FINAL-ryxaMBUWZ_.pdf
27. Svenska Ledprotesregistret. https://shpr.registercentrum.se/ (accessed on March 24. 2022)
28. Cordtz RL, Zobbe K, Højgaard P, Kristensen LE, Overgaard S, Odgaard A, et al. Predictors of revision, prosthetic joint infection and mortality following total hip or total knee arthroplasty in patients with rheumatoid arthritis: a nationwide cohort study using Danish healthcare registers. Ann Rheum Dis. 2018;77(2):281-8.
29. The Swedish Knee Arthroplasty Register. http://www.myknee.se/ (accessed on March 24. 2022)
30. Jansson V, Grimberg A, Melsheimer O, Perka C, Steinbrück A. Orthopaedic registries: the German experience. EFORT Open Rev. 2019;4(6):401-8.
31. Aaltonen KJ, Virkki LM, Jämsen E, Sokka T, Konttinen YT, Peltomaa R, et al. Do biologic drugs affect the need for and outcome of joint replacements in patients with rheumatoid arthritis? A register-based study. Semin Arthritis Rheum. 2013;43(1):55-62.
32. Cebatorius A, Robertsson O, Stucinskas J, Smailys A, Leonas L, Tarasevicius S. Choice of approach, but not femoral head size, affects revision rate due to dislocations in THA after femoral neck fracture: results from the Lithuanian Arthroplasty Register. Int Orthop. 2015;39(6):1073-6.
33. The Norwegian Arthroplasty Register. Norwegian National Advisory Unit on Arthroplasty and Hip Fractures. Annual report 2021. Accessible: https://www.researchgate.net/publication/356998659_Annual_report_2021_Norwegian_National_Advisory_Unit_on_Arthroplasty_and_Hip_Fractures_Norwegian_Arthroplasty_Register_Norwegian_Cruciate_Ligament_Register_Norwegian_Hip_Fracture_Register_Norwegian_Pae
34. Rant L. Building The National Arthroplasty Registry of Slovenia in the Parent Project. November 2015.
35. Torre M, Romanini E, Zanoli G, Carrani E, Luzi I, Leone L, et al. Monitoring Outcome of Joint Arthroplasty in Italy: Implementation of the National Registry. Joints. 2017;5(2):70-8.
36. Italian Arthroplasty Registry (RIAP). Addendum to the Annual Report 2019. October 2020. Accessible: https://riap.iss.it/riap/en/activities/reports/2020/11/23/2019-riap-annual-report-english-addendum/
37. Bordini B, Stea S, Falcioni S, Ancarani C, Toni A. Unicompartmental knee arthroplasty: 11-year experience from 3929 implants in RIPO register. Knee. 2014;21(6):1275-9.
38. Registo Portugues de Artroplastias (RPA). 2013 Annual Report. Accessible: http://www.rpa.spot.pt/getdoc/c3d0a244-c056-4949-a50b-07d0fdeac2b9/RPA-Report-2013.aspx

39. Schweizerisches implantat-register registre suisse des implants (SIRIS). Swiss National Hip & Knee Joint Registry Report 2020. Accessible: https://www.anq.ch/wp-content/uploads/2021/01/ANQakut_SIRIS_Hips-Knee_Annual-Report_2020.pdf
40. Delaunay C, Hamadouche M, Girard J, Duhamel A. What are the causes for failures of primary hip arthroplasties in France? Clin Orthop Relat Res. 2013;471(12):3863-9.
41. Chatziagorou G, Lindahl H, Garellick G, Kärrholm J. Incidence and demographics of 1751 surgically treated periprosthetic femoral fractures around a primary hip prosthesis. Hip Int. 2019;29(3):282-8.
42. Cnudde P, Rolfson O, Nemes S, Kärrholm J, Rehnberg C, Rogmark C, et al. Linking Swedish health data registers to establish a research database and a shared decision-making tool in hip replacement. BMC Musculoskelet Disord. 2016;17(1):414.
43. El-Galaly A, Kappel A, Nielsen PT, Jensen SL. Revision Risk for Total Knee Arthroplasty Converted from Medial Unicompartmental Knee Arthroplasty: Comparison with Primary and Revision Arthroplasties, Based on Mid-Term Results from the Danish Knee Arthroplasty Registry. J Bone Joint Surg Am. 2019;101(22):1999-2006.
44. Pedersen AB, Mehnert F, Odgaard A, Schrøder HM. Existing data sources for clinical epidemiology: The Danish Knee Arthroplasty Register. Clin Epidemiol. 2012;4:125-35.
45. Endoprothesenregister Deutschland (EPRD). The German Arhtroplatsy Registry Annual Report 2020. 2020. Accessible: https://www.eprd.de/fileadmin/user_upload/Dateien/Publikationen/Berichte/AnnualReport2020-Web_2021-05-11_F.pdf
46. Blömer W, Steinbrück A, Schröder C, Grothaus FJ, Melsheimer O, Mannel H, et al. A new universal, standardized implant database for product identification: a unique tool for arthroplasty registries. Arch Orthop Trauma Surg. 2015;135(7):919-26.
47. Karvonen M, Laaksonen I, Pulkkinen P, Eskelinen A, Haapakoski J, Puhto AP, et al. Implant Survival of Constrained Acetabular Device in Primary Total Hip Arthroplasty Based on Data From the Finnish Arthroplasty Register. J Arthroplasty. 2020;35(1):219-23.
48. Irish National Orthopaedic Register (INOR). IRISH NATIONAL ORTHOPAEDIC REGISTER FIRST REPORT. 2021. Accessible: https://www.noca.ie/documents/irish-national-orthopaedic-register-first-report
49. Stucinskas J, Grigaitis K, Smailys A, Robertsson O, Tarasevicius S. Bipolar hemiarthroplasty versus total hip arthroplasty in femoral neck fracture patients: results from Lithuanian Arthroplasty Register. Hip Int. 2021;31(5):691-5.
50. Van Steenbergen LN, Mäkelä KT, Kärrholm J, Rolfson O, Overgaard S, Furnes O, et al. Total hip arthroplasties in the Dutch Arthroplasty Register (LROI) and the Nordic Arthroplasty Register Association (NARA): comparison of patient and procedure characteristics in 475,685 cases. Acta orthopaedica. 2021;92(1):15-22.

51. Dutch Arthroplasty Register (LROI). Online LROI annual report 2020 - PDF. 2020. Accessible: https://www.lroi-report.nl/app/uploads/2021/03/PDF-LROI-annual-report-2020.pdf
52. van Steenbergen LN, Denissen GA, Spooren A, van Rooden SM, van Oosterhout FJ, Morrenhof JW, et al. More than 95% completeness of reported procedures in the population-based Dutch Arthroplasty Register. Acta Orthop. 2015;86(4):498-505.
53. Blågestad T, Nordhus IH, Grønli J, Engesæter LB, Ruths S, Ranhoff AH, et al. Prescription trajectories and effect of total hip arthroplasty on the use of analgesics, hypnotics, antidepressants, and anxiolytics: results from a population of total hip arthroplasty patients. Pain. 2016;157(3):643-51.
54. Robertsson O, Bizjajeva S, Fenstad AM, Furnes O, Lidgren L, Mehnert F, et al. Knee arthroplasty in Denmark, Norway and Sweden. A pilot study from the Nordic Arthroplasty Register Association. Acta orthopaedica. 2010;81(1):82-9.
55. Havelin LI, Engesaeter LB, Espehaug B, Furnes O, Lie SA, Vollset SE. The Norwegian Arthroplasty Register: 11 years and 73,000 arthroplasties. Acta Orthop Scand. 2000;71(4):337-53.
56. National Joint Registry (NJR). National Joint Registry 18th Annual Report 2021 2021. Accessible: https://reports.njrcentre.org.uk/
57. Orthopride. Belgian Hip and Knee Arthroplasty Registry Annual Report 2018. 2019.
58. ORTHOpride. ORTHOpedic Prosthesis Identification Data Electronic Registry Gebruikershandleiding. Accessible: https://docplayer.nl/58485798-Orthopride-orthopedic-prosthesis-identification-data-electronic-registry-gebruikershandleiding.html
59. Arias-de la Torre J, Smith K, Dregan A, Valderas JM, Evans JP, Prieto-Alhambra D, et al. Impact of comorbidity on the short- and medium-term risk of revision in total hip and knee arthroplasty. BMC Musculoskelet Disord. 2020;21(1):447.
60. Register endoprotetike Slovenije. The National Arthroplasty Registry of Slovenia (RES) 2st Annual Report - Hip Replacement (data from 2020). 2021. Accessible: https://www.ob-valdoltra.si/sites/www.ob-valdoltra.si/files/upload/files/2st_res_annual_report-hipreplacement_2021_0.pdf
61. Register endoprotetike Slovenije. The National Arthroplasty Registry of Slovenia (RES) 2st Annual Report - Knee Replacement (data 2020). 2021. Accessible: https://www.ob-valdoltra.si/sites/www.ob-valdoltra.si/files/upload/files/2nd_res_annual_report_-_knee_replacement_data_2020.pdf
62. Italian Arthroplasty Registry (RIAP). Addendum to the Annual Report 2019. October 2020. Accessible: https://riap.iss.it/riap/en/activities/reports/2020/11/23/2019-riap-annual-report-english-addendum/
63. REPORT of R.I.P.O. Regional Register of Orthopaedic Prosthetic Implantology. OVERALL DATA HIP, KNEE AND SHOULDER ARTHROPLASTY IN EMILIA-ROMAGNA REGION (ITALY) 2000-2018. 2020. Accessible: https://ripo.cineca.it/authzssl/pdf/report_RIPO_2018_ENG.pdf

64. Romanian Arthroplasty Register (RAR). Value of different types of literature and requirements for running a successful Arthroplasty Register 2016. Accessible: https://www.yumpu.com/en/document/view/43437948/development-of-the-romanian-register-ear-efort
65. Slovakian arthroplatsy Register. SURVIVAL ANALYSIS OF TOTAL HIP AND KNEE REPLACEMENT IN SLOVAKIA 2003-2011. 2013. Accessible: https://www.researchgate.net/publication/296018643_Slovakian_arthroplasty_register_Survival_analysis_of_total_hip_and_knee_replacement_in_Slovakia_2003-2011
66. SwissRDL. French hip arthroplasty (SOFCOT) https://www.swissrdl.unibe.ch/registries/french_hip_arthroplasty_sofcot/index_eng.html (accessed on April 1. 2022)
67. Dansk Hoftealloplastik Register Danish Hip Arthroplasty Register. National arsrapport 2020. 2020. Accessible: http://danskhoftealloplastikregister.dk/wp-content/uploads/2020/11/dhr-aarsrapport-2020_til_offentliggoerelse-1.pdf
68. Glassou EN, Pedersen AB, Hansen TB. Risk of re-admission, reoperation, and mortality within 90 days of total hip and knee arthroplasty in fast-track departments in Denmark from 2005 to 2011. Acta Orthop. 2014;85(5):493-500.
69. Kärrholm J, Rogmark C, Naucler E, Nåtman J, Vinblad J, Mohaddes M, et al. Swedish Hip Arthroplasty Register Annual report 20192021.
70. Daugberg L, Jakobsen T, Nielsen PT, Rasmussen M, El-Galaly A. A projection of primary knee replacement in Denmark from 2020 to 2050. Acta Orthop. 2021;92(4):448-51.
71. El-Galaly A, Grazal C, Kappel A, Nielsen PT, Jensen SL, Forsberg JA. Can Machine-learning Algorithms Predict Early Revision TKA in the Danish Knee Arthroplasty Registry? Clin Orthop Relat Res. 2020;478(9):2088-101.
72. The Swedish Knee Arthroplasty Register. Annual Report 2020. 2020. Accessible: https://www.researchgate.net/publication/345602419_The_Swedish_Knee_Arthroplasty_Register_-_Annual_report_2020
73. Ogino D, Kawaji H, Konttinen L, Lehto M, Rantanen P, Malmivaara A, et al. Total hip replacement in patients eighty years of age and older. J Bone Joint Surg Am. 2008;90(9):1884-90.
74. Terteliene E, Grigaitis K, Robertsson O, Stucinskas J, Tarasevicius S, Porvaneckas N, et al. Inadequate evaluation and management of suspected -infections after TKA surgery in Lithuania: a retrospective study of 2,769 patients with 2-year follow-up. Acta Orthop. 2019;90(4):373-6.
75. Catalan Arthroplasty Register. Catalan Arthroplasty Register: Third report (2005-2014). January 2017. Accessible: https://scientiasalut.gencat.cat/bitstream/handle/11351/3388/catalan_arthroplasty_register_third_report_2005-2014_2017.pdf;jsessionid=4F83828F62E3CB0B7B2B672561062C94?sequence=1

76. Schelde AB, Petersen J, Jensen TB, Gromov K, Overgaard S, Olesen JB, et al. Thromboembolic and bleeding complications following primary total knee arthroplasty : a Danish nationwide cohort study. Bone Joint J. 2021;103-b(10):1571-7.
77. Romanian Arthroplasty Register. Annual Report 2011. Accessible: https://www.rne.ro/rnemedia/download/RNE_AnnualReport_2001-2011_en_oct2013.pdf
78. Swiss National Hip & Knee Joint Registry. Report 2020 Annual Report of the SIRIS Registry Hip & Knee, 2012 - 2019. 2020. Accessible: https://www.anq.ch/wp-content/uploads/2021/01/ANQakut_SIRIS_Hips-Knee_Annual-Report_2020.pdf
79. SoFCOT. SoFCOT Total Hip Arthroplasty Register Biennial Report 2020 2006-2019. 2020. Accessible: https://www.sofcot.fr/files/medias/documents/2020%20global%20biennial_report_2020_final.pdf
80. Scottish Arthroplasty Project Joint Registry. Statistical report https://publichealthscotland.scot/publications/scottish-arthroplasty-project/scottish-arthroplasty-project-14-september-2021/dashboard/2021 (accessed on April 1. 2022)
81. Puolakka TJ, Pajamäki KJ, Halonen PJ, Pulkkinen PO, Paavolainen P, Nevalainen JK. The Finnish Arthroplasty Register: report of the hip register. Acta Orthop Scand. 2001;72(5):433-41.
82. LROI. LROI Report 2013. Insight into Quality & Safety. Annual Report of the Dutch Arhtroplasty Register (Landelijke Registratie Orthopedische Implantaten) 2013. https://www.lroi.nl/base/downloads/lroi-report-2013-english.pdf (accessed on February 27. 2022)
83. Mäkelä KT, Furnes O, Hallan G, Fenstad AM, Rolfson O, Kärrholm J, et al. The benefits of collaboration: the Nordic Arthroplasty Register Association. EFORT Open Rev. 2019;4(6):391-400.
84. Sciensano. General Orthopride Hip project information. https://docs.healthdata.be/documentation/quality-electronic-registration-medical-acts-implants-and-devices-orthopedic-65 (accessed on February 27. 2022)
85. Registo Portugues de Artroplastias (RPA). 1st Annual Report June 2009 – May 2010. Accessible: file:///C:/Users/lahoogervorst/Downloads/1106-001-172_Relatorio-ING%20(6).pdf
86. Slovak Arthroplasty Register. https://sar.mfn.sk/ (accessed on February 27. 2022)
87. Institute of Health Information and Statistics of the Czech Republic. National register of joint replacements. https://www.uzis.cz/index.php?pg=registry-sber-dat--narodni-zdravotni-registry--narodni-registr-kloubnich-nahrad (accessed on February 27. 2022)
88. Gundtoft PH, Varnum C, Pedersen AB, Overgaard S. The Danish Hip Arthroplasty Register. Clin Epidemiol. 2016;8:509-14.
89. Swedish Hip Arthroplasty Register. Swedish Hip Arthroplasty Register Annual report 2012. Accessible: https://registercentrum.blob.core.windows.net/shpr/r/Annual-report-2012-HJBqtLpig.pdf
90. Slovakian National Arthroplasty Register. Pracoviská SAR. https://sar.mfn.sk/ (accessed on February 27. 2022)
